# Nickel Catalyzed Carbonylation/Carboxylation Sequence via Double CO_2_ Incorporation

**DOI:** 10.1021/acs.orglett.3c02394

**Published:** 2023-09-05

**Authors:** Riccardo Giovanelli, Lorenzo Lombardi, Riccardo Pedrazzani, Magda Monari, Marta Castiñeira Reis, Carlos Silva López, Giulio Bertuzzi, Marco Bandini

**Affiliations:** † Dipartimento di Chimica “Giacomo Ciamician”, Alma Mater Studiorum − Università di Bologna, Via P. Gobetti 85, 40129, Bologna, Italy; ‡ Center for Chemical Catalysis − C3, Dipartimento di Chimica “Giacomo Ciamician”, Alma Mater Studiorum − Università di Bologna, Via P. Gobetti 85, 40129, Bologna, Italy; § Departamento de Química Orgánica, Universidad de Vigo, As Lagoas-Marcosende, 36310, Vigo, Spain

## Abstract

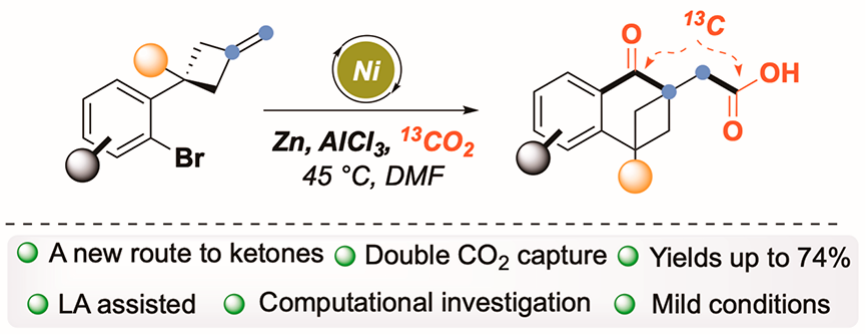

A carbonylation–carboxylation synthetic sequence, via double CO_2_ fixation, is described. The productive merger of a Ni-catalyzed cross-electrophile coupling manifold, with the use of AlCl_3_, triggered a cascade reaction with the formation of three consecutive C–C bonds in a single operation. This strategy traces an unprecedented synthetic route to ketones under Lewis acid assisted carbon dioxide valorization. Computational insights revealed a unique double function of AlCl_3_, and labeling (^13^CO_2_) experiments validate the genuine incorporation of CO_2_ in both functional groups.

The fixation of CO_2_ into value-added long-term organic vectors is consolidating a prominent role in many scientific disciplines with direct application outlets, like energy storage and smart material development.^1,2^ Catalytic *carboxylation* reactions based on CO_2_ (i.e., C–C bond forming protocols)^3^ strongly contributed to this flourishing scenario, enabling mild conditions and site-selective processes to be effectively combined. Concomitantly, CO_2_ is emerging as a chemical surrogate of carbon monoxide for *carbonylation* procedures such as hydroformylations of amines, aryl halides, and alkenes, and redox-neutral cyclizations via amino- or alkoxycarbonylation (Figure 1a).^4^ However, the use of CO_2_ in the carbonylative synthesis of ketones (i.e., formation of two C–C bonds) has not found a general solution, and features in only a limited number of examples so far (Figure 1b). To date, two main strategies have been pursued, namely: the use of strongly nucleophilic reagents (i.e., organolithium and Grignard compounds, very limited in number)^5^ and the *in situ* CO_2_ → CO reduction and subsequent fixation.^6,7^ The narrow functional group tolerance, arising from the requirement of harsh organometallics or strong reductive conditions, substantially limits the synthetic perspective of these approaches. In line with this background, we reasoned that a metal catalyzed CO_2_ based carboxylation and consequent electrophilic activation of the carboxylic moiety could pave the way for a conceptually new synthetic approach to ketones from CO_2_, by means of a final site-specific insertion step. The activation of carboxylic compounds toward nucleophilic acylation reactions has been a longstanding topic in organic synthesis, accounting nowadays for multiple extraordinary solutions.^8^ Among them, we were attracted by the possibility to employ oxophilic *Lewis acids* (LAs, as oxygen atom scavengers)^9^ that have been very recently utilized in direct borylation reactions of aromatic carboxylic compounds under nickel catalysis.^10^ Interestingly, the employment of a LA could also play the additional role of assisting the initial carboxylation event via electrophilic activation of CO_2_. As a matter of fact, despite the scarce Lewis basicity of carbon dioxide, an acid–base adduct between AlCl_3_ and CO_2_ has been identified, fully characterized (i.e., O=C=O···AlCl_3_), and successfully employed as an activated form of carbon dioxide in the direct carboxylation reaction of unactivated arenes.^11^ Our recent report on AlCl_3_-assisted CO_2_ fixation^12^ strongly supports the compatibility of metal (i.e., Ni)-catalyzed carboxylative cross-electrophile-couplings (XECs) with oxophilic LAs.^13^ In this scenario, we envisioned that, by subjecting an *ortho*-substituted aryl-halide, carrying a terminal π-system (**A**), to TM catalyzed reductive carboxylation in the presence of AlCl_3_, the acyloxy-aluminum species **C** could be generated. The electrophilic activation played by the aluminum coordination could trigger a metal mediated intramolecular C–C bond forming process involving the proximal olefin.

**Figure 1:**
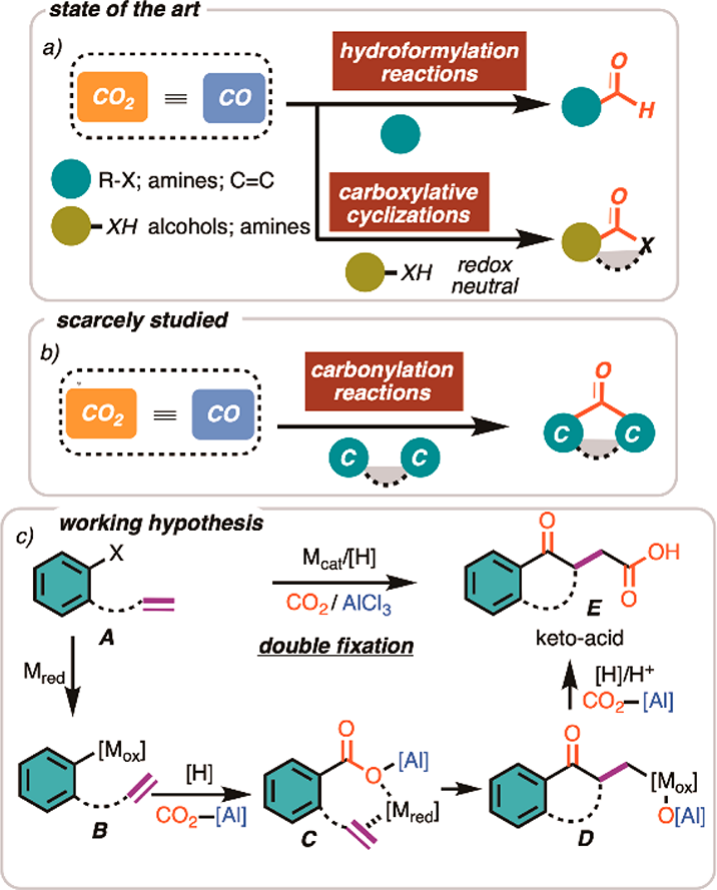
(a) State of the art on the use of “CO_2_≡CO” as C1 synthon in organic synthesis. (b) “Vacancy” on the direct use of CO_2_ for the catalytic synthesis of ketones. (c) The present working idea ([M_red_] and [M_ox_] indicate different oxidation states of the metal catalyst, [H]: reductant).

The resulting alkyl-organometallic species **D** could finalize the catalytic cycle engaging a second molecule of CO_2_, thus realizing the first example of a carbonylation/cyclization/carboxylation sequence based on carbon dioxide (Figure 1c).^14^ Synthetically valuable γ-ketoacids **E** should therefore be readily accessible under the catalytic regime. Remarkably, three carbon–carbon bonds are created in the present carbonylation/carboxylation protocol with two CO_2_ units participating in the catalytic cycle.^15^

The investigation began with the design of a proper substrate to test the working hypothesis. To this end, readily accessible alkylidenecyclobutane **1a** was elected as the model platform, displaying the aryl halide and the pendant olefin in adequate and mutual positions. Based on previous achievements by the group^12,16^ and on the well-consolidated efficiency of organo-Ni intermediates toward CO_2_-based carboxylations,^17^ we elected air-stable [Ni(*N^N*)_2_X_2_] complexes as potential (pre)catalysts for the tandem carbonylation/carboxylation reaction sequence. An extensive survey of reaction parameters, regarding the nature of the ligand, nickel counterion, solvent, reducing agent, temperature, reaction stoichiometry, and additives, was undertaken (see Tables S2–S3, section 3 for an exhaustive list of results). From this investigation, the use of preformed [Ni(**L1**)_2_Cl_2_] complex (10 mol %),^18^ AlCl_3_ as a stoichiometric additive, and Zn as reducing agent (DMF, 45 °C, 16 h, CO_2_ 1 atm) delivered the tetralone-α-acetic acid **2a** in 70% isolated yield, along with the formation of dimethyl-tetralone **2a′** (7% yield, entry 1, Table 1). No trace of ring-opening of the C4-membered ring was observed under the present conditions.

**Table 1:**
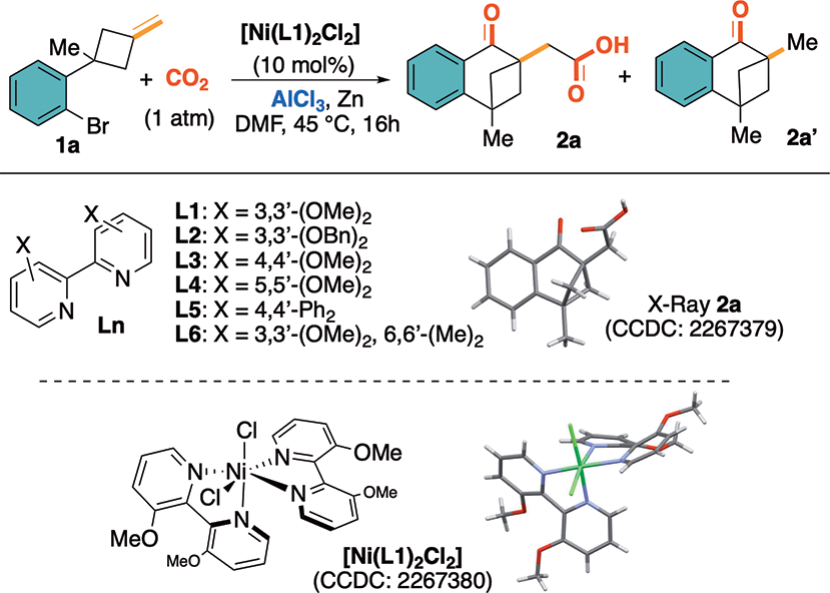
Optimization of the Reaction Conditions

Entry^a^	Deviation from optimal	Yield **2a**/**2a′** (%)^b^
1	–c	70/7
2	[Ni(**L2**)_2_Cl_2_]	64/7
3	[Ni(**L3**)_2_Cl_2_]	61/4
4	[Ni(**L4**)_2_Cl_2_]	19/–
5	[Ni(**L5**)_2_Cl_2_]	15/5
6	[Ni(**L6**)Cl_2_]	–/–
7	[Ni(**L1**)_2_I_2_]	64/10
8	[Ni(**L1**)Cl_2_]	64/5
9	without AlCl_3_	–d
10	without [Ni(**L1**)_2_Cl_2_]	–e
11	under N_2_ instead of CO_2_	–d
12	under CO instead of CO_2_	NR
13	DMA instead of DMF	41/10
14	Mn instead of Zn	60/5

a All reactions were carried out under CO_2_ atmosphere and with dry solvents.

b Isolated yields after flash chromatography.

c **1a**/[Ni(**L1**)_2_Cl_2_]/Al/Zn, 1/0.1/4.5/3 equiv, [**1a**] = 0.1 M.

d Variable amounts of proto-debrominated-**1a** (59–97%) were recorded (^1^H NMR analysis on the reaction crude).

e Variable amounts of unreacted **1a** (75–77%) were recorded in the reaction crude. See Table S2 for details. NR: no reaction.

Conclusions from the reaction optimization study can be summarized as follows: bipyridines carrying electron-donating groups (i.e., alkoxy: **L1**–**4**) proved to have the best performance among the ligands tested (see Table S3, section 3). Interestingly, the introduction of methyl groups at the 6,6′-positions (**L6**) caused a suppression of the catalytic activity (entry 6).^19^ Generally, preformed complexes guaranteed higher reproducibility in comparison to *in situ* formed ones. However, the similar chemical outcome (64% yield) obtained with the complex [Ni(**L1**)Cl_2_] (10 mol %, entry 8) led us to propose a complex having a 1:1 Ni–ligand ratio as the catalytically active one. As expected, all components, AlCl_3_, CO_2_, and the Ni complex, proved to be essential in triggering the cascade process. Their absence caused the recovery of **1a** (w/o [Ni], entry 10) or partial debromination (w/o CO_2_, entry 11). The *in situ* formation of carbon monoxide and subsequent carbonylation reaction were excluded by running the model transformation under a CO atmosphere (entry 12). Other solvents (i.e., DMA, entry 13) did not improve the formation of **2a**, and the employment of Mn powder as a reductant provided **2a** in a lower yield (60%, entry 14) with respect to Zn. Finally, AlCl_3_ showed superior activity with respect to other LAs and additives (Table S2).

With the optimized reaction conditions settled, we tackled the versatility of the protocol (Scheme 1). Therefore, a range of alkylidene-cyclobutanes **1b**–**r** were synthesized and subjected to the double CO_2_-trapping cross-coupling reaction. Electron-rich (**1b**–**h**) and electron-poor (**1i**–**j**) substituents were accommodated at different positions of the aromatic ring. Moderate to good yields were always obtained (32–74%). As a general trend, alkylidenecyclobutanes featuring electron-rich arenes performed better than those having electron-withdrawing-group-substituted ones, likely suggesting the beneficial role of more nucleophilic aryl-Ni intermediates during the catalytic cycle (*vide infra*). In addition, the procedure resulted in suitable production of γ-keto-acids starting also from precursors featuring different substitutions at the C4-position (**1l**–**r**). Here, both aliphatic (yields up to 74%) and aromatic groups (yield up to 45%) were accommodated in the final tetralone α-acetic acid scaffold. A 1 mmol scale reaction was also attempted in the presence of substrate **1a**, and gratifyingly, **2a** was obtained in 60% yield under unmodified conditions.^20^

**Scheme 1:**
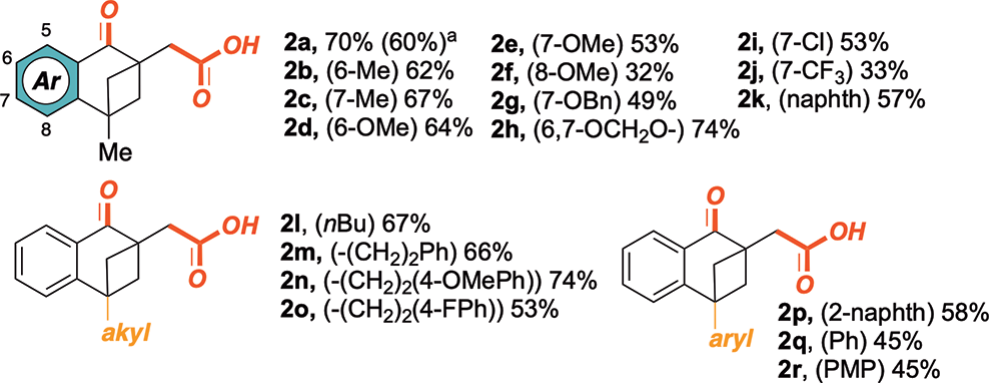
Scope of the Carbonylation/Carboxylation Reaction mmol scale reaction.

The synthetic versatility of the resulting tetralones **2** was then accounted via dedicated transformations (Scheme 2). In particular, keto-acid **2a** was effectively utilized as a precursor of nucleophilic alkyl-radicals to be employed in the site-selective alkylation of Morita–Baylis–Hillman (MBH) adduct **4**.^21^ In this approach, **2a** was initially transformed into the phthalimide-based redox-active ester (RAE) **3** and subsequently subjected to electrolysis in the presence of **4**. The desired α-alkylated cinnamate **5** was isolated in 31% yield (unoptimized conditions). The carboxylic acid group was also effectively converted into a protected amino group (**6**, 50% yield) via Curtius rearrangement. This, along with the previous transformation, shows the potential of the COOH group, generated by CO_2_-based carboxylation, as a reactive handle to introduce chemical complexity via subsequent decarboxylation. Additionally, both functional groups were effectively and synergistically employed for the generation of chemical diversity. In particular, pyridazinone **7** could be directly achieved in 74% yield by condensation of **2a** with hydrazine, and lactone **9** was prepared via an esterification/reduction/lactonization synthetic sequence.

**Scheme 2:**
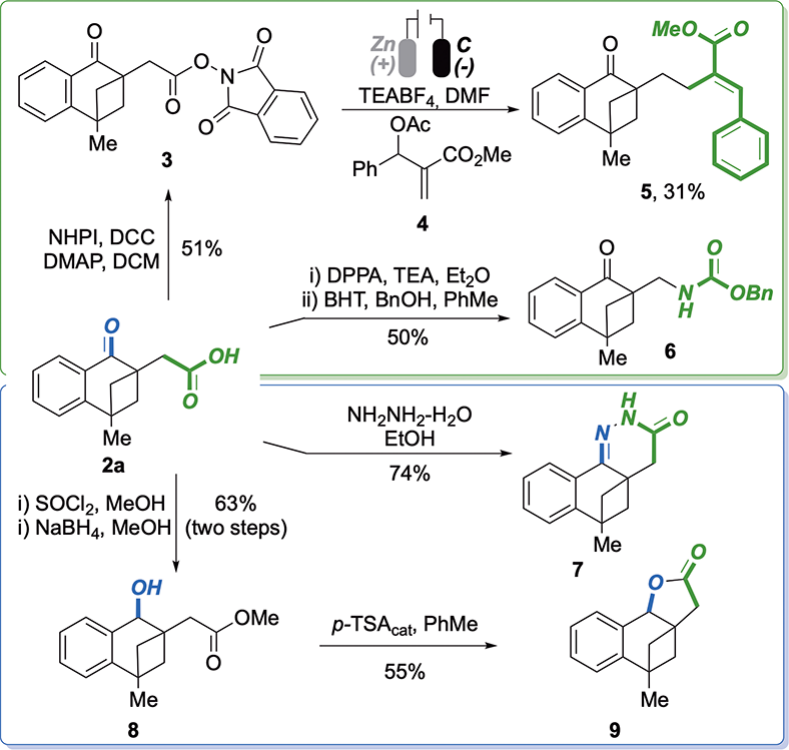
Proving the Synthetic Versatility of Tetralone **2a**

Encouraged by our findings, we conducted molecular modeling to deepen our understanding of the mechanism operating in this catalytic process (Scheme 3 top). Initially, a Zn generated Ni(I) catalyst coordinates substrate **II**, rendering complex **III**. Then, **III** can evolve via an oxidative addition, forming a Ni(III) complex (**IV**).

**Scheme 3:**
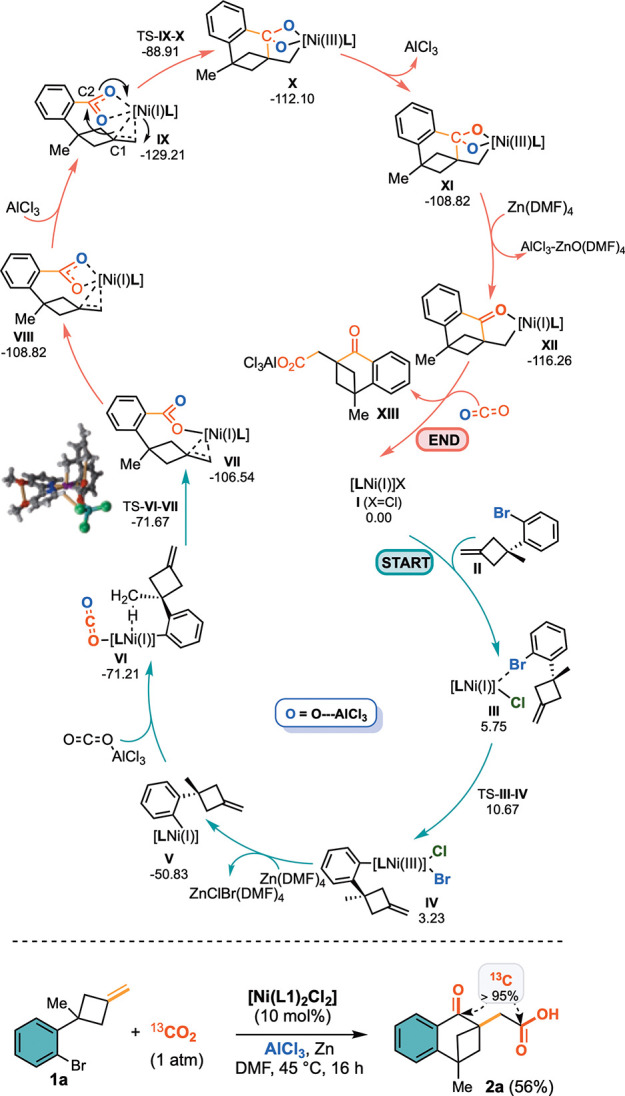
Top: Proposed Mechanism for the Formation of **2a**; Bottom: Proving the Double CO_2_ Incorporation via Labelling Experiments Relative Gibbs free energies are expressed in kcal/mol. Oxygen atoms coordinated to AlCl_3_ are depicted in red.

Additional Zn-mediated heterogeneous reduction is envisioned to transform complex **IV** to the Ni(I) analogue **V**, which coordinates an AlCl_3_-activated CO_2_ molecule (**VI**). CO_2_ inserts in the Ni–C bond, and concomitantly, the exocyclic carbon–carbon double bond coordinates the Ni(I) species, keeping the active metal in close proximity to the activated carboxylic group (species **VII** and **VIII**) and favoring the subsequent intramolecular C–C bond forming event. It is in this initial carboxylation step that the alkylidenecyclobutane methyl substituent plays a key role in order to trigger the desired reactivity.^22^ We have found a very strong Me···Ni interaction that favors the course of this reaction (these interactions are highlighted in golden in the inset of Scheme 3 top. Additional calculations in which the methyl group is absent account for ∼23 kcal/mol destabilization of **VI**.^23^ In the second stage of this catalytic cycle, the coordination of a second molecule of AlCl_3_ to the benzoate (intermediate **IX**) is crucial to trigger the intramolecular cyclization **IX** → **X** via oxidative nickel insertion onto the C=C double bond. Reduction of the resulting [Ni(III)] species occurs concomitantly to the scavenging of one oxygen atom, resulting in the alkylnickel intermediate **XII**, where the keto group is irreversibly formed. The final second carboxylation of the carbon scaffold is then operated by the [AlCl_3_···CO_2_] adduct, delivering the final keto-acid precursor **XIII**. This mechanistic picture highlights the multiple roles exerted by AlCl_3_ in the process: as a matter of fact, besides the expected electrophilic activation of CO_2_, it results pivotal in triggering the intramolecular C–C bond forming step and in scavenging the oxygen atom from the activated carboxylate for the formation of the keto group. The impact of AlCl_3_ in assisting these steps was also computationally estimated by determining the energy barriers (stabilization values over 20 kcal/mol) associated with the steps: **VI** → **VII** (aryl carboxylation) and **IX** → **X** (skeletal rearrangement) in the absence of LA-*co*-assistance.

Finally, to verify the double incorporation of CO_2_ into the final keto acids **2**, we carried out a labeled ^13^C-experiment under optimal conditions on **1a**. Satisfyingly, a complete fixation of ^13^C-carbon atoms both at the C=O and CO_2_H groups (>95% labeling by ^13^C and ^1^H NMR analyses) was recorded (Scheme 3, bottom).

In conclusion, we have reported a nickel catalyzed double incorporation of CO_2_ featuring an unprecedented strategy to synthesize ketones under Lewis acid assisted carbon dioxide valorization. The judicious choice of the catalytic system enabled concomitant exploitation of CO_2_ as a valuable carbonylating and carboxylating C1-unit. A detailed computational investigation shed light on the multiple roles of AlCl_3_, dealing with the electrophilic activation of CO_2_ and scavenging of oxygen atoms. Studies addressing the interplay of Ni-catalyzed XECs, AlCl_3_ and CO_2_, for direct access to synthetically relevant organic scaffolds are underway in our laboratories.

## Data Availability

The data underlying this study are available in the published article and its Supporting Information.

## Supporting Information Available

Synthetic procedures and analytic characterization; Computational details (PDF)
