# Supplementary material for: Nickel Catalyzed Carbonylation/Carboxylation Sequence via Double CO2 Incorporation
Source: Org Lett. 2023 Sep 5;25(38):6969–74. doi: 10.1021/acs.orglett.3c02394 (PMC10546374; doi:10.1021/acs.orglett.3c02394)
Supplement: Supplementary file 1 — ol3c02394_si_001.pdf [file ol3c02394_si_001.pdf]

Supporting Information

Nickel Catalyzed Carbonylation/Carboxylation Sequence via double CO_2 incorporation

Riccardo Giovanelli,[a,b] Lorenzo Lombardi,[a,b] Riccardo Pedrazzani,[a,b] Magda Monari,[a,b] Marta Castiñeira Reis,[c] Carlos Silva López,*[c] Giulio Bertuzzi,*[a,b] Marco Bandini*[a,b]

[a] Dipartimento di Chimica "Giacomo Ciamician", Alma Mater Studiorum – Università di Bologna. Via Selmi 2, 40126, Bologna, Italy

[b] Center for Chemical Catalysis – C3, Alma Mater Studiorum – Università di Bologna. Via Selmi 2, 40126, Bologna, Italy

[C] Departamento de Química Orgánica, Universidad de Vigo. As Lagoas-Marcosende, 36310, Vigo, Spain

Table of Contents

1. General experimental methods S3

  1.1 General crystallographic methods S4

  1.2 General computational details and considerations S5

2. Synthesis of starting materials S7

  2.1 Synthesis of cyclobutan-alkylidenes S7

  2.2 Preparation of Nickel complex [Ni(L1)$_2$Cl$_2$] S11

3. Additional Optimization Tables S14

  3.1 Screening of reaction condition (Table S2) S14

  3.2 Extended screening of catalysts (Table S3) S15

  3.3 Catalysts performances: reaction parameters variations S16

  3.4 List of tested ligands S18

4. Computational mechanistic studies S20

5. Nickel catalyzed carbonylation-carboxylation sequence S25

6. Functionalization and derivatization of products S34

7. ^{1}H-, ^{19}F-, ^{13}C-NMR Spectra S40

8. Cartesians S87

1. General methods

^{1}H NMR, ^{13}C NMR and ^{19}F NMR spectra were recorded on Varian 400-MR (400 MHz) or Brucker (600 MHz). Data are reported as follows: chemical shift, multiplicity (s = singlet, d = doublet, dd= double doublet, t = triplet, td = triple doublet, dt = double triplet, q = quartet, sext = sextet, sept = septet, p = pseudo, b = broad, m = multiplet), coupling constants (Hz). Chemical shifts are reported in ppm from TMS with the solvent resonance as the internal standard.

HRMS spectra were obtained with a G2XS QTof mass spectrometer using either ESI or APCI ionization techniques, as specified case by case.

Melting points were determined with a Büchi Melting Point B-540 apparatus and are not corrected.

Chromatographic purification was done with 240-400 mesh silica gel.

Anhydrous solvents, except for DMF, were supplied by Sigma Aldrich in Sureseal® bottles and used without any further purification. DMF was dried as follows: 3 Å molecular sieves (MS) were activated under microwave irradiation for 10 minutes, then charged in a flask connected to the Schlenk line and heat-gun dried under vacuum three additional times, then allowed to cool to room temperature. Under an Argon flow, anhydrous DMF (Sureseal® bottle) was transferred to the flask with the 3Å MS and was stored. After 72 h the activation procedure was repeated into another flask with new 3Å MS and DMF was transferred from the first flask to the second one under a continuous argon flow. After 72 additional hours we directly used the DMF from the latter flask and stored it under argon after every use.

Room temperature (rt) refers to the ambient temperature of the laboratory, ranging from 20 °C to 26 °C.

Commercially available chemicals and (non-anhydrous) solvents were purchased from Sigma Aldrich, Fluorochem and TCI Chemicals and used without any further purification.

Zn dust refers to a particle size <10 μm and was purchased from Sigma Aldrich, having ≥98% purity.

1.1. General crystallographic methods

The X-ray intensity data were measured on a Bruker Apex III CCD diffractometer. Cell dimensions and the orientation matrix were initially determined from a least-squares refinement on reflections measured in four sets of 20 exposures, collected in three different ω regions, and eventually refined against all data. A full sphere of reciprocal space was scanned by 0.5° ω steps. The software SMART[3] was used for collecting frames of data, indexing reflections and determination of lattice parameters. The collected frames were then processed for integration by the SAINT program,[1] and an empirical absorption correction was applied using SADABS.[2] The structures were solved by direct methods (SIR 2014)[3] and subsequent Fourier syntheses and refined by full-matrix least-squares on F^2 (SHELXTL)[4] using anisotropic thermal parameters for all non-hydrogen atoms. The aromatic, methyl and methylene hydrogen atoms were placed in calculated positions, refined with isotropic thermal parameters *U(H)* = 1.2 *Ueq*(C) and allowed to ride on their carrier carbons.

Crystal data and details of the data collection for compounds [Ni(**L1**)$_2$Cl$_2$] and **2a** are reported in **Table S1** and **S4**. Molecular drawings were generated using Mercury.[5]

Crystallographic data have been deposited with the Cambridge Crystallographic Data Centre (CCDC) with deposition number CCDC 2267379-2267380 Copies of the data can be obtained free of charge via www.ccdc.cam.ac.uk/getstructures.

[1] *SMART & SAINT Software Reference Manuals*, version 5.051 (Windows NT Version), Bruker Analytical X-ray Instruments Inc.: Madison, Wi, 1998.
[2] Sheldrick, G. M.; *SADABS-2008/1 - Bruker AXS Area Detector Scaling and Absorption Correction*, Bruker AXS: Madison, Wisconsin, USA, 2008.
[3] M.C. Burla, R. Caliandro, B. Carrozzini, G.L. Cascarano, C. Cuocci, C. Giacovazzo, M. Mallamo, A. Mazzone, G. Polidori, "Crystal structure determination and refinement via SIR2014" *J. Appl. Cryst.* **2015**, 48, 306.
[4] G.M. Sheldrick, *Acta Cryst C71*, **2015**, 3.
[5] C.F. Macrae, I. Sovago, S.J. Cottrell, P.T.A. Galek, P. McCabe, E. Pidcock, M. Platings, G.P. Shields, J.S. Stevens, M. Towler P.A. Wood, "*Mercury 4.0*: from visualization to analysis, design and prediction", *J. Appl. Cryst.*, **2020**, *53*, 226.

1.2. General computational details and considerations

We have used the Density Functional Theory (DFT) in the Kohn-Sham formulation to optimize all the stationary points presented in this manuscript. Geometries of all the stationary points were fully optimized at the M06[6]/def2svpp[7] computational level. The effect of solvent (n,n-dimethylformaamide, abbreviated as DMF) was modeled using the polarizable continuum model (PCM)[8] with the default parameters implemented in the Gaussian 09 package.[9] Explicit solvation was also included in some instances since the solvent has the potential ability to coordinate the metallic center.

All geometry optimizations have been optimized using tight convergence criteria in the SCF and requesting a pruned (99.590) grid to guarantee the accuracy of the reported results. Moreover, the calculations were performed considering 1.0 atm and 298 K to simulate the reaction conditions.

Frequency analysis was used to establish the nature of all optimized structures as either minima or transition structures. For all stationary points, the stability of the wave function was also examined.[10] When different spin-states are possible for a stationary point those states were explored by running single point energy calculations starting from the optimized structure with the expected multiplicity. Electronic energies for the optimized structures of the different possible multiplicities are specified in the Cartesian section.

IRC calculations[11] were conducted for important transition states to ensure their connectivity with the expected reactants and products. When the substrates showed conformational freedom,

---

[6] a) Y. Zhao, D. G. Truhlar, The M06 suite of density functionals for main group thermochemistry, thermochemical kinetics, noncovalent interactions, excited states, and transition elements: two new functionals and systematic testing of four M06-class functionals and 12 other functionals. *Theor. Chem. Acc.* **2008**, *120*, 215. b) Y. Zhao, D. G. Truhlar. Density Functionals with Broad Applicability in Chemistry. Acc. Chem. Res. **2008**, *41*, 157.

[7] a) F. Weigend, Accurate Coulomb-fitting basis sets for H to Rn. *Phys. Chem. Chem. Phys.* **2006**, *8*, 1057. b) F. Weigend, R. Ahlrichs, Balanced basis sets of split valences, triple zeta valence and quadruple zeta valence quality for H to Rn: Design and assessment of accuracy. *Phys. Chem. Chem. Phys.* **2005**, *7*, 3297.

[8] J. Tomasi, B. Mennucci, R. Cammi, Quantum Mechanical Continuum Solvation Models. *Chem. Rev.* **2005**, 105, 2999.

[9] Gaussian 09, Revision A.02, M. J. Frisch, G. W. Trucks, H. B. Schlegel, G. E. Scuseria, M. A. Robb, J. R. Cheeseman, G. Scalmani, V. Barone, G. A. Petersson, H. Nakatsuji, X. Li, M. Caricato, A. Marenich, J. Bloino, B. G. Janesko, R. Gomperts, B. Mennucci, H. P. Hratchian, J. V. Ortiz, A. F. Izmaylov, J. L. Sonnenberg, D. Williams-Young, F. Ding, F. Lipparini, F. Egidi, J. Goings, B. Peng, A. Petrone, T. Henderson, D. Ranasinghe, V. G. Zakrzewski, J. Gao, N. Rega, G. Zheng, W. Liang, M. Hada, M. Ehara, K. Toyota, R. Fukuda, J. Hasegawa, M. Ishida, T. Nakajima, Y. Honda, O. Kitao, H. Nakai, T. Vreven, K. Throssell, J. A. Montgomery, J. E. Peralta, F. Ogliaro, M. Bearpark, J. J. Heyd, E. Brothers, K. N. Kudin, V. N. Staroverov, T. Keith, R. Kobayashi, J. Normand, K. Raghavachari, A. Rendell, J. C. Burant, S. S. Iyengar, J. Tomasi, M. Cossi, J. M. Millam, M. Klene, C. Adamo, R. Cammi, J. W. Ochterski, R. L. Martin, K. Morokuma, O. Farkas, J. B. Foresman, D. J. Fox, Gaussian, Inc., Wallingford CT.

[10] a) H. B. Schlegel, J. J. W. McDouall, "Do You Have SCF Stability and Convergence Problems?" in Computational Advances in Organic Chemistry: Molecular Structure and Reactivity, Dordrecht: Springer Netherlands, **1991**, pp. 167–185. b) R. Bauernschmitt, R. Ahlrichs, Stability analysis for solutions of the closed shell Kohn– Sham equation. *J. Chem. Phys.* **1996**, 104, 9047. c) R. Seeger, J. A. Pople, Self-consistent molecular orbital methods. XVIII. Constraints and stability in Hartree–Fock theory. *J. Chem. Phys.* **1977**, *66*, 3045.

[11] a) S. Maeda, Y. Harabuchi, Y. Ono, T. Taketsugu, K. Morokuma, Intrinsic reaction coordinate: Calculation, bifurcation, and automated search. Inter. *J. Quantum Chem*. **2015**, *115*, 258. b) K. Fukui, The path of chemical reactions - the IRC approach. *Acc. Chem. Res.* **1981**, *14*, 363.

conformational analysis was performed manually, it must be indicated that only the most stable conformer of each stationary point was considered and reported unless otherwise indicated. The visualization of the reported structures was performed using MOLDEN.[12] The representation of the structures here presented were generated using CYLView.[13] The reduction steps constitute a troublesome point in this research since they involve metallic Zn. We have worked here under the consideration that in the presence of such a coordinating solvent such as DMF part of the metallic Zn will be efficiently solvated and leached in the form of $Zn(DMF)_3$, in agreement with our previous results.

Topological analysis of the electron density was done via analysis of the wavefunction using Multiwfn.[14]

[12] a) S. Maeda, Y. Harabuchi, Y. Ono, T. Taketsugu, K. Morokuma, Intrinsic reaction coordinate: Calculation, bifurcation, and automated search. Inter. *J. Quantum Chem*. **2015**, *115*, 258. b) K. Fukui, The path of chemical reactions - the IRC approach. *Acc. Chem. Res.* **1981**, *14*, 363.
[13] CYLview20; C. Y. Legault, Université de Sherbrooke, 2020 (http://www.cylview.org).
[14] T. Lu, F. Chen, Multiwfn: A multifunctional wavefunction analyzer. *J. Comput. Chem*., **2012**, 33, 580.

2. Synthesis of starting materials

2.1 Synthesis of cyclobutan-alkylidenes

2.1.1 Substrate preparation from corresponding cyclobutanones

Substrates **1** were prepared from the corresponding cyclobutanones **1S** by a Wittig olefination. Cyclobutanones **1S** are known compounds and were prepared following unmodified literature procedures.[15]

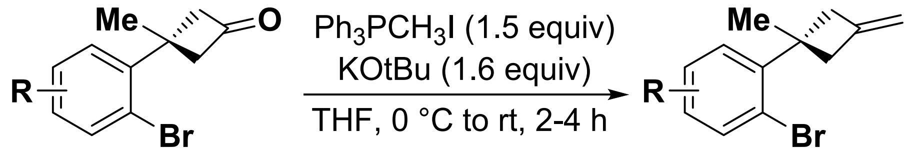

1S **1**

In a heat-gun dried Schlenk flask, under a N_2 atmosphere and magnetic stirring, Ph_3PCH_3I (1.5 equiv, 1.5 mmol, 606 mg) and KOtBu (1.6 equiv, 1.6 mmol, 180 mg) were charged, followed by dry THF (5 mL). The resulting yellow suspension was stirred for 1 h at room temperature and then cooled to 0°C. The desired cyclobutanone **S** (1 mmol, 1 M solution in THF), was added dropwise under vigorous stirring. After the addition, the reaction was stirred at room temperature and monitored by TLC (100% hexane). When full consumption of **S** was observed (ca. 2-4 h), saturated $NH_4Cl_{(aq)}$ (20 mL) was added to the reaction flask and the mixture, transferred to a separatory funnel, was extracted with EtOAc (3x20 mL). The organic phase was dried over Na_2SO_4 and the solvent evaporated *in vacuo*. The reaction crude was finally purified by flash chromatography on silica gel (FC, 100% *n*-hex).

2.1.2 Characterization of substrates 1

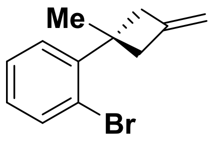

1a. Colorless oil. FC eluent: *n*Hex 100%. Yield = 83% (0.83 mmol, 245.4 mg). **^{1}H NMR** (401 MHz, $CDCl_3$) δ 7.53 (d, *J* = 9.1 Hz, 1H), 7.33 – 7.24 (m, 1H), 7.22 – 7.16 (m, 1H), 7.10 – 7.03 (m, 1H), 4.90 (s, 2H), 3.20 – 3.16 (m, 2H), 2.89 – 2.85 (m, 2H), 1.54 (s, 3H). **^{13}C NMR** (101 MHz, $CDCl_3$) δ 148.2, 144.7, 133.9, 127.9, 127.4, 127.3, 121.7, 106.4, 45.2 (s. 2C), 41.1, 26.8. **HRMS (*ESI*)** m/z: $[M+H]^+$ calcd. for $C_{12}H_{14}Br$ 237.0273; found 237.0267.

[15] L. Lombardi, A. Cerveri, L. Ceccon, R. Pedrazzani, M. Monari, G. Bertuzzi, M. Bandini, *Chem. Commun.*, **2022**, *58*, 4071.

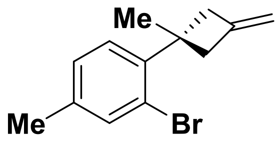

1b. Colorless oil. FC eluent: *n*Hex 100%. Yield = 81% (0.81 mmol, 242.2 mg). **^{1}H NMR** (401 MHz, $CDCl_3$) δ 7.37 (s, 1H), 7.12 – 7.02 (m, 2H), 4.90 (s, 2H), 3.21 – 3.09 (m, 2H), 2.90 – 2.79 (m, 2H), 2.32 (s, 3H), 1.52 (s, 3H). **^{13}C NMR** (101 MHz, $CDCl_3$) δ 145.1, 144.8, 137.3, 134.2, 128.0, 127.6, 121.5, 106.4, 45.2 (2C), 40.6, 26.9, 20.4. **HRMS (*ESI)*** m/z: $[M+H]^+$ calcd. for $C_{13}H_{16}Br$ 251.0430; found 251.0439.

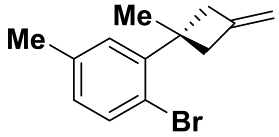

1c. Colorless oil. FC eluent: *n*Hex 100%. Yield = 83% (0.83 mmol, 248.5 mg). **^{1}H NMR** (401 MHz, $CDCl_3$) δ 7.40 (d, *J* = 8.0 Hz, 1H), 6.99 (s, 1H), 6.88 (d, *J* = 8.0 Hz, 1H), 4.90 (s, 2H), 3.24 – 3.09 (m, 2H), 2.93 – 2.78 (m, 2H), 2.32 (s, 3H), 1.53 (s, 3H). **^{13}C NMR** (101 MHz, $CDCl_3$) δ 147.9, 144.8, 137.0, 133.6, 128.7, 128.3, 118.3, 106.3, 45.2 (2C), 40.9, 26.8, 21.0. **HRMS (*ESI)*** m/z: $[M+H]^+$ calcd. for $C_{13}H_{16}Br$ 251.0430; found 251.0435.

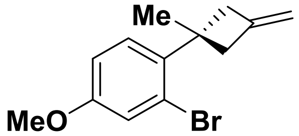

1d. Colorless oil. FC eluent: *n*Hex 100%. Yield = 86% (0.86 mmol, 273.2 mg). **^{1}H NMR** (401 MHz, $CDCl_3$) δ 7.12 – 7.06 (m, 2H), 6.83 (dd, *J* = 8.6, 2.6 Hz, 1H), 4.89 (s, 2H), 3.79 (s, 3H), 3.20 – 3.07 (m, 2H), 2.89 – 2.77 (m, 2H), 1.50 (s, 3H). **^{13}C NMR** (101 MHz, $CDCl_3$) δ 158.1, 144.9, 140.3, 128.2, 121.8, 118.8, 113.3, 106.3, 55.5, 45.3 (2C), 40.3, 27.0. **HRMS (*ESI)*** m/z: $[M+H]^+$ calcd. for $C_{13}H_{16}OBr$ 267.0379; found 267.0367.

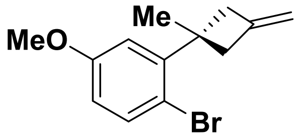

1e Colorless oil. FC eluent: *n*Hex 100%. Yield = 80% (0.83 mmol, 264.0 mg). **^{1}H NMR** (401 MHz, $CDCl_3$) δ 7.42 (d, *J* = 8.7 Hz, 1H), 6.75 (d, *J* = 3.0 Hz, 1H), 6.63 (dd, *J* = 8.7, 3.0 Hz, 1H), 4.92 (s, 2H), 3.81 (s, 3H), 3.24 – 3.09 (m, 2H), 2.93 – 2.76 (m, 2H), 1.54 (s, 3H). **^{13}C NMR** (101 MHz, $CDCl_3$) δ 158.9, 149.4, 144.4, 134.5, 114.3, 112.4, 112.2, 106.5, 55.4, 45.1 (2C), 41.1, 26.7. **HRMS (*ESI)*** m/z: $[M+H]^+$ calcd. for $C_{13}H_{16}OBr$ 267.0379; found 267.0381.

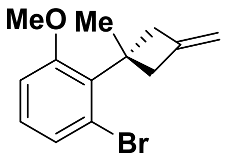

1f. Colorless oil. FC eluent: *n*Hex 100%. Yield = 89% (0.89 mmol, 283.7 mg). **^{1}H NMR** (401 MHz, $CDCl_3$) δ ^{1}H NMR (401 MHz, cdcl$_3$) δ 7.18 (dd, *J* = 8.0, 1.1 Hz, 1H), 7.02 (t, *J* = 8.1 Hz, 1H), 6.83 (dd, *J* = 8.2, 0.9 Hz, 1H), 4.86 (s, 2H), 3.81 (s, 3H), 3.30 – 3.13 (m, 2H), 3.01 – 2.82 (m, 2H), 1.52 (s, 3H). **^{13}C NMR** (101 MHz, $CDCl_3$) δ 158.8, 147.2, 136.3, 127.4, 126.2, 122.5, 110.2, 104.8, 55.4, 47.1 (2C), 40.2, 24.2. **HRMS (*ESI)*** m/z: $[M+H]^+$ calcd. for $C_{13}H_{16}OBr$ 267.0379; found 267.0368.

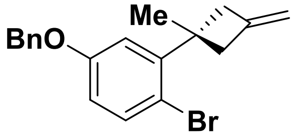

1g. Colorless oil. FC eluent: *n*Hex 100%. Yield = 77% (0.77 mmol, 258.9 mg). **^{1}H NMR** (401 MHz, $CDCl_3$) δ 7.48 – 7.39 (m, 5H), 7.38 – 7.33 (m, 1H), 6.82 (d, *J* = 3.0 Hz, 1H), 6.70 (dd, *J* = 8.7, 3.0 Hz, 1H), 5.05 (s, 2H), 4.90

(s, 2H), 3.20 – 3.09 (m, 2H), 2.89 – 2.80 (m, 2H), 1.52 (s, 3H). **^{13}C NMR** (101 MHz, $CDCl_3$) δ 158.0, 149.4, 144.5, 136.6, 134.4, 128.6 (2C), 128.1, 127.5 (2C), 115.3, 113.3, 112.4, 106.4, 70.2, 45.1 (2C), 41.1, 26.7. **HRMS (*ESI*)** m/z: $[M+H]^+$ calcd. for $C_{19}H_{20}OBr$ 343.0692; found 343.0697**.**

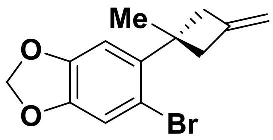

1h. Colorless oil. FC eluent: *n*Hex 100%. Yield = 67% (0.67 mmol, 188.1 mg). **^{1}H NMR** (401 MHz, $CDCl_3$) δ 6.97 (s, 1H), 6.66 (s, 1H), 5.96 (s, 2H), 4.88 (s, 2H), 3.15 – 3.03 (m, 2H), 2.85 – 2.74 (m, 2H), 1.48 (s, 3H). **^{13}C NMR** (101 MHz, $CDCl_3$) δ 147.2, 146.4, 144.5, 141.7, 113.6, 111.7, 107.7, 106.4, 101.6, 45.4 (2C), 41.0, 26.7 **HRMS (*ESI*)** m/z: $[M+H]^+$ calcd. for $C_{14}H_{16}OBr$ 279.0379; found 279.0371.

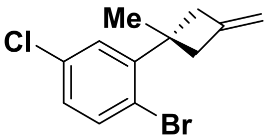

1i. Colorless oil. FC eluent: *n*Hex 100%. Yield = 74% (0.74 mmol, 261.3 mg). **^{1}H NMR** (401 MHz, $CDCl_3$) δ 7.44 (d, *J* = 8.4 Hz, 1H), 7.15 (d, *J* = 2.5 Hz, 1H), 7.04 (dd, *J* = 8.4, 2.6 Hz, 1H), 4.92 (s, 2H), 3.19 – 3.10 (m, 2H), 2.90 – 2.82 (m, 2H), 1.53 (s, 3H). **^{13}C NMR** (101 MHz, $CDCl_3$) δ 150.0, 143.8, 134.9, 133.3, 128.1, 127.5, 119.5, 106.9, 45.0 (2C), 41.0, 26.5. **HRMS (*ESI*)** m/z: $[M+H]^+$ calcd. for $C_{12}H_{13}BrCl$ 270.9884; found 270.9891.

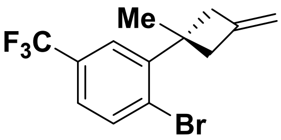

1j. Colorless oil. FC eluent: *n*Hex 100%. Yield = 69% (0.69 mmol, 210.4 mg). **^{1}H NMR** (401 MHz, $CDCl_3$) δ 7.65 (d, *J* = 8.3 Hz, 1H), 7.40 (d, *J* = 1.7 Hz, 1H), 7.31 (dd, *J* = 8.3, 1.9 Hz, 1H), 4.92 (s, 2H), 3.21 – 3.13 (m, 2H), 2.94 – 2.86 (m, 2H), 1.55 (s, 3H). **^{13}C NMR** (101 MHz, $CDCl_3$) δ 149.3, 143.6, 134.5, 129.8 (q, *J* = 32.7 Hz), 125.6 (q, *J* = 1.5 Hz), 124.8 (q, *J* = 3.8 Hz), 124.2 (q, *J* = 3.7 Hz), 124.0 (q, *J* = 273.2 Hz) 107.0, 45.1 (2C), 41.2, 26.5. **^{19}F NMR** (377 MHz, $CDCl_3$) δ -62.6 (s, 3F). **HRMS (*ESI*)** m/z: $[M+H]^+$ calcd. for $C_{13}H_{13}BrF_3$ 305.0147; found 305.0140.

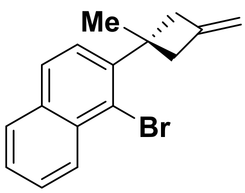

1k. Colorless oil. FC eluent: *n*Hex 100%. Yield = 79% (0.79 mmol, 226.6 mg). **^{1}H NMR** (401 MHz, $CDCl_3$) δ 8.41 (d, *J* = 8.6 Hz, 1H), 7.82 – 7.78 (m, 2H), 7.60 (t, *J* = 7.7 Hz, 1H), 7.51 (t, *J* = 7.5 Hz, 1H), 7.31 (d, *J* = 8.5 Hz, 1H), 4.95 (s, 2H), 3.36 – 3.26 (m, 2H), 3.06 – 2.93 (m, 2H), 1.63 (s, 3H). **^{13}C NMR** (101 MHz, $CDCl_3$) δ 146.2, 144.6, 133.0, 132.8, 127.9, 127.7, 127.4, 126.9, 126.0, 125.6, 121.3, 106.4, 45.8 (2C), 42.2, 26.5. **HRMS (*ESI*)** m/z: $[M+H]^+$ calcd. for $C_{16}H_{16}Br$ 287.0430; found 287.0438.

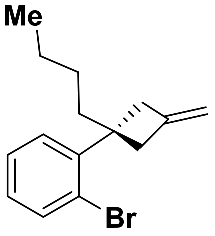

1l. Colorless oil. FC eluent: *n*Hex 100%. Yield = 90% (0.90 mmol, 251.4 mg). **^{1}H NMR** (401 MHz, $CDCl_3$) δ 7.52 (d, *J* = 7.9 Hz, 1H), 7.29 – 7.22 (m, 1H), 7.10 (d, *J*

= 7.8 Hz, 1H), 7.05 (td, J = 7.6, 1.7 Hz, 1H), 4.86 (s, 2H), 3.17 – 3.07 (m, 2H), 3.00 – 2.82 (m, 2H), 1.94 (bs, 2H), 1.30 – 1.14 (m, 2H), 0.97 (bs, 2H), 0.82 (t, J = 7.3 Hz, 3H). **^{13}C NMR** (101 MHz, $CDCl_3$) δ 146.1, 145.3, 133.9, 129.6, 127.4, 126.6, 121.9, 106.1, 44.6, 43.8 (b, 2C), 37.4, 27.4, 23.0, 14.0. **HRMS (*ESI)*** m/z: $[M+H]^+$ calcd. for $C_{15}H_{20}Br$ 279.0743; found 279.0759.

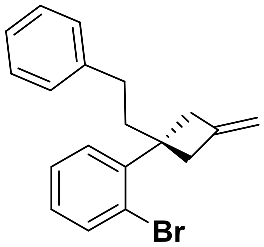

1m. Colorless oil. FC eluent: *n*Hex 100%. Yield = 75% (0.75 mmol, 244.5 mg). **^{1}H NMR** (401 MHz, $CDCl_3$) δ 7.58 (dd, J = 7.9, 1.3 Hz, 1H), 7.34 – 7.28 (m, 1H), 7.28 – 7.22 (m, 2H), 7.22 – 7.14 (m, 2H), 7.14 – 7.07 (m, 3H), 4.91 (s, 2H), 3.27 – 3.13 (m, 2H), 3.07 – 2.94 (m, 2H), 2.32 (bs, 4H). **^{13}C NMR** (101 MHz, $CDCl_3$) δ 145.5, 144.7, 142.3, 134.1, 129.6, 128.24 (2C), 128,23 (2C), 127.7, 126.7, 125.6, 121.9, 106.5, 44.7, 43.9 (b, 2C), 39.5, 31.8. **HRMS (*ESI)*** m/z: $[M+H]^+$ calcd. for $C_{19}H_{20}Br$ 327.0743; found 327.0755.

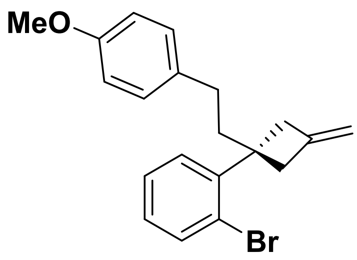

1n. Colorless oil. FC eluent: *n*Hex 100%. Yield = 70% (0.70 mmol, 249.3 mg). **^{1}H NMR** (401 MHz, $CDCl_3$) δ 7.61 – 7.54 (m, 1H), 7.33 – 7.28 (m, 1H), 7.21 – 7.16 (m, 1H), 7.13 – 7.06 (m, 1H), 7.05 – 6.98 (m, 2H), 6.83 – 6.75 (m, 2H), 4.89 (s, 2H), 3.78 (s, 3H), 3.24 – 3.14 (m, 2H), 3.05 – 2.92 (m, 2H), 2.38 – 2.14 (bm, 4H). **^{13}C NMR** (101 MHz, $CDCl_3$) δ 157.6, 145.5, 144.7, 134.3, 134.1, 129.6, 129.1 (2C), 127.6, 126.7, 121.9, 113.7 (2C), 106.4, 55.2, 44.7 (b, 2C), 43.2, 39.7, 30.8. **HRMS (*ESI)*** m/z: $[M+H]^+$ calcd. for $C_{20}H_{22}OBr$ 357.0849; found 357.0851.

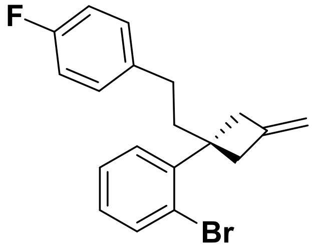

1o. Colorless oil. FC eluent: *n*Hex 100%. Yield = 62% (0.62 mmol, 214.4 mg). **^{1}H NMR** (401 MHz, $CDCl_3$) δ 7.58 (d, J = 7.9 Hz, 1H), 7.31 (t, J = 7.5 Hz, 1H), 7.19 (dd, J = 7.8, 1.6 Hz, 1H), 7.11 (t, J = 7.6, 1.7 Hz, 1H), 7.08 – 7.02 (m, 2H), 6.97 – 6.90 (m, 2H), 4.92 (s, 2H), 3.30 – 3.15 (m, 2H), 3.08 – 2.91 (m, 2H), 2.30 (bm, 4H). **^{13}C NMR** (101 MHz, $CDCl_3$) δ 161.1 (d, J = 243.1 Hz), 145.3, 144.6, 137.8 (d, J = 3.2 Hz), 134.1, 129.6, 129.5 (d, J = 7.7 Hz, 2C), 127.7, 126.8, 121.9, 114.9 (d, J = 21.1 Hz, 2C), 106.6, 44.7, 43.8 (b, 2C), 39.6, 31.0. **^{19}F NMR** (377 MHz, $CDCl_3$) δ -118.0 (m, 1F). **HRMS (*ESI)*** m/z: $[M+H]^+$ calcd. for $C_{19}H_{19}BrF$ 345.0649; found 345.0645.

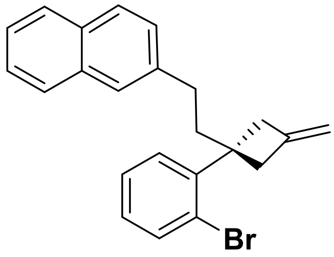

1p. Colorless oil. FC eluent: *n*Hex 100%. Yield = 68% (0.68 mmol, 260.1 mg). **^{1}H NMR** (401 MHz, $CDCl_3$) δ 7.82 – 7.70 (m, 3H), 7.58 (d, J = 7.9 Hz, 1H), 7.53 (s, 1H), 7.47 – 7.37 (m, 2H), 7.32 (td, J = 7.7, 1.3 Hz, 1H),

7.26 – 7.20 (m, 2H), 7.11 (td, *J* = 7.6, 1.7 Hz, 1H), 4.90 (s, 2H), 3.31 – 3.13 (m, 2H), 3.11 – 2.92 (m, 2H), 2.56 – 2.26 (bm, 4H). **^{13}C NMR** (101 MHz, $CDCl_3$) δ 145.5, 144.7, 139.8, 134.1, 133.6, 131.9, 129.7, 127.72, 127.70, 127.5,127.34, 127.32, 126.8, 126.1, 125.8, 125.0, 122.0, 106.5, 44.8, 43.8 (b, 2C), 39.4, 32.0. **HRMS (*ESI)*** m/z: $[M+H]^+$ calcd. for $C_{23}H_{22}Br$ 377.0899; found 377.0905.

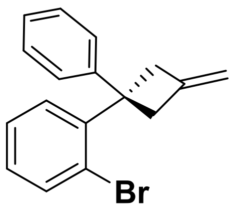

1q. Pale yellow oil. FC eluent: *n*Hex 100%. Yield = 63% (0.63 mmol, 188.8 mg). **^{1}H NMR** (401 MHz, $CDCl_3$) δ 7.56 (d, *J* = 7.9 Hz, 1H), 7.51 (d, *J* = 6.5 Hz, 1H), 7.40 (t, *J* = 8.1 Hz, 1H), 7.34 – 7.24 (m, 4H), 7.23 – 7.11 (m, 2H), 4.95 (s, 2H), 3.62 – 3.52 (m, 2H), 3.50 – 3.38 (m, 2H). **^{13}C NMR** (101 MHz, $CDCl_3$) δ 146.5, 146.4, 143.9, 134.3, 129.3, 128.1 (2C), 128.0, 127.0, 126.1 (2C), 125.8, 123.7, 106.3, 48.3, 46.4 (2C). **HRMS (*ESI)*** m/z: $[M+H]^+$ calcd. for $C_{17}H_{16}Br$ 299.0430; found 299.0425.

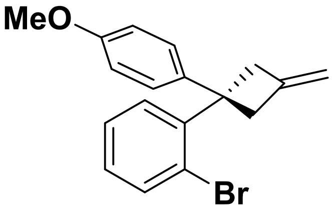

1r.[16] Colorless oil. FC eluent: *n*Hex 100%. Yield = 77% (0.77 mmol, 253.6 mg). **^{1}H NMR** (401 MHz, $CDCl_3$) δ ^{1}H NMR 7.66 (d, *J* = 7.9 Hz, 1H), 7.60 (d, *J* = 7.8 Hz, 1H), 7.47 (t, *J* = 7.5 Hz, 1H), 7.36 (d, *J* = 8.8 Hz, 2H), 7.20 (t, *J* = 7.6 Hz, 1H), 6.95 – 6.92 (m, 2H), 5.08 (s, 2H), 3.85 (s, 3H), 3.71 – 3.61 (m, 2H), 3.59 – 3.45 (m, 2H). **^{13}C NMR** (101 MHz, $CDCl_3$) δ 157.5, 146.6, 143.9, 138.4, 134.1, 129.1, 127.8, 127.1 (2C), 126.9, 123.4, 113.3 (2C), 106.1, 54.9, 47.6, 46.3 (2C). **HRMS (*ESI)*** m/z: $[M+H]^+$ calcd. for $C_{18}H_{18}OBr$ 329.0536; found 329.0541.

2.2 Preparation of Nickel complex [Ni(L1)$_2$Cl$_2$]

A heat-gun dried Schlenk tube equipped with a magnetic stirring bar under a continuous N_2 flow was charged with dry THF (5 mL). $NiCl_2$·glyme (0.2 mmol) was added, followed by **L1** (2 equiv, 0.4 mmol, 86.4 mg); the tube was then sealed, and the mixture was refluxed for 16 h. The resulting suspension was purified by centrifugation (8000 rpm, 3 min) washing with EtOAc (2 x 10 mL). The obtained complex was then transferred to the final vessel and dried under vacuum for several hours. Complex **[Ni(L1)$_2$Cl$_2$]** was thus obtained as a pale green fine powder in quantitative yield (0.2 mmol, 112.2 mg). The mother liquor from all the washings was evaporated and the residues were filtered over a short Celite pad and analyzed by ^{1}H-NMR spectroscopy. If no traces of ligand **L1** were detected, the

[16] Isolated along with about 12% of monochlorinated alkylidene cyclobutane (deriving from the preparation, inseparable mixture). This chlorinated impurity does not affect the catalytic process, nor it is involved in it, therefore the corresponding product **2r** could be easily purified and isolated.

1:2 (Ni:**L1**) stoichiometry of the complex was assumed to be correct. Structural elucidation was successively carried out by single-crystal X-ray diffraction analysis (*vide infra*).

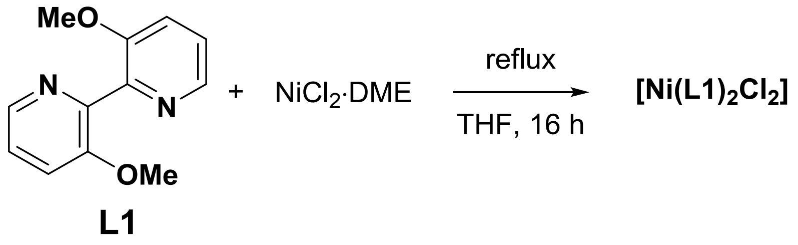

2.2.1 Crystal data and structure refinement for compounds [Ni(L1)$_2$Cl$_2$].

SC-XRD suitable crystals were obtained from layering a saturated solution of **[Ni(L1)2Cl2]** in acetonitrile on toluene in a closed cup vial. Small deep green rhombohedral crystals were observed.

**Compound**	**[Ni(L1)$_2$Cl$_2$]**
Formula	$C_{24}H_{24}Cl_2N_4NiO_4 \cdot CH_3CN$
Fw	603.13
T, K	296(2)
λ, Å	0.71073
Crystal symmetry	Triclinic
Space group	P -1
a, Å	10.1973(5)
b, Å	11.0334(5)
c, Å	13.5797(6)
α	101.161(2)
β	106.863(2)
γ	101.404(2)
Cell volume, Å^3	1381.1(1)
Z	2
D_c, Mg m^{-3}	1.450
μ(Mo-K_α), mm^{-1}	0.937
F(000)	624
Crystal size/ mm	0.12 x 0.08 x 0.06
θ limits, °	1.626 to 27.000
Reflections collected	18647
Unique obs. Reflections [$F_o > 4\sigma(F_o)$]	5891 [R(int) = 0.0442]
Goodness-of-fit-on F^2	0.885

R_1 (F)[a], wR_2 (F^2) [I > 2σ(I)][b]	R1 = 0.0596, wR2 = 0.1255
Largest diff. peak and hole, e. $Å^{-3}$	0.596 and -0.567

Table S1. [a)] $R_1 = \Sigma||F_o|-|F_c||/\Sigma|F_o|$. [b] $wR_2 = [\Sigma w(F_o^2-F_c^2)^2/\Sigma w(F_o^2)^2]^{1/2}$ where $w = 1/[\sigma^2(F_o^2) + (aP)^2 + bP]$ where $P = (F_o^2 + F_c^2)/3$.

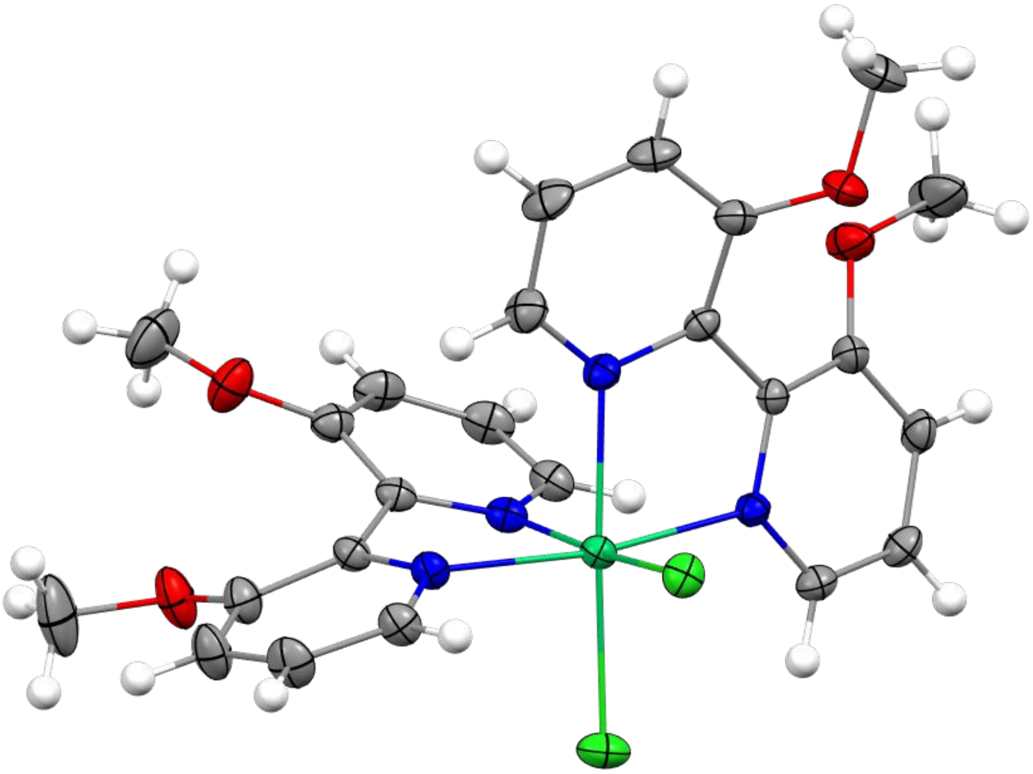

Figure S1. ORTEP drawing of **[Ni(L1)$_2$Cl$_2$]**. Thermal ellipsoid are drawn at 30% of the probability level. Solvated acetonitrile molecule removed for clarity.

3. Additional optimization Tables

3.1 Screening of reaction condition (Table S2).

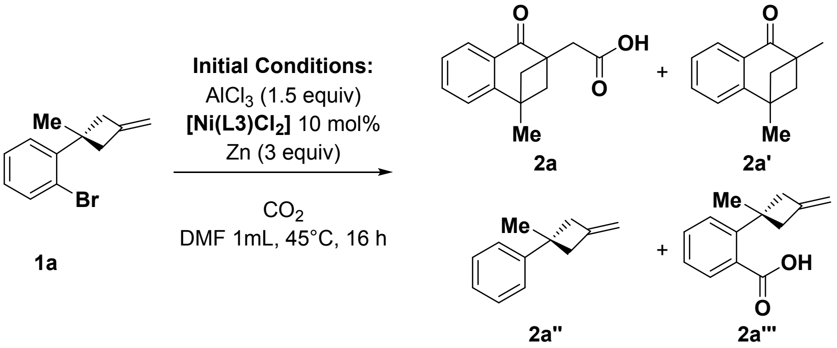

Entry	Deviation from Initial Conditions[a]	1a	2a	2a'	2a''	2a'''
1	--	16	9.5	2.5	68.5	3.5
2[b]	$AlCl_3$ 3.0 equiv	7	11.5	3	76	2.5
3[b]	$AlCl_3$ 4.5 equiv	13	45	5	33	4
4[b]	$AlCl_3$ 6.0 equiv	8.5	25.0	4.5	57	5.0
5	$[Ni(\mathbf{L3})_2Cl_2]$	traces	57	3	29	10
6[c]	Zn 2.0 equiv	14	31	8	44.5	2.5
7[c]	Zn 4.0 equiv	9	28	10	45	8
8	5 mol% $[Ni(\mathbf{L3})_2Cl_2]$	14	21.5	7.5	51.5	5.5
9	15 mol% $[Ni(\mathbf{L3})_2Cl_2]$	traces	47	13	31	8
10	$[Ni(\mathbf{L1})_2Cl_2]$	traces	70	7	21	traces
11[d,e]	rT	traces	53.5	3	38.5	4
12[d]	55°C	6	29	6.5	58	traces
13[d]	6 h	24	10	12.5	52.5	traces
14[d]	48 h	4	49	9	33	5
15[d]	2 mL DMF	4.5	50	10	33.5	2
16[d]	ACN	traces	traces	traces	95	0
17[d]	DMSO	10	traces	traces	88	0
18	$Al(OTf)_3$ instead of $AlCl_3$	78	traces	traces	traces	traces
19	$Al(OTf)_3$ + LiCl instead of $AlCl_3$	73	traces	traces	traces	traces
20	$MgCl_2$ instead of $AlCl_3$	79	traces	traces	traces	traces
21	$ZnCl_2$ instead of $AlCl_3$	18	traces	traces	82	traces
22	TMSCl instead of $AlCl_3$	70	traces	traces	traces	traces

[a] Yields NMR given by standard addition of mesitylene 0.5 equiv. [b] Performed with $[Ni(\mathbf{L3})Cl_2]$. [c] Performed with $[Ni(\mathbf{L3})_2Cl_2]$. [d] Performed with $[Ni(\mathbf{L1})_2Cl_2]$. [e] Rt is the laboratory medium temperature 20 - 26°C

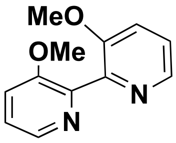

L1

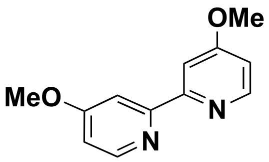

L3

3.2 Extended screening of catalysts (Table S3).

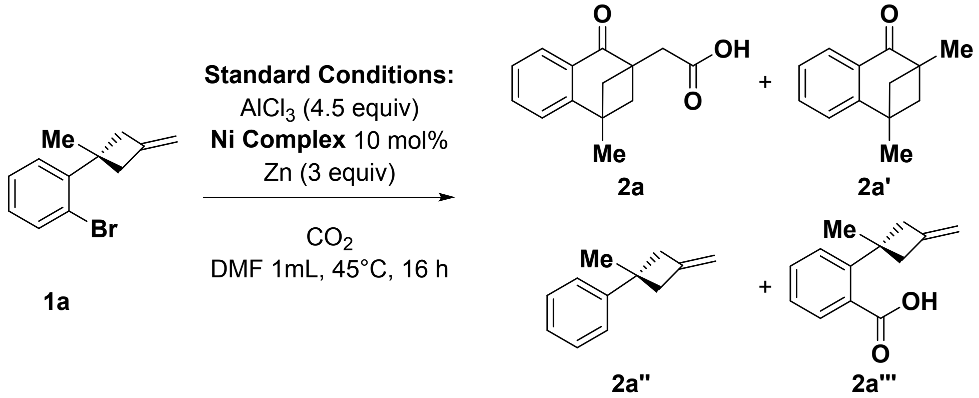

Entry	Nickel Complex[a]	1a (%)	2a (%)	2a' (%)	2a'' (%)	2a''' (%)
1	*Optimal Conditions*	traces	70	7	21	traces
2	[Ni(**L1**)$_2$I$_2$][b]	traces	64	10	25	0
3	[Ni(**L2**)$_2$Cl$_2$][b]	traces	64	7	26.5	2
4	[Ni(**L2**)$_2$I$_2$]	traces	53	10.5	30.5	5
5	[Ni(**L3**)Cl$_2$]	13	45	5	33	4
6	[Ni(**L3**)Br$_2$]	7	32	8	41.5	11.5
7	[Ni(**L3**)I$_2$]	traces	32.5	11	49.5	6
8	[Ni(**L3**)$_2$Cl$_2$][b]	4	61	4	20.5	10.5
9	[Ni(**L3**)$_3$Cl$_2$]	12	8	8	50	22
10	[Ni(**L4**)$_2$Cl$_2$][b]	traces	19	traces	46	33
11	[Ni(**L4**)$_2$I$_2$]	traces	33	2.5	34.5	29
12	[Ni(**L5**)$_2$Cl$_2$][b]	8	15	4.5	65	7.5
13	[Ni(**L6**)Cl$_2$][b]	6	0	0	89	5
14	[Ni(**L7**)Cl$_2$]	8	6	5	61	20
15	[Ni(**L8**)Cl$_2$][c]	22	0	5	68	5
16	[Ni(**L9**)Cl$_2$][c]	15.5	6	20	50	8.5
17	[Ni(**L10**)Cl$_2$]	10	traces	traces	88	0
18	[Ni(**L11**)Cl$_2$]	2.5	traces	traces	95.5	0
19	[Ni(**L12**)$_2$Cl2]	58	0	0	42	0

Table S3. [a] Yields NMR given by standard addition of mesitylene 0.5 equiv. **[b]** Also reported in **Table 1** of the main text. [c]Complex formed *in situ*.

3.3 Catalysts performances: reaction parameters variations

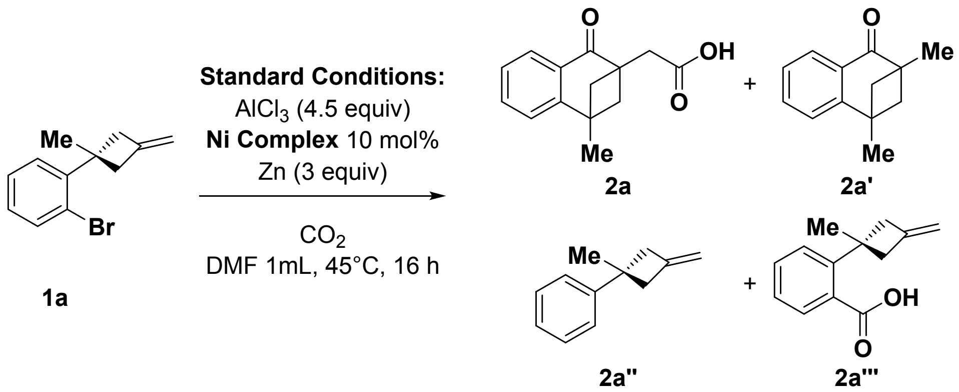

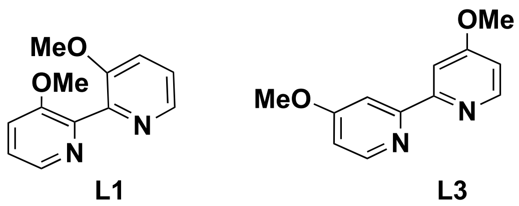

Standard Conditions

10 mol% cat; 4.5 eq $AlCl_3$; 3 eq Zn; 1 mL DMF; 45 °C; 16 h

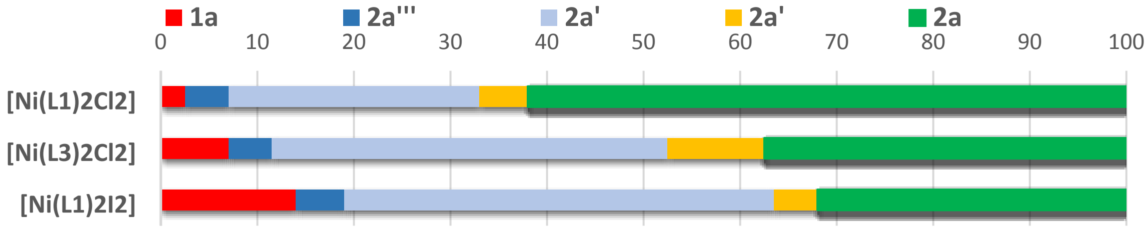

10 mol% cat; 3.5 eq $AlCl_3$; 3 eq Zn; 1 mL DMF; 45 °C; 16 h

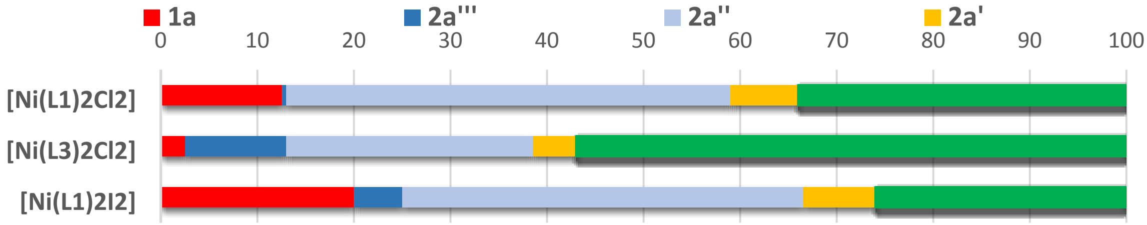

10 mol% cat; 4.5 eq $AlCl_3$; 3 eq Zn; 1 mL DMF; 45 °C; 72 h

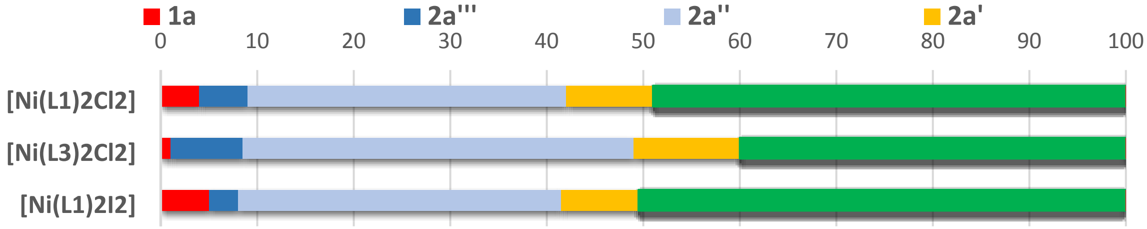

10 mol% cat; 4.5 eq $AlCl_3$; 3 eq Zn; 2 mL DMF; 45 °C; 16 h

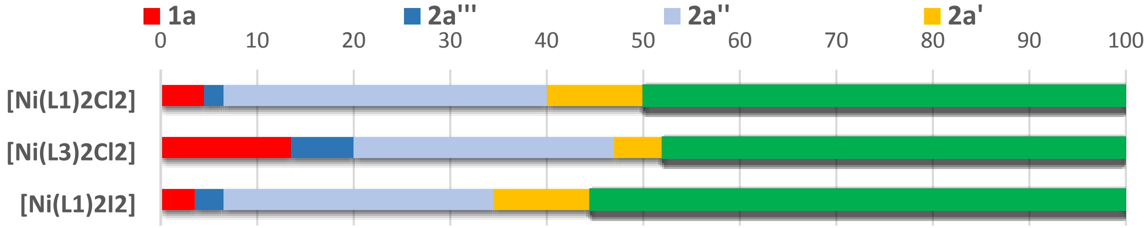

5 mol% cat; 4.5 eq $AlCl_3$; 3 eq Zn; 2 mL DMF; 45 °C; 16 h

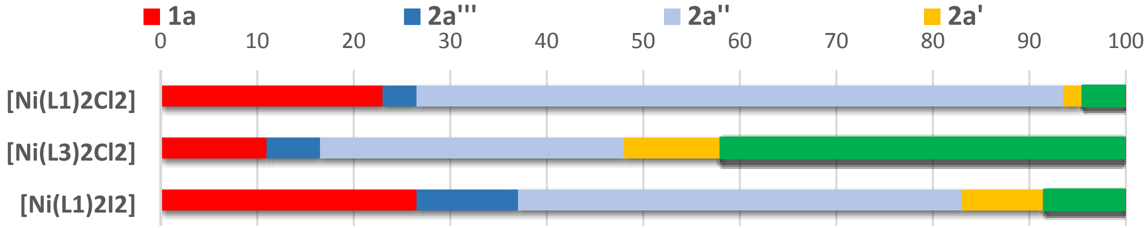

15 mol% cat; 4.5 eq $AlCl_3$; 3 eq Zn; 2 mL DMF; 45 °C; 16 h

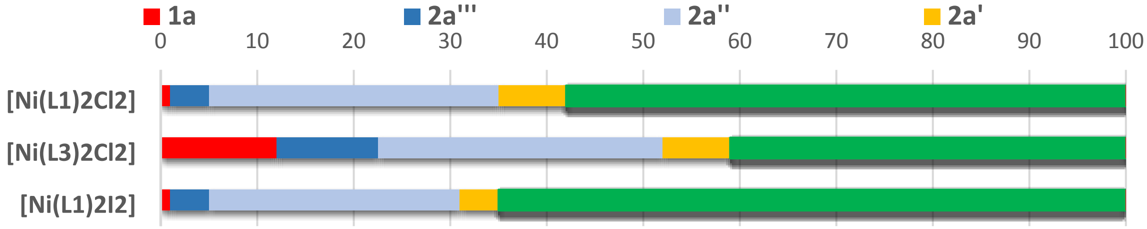

Table S4

3.4 List of tested ligands

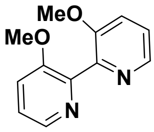

L1

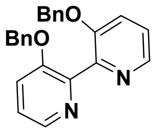

L2

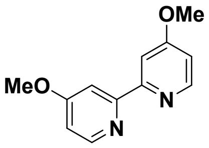

L3

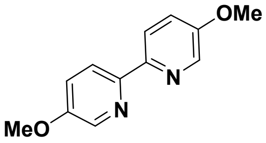

L4

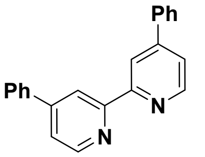

L5

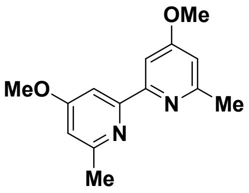

L6

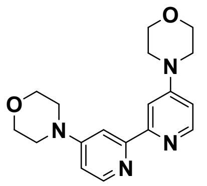

L7

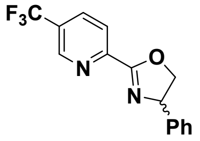

L8

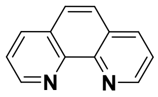

L9

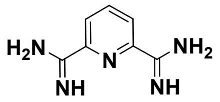

L10

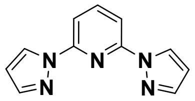

L11

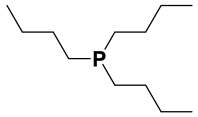

L12

4. Computational mechanistic studies

To gain insights into the reaction mechanism behind the presented transformation we resorted to molecular modelling. We envisioned that the first step of this mechanism would involve the endergonic coordination of **II** to the Ni(I) catalyst (**I**), resulting in **III**. After, the resulting complex can further progress via an oxidative addition towards **IV**. The overall energy penalization of the process is 10.67 kcal/mol. Alternatively, the catalyst in the presence of an excess of Zn(0) could be reduced initially (from Ni(I) to Ni(0)) and then coordinate **II**, we have found that this potential path is energetically less favorable (see **Figure S3**).

Continuing with the initially proposed path, once **IV** is formed, in the presence of an excess of Zn(0), it can evolve through two subsequent highly exergonic reductions forming ultimately **VI**. Then, **VI** can coordinate an $AlCl_3$-activated-CO_2 molecule yielding **VII**. At this stage, we wondered whether it could happen that the coordination of the activated-CO_2, had taken place in a previous step. However, all our attempts to find the coordination of activated-CO_2 to **IV** and **Va** yielded the two independent molecules.

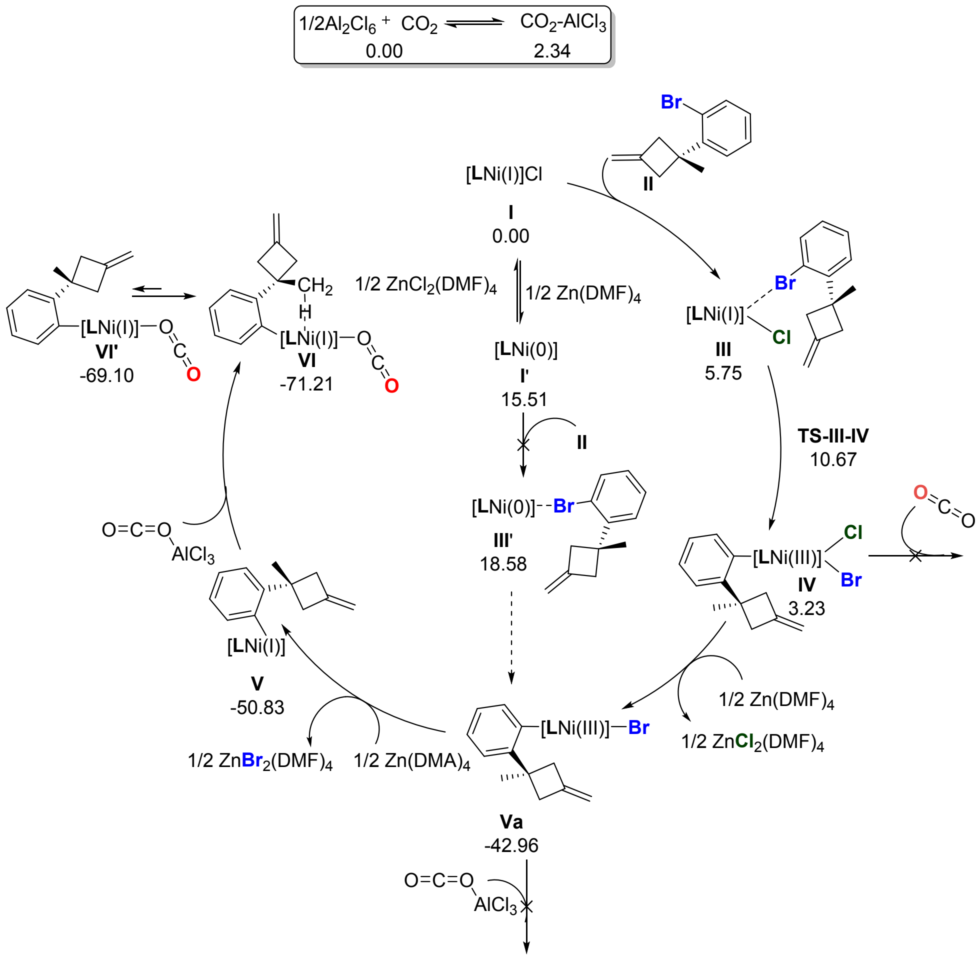

Figure S2. Explored paths for the initial interaction of the catalyst **I** with **II** and $AlCl_3$-activated-CO_2. Energies correspond to relative Gibbs free energies and are expressed in kcal/mol.

Once **VI** is formed it can evolve towards **VII**. Subsequently, the carbonyl group can further coordinate to the metallic center resulting in **VIII** and then coordinate a second $AlCl_3$ molecule rendering **IX**. **IX** can progress via an oxidative insertion on the alkene with the simultaneous addition of the internal carbon of the alkene at the carbonylic carbon rendering **X**. The resulting complex, can release an

$AlCl_3$ unit and the progress via a Zn(0) promoted reduction so that **XII** is formed. **XII** can finally be quenched in the presence of $AlCl_3$ yielding the product (see **Figure S4**).

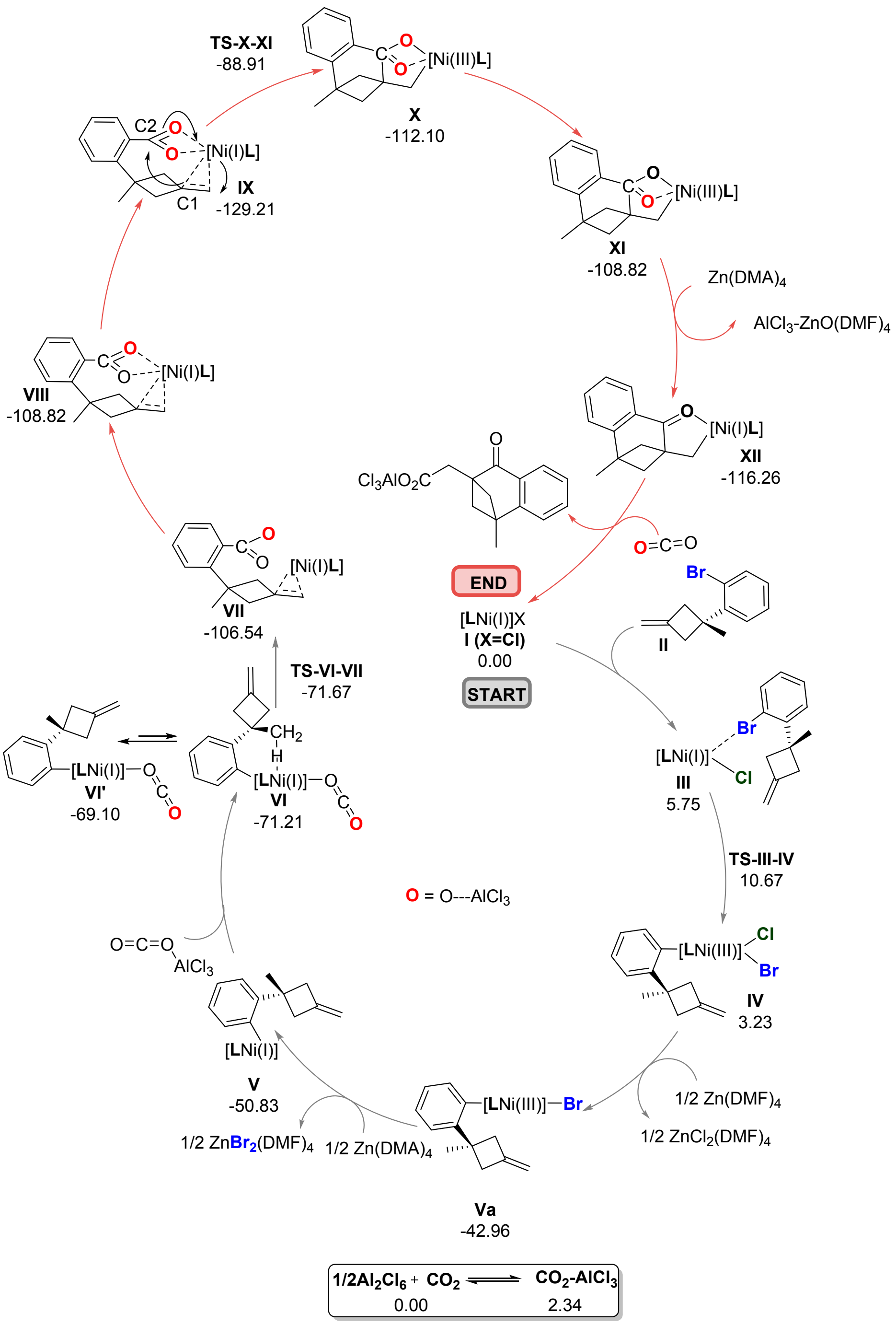

Figure S3. Explored paths for the evolution of **VI** towards the formation of the product and the recovery of the initial catalyst **Ia**. Energies correspond to relative Gibbs free energies and are expressed in kcal/mol.

Analysis of the influence of the methyl group:

We have analyzed structurally (**VI** and **TS-VI-VII**) and using the QTAIM theory the bond critical points at **TS-VI-VII** and we have obtained that for both the minima and transition state there is an interaction among one of the hydrogens of the methyl group and the metal center (Figure S3). This interaction

is manifested through the proximity between the methyl group and the metal center (2.1 Å at **VI** and 2.0 Å at **TS-VI-VII**) but also by the presence of a bond critical point (BCP) between the methyl group and the metal center both at the transition state **TS-VI-VII** (see Figure S3 bottom). Note that at the transition state the Ni-H bond length is shorter than in **VI**, pointing to the fact that this agostic interaction might donate electron density to the metal center ($q_{Ni\ \mathbf{VI}}$ = 1.13 and $q_{Ni\ \mathbf{TS\text{-}VI\text{-}VII}}$ = 0.96)[17] and facilitate the formation of the new C-C bond and the release of the organic fragment.

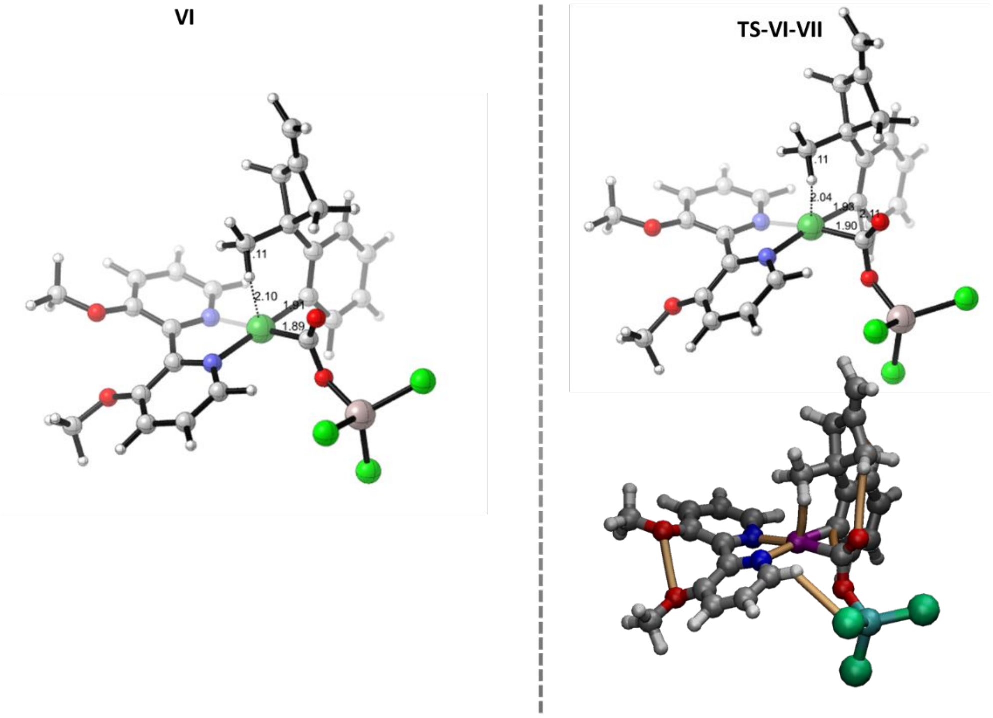

Figure S4. Structural and topological analysis of intermediate **VI** and **TS-VI-VII**. The lines in brown represent bond paths among atoms.

Analysis of the influence of $AlCl_3$:

Having identified the reaction mechanism behind the presented transformation, we wondered up to which extent the presence of $AlCl_3$ was relevant for this Ni(I) catalyzed transformation. We have found that $AlCl_3$ severely stabilizes **VI** and **VI'** as it does with intermediate **IX** (see table S1). Hence, in the absence of this LA the equilibriums of their formation would be shifted towards reactants turning the reaction less efficient.

Table S5. Relative Gibbs Free Energies of intermediates **VI** and I**X** in the presence and absence of $AlCl_3$.

**ID**	**ΔG** (kcal/mol)
**VI**	0.00
**VI-d-a**	47.56
**IX**	0.00
**IX-d-a**	20.92

[17] These charges correspond to APT charges with hydrogens summed into heavy atoms.

**IX-d-b**	59.24

Table S6. Energy report of the explored intermediates and transition states discussed in this manuscript.

ID	ImFreqs	Stable	SCF	SCF+ZPVE	H	G
**I-d**	34.21	Yes	-2691.59765098	-2691.37301300	-2691.35471900	-2691.42037400
**I-q**	40.72	Yes	-2691.56341565	-2691.34044900	-2691.32213400	-2691.38813000
**II**	33.00	Yes	-3038.07095438	-3037.85929400	-3037.84614700	-3037.89860800
**III-d**	19.21	Yes	-5729.68226576	-5729.24438300	-5729.21227500	-5729.30981300
**III-q**	25.16	Yes	-5729.65790751	-5729.22145100	-5729.18965800	-5729.28399700
**TS-III-IV-d**	-139.34	Yes	-5729.67586879	-5729.23895500	-5729.20735500	-5729.30197700
**IV-d**	18.41	Yes	-5729.68889669	-5729.25042300	-5729.21869600	-5729.31383300
**IV-q**	17.39	Yes	-5729.66972042	-5729.23275600	-5729.20016100	-5729.29936700
**Va-s**	17.45	Yes	-5269.63667315	-5269.20025800	-5269.17021500	-5269.26130100
**Va-t**	13.88	Yes	-5269.62113230	-5269.18529100	-5269.15489100	-5269.24871000
**V-d**	11.29	Yes	-2695.80889097	-2695.37517600	-2695.34664000	-2695.43596300
**V-q**	17.67	Yes	-2695.77916957	-2695.34681800	-2695.31836300	-2695.40749900
**VI'-d**	17.13	Yes	-4506.89527489	-4506.44160300	-4506.40300700	-4506.51513700
**VI'-q**	21.26	Yes	-4506.89021875	-4506.43662300	-4506.39789700	-4506.51090800
**VI-d**	17.13	Yes	-4506.89527489	-4506.44160300	-4506.40300700	-4506.51513700
**VI-q**	21.26	Yes	-4506.89021875	-4506.43662300	-4506.39789700	-4506.51090800
**TS-VI-VII-d**	-87.43	Yes	-4506.94576534	-4506.49141100	-4506.45390900	-4506.56438700
**TS-VI-VII-q**	-203.39	Yes	-4506.86288552	-4506.41136900	-4506.37296600	-4506.48578700
**VII-d**	37.72	Yes	-4507.01256756	-4506.55380500	-4506.51738500	-4506.61995300

**VII-q**	18.93	Yes	-4506.94702812	-4506.49071900	-4506.45379000	-4506.55961600
**VIII-d**	7.48	Yes	-4507.01283307	-4506.55370700	-4506.51706600	-4506.62358700
**VIII-q**	13.03	Yes	-4506.94712677	-4506.49119400	-4506.45415000	-4506.56224500
**IX-d**	14.31	Yes	-6129.76846408	-6129.30348800	-6129.25984800	-6129.38247100
**IX-q**	9.36	Yes	-6129.71185873	-6129.24921500	-6129.20561400	-6129.32889100
**TS-IX-X-d**	-212.85	Yes	-6129.70757314	-6129.24459600	-6129.20226900	-6129.31825500
**X-d**	28.38	Yes	-6129.74843724	-6129.28198000	-6129.24016400	-6129.35520700
**X-q**	21.54	Yes	-6129.69100954	-6129.22736200	-6129.18458800	-6129.30392600
**XI-d**	7.48	Yes	-4507.01283307	-4506.55370700	-4506.51706600	-4506.62358700
**XII-d**	9.50	Yes	-2809.04735243	-2808.60019700	-2808.57203700	-2808.65890400
**XII-q**	15.94	Yes	-2809.02900464	-2808.58384900	-2808.55496200	-2808.64447600
**VI-d-a**	10.38	Yes	-2884.14444642	-2883.69711900	-2883.66546800	-2883.76145900
**IX-d-a**	17.42	Yes	-4507.01077605	-4506.55243100	-4506.51557200	-4506.62273200
**IX-d-b**	13.99	Yes	-2884.22615044	-2883.77471600	-2883.74460900	-2883.83527000

5. Nickel catalyzed carbonylation-carboxylation sequence

5.1 General procedure for the nickel catalyzed carbonylation-carboxylation sequence

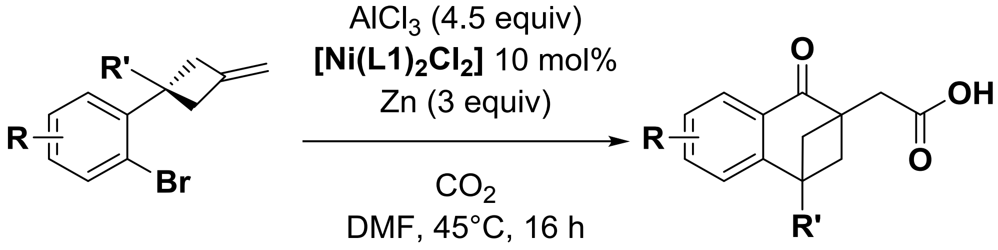

1 **2**

A heat-gun dried Schlenk tube under a constant CO_2 flow was charged with anhydrous DMF (1 mL). Then, under vigorous stirring (1000 rpm, two magnetic stirring bars) $AlCl_3$ (4.5 equiv, 0.45 mmol, 60.0 mg) was added and the Schlenk tube was warmed to 60°C for one minute, in order to solubilize all the solid. The Schlenk tube was then allowed to cool to room temperature and three vacuum/CO_2 cycles were performed. Under a constant flow of CO_2, nickel catalyst [Ni(**L1**)$_2$Cl$_2$] was added (10 mol%, 0.01 mmol, 5.6 mg), followed by the desired starting material **1** (0.1 mmol) and zinc powder (3 equiv, 0.30 mmol, 19.8 mg). A pasteur pipette connected to the Schlenk line was used to bubble CO_2 in the reaction mixture for ca. 10 seconds and then, the tube was screw capped and warmed at 45°C.

After 16 hours the reaction mixture was quenched with HCl (2 M, 5 mL) and extracted with EtOAc (3 x 5 mL), the organic phase was washed with HCl (0.2 M, 3 x 15 mL), dried over Na_2SO_4 and evaporated *in vacuo*. The crude mixture was purified by flash chromatography on silica gel (FC, *n*-hexane/EtOAc + 1% HCOOH mixtures).

5.2 Characterization data of products 2

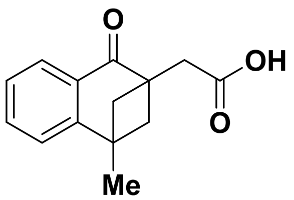

2a. White crystalline solid. FC eluent: *n*Hex/EtOAc 8:2 + 1% HCOOH. Yield = 70% (0.070 mmol, 16.0 mg). **^{1}H NMR** (400 MHz, $CDCl_3$) δ 8.01 (dd, *J* = 7.6, 1.1 Hz, 1H), 7.50 (td, *J* = 7.5, 1.4 Hz, 1H), 7.36 (td, *J* = 7.5, 1.1 Hz, 1H), 7.27 - 7.25 (m, 1H, partially overlapped with the residual solvent signal), 2.79 (s, 2H), 2.72 – 2.62 (m, 2H), 2.55 – 2.47 (m, 2H), 1.56 (s, 3H); the COOH proton signal was not detected. **^{13}C NMR** (101 MHz, $CDCl_3$) δ 201.3, 175.6, 153.5, 133.3, 128.3, 127.0, 126.8, 121.9, 53.5 (2C), 49.1, 39.5, 38.3, 22.1. **HRMS (*ESI)*** m/z: $[M-H]^-$ calcd. for $C_{14}H_{13}O_3$ 229.0870; found 229.0875.

Preparation of 2a on 1 mmol scale. A heat-gun dried Schlenk tube under a constant CO_2 flow was charged with anhydrous DMF (6 mL). Then, under vigorous stirring (1200 rpm, two magnetic stirring bars) $AlCl_3$ (4.5 equiv, 4.5 mmol, 600 mg) was added and the Schlenk tube was warmed to 60°C for 5 minutes, in order to solubilize all the solid. The Schlenk tube was then allowed to cool to room temperature and three vacuum/CO_2 cycles were performed. Under a constant flow of CO_2, nickel catalyst [Ni(**L1**)$_2$Cl$_2$] was added (10 mol%, 0.10 mmol, 56.0 mg), followed by starting material **1a** (1.0 mmol, 237 mg) and zinc powder (3 equiv, 3.0 mmol, 200 mg). A pasteur pipette connected to the Schlenk line was used to bubble CO_2 in the reaction mixture for ca. 40 seconds and then, the tube was screw capped and warmed at 45°C.

After 16 hours the reaction mixture was quenched with HCl (2 M, 30 mL) and extracted with EtOAc (3 x 25 mL), the organic phase was washed with HCl (0.2 M, 3 x 15 mL), dried over Na_2SO_4 and evaporated *in vacuo*. The crude mixture was purified by flash chromatography on silica gel (FC, *n*-hexane/EtOAc + 1% HCOOH mixtures) to afford **2a** in 60% yield (0.60 mmol, 138 mg).

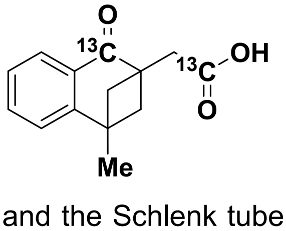

(^{13}C)$_2$-2a. Obtained following a modification of the general procedure, as follows. A heat-gun dried Schlenk tube ***under a constant N_2 flow*** was charged with anhydrous DMF (1mL). Then, under vigorous stirring (1000 rpm, two magnetic stirring bars) $AlCl_3$ (4.5 equiv, 0.45 mmol, 60.0 mg) was added and the Schlenk tube was warmed to 60°C for one minute, in order to solubilize all the solid. The Schlenk tube was then allowed to cool to room temperature and ***three vacuum/N_2 cycles*** were performed. ***Under a constant flow of N_2***, nickel catalyst [Ni(**L1**)$_2$Cl$_2$] was added (10 mol%, 0.01 mmol, 5.6 mg), followed by the desired starting material **1** (0.1 mmol) and zinc powder (3 equiv, 0.30 mmol, 19.8 mg). Then, a mild vacuum was created in the Schlenk tube that was promptly sealed. The vacuum line was switched with a line directly connected to a low-pressure (2.54 atm) flask of $^{13}CO_2$ and opened to let the gas flow in. The operation was repeated once more, then, the tube was screw capped and warmed at 45°C.

After 16 hours the reaction mixture was quenched with HCl (2 M, 5 mL) and extracted with EtOAc (3 x 5 mL), the organic phase was washed with HCl (0.2 M, 3x15 mL), dried over Na_2SO_4 and evaporated *in vacuo*. The crude mixture was purified by flash chromatography on silica gel (FC, *n*-hexane/EtOAc + 1% HCOOH mixtures).

White crystalline solid. FC eluent: *n*Hex/EtOAc 8:2 + 1% HCOOH. Yield = 56% (0.056 mmol, 13.3 mg). ^{1}**H NMR** (600 MHz, $CDCl_3$) δ 7.93 (ddd, *J* = 7.6, 3.8, 1.4 Hz, 1H), 7.41 (td, *J* = 7.6, 1.5 Hz, 1H), 7.27 (t, *J* = 7.5 Hz, 1H), 7.17 (d, *J* = 7.7 Hz, 1H), 2.70 (dd, *J* = 7.2, 4.2 Hz, 2H), 2.61 – 2.56 (m, 2H), 2.45 – 2.40 (m, 2H), 1.48 (s, 3H); the COOH proton signal was not detected. ^{13}**C NMR** (151 MHz, $CDCl_3$) δ 201.1 (>95% ^{13}C abundance), 176.9 (>95% ^{13}C abundance), 153.5 (d, *J* = 3.1 Hz), 133.2, 128.3 (d, *J* = 51.8 Hz), 127.0, 126.8 (d, *J* = 3.7 Hz), 121.9 (d, *J* = 3.7 Hz), 53.5 (d, *J* = 2.9 Hz, 2C), 49.1 (dd, *J* = 40.4, 2.0 Hz), 39.5, 38.3 (d, *J* = 55.7 Hz), 22.1. **HRMS (*ESI)*** m/z: $[M-H]^-$ calcd. for $^{12}C_{12}{}^{13}C_2H_{13}O_3$ 231.0937; found 231.0942.

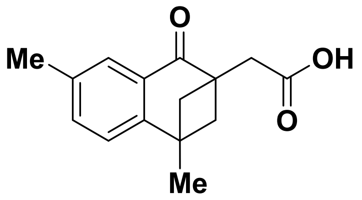

2b. White crystalline solid. FC eluent: *n*Hex/EtOAc 8:2 + 1% HCOOH. Yield = 62% (0.062 mmol, 15.1 mg). ^{1}**H NMR** (400 MHz, $CDCl_3$) δ 7.82 (s, 1H), 7.31 (d, *J* = 7.8 Hz, 1H), 7.14 (d, *J* = 7.8 Hz, 1H), 2.78 (s, 2H), 2.66 – 2.59 (m, 2H), 2.53 – 2.45 (m, 2H), 2.39 (s, 3H), 1.54 (s, 3H); the COOH proton signal was not detected. ^{13}**C NMR** (101 MHz, $CDCl_3$) δ 201.6, 175.8, 150.8, 136.5, 133.9, 128.1, 127.4, 121.8, 53.7 (2C), 49.1, 39.2, 38.4, 22.1, 20.9. **HRMS (*ESI)*** m/z: $[M-H]^-$ calcd. for $C_{15}H_{15}O_3$ 243.1027; found 243.1033.

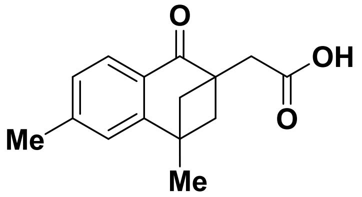

2c. White crystalline solid. FC eluent: *n*Hex/EtOAc 8:2 + 1% HCOOH. Yield = 67% (0.067 mmol, 16.3 mg). ^{1}**H NMR** (400 MHz, $CDCl_3$) δ 7.90 (d, *J* = 7.7 Hz, 1H), 7.16 (d, *J* = 7.7 Hz, 1H), 7.05 (s, 1H), 2.77 (s, 2H), 2.66 – 2.59 (m, 2H), 2.52 – 2.46 (m, 2H), 2.42 (s, 3H), 1.54 (s, 3H)¸ the COOH proton signal was not detected. ^{13}**C NMR** (101 MHz, $CDCl_3$) δ 201.1, 176.3, 153.6, 144.1, 127.4, 127.1, 125.9, 122.7, 53.5 (2C), 48.9, 39.3, 38.4, 22.13, 22.11. **HRMS (*ESI)*** m/z: $[M-H]^-$ calcd. for $C_{15}H_{15}O_3$ 243.1027; found 243.1022.

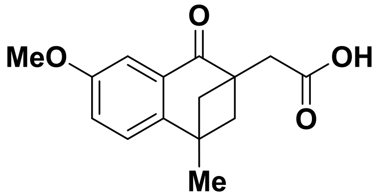

2d. White crystalline solid. FC eluent: *n*Hex/EtOAc 7:3 + 1% HCOOH. Yield = 64% (0.064 mmol, 16.7 mg). ^{1}**H NMR** (400 MHz, acetone-d_6) δ 7.45 (d, *J* = 2.8 Hz, 1H), 7.28 (d, *J* = 8.4 Hz, 1H), 7.12 (dd, *J* = 8.4, 2.8 Hz, 1H), 3.88 (s, 3H), 2.74 (s, 2H), 2.63 – 2.53 (m, 4H), 1.56 (s, 3H)¸ the COOH proton signal was not detected. ^{13}**C NMR** (101 MHz, acetone-d_6) δ

200.5, 172.5, 159.2, 146.9, 130.2, 123.8, 119.5, 110.9, 55.5, 53.9 (2C), 49.7, 39.4, 38.0, 22.0. **HRMS (*ESI)*** m/z: $[M-H]^-$ calcd. For $C_{15}H_{15}O_4$ 259.0976; found 259.0985.

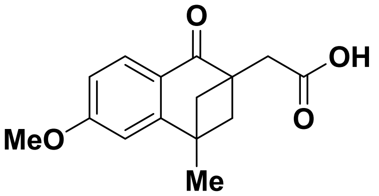

2e. White crystalline solid. FC eluent: *n*Hex/EtOAc 7:3 + 1% HCOOH. Yield = 53% (0.053 mmol, 13.8 mg). ^{1}**H NMR** (400 MHz, $CDCl_3$) δ 7.99 (d, *J* = 8.5 Hz, 1H), 6.83 (dd, *J* = 8.5, 2.4 Hz, 1H), 6.74 (d, *J* = 2.4 Hz, 1H), 3.89 (s, 3H), 2.78 (s, 2H), 2.66 – 2.59 (m, 2H), 2.52 – 2.44 (m, 2H), 1.53 (s, 3H)¸ the COOH proton signal was not detected. ^{13}**C NMR** (101 MHz, $CDCl_3$) δ 200.6, 175.6, 163.7, 156.0, 129.5, 121.6, 111.1, 108.5, 55.5, 53.4 (2C), 48.7, 39.6, 38.6, 22.1. **HRMS (*ESI)*** m/z: $[M-H]^-$ calcd. for $C_{15}H_{15}O_4$ 259.0976; found 259.0981.

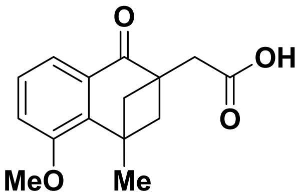

2f. White crystalline solid. FC eluent: *n*Hex/EtOAc 8:3 + 1% HCOOH. Yield = 32% (0.032 mmol, 8.3 mg). ^{1}**H NMR** (400 MHz, $CDCl_3$) δ 7.68 (dd, *J* = 7.6, 1.1 Hz, 1H), 7.27 (t, *J* = 7.9 Hz, 1H, partially overlapped with the residual solvent signal), 7.06 (d, *J* = 8.2 Hz, 1H), 3.80 (s, 3H), 2.76 (s, 2H), 2.75 – 2.71 (m, 2H), 2.45 – 2.37 (m, 2H), 1.77 (s, 3H)¸ the COOH proton signal was not detected. ^{13}**C NMR** (101 MHz, $CDCl_3$) δ ^{13}C NMR (101 MHz, $cdcl_3$) δ 201.4, 175.6, 156.0, 139.9, 130.5, 127.4, 119.9, 117.8, 55.9, 55.2 (2C), 48.8, 40.4, 38.3, 26.9. **HRMS (*ESI)*** m/z: $[M-H]^-$ calcd. for $C_{15}H_{15}O_4$ 259.0976; found 259.0980.

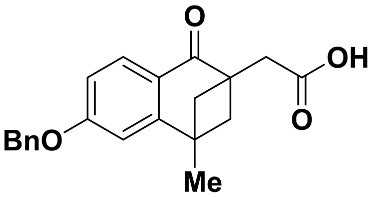

2g. White crystalline solid. FC eluent: *n*Hex/EtOAc 8:2 + 1% HCOOH. Yield = 49% (0.049 mmol, 16.5 mg). ^{1}**H NMR** (400 MHz, acetone-d_6) δ 7.87 (d, *J* = 8.4 Hz, 1H), 7.52 (d, *J* = 7.3 Hz, 2H), 7.46 – 7.39 (m, 2H), 7.38 – 7.32 (m, 1H), 7.00 (dd, *J* = 8.4, 2.3 Hz, 1H), 6.91 (d, *J* = 2.3 Hz, 1H), 5.24 (s, 2H), 2.68 (s, 2H), 2.60 – 2.47 (m, 4H), 1.53 (s, 3H)¸ the COOH proton signal was not detected. ^{13}**C NMR** (101 MHz, acetone-d_6) δ 199.4, 172.4, 163.1, 156.8, 137.5, 129.14 (2C), 129.11, 128.6, 128.3 (2C), 122.6, 112.6, 109.6, 70.5, 53.2 (2C), 49.1, 40.1, 37.8, 21.9. **HRMS (*ESI)*** m/z: $[M-H]^-$ calcd. for $C_{21}H_{19}O_4$ 335.1289; found 335.1296.

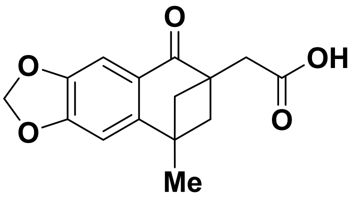

2h. White crystalline solid. FC eluent: *n*Hex/EtOAc 7:3 + 1% HCOOH up to 6:4 + 1% HCOOH. Yield = 74% (0.074 mmol, 20.3 mg). ^{1}**H NMR** (400 MHz, acetone-d_6) δ 7.33 (s, 1H), 6.87 (s, 1H), 6.12 (s, 2H), 2.70 (s, 2H), 2.61 – 2.50 (m, 4H), 1.54 (s, 3H)¸ the COOH proton signal was not detected. ^{13}**C NMR** (101 MHz, acetone-d_6) δ 199.1, 172.3, 151.9,

151.6, 146.8, 123.3, 106.2, 103.6, 102.3, 54.0 (2C), 49.0, 39.8, 37.7, 22.1. **HRMS (*ESI)*** m/z: [M-H]$^-$ calcd. for $C_{15}H_{13}O_5$ 273.0768; found 273.0762.

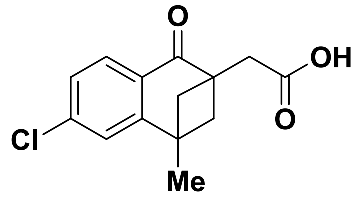

2i. White crystalline solid. FC eluent: *n*Hex/EtOAc 8:2 + 1% HCOOH up to 100% EtOAc + 1% HCOOH. Yield = 53% (0.053 mmol, 14.1 mg). **^{1}H NMR** (400 MHz, DMSO) δ 8.04 – 7.89 (m, 2H), 7.85 (s, 1H), 2.66 (s, 2H), 2.62 – 2.56 (m, 2H), 2.52 – 2.44 (m, 2H), 1.56 (s, 3H)¸ the COOH proton signal was not detected. **^{13}C NMR** (101 MHz, DMSO) δ 200.9, 173.3, 168.5, 154.4, 131.0, 128.4, 126.8, 123.5, 53.5 (2C), 50.0, 41.1, 38.5, 22.5. **HRMS (*ESI)*** m/z: [M-H]$^-$ calcd. for $C_{14}H_{12}{}^{35}ClO_3$ 263.0480; found 263.0477; calcd. for $C_{14}H_{12}{}^{37}ClO_3$ 265.0450; found 265.0439.

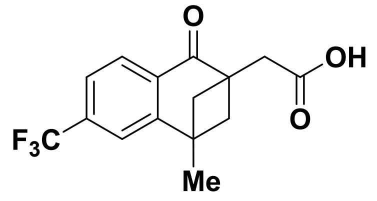

2j. White crystalline solid. FC eluent: *n*Hex/EtOAc 8:2 + 1% HCOOH. Yield = 33% (0.033 mmol, 9.8 mg). **^{1}H NMR** (400 MHz, $CDCl_3$) δ 8.10 (d, *J* = 7.8 Hz, 1H), 7.62 (d, *J* = 7.5 Hz, 1H), 7.49 (s, 1H), 2.78 (s, 2H), 2.70 – 2.62 (m, 2H), 2.58 – 2.47 (m, 2H), 1.59 (s, 3H)¸ the COOH proton signal was not detected. **^{13}C NMR** (101 MHz, $CDCl_3$) δ 199.8, 175.5, 154.1, 134.1, 131.0 (q, *J* = 1.0 Hz), 127.5, 127.4 (q, *J* = 190.8 Hz), 123.8 (q, *J* = 3.9 Hz), 119.1 (q, *J* = 3.8 Hz), 53.0 (2C), 39.7, 29.7, 21.9. **^{19}F NMR** (377 MHz, $CDCl_3$) δ -63.9 (s, 3F). **HRMS (*ESI)*** m/z: [M-H]$^-$ calcd. For $C_{15}H_{12}O_3F_3$ 297.0744; found 297.0750.

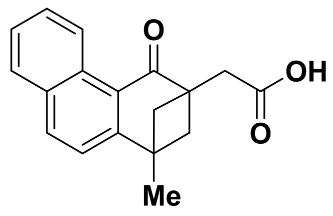

2k. White crystalline solid. FC eluent: *n*Hex/EtOAc 8:2 + 1% HCOOH. Yield = 57% (0.057 mmol, 16.0 mg). **^{1}H NMR** (400 MHz, acetone-d$_6$) δ 9.62 (d, *J* = 8.2 Hz, 1H), 8.18 (d, *J* = 8.5 Hz, 1H), 8.00 (d, *J* = 8.6 Hz, 1H), 7.68 (t, *J* = 7.1 Hz, 1H), 7.63 (d, *J* = 8.5 Hz, 1H), 7.56 (t, *J* = 7.0 Hz, 1H), 2.81 (s, 2H), 2.80 – 2.74 (m, 2H), 2.70 – 2.62 (m, 2H), 1.70 (s, 3H)¸ the COOH proton signal was not detected. **^{13}C NMR** (101 MHz, acetone-d$_6$) δ 203.2, 172.5, 157.2, 134.8, 133.8, 132.4, 129.3, 129.2, 126.5, 126.1, 121.5, 121.4, 55.1 (2C), 50.8, 40.6, 38.4, 22.9. **HRMS (*ESI)*** m/z: [M-H]$^-$ calcd. for $C_{18}H_{15}O_3$ 279.1027; found 279.1029.

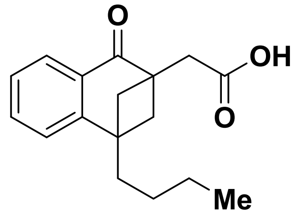

2l. White crystalline solid. FC eluent: *n*Hex/EtOAc 8:2 + 1% HCOOH. Yield = 67% (0.067 mmol, 18.2 mg). **^{1}H NMR** (400 MHz, $CDCl_3$) δ 8.02 (dd, *J* = 7.6, 1.3 Hz, 1H), 7.49 (td, *J* = 7.6, 1.4 Hz, 1H), 7.34 (td, *J* = 7.5, 1.0 Hz, 1H), 7.26 (d, *J* = 7.7 Hz, 1H), 2.78 (s, 2H), 2.63 – 2.55 (m, 2H), 2.55 – 2.46 (m, 2H), 1.93 – 1.85 (m, 2H), 1.51 – 1.35 (m, 4H), 0.99 (t, *J* = 7.0 Hz, 3H)¸ the COOH proton signal was not detected. **^{13}C NMR** (101 MHz, $CDCl_3$) δ 201.1, 176.8, 152.9, 133.1,

128.8, 127.1, 126.6, 122.3, 51.5 (2C), 48.9, 42.8, 38.3, 34.6, 26.8, 23.2, 14.1. **HRMS (*ESI*)** m/z: [M-H]$^-$ calcd. for $C_{17}H_{19}O_3$ 271.1340; found 271.1346.

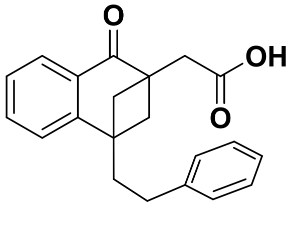

2m. White crystalline solid. FC eluent: *n*Hex/EtOAc 8:2 + 1% HCOOH. Yield = 66% (0.066 mmol, 21.1 mg). **^{1}H NMR** (400 MHz, acetone-d$_6$) δ 8.00 (d, *J* = 7.5 Hz, 1H), 7.63 (t, *J* = 7.1 Hz, 1H), 7.54 (d, *J* = 7.6 Hz, 1H), 7.46 – 7.34 (m, 5H), 7.25 (t, *J* = 7.0 Hz, 1H), 2.87 – 2.80 (m, 2H), 2.79 (s, 2H), 2.77 – 2.70 (m, 2H), 2.64 – 2.56 (m, 2H), 2.34 – 2.26 (m, 2H); the COOH proton signal was not detected. **^{13}C NMR** (101 MHz, acetone-d$_6$) δ 200.4, 172.4, 153.3, 142.9, 133.5, 129.7, 129.0 (2C), 128.9 (2C), 127.12, 127.10, 126.4, 123.1, 51.6 (2C), 49.4, 43.3, 37.8, 37.4, 31.3. **HRMS (*ESI*)** m/z: [M-H]$^-$ calcd. for $C_{21}H_{19}O_3$ 319.1340; found 319.1328.

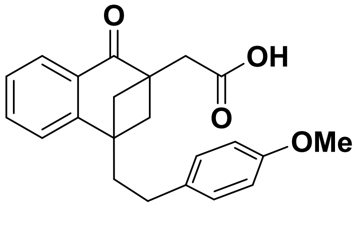

2n. White crystalline solid. FC eluent: *n*Hex/EtOAc 8:2 + 1% HCOOH. Yield = 74% (0.074 mmol, 25.9 mg). **^{1}H NMR** (400 MHz, acetone-d$_6$) δ 7.93 (d, *J* = 7.5 Hz, 1H), 7.56 (t, *J* = 6.9 Hz, 1H), 7.46 (d, *J* = 7.6 Hz, 1H), 7.38 (t, *J* = 7.5 Hz, 1H), 7.25 (d, *J* = 8.5 Hz, 2H), 6.88 – 6.85 (m, 2H), 3.76 (s, 3H), 2.75 – 2.64 (m, 6H), 2.56 – 2.50 (m, 2H), 2.24 – 2.16 (m, 2H)¸ the COOH proton signal was not detected. **^{13}C NMR** (101 MHz, acetone-d$_6$) δ 200.4, 172.4, 158.7, 153.4, 134.7, 133.5, 129.8 (2C), 127.1, 127.0, 123.1, 114.4 (2C), 55.1 (2C), 51.6, 49.5, 43.3, 37.8, 37.7, 30.4. **HRMS (*ESI*)** m/z: [M-H]$^-$ calcd. for $C_{22}H_{21}O_4$ 349.1445; found 349.1449.

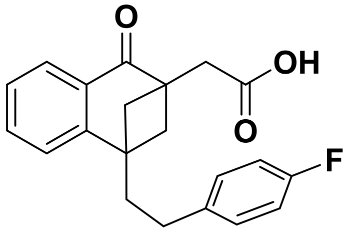

2o. White crystalline solid. FC eluent: *n*Hex/EtOAc 8:2 + 1% HCOOH. Yield = 53% (0.053 mmol, 17.9 mg). **^{1}H NMR** (400 MHz, , acetone-d$_6$) δ 7.99 (d, *J* = 7.6 Hz, 1H), 7.62 (t, *J* = 7.5 Hz, 1H), 7.53 (d, *J* = 7.6 Hz, 1H), 7.46 – 7.42 (m, 3H), 7.14 – 7.10 (m, 2H), 2.88 – 2.81 (m, 2H), 2.80 – 2.70 (m, 4H), 2.63 – 2.55 (m, 2H), 2.33 – 2.25 (m, 2H)¸ the COOH proton signal was not detected. **^{13}C NMR** (101 MHz, , acetone-d$_6$) δ ^{13}C NMR (101 MHz, acetone-d$_6$) δ 200.4, 172.4, 161.9 (d, *J* = 241.6 Hz), 153.3, 139.0 (d, *J* = 3.2 Hz), 133.6, 130.6 (d, *J* = 7.9 Hz, 2C), 129.7, 127.2, 127.1, 123.1, 115.5 (d, *J* = 21.1 Hz, 2C), 51.6 (2C), 49.5, 43.3, 37.8, 37.5, 30.5. **^{19}F NMR** (377 MHz, acetone-d$_6$) -119.17 – -119.30 (m, 1F). **HRMS (*ESI*)** m/z: [M-H]$^-$ calcd. for $C_{21}H_{18}FO_3$ 337.1245; found 337.1237.

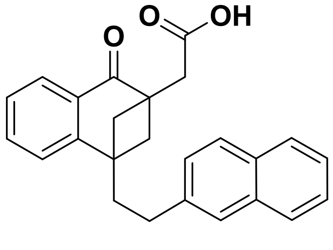

2p. White crystalline solid. FC eluent: *n*Hex/EtOAc 8:2 + 1% HCOOH. Yield = 58% (0.058 mmol, 21.5 mg). **^{1}H NMR** (400 MHz, acetone-d$_6$) δ 8.01 (d, *J* = 7.5 Hz, 1H), 7.97 – 7.85 (m, 4H), 7.67 – 7.55 (m, 3H), 7.55

– 7.41 (m, 3H), 3.05 – 2.96 (m, 2H), 2.86 – 2.74 (m, 4H), 2.68 – 2.60 (m, 2H), 2.45 – 2.36 (m, 2H), the COOH proton signal was not detected. **^{13}C NMR** (101 MHz, acetone-d$_6$) δ 200.4, 172.4, 153.4, 140.6, 134.5, 133.6, 132.9, 129.8, 128.6, 128.2, 128.03, 128.01, 127.2, 127.1, 126.8, 126.5, 125.8, 123.2, 51.7 (2C), 49.5, 43.4, 37.9, 37.3, 31.5. **HRMS (*ESI)*** m/z: [M-H]$^-$ calcd. for $C_{25}H_{21}O_3$ 369.1496; found 369.1506.

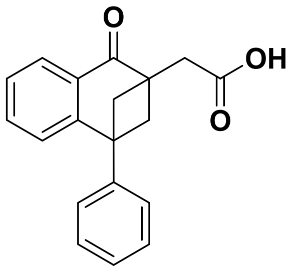

2q. White crystalline solid. FC eluent: *n*Hex/EtOAc 8:2 + 1% HCOOH. Yield = 45% (0.045 mmol, 13.1 mg). **^{1}H NMR** (400 MHz, acetone-d$_6$) δ 8.03 – 7.96 (m, 1H), 7.53 – 7-50 (m, 2H), 7.44 – 7.39 (m, 3H), 7.30 – 7.28 (m, 2H), 6.52 – 6.45 (m, 1H), 3.14 – 3.06 (m, 2H), 3.03 – 2.96 (m, 2H), 2.84 (s, 2H)¸ the COOH proton signal was not detected. **^{13}C NMR** (101 MHz, acetone-d$_6$) δ 199.9, 172.4, 155.1, 144.5, 133.4, 129.3 (2C), 129.1, 127.5, 127.4, 127.1 (2C), 126.9, 124.7, 52.4 (2C), 49.6, 48.1, 37.7. **HRMS (*ESI)*** m/z: [M-H]$^-$ calcd. for $C_{19}H_{15}O_3$ 291.1027; found 291.1033.

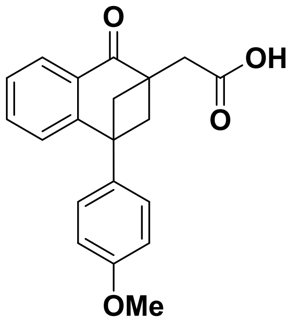

2r. White crystalline solid. FC eluent: *n*Hex/EtOAc 7:3 + 1% HCOOH. Yield = 45% (0.045 mmol, 14.5 mg). **^{1}H NMR** (400 MHz, $CDCl_3$) δ 8.06 – 7.99 (m, 1H), 7.36 – 7.28 (m, 2H), 7.10 – 7-07 (m, 2H), 6.99 – 6-96 (m, 2H), 6.58 – 6.46 (m, 1H), 3.87 (s, 3H), 3.13 – 3.02 (m, 2H), 2.91 – 2.85 (m, 2H), 2.81 (s, 2H)¸ the COOH proton signal was not detected. **^{13}C NMR** (101 MHz, $CDCl_3$) δ 200.5, 175.8, 158.5, 154.3, 135.3, 133.1, 128.0, 127.5 (2C), 126.94, 126.90, 124.3, 114.1 (2C), 55.3 (2C), 52.4, 49.0, 47.0, 29.7. **HRMS (*ESI)*** m/z: [M+Na]$^+$ calcd. for $C_{20}H_{18}O_4Na$ 345.1097; found 345.1094. **HRMS (*ESI)*** m/z: [M-H]$^-$ calcd. for $C_{20}H_{17}O_4$ 321.1132; found 321.1126.

5.2.1 Crystal data and structure refinement for compounds 2a

SC-XRD suitable crystals of **2a** were obtained from slow evaporation of it in a 1 to 1 DCM/EtOAc mixture. Colourless needle crystals were observed.

Fw	230.25
T, K	296(2)
λ, Å	0.71073
Crystal symmetry	Monoclinic
Space group	P 2_1/c
a, Å	12.9969(6)
b, Å	5.9499(3)
c, Å	15.2846(7)
α	90
β	93.085(2)
γ	90
Cell volume, $Å^3$	1180.2(1)
Z	4
D_c, Mg m^{-3}	1.296
μ(Mo-K_α), mm^{-1}	0.091
F(000)	488
Crystal size/ mm	0.62 x 0.23 x 0.18
θ limits, °	1.569 to 25.491
Reflections collected	17488
Unique obs. Reflections [$F_o > 4\sigma(F_o)$]	2194 [R(int) = 0.0531]
Goodness-of-fit-on F^2	1.070
R_1 (F)[a], wR_2 (F^2) [I > 2σ(I)][b]	R1 = 0.0505, wR2 = 0.1240
Largest diff. peak and hole, e. $Å^{-3}$	0.181 and -0.205

Table S4. [a)] $R_1 = \Sigma||F_o|-|F_c||/\Sigma|F_o|$. [b] $wR_2 = [\Sigma w(F_o^2-F_c^2)^2/\Sigma w(F_o^2)^2]^{1/2}$ where $w = 1/[\sigma^2(F_o^2) + (aP)^2 + bP]$ where $P = (F_o^2 + F_c^2)/3$.

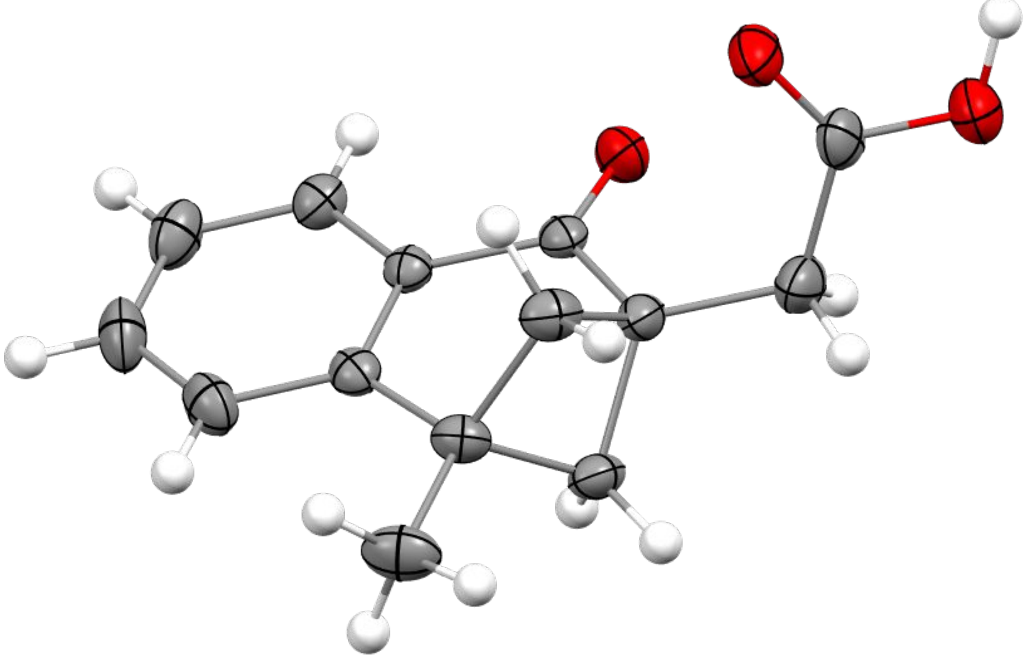

Figure S4. ORTEP drawing of **2a**. Thermal ellipsoid are drawn at 30% of the probability level.

6. Functionalization and derivatization of products

6.1 Electrochemical derivatization for the preparation of cinnamate 5

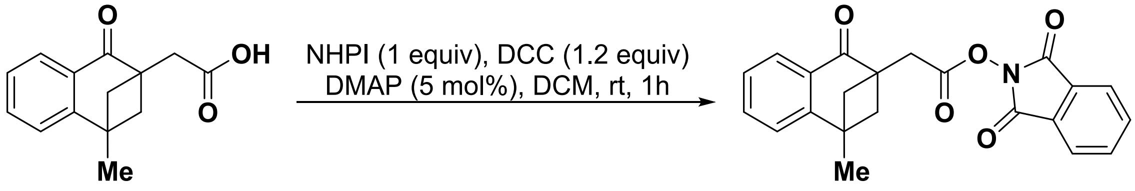

2a **3**

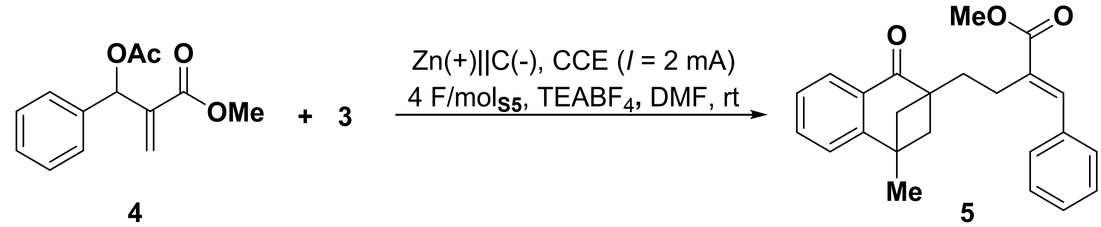

4 **5**

STEP 1. Compound **3** was synthesized according to a modified literature procedure[18]

In a Schlenk tube, under N_2 flow and magnetic stirring, acid **2a** (138 mg, 0.6 mmol) and *N*-hydroxyphthalimide (NHPI, 1 equiv, 97.8 mg, 0.6 mmol), were suspended in dry DCM (5 mL). Then, DCC (148.3 mg, 0.72 mmol) and DMAP (5 mol%, 3.7 mg) were added and the resulting orange suspension was stirred at room temperature until the color disappeared (1 h). The reaction mixture was then filtered over a Celite pad, washed with DCM (5 mL) and evaporated *in vacuo*. The crude product was finally purified by FC to afford pure product **3**.

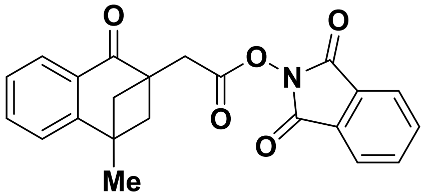

3. White crystalline solid. FC eluent: *n*Hex/EtOAc 5:1 up to 1:1. Yield = 51% (0.31 mmol, 191.3 mg). **^{1}H NMR** (400 MHz, $CDCl_3$) δ 8.05 (dd, *J* = 7.6, 1.0 Hz, 1H), 7.93 – 7.87 (m, 2H), 7.84 – 7.77 (m, 2H), 7.51 (td, *J* = 7.6, 1.4 Hz, 1H), 7.37 (td, *J* = 7.5, 1.0 Hz, 1H), 7.27 (d, *J* = 7.5 Hz, 1H), 3.10 (s, 2H), 2.79 – 2.70 (m, 2H), 2.67 – 2.58 (m, 2H), 1.61 (s, 3H). **^{13}C NMR** (101 MHz, $CDCl_3$) δ 199.5, 167.7 (2C), 161.9, 153.4, 134.7 (2C), 133.3 (2C), 128.9, 128.0, 127.2, 126.8, 123.9 (2C), 121.9, 52.3 (2C), 48.8, 39.0, 34.4, 21.9. **HRMS (*ESI)*** m/z: $[M+Na]^+$ calcd. for $C_{22}H_{17}NO_5Na$ 398.0999; found 398.0996.

STEP 2. Compound **5** was synthesized according to our previously reported literature procedure[19] The ElectraSyn vial (5 mL), equipped with a stir bar, was charged with Morita-Baylis-Hillman acetate **4** (0.15 mmol, 35.0 mg), **3** (0.30 mmol, 112.5 mg) and $TEABF_4$ (0.30 mmol, 65.0 mg). The ElectraSyn

[18] C. V. Deliwala, M. Y. Mhasalkar, M. H. Shah, S. T. Nikam, K. G. Anantanarayanan, *J. Med. Chem.* **1971***, 14,* 262-264.

[19] G. Bertuzzi, G. Ombrosi, M. Bandini, *Org. Lett.* **2022***, 24,* 4354-4359

vial cap, equipped with anode (Zn) and cathode (graphite), was inserted into the mixture and closed with a rubber septum. The vessel was evacuated and backfilled with N_2 three times, then dry DMF (3.0 mL) was added and the mixture stirred until complete dissolution of the solids occurred. The reaction mixture was electrolyzed (under N_2, balloon) at a constant current of 2 mA, until a total charge of 0.6 F (4 F/mol_{I4}) was reached. The ElectraSyn vial cap was removed, and the electrodes and vial were rinsed with EtOAc (10 mL) and $HCl_{(aq)}$ (1 M, 10 mL), which were combined with the crude mixture in a separatory funnel. Then, the organic layer was separated, the aqueous layer was extracted with EtOAc (2 x 10 mL) and the combined organic layers were washed with $HCl_{(aq)}$ (0.1 M, 3 x 10 mL), dried over Na_2SO_4 and concentrated *in vacuo*. The crude product was finally purified by FC to afford pure product **5**.

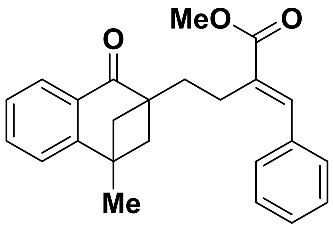

5. Viscous oil. FC eluent: *n*Hex/EtOAc 10:1. Yield = 31% (0.047 mmol, 16.7 mg). *E*/*Z* > 20:1. **^{1}H NMR** (400 MHz, $CDCl_3$) δ 8.02 (d, *J* = 7.6 Hz, 1H), 7.71 (s, 1H), 7.65 – 7-63 (m, 2H), 7.52 – 7.44 (m, 3H), 7.40 – 7.31 (m, 2H), 7.25 (d, *J* = 7.5 Hz, 1H), 3.86 (s, 3H), 2.68 – 2.59 (m, 2H), 2.56 – 2.49 (m, 2H), 2.47 – 2.39 (m, 2H), 2.05 – 1.95 (m, 2H), 1.54 (s, 3H). **^{13}C NMR** (101 MHz, $CDCl_3$) δ 202.2, 169.0, 153.7, 139.6, 135.4, 132.8, 132.4, 129.7 (2C), 129.1, 128.64 (2C), 128.61, 126.6, 126.5, 121.7, 53.1 (2C), 52.0, 51.7, 38.6, 33.0, 22.8, 22.2. **HRMS (*ESI)*** m/z: $[M+Na]^+$ calcd. for $C_{24}H_{24}O_3Na$ 383.1618; found 383.1616.

6.2 Synthesis and characterization of Carbamate 6

Compound **6** was synthesized *via* a Curtius rearrangement, according to a modified literature procedure [20]

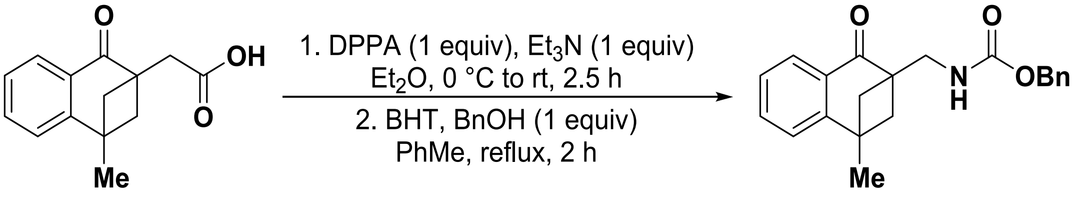

2a **6**

In a Schlenk tube, acid **2a** (138 mg, 0.6 mmol) was suspended in dry Et_2O (5 mL) under nitrogen atmosphere. The solution was cooled in ice bath and dry Et_3N (83 µL, 0.6 mmol) was added. After stirring for 5 minutes, diphenyl phosphoryl azide (129 µL, 0.6 mmol) was added and the reaction was warmed up to room temperature. After stirring for 2.5 h, the reaction was quenched with sat. $NaHCO_{3(aq.)}$ and the mixture was extracted three times with Et_2O. The combined organic layer was washed with brine, dried over Na_2SO_4 and evaporated. The resulting brown oil was used immediately in the next step without further purification. A solution of 2,6-di-*tert*-butyl-4-methylphenol (1.0 mg) and benzyl alcohol (1.0 mL) in dry toluene (2.0 mL) was heated under reflux. To the refluxed solution was added dropwise a solution of the crude acyl azide in dry toluene (1.5 mL) over 5 min (gas evolution was observed during the addition). After refluxing for 2 h, the reaction was cooled to room temperature and then concentrated (high vacuum). The resulting crude product was purified by silica gel chromatography (n-Hex/EtOAc 5:1) to give title compound **6**.

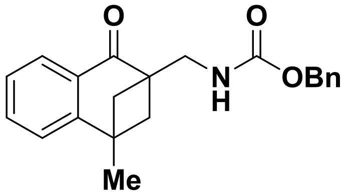

6. Viscous oil. FC eluent: from *n*Hex/EtOAc 5:1 to 4:1. Yield = 50% (0.30 mmol, 100.5 mg). **^{1}H NMR** (400 MHz, $CDCl_3$) δ 7.98 (d, *J* = 7.6 Hz, 1H), 7.50 (t, *J* = 7.5 Hz, 1H), 7.44 – 7.17 (m, 8H), 5.09 (s, 2H), 3.57 (d, *J* = 6.5 Hz, 2H), 2.48 (s, 4H), 1.55 (s, 3H); the NH proton signal was not detected. **^{13}C NMR** (101 MHz, $CDCl_3$) δ 202.4, 156.9, 153.6, 136.6, 133.3 (2C), 128.5, 128.4 (2C), 128.0, 127.9, 126.7, 126.6, 122.0, 66.6, 51.7, 51.5 (2C), 44.0, 38.7, 22.0. **HRMS (*ESI)*** m/z: $[M+Na]^+$ calcd. for $C_{21}H_{21}NO_3Na$ 358.1414; found 358.1412.

[20] Y. Nishikawa , S. Nakano ,Y. Tahira, K. Terazawa, K. Yamazaki, C. Kitamura, and O. Hara, *Org. Lett.* **2016**, *18*, 2004-2007.

6.3 Synthesis and characterization of Pyridazinone 7

Compound **7** was synthesized according to a modified literature procedure[21]. In a 4 mL screw capped vial, product **2a** (0.09 mmol, 20.8 mg) was dissolved in ethanol (0.04 M, 2.25 mL). Under magnetic stirring, monohydrated NH_2NH_2 (64-65%, 2 equiv, 8.7 µL) was added, then the vial was sealed, and the solution refluxed for 3 hours. A second portion of monohydrated NH_2NH_2 (2 equiv, 8.7 µL) was added to the reaction and the mixture was refluxed again for 3 hours. Reaction was monitored by TLC (7:3 *n*Hex:AcOEt + 1% HCOOH) and showed complete conversion. Pure product **7** was directly obtained by drying the reaction mixture under vacuum for several hours without any further purification.

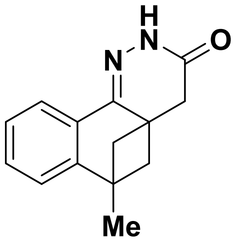

7. Pale yellow crystalline solid. FC eluent: *n*Hex/EtOAc 7:3 + 1% HCOOH. Yield = 74% (0.067 mmol, 15.2 mg). **^{1}H NMR** (400 MHz, $CDCl_3$) δ 8.60 (bs, 1H), 7.93 (dd, *J* = 7.3, 1.4 Hz, 1H), 7.33 – 7-29 (m, 3H), 7.20 (d, *J* = 7.6 Hz, 1H), 2.83 (s, 2H), 2.49 – 2.39 (m, 2H), 2.22 – 2.11 (m, 2H), 1.51 (s, 3H). **^{13}C NMR** (101 MHz, $CDCl_3$) δ 164.6, 153.4, 148.7, 129.5, 128.4, 126.7, 123.7, 122.1, 53.2 (2C), 39.4, 38.7, 37.6, 22.2. **HRMS (*ESI*)** m/z: $[M+H]^+$ calcd. for $C_{14}H_{15}N_2O$ 227.1179; found 227.1179.

[21] C. V. Deliwala, M. Y. Mhasalkar ,M. H. Shah, S. T. Nikam, K. G. Anantanarayanan, *J. Med. Chem.* **1971***, 14,* 262-264.

6.4 Synthesis of Lactone 9

Compound **9** was synthesized according to a modified literature procedure.[22]

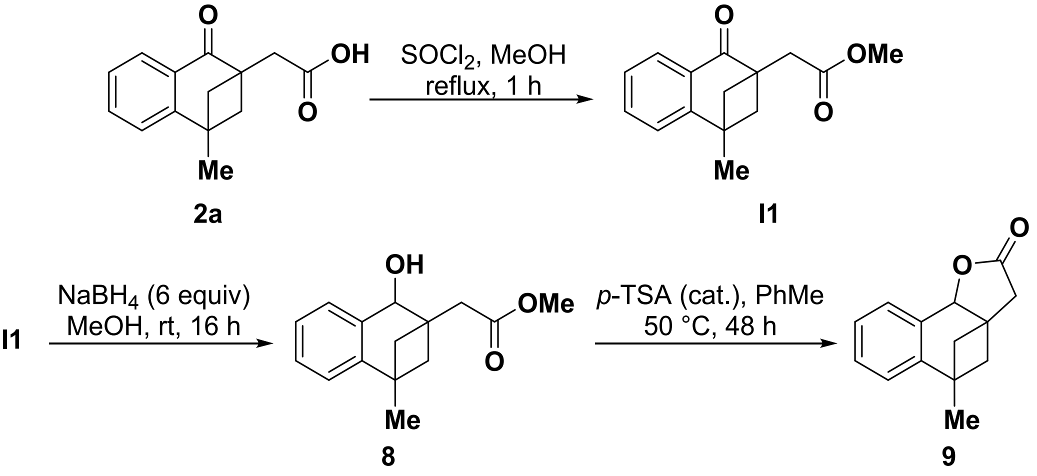

STEP 1. In a 4 mL screw capped vial, product **2a** (0.1 mmol) was dissolved in methanol (0.05 M, 2 mL), one drop of $SOCl_2$ was added and the solution was heated to reflux for 1 hour. Complete starting material conversion was confirmed by TLC (8:2 *n*Hex:AcOEt + 1% HCOOH). The reaction mixture was dried under vacuum for several hours to afford methyl ester **I1** showing sufficient purity to be used in the next step without further purification.

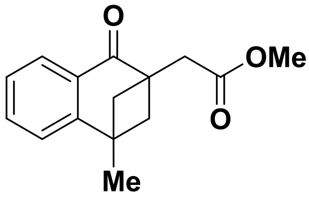

I1. Pale yellow crystalline solid. Yield = 98% (0.098 mmol, 23.9 mg). **^{1}H NMR** (400 MHz, $CDCl_3$) δ 8.00 (d, *J* = 7.5 Hz, 1H), 7.49 (t, *J* = 7.5 Hz, 1H), 7.35 (t, *J* = 7.5 Hz, 1H), 7.25 (d, *J* = 7.6 Hz, 1H), 3.70 (s, 3H), 2.74 (s, 2H), 2.69 – 2.61 (m, 2H), 2.53 – 2.45 (m, 2H), 1.55 (s, 3H). **^{13}C NMR** (101 MHz, $CDCl_3$) δ 200.8, 172.0, 153.5, 133.0, 128.4, 126.9, 126.7, 121.8, 53.4 (2C), 51.5, 49.2, 39.5, 37.9, 22.1. **HRMS (*ESI)*** m/z: $[M+H]^+$ calcd. for $C_{15}H_{17}O_3$ 245.1172; found 245.1167.

STEP 2. In a 4 mL screw capped vial, under air methyl ester **I1** (0.098 mmol, 24.1 mg) was dissolved in methanol (0.03 M, 3 mL), $NaBH_4$ (6 equiv, 0.59 mmol, 22.2 mg) were added then the reaction mixture was stirred for 16 hours (providing sufficient venting for the developing hydrogen gas). The reaction was monitored by TLC (8:2 *n*Hex:AcOEt + 1% HCOOH) showing satisfactory conversion. The reaction crude was quenched with HCl (2M, 3 mL) and extracted with EtOAc (3x5 mL), the organic phase was dried over Na_2SO_4 and the solvent was removed *in vacuo*. The reaction crude was purified by FC to afford alcohol **8.**

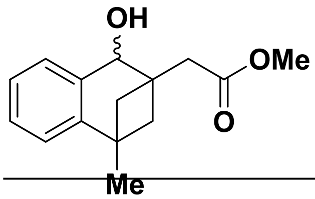

8. Viscous oil. FC eluent: *n*Hex/EtOAc 8:2. Yield = 65% (0.064 mmol, 15.8 mg). **^{1}H NMR** (400 MHz, $CDCl_3$) δ 7.51 (dd, *J* = 7.1, 1.0 Hz, 1H), 7.28 –

[22] S.-T. Yang, J.-B. fan, T.-T. Liu, S. Ning, J.-H. Xu, Y.-J. Zhou, X. Deng *J. Med. Chem.* **2022**, *65*, 9706.

7.24 (m, 1H), 7.21 (td, *J* = 7.4, 1.6 Hz, 1H), 7.11 (dd, *J* = 7.5, 1.3 Hz, 1H), 4.90 (s, 1H), 3.70 (s, 3H), 2.81 (d, *J* = 14.6 Hz, 1H), 2.59 (d, *J* = 14.6 Hz, 1H), 2.34 (bs, 1H), 2.07 – 2.04 (m, 1H), 2.00 – 1.92 (m, 1H), 1.92 – 1.83 (m, 2H), 1.45 (s, 3H. **^{13}C NMR** (101 MHz, $CDCl_3$) δ 172.7, 149.0, 136.7, 128.5, 127.1, 126.4, 121.1, 74.9, 51.5 (2C), 47.8, 41.5, 41.3, 39.0, 22.2. **HRMS (*ESI)*** m/z: $[M+H]^+$ calcd. for $C_{15}H_{19}O_3$ 247.1329; found 247.1331.

STEP 3. In a 4 mL screw capped vial, under air, alcohol **8** (0.060 mmol, 14.8 mg) was dissolved in toluene (0.04 M, 1.5 mL), then toluene-4-sulfonic acid monohydrated (*p*-TSA·H_2O, 1 mg) was added and the reaction mixture was heated at 50°C for 2 days. Reaction was monitored by TLC (3:1 *n*Hex:AcOEt + 1% HCOOH) showing satisfactory conversion. The reaction crude was dried under vacuum and directly purified by FC to afford lactone **9**.

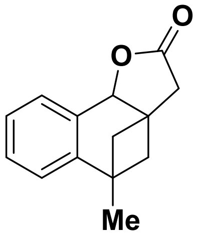

9. White crystalline solid. FC eluent: *n*Hex/EtOAc 7:2. Yield = 55% (0.033 mmol, 7.1 mg). **^{1}H NMR** (400 MHz, $CDCl_3$) δ 7.55 – 7.50 (m, 1H), 7.35 – 7.28 (m, 2H), 7.25 – 7.19 (m, 1H), 5.37 (s, 1H), 2.72 (d, *J* = 16.6 Hz, 1H), 2.54 (d, *J* = 16.6 Hz, 1H), 2.37 (d, *J* = 9.3 Hz, 1H), 2.07 (t, *J* = 9.4 Hz 1H), 1.89 (d, *J* = 8.7 Hz, 1H), 1.80 (t, *J* = 8.3 Hz, 1H), 1.51 (s, 3H). **^{13}C NMR** (101 MHz, $CDCl_3$) δ 175.6, 148.5, 129.6, 128.33, 128.31, 126.8, 122.5, 84.4, 44.3 (2C), 43.5, 43.4, 39.9, 22.2. **HRMS (*ESI)*** m/z: $[M+Na]^+$ calcd. for $C_{14}H_{14}O_2Na$ 237.0886; found 237.0879.

7. ^{1}H-, ^{13}C-, ^{19}F-NMR Spectra

1a ^{1}HNMR (400 MHz, $CDCl_3$)

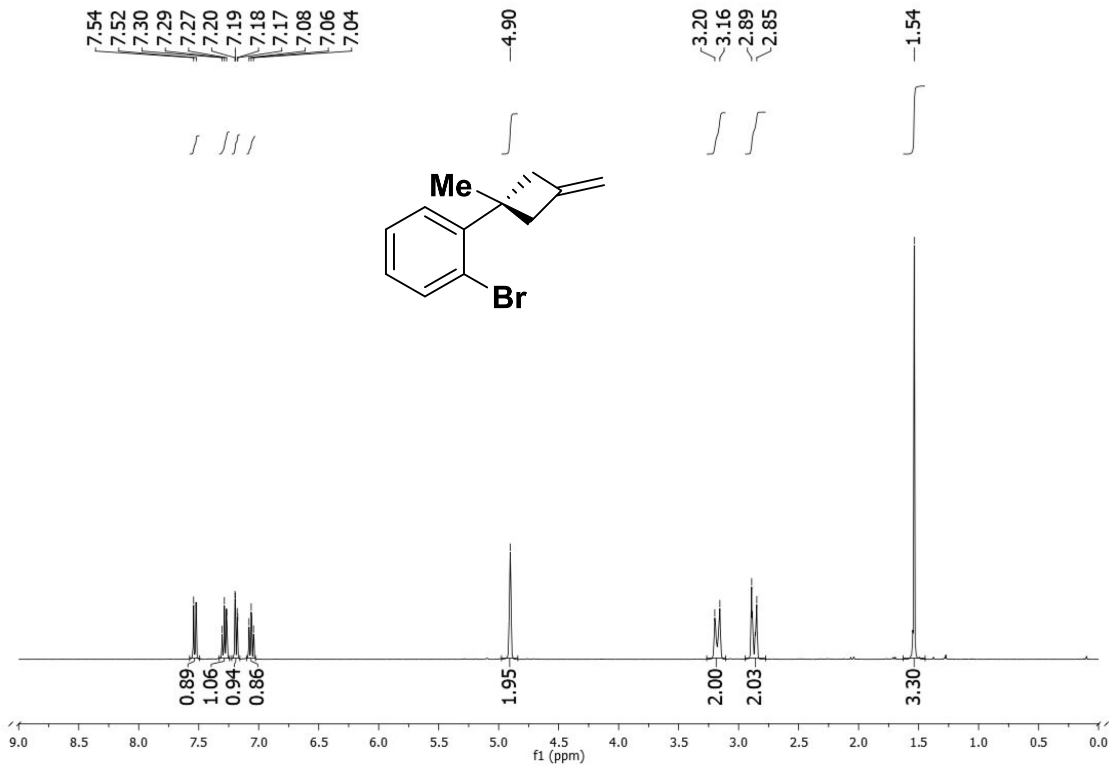

1a ^{13}CNMR (100 MHz, $CDCl_3$)

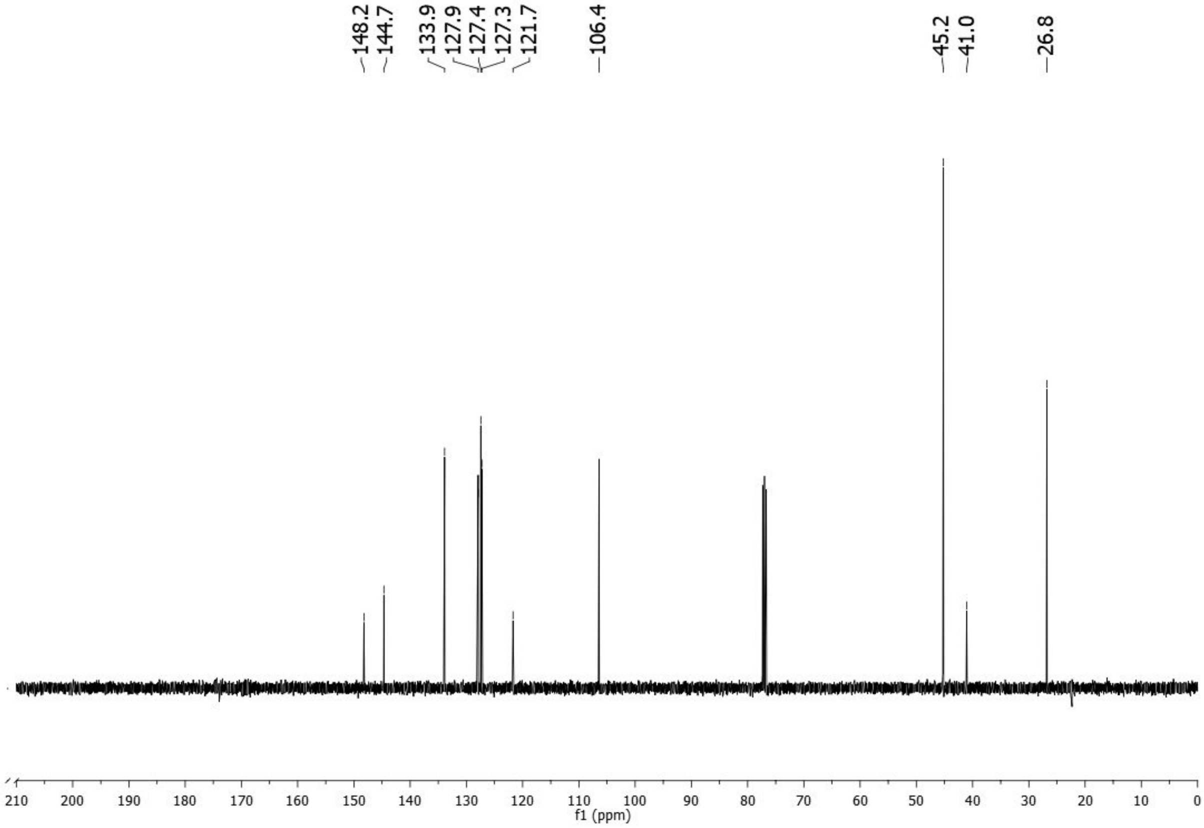

1b ^{1}HNMR (400 MHz, $CDCl_3$)

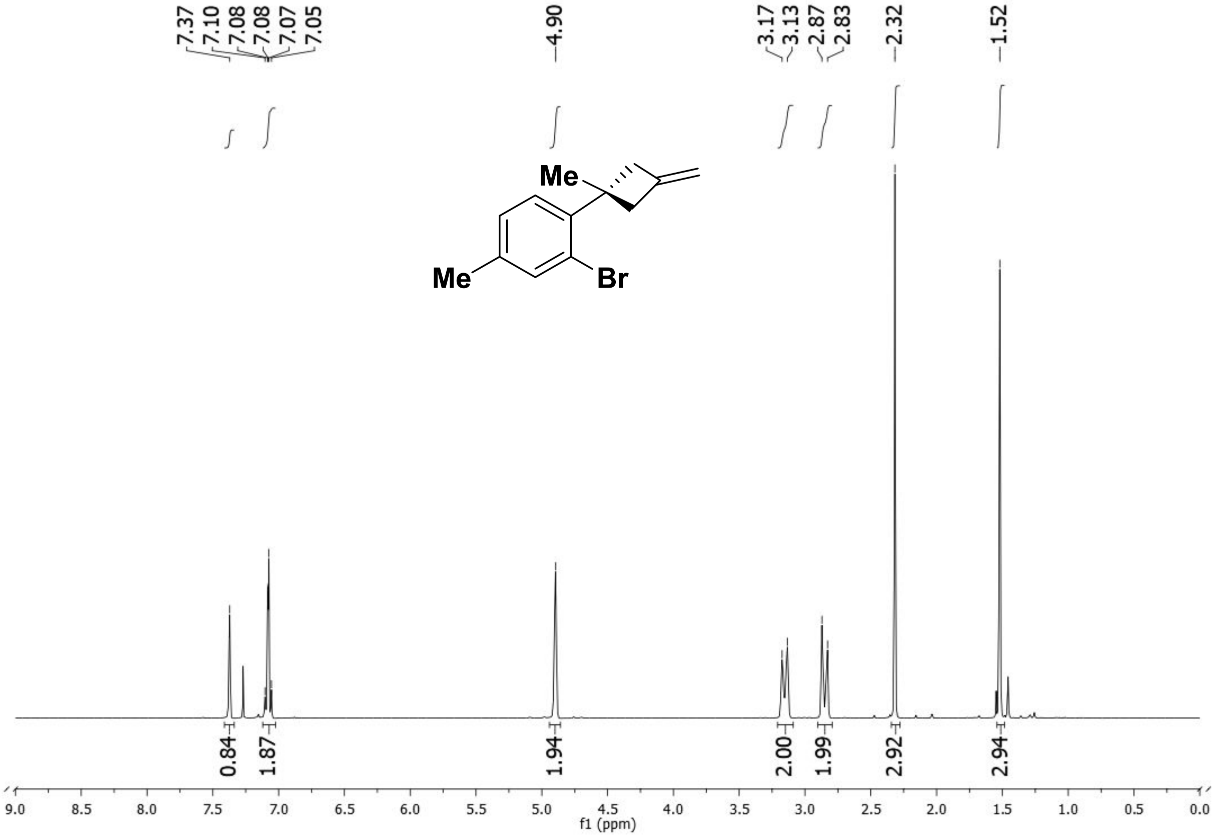

1b ^{13}CNMR (100 MHz, $CDCl_3$)

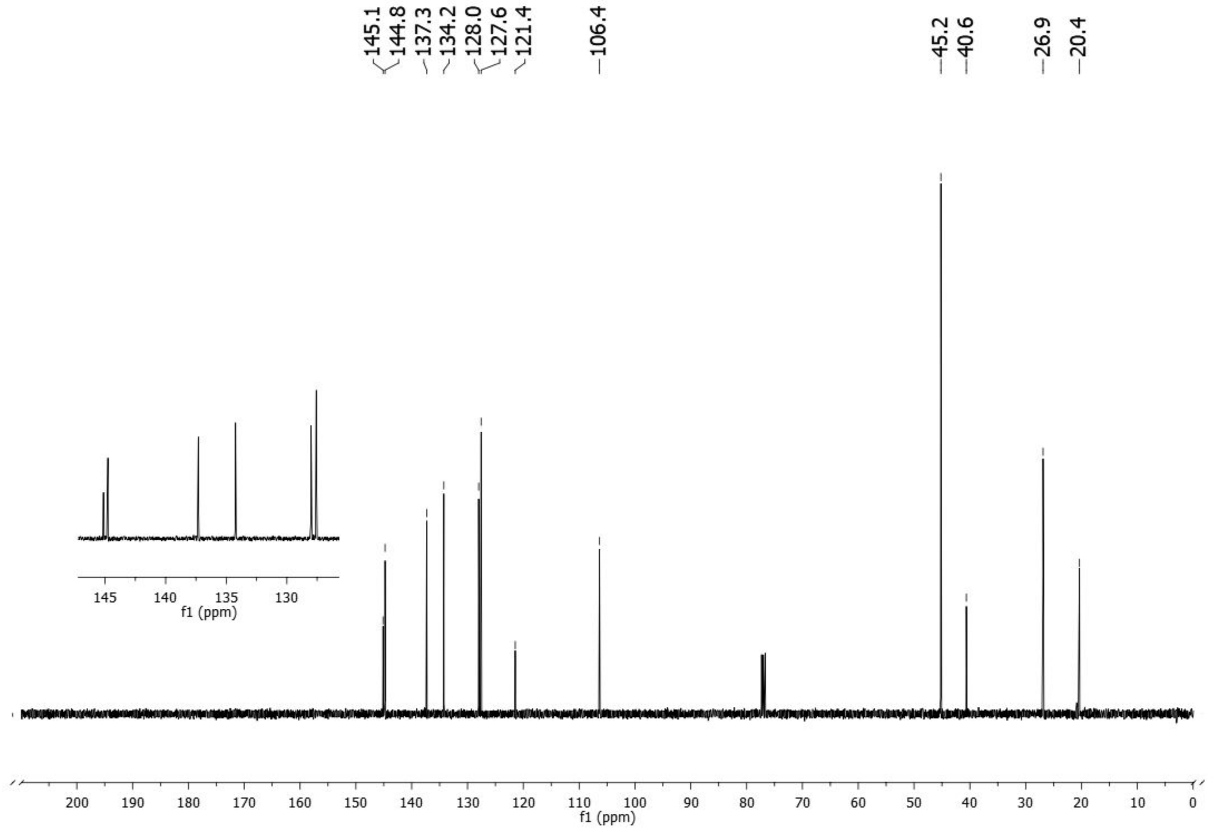

1c ^{1}HNMR (400 MHz, $CDCl_3$)

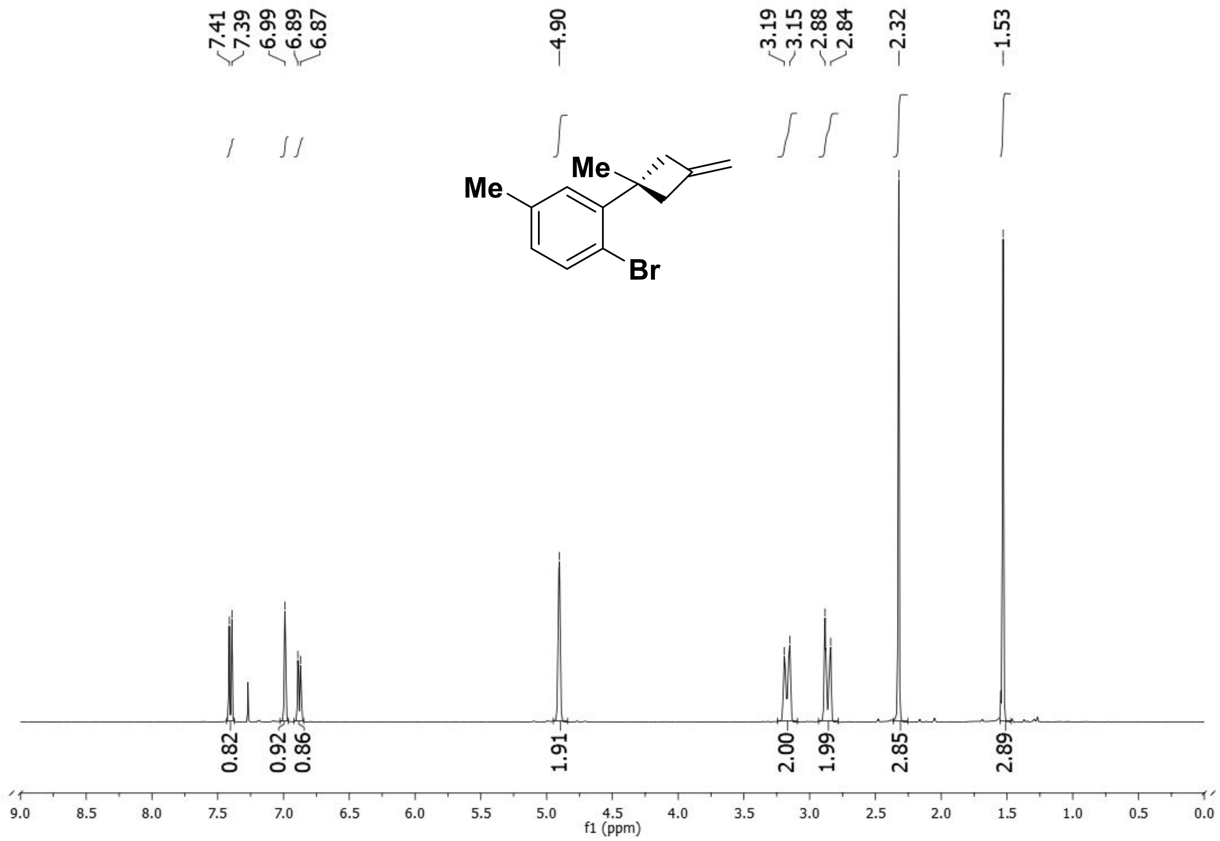

1c ^{13}CNMR (100 MHz, $CDCl_3$)

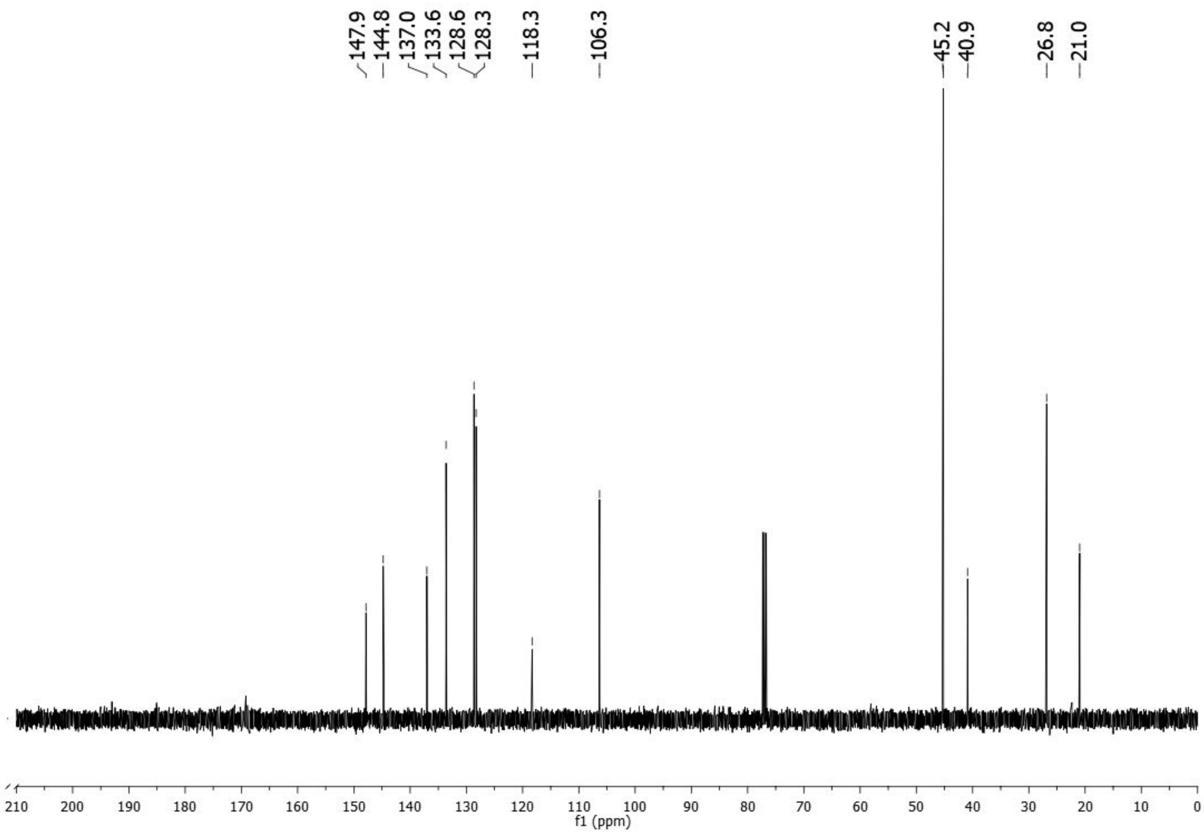

1d ^{1}HNMR (400 MHz, $CDCl_3$)

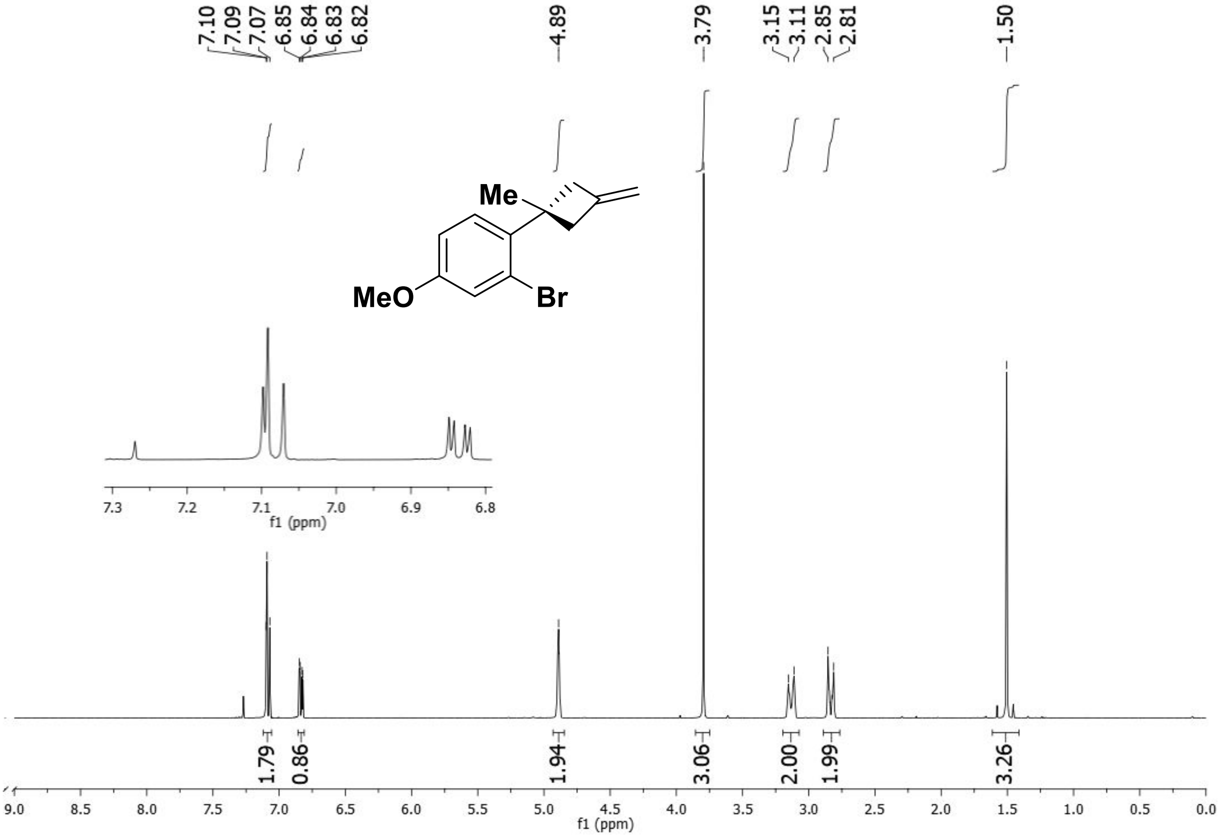

1d ^{13}CNMR (100 MHz, $CDCl_3$)

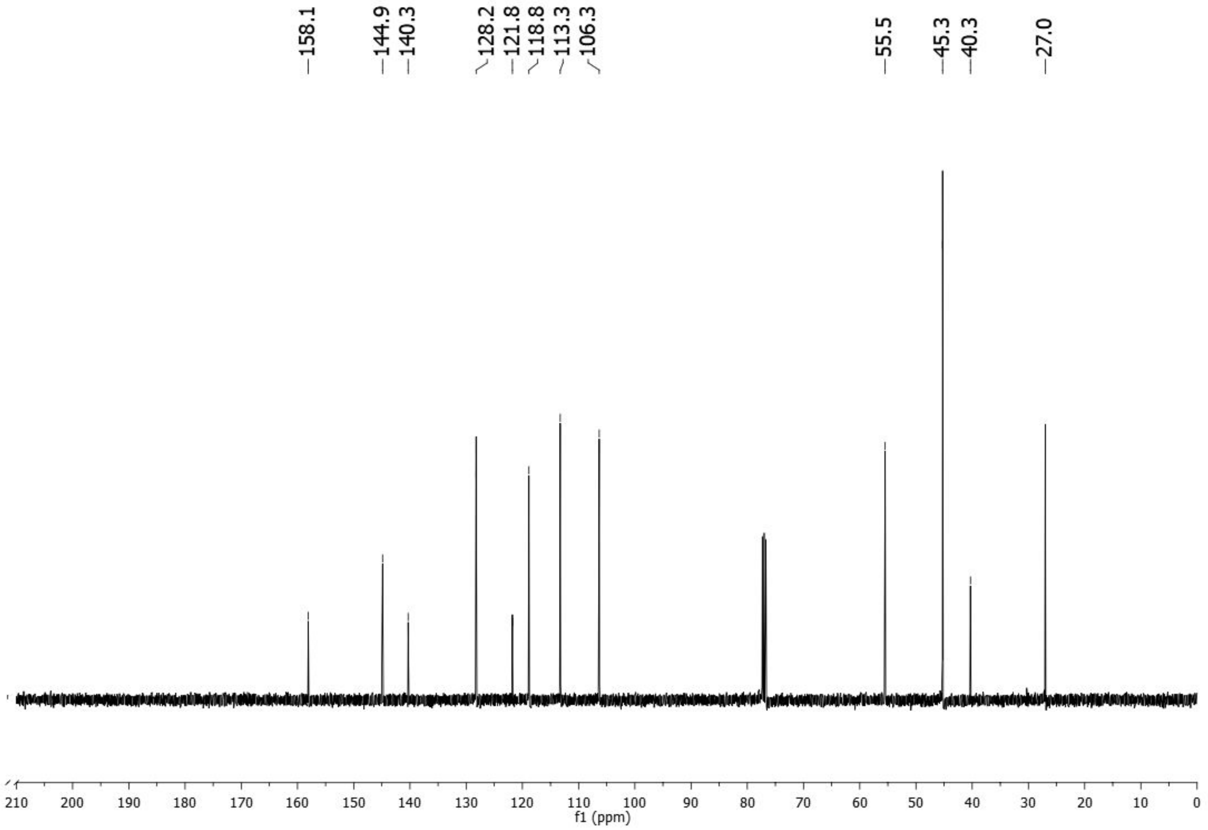

1e ^{1}HNMR (400 MHz, $CDCl_3$)

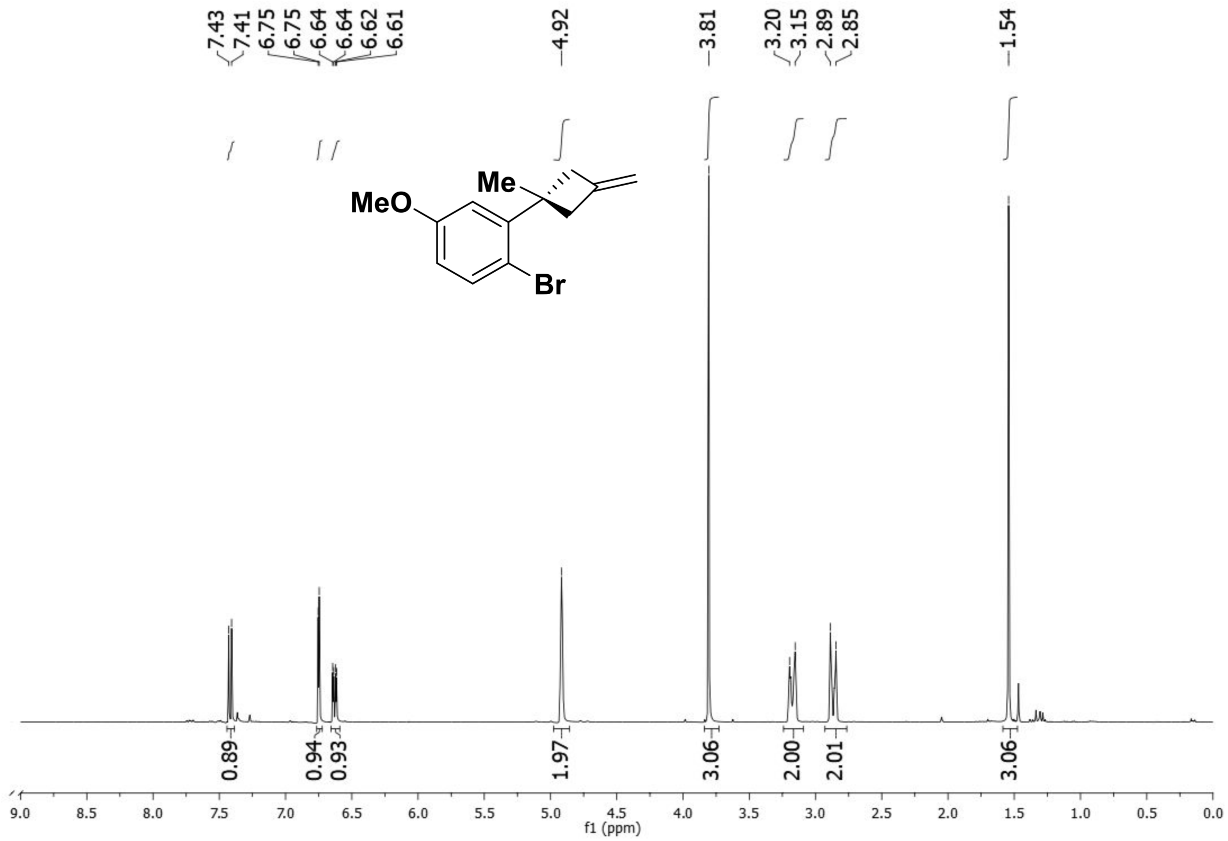

1e ^{13}CNMR (100 MHz, $CDCl_3$)

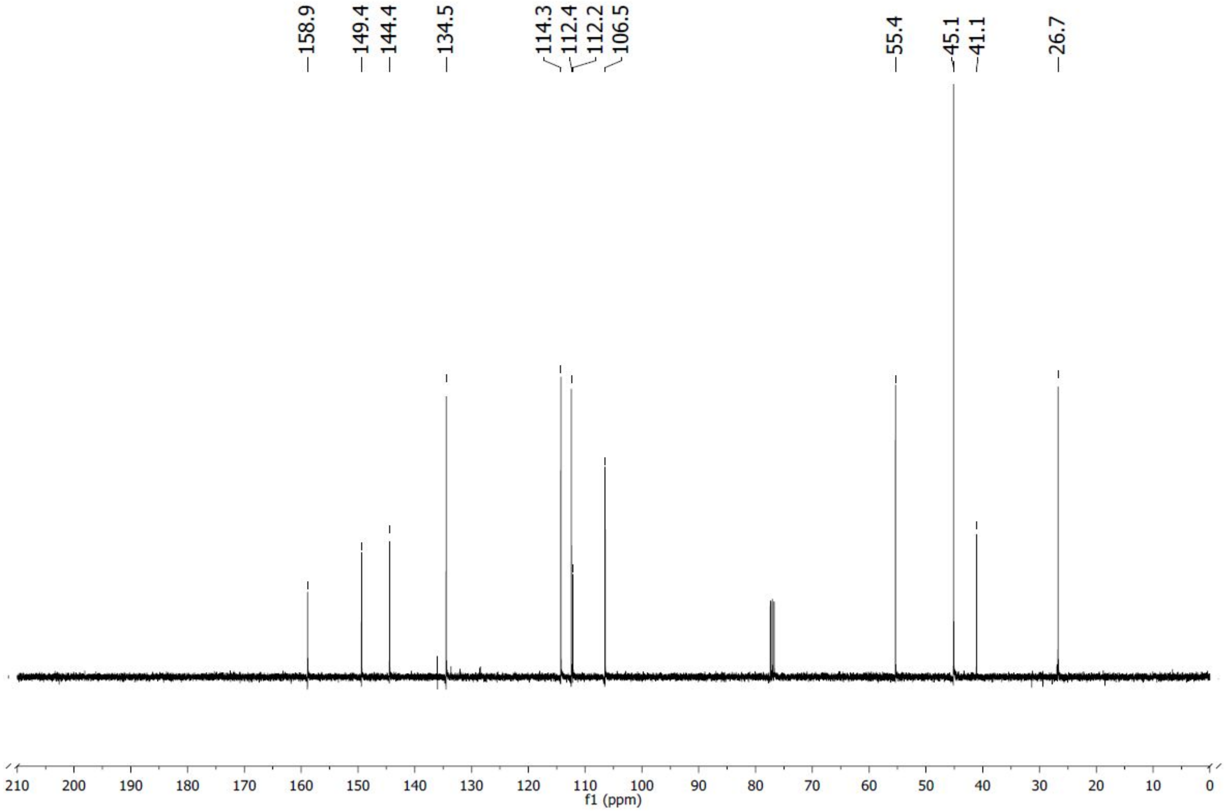

1f ^{1}HNMR (400 MHz, $CDCl_3$)

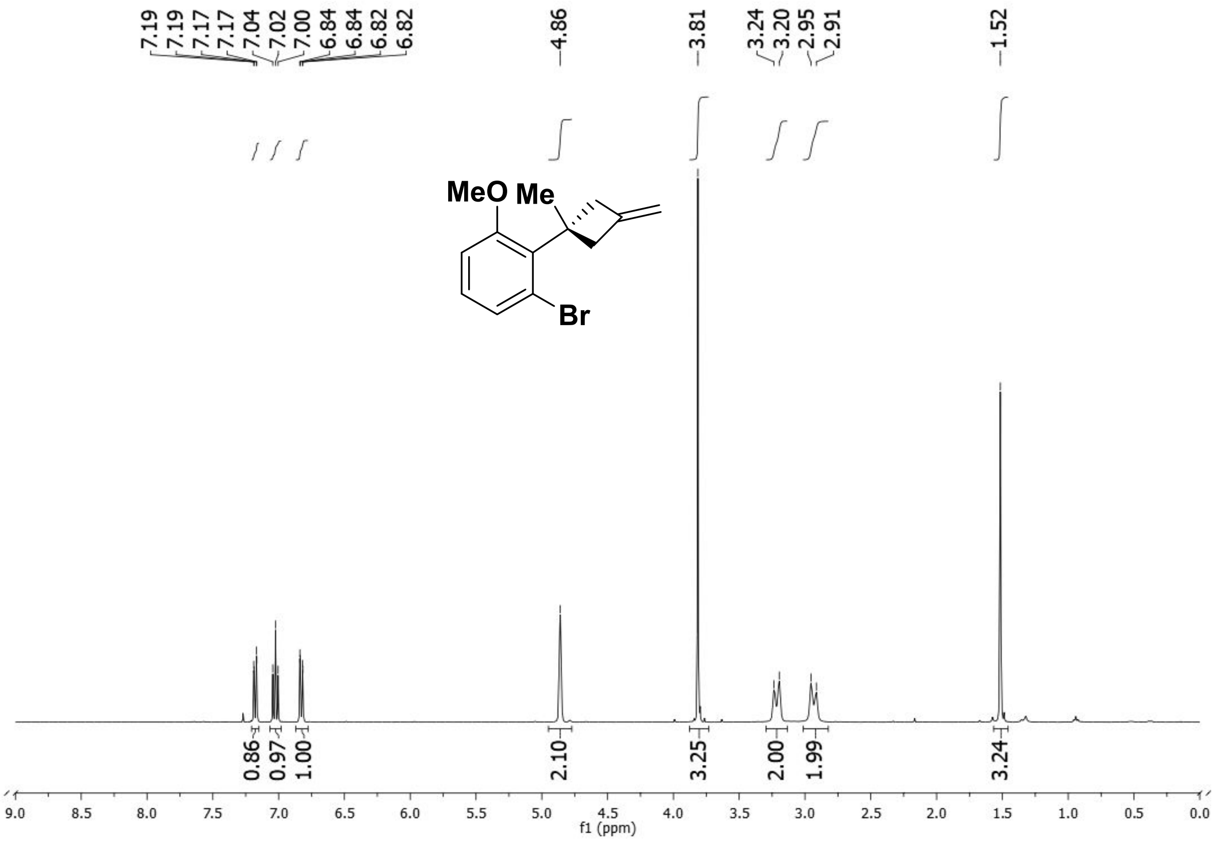

1f ^{13}CNMR (100 MHz, $CDCl_3$)

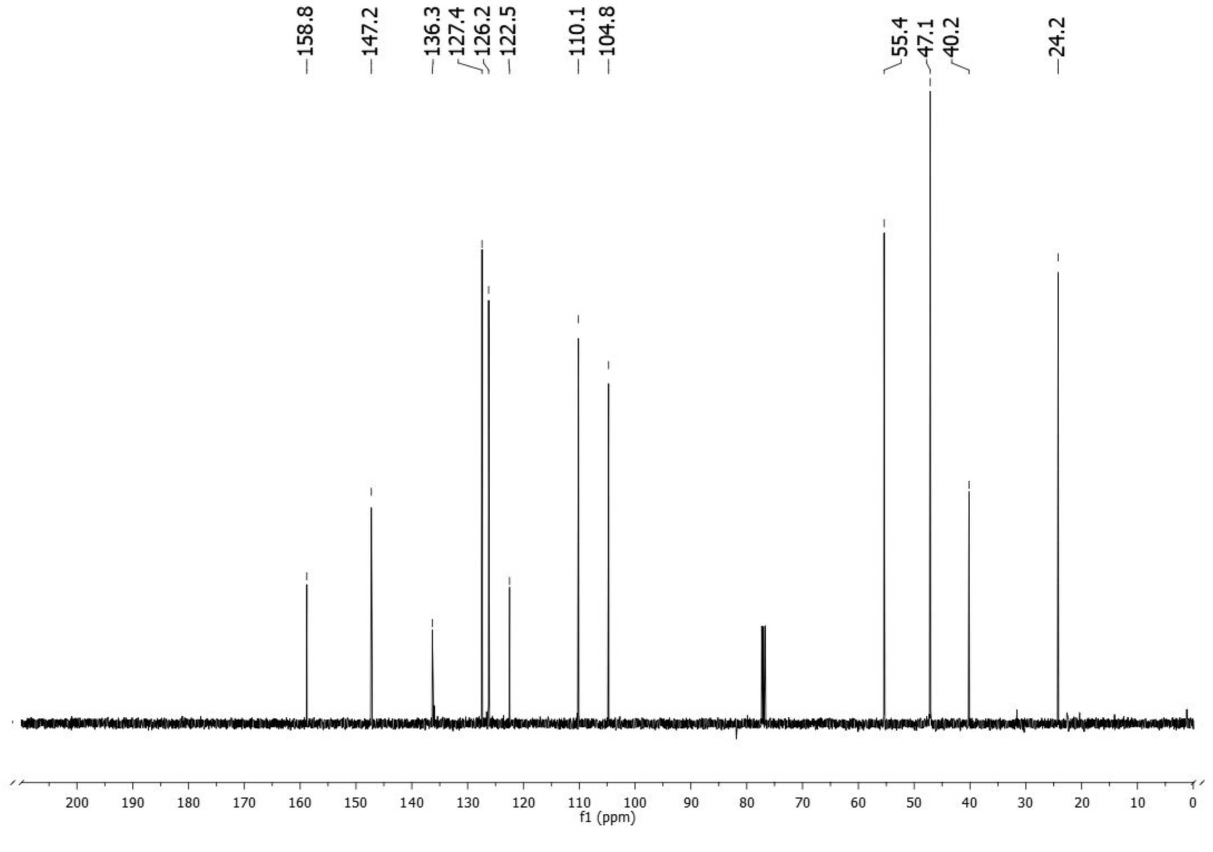

1g ^{1}HNMR (400 MHz, $CDCl_3$)

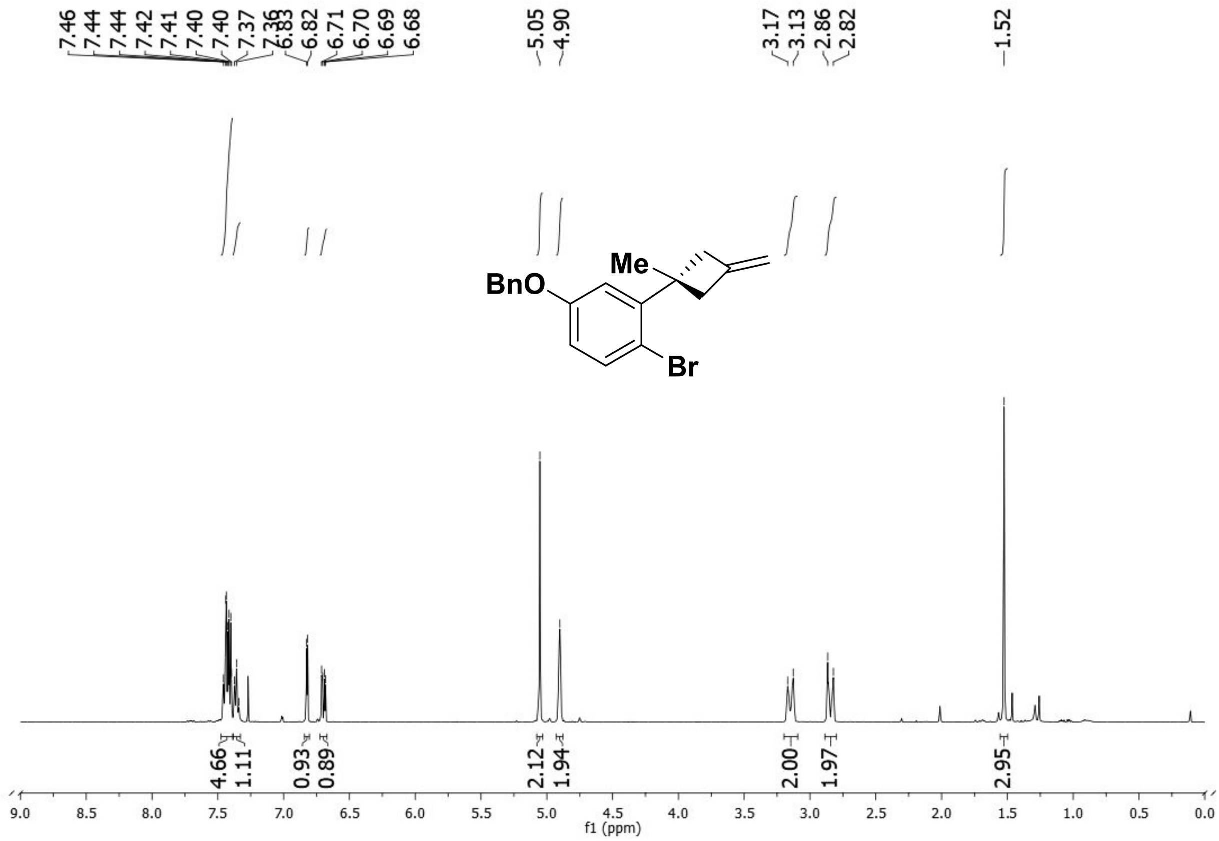

1g ^{13}CNMR (100 MHz, $CDCl_3$)

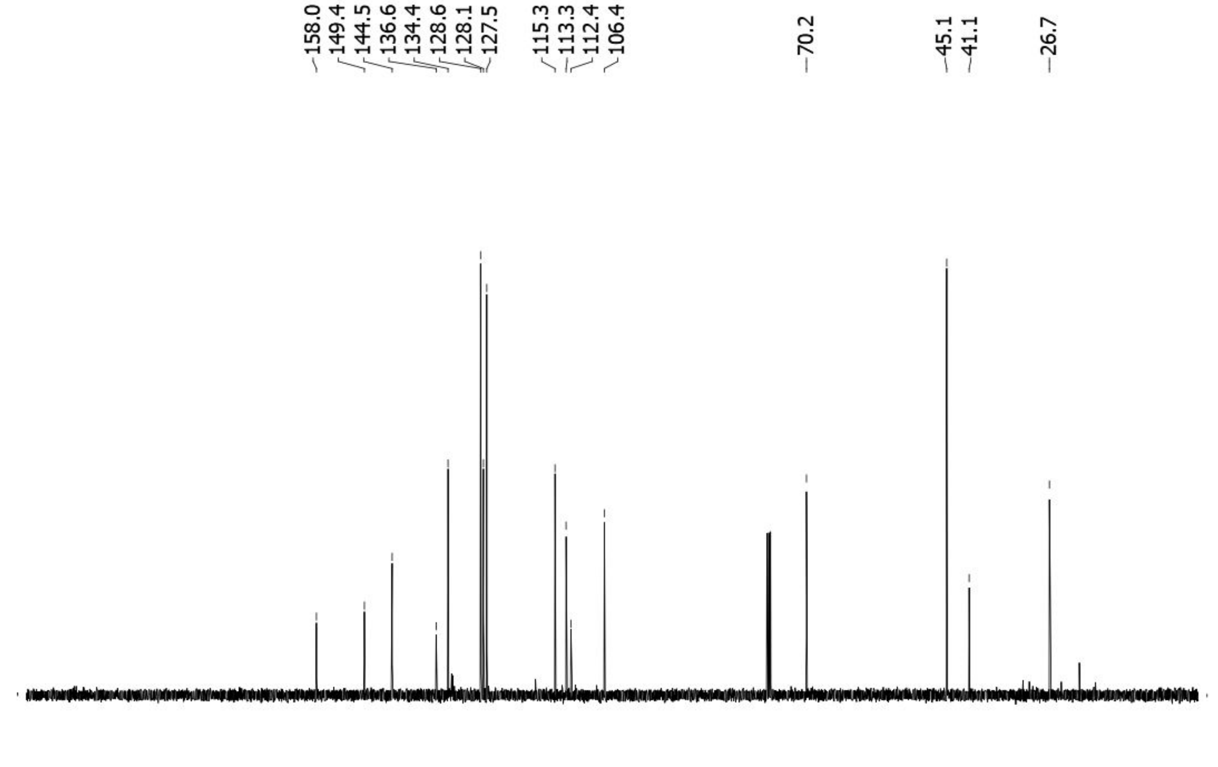

1h ^{1}HNMR (400 MHz, $CDCl_3$)

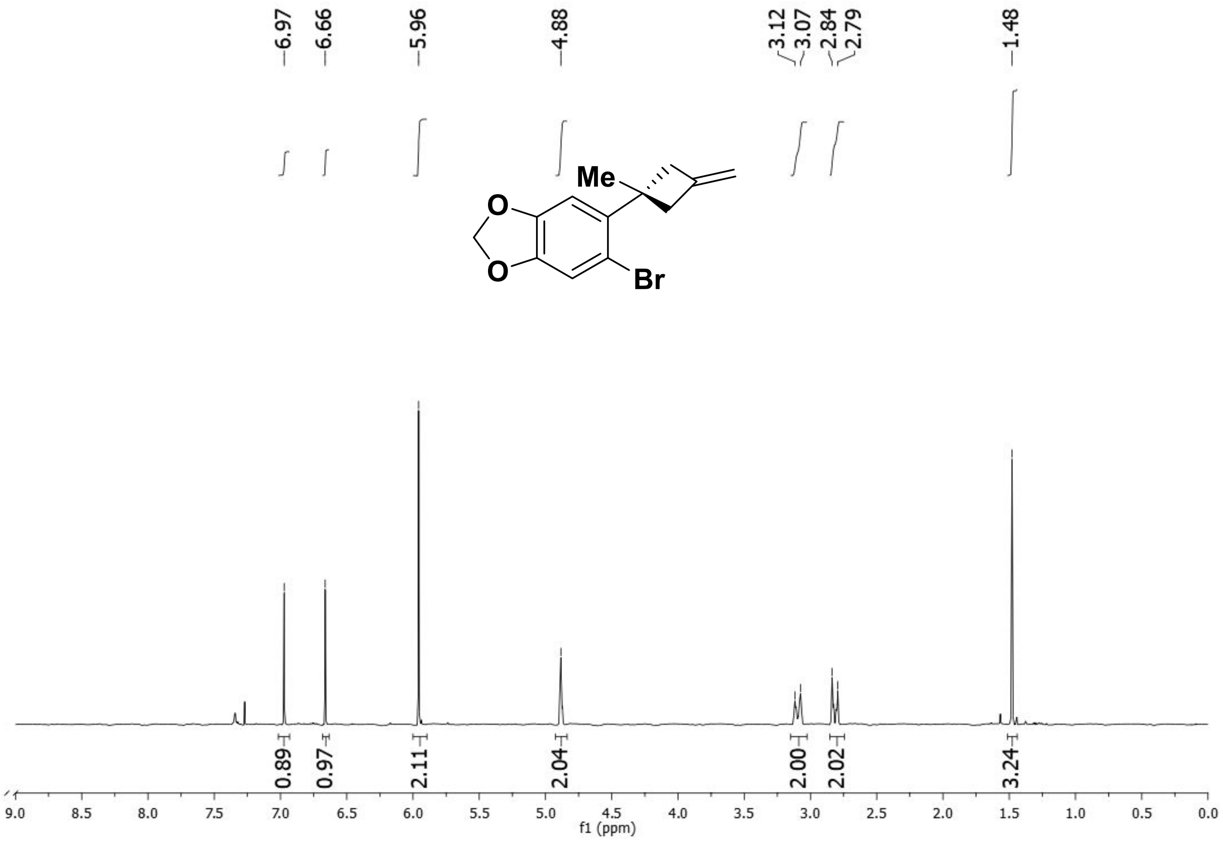

1h ^{13}CNMR (100 MHz, $CDCl_3$)

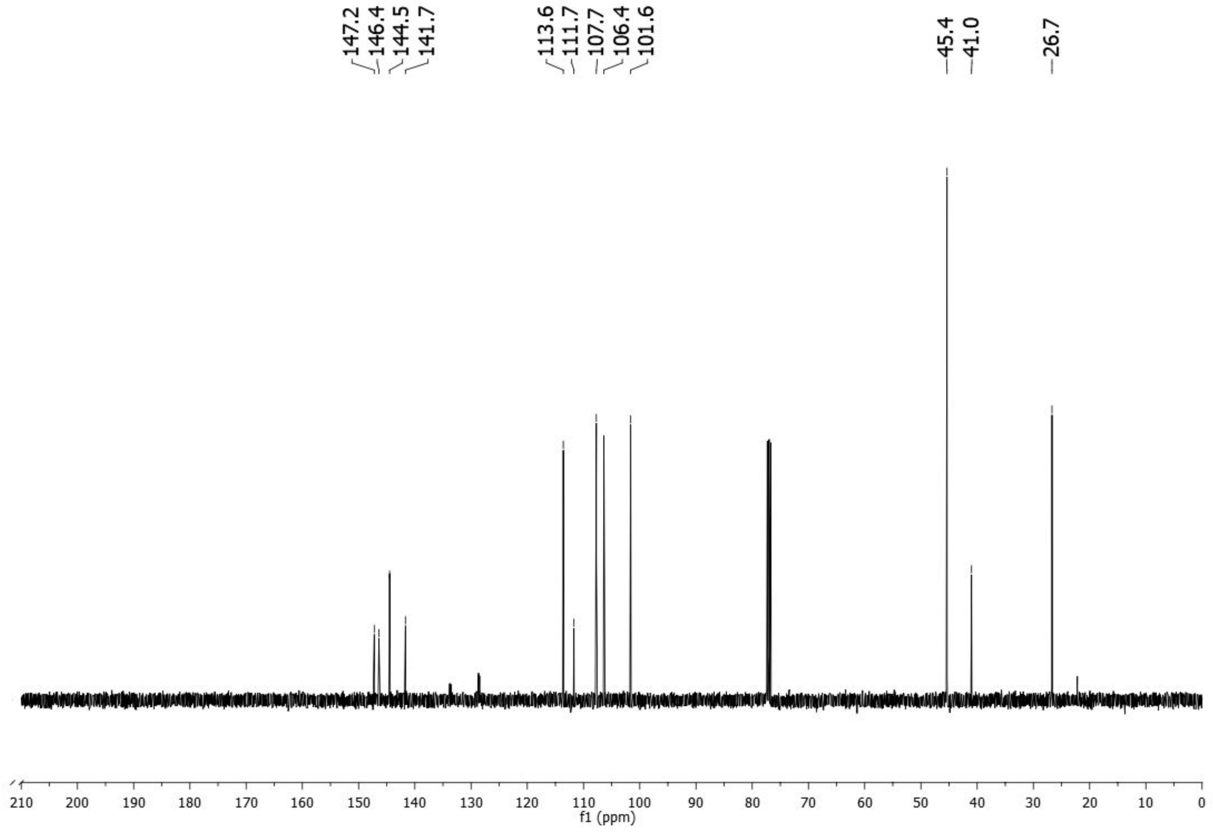

1i ^{1}HNMR (400 MHz, $CDCl_3$)

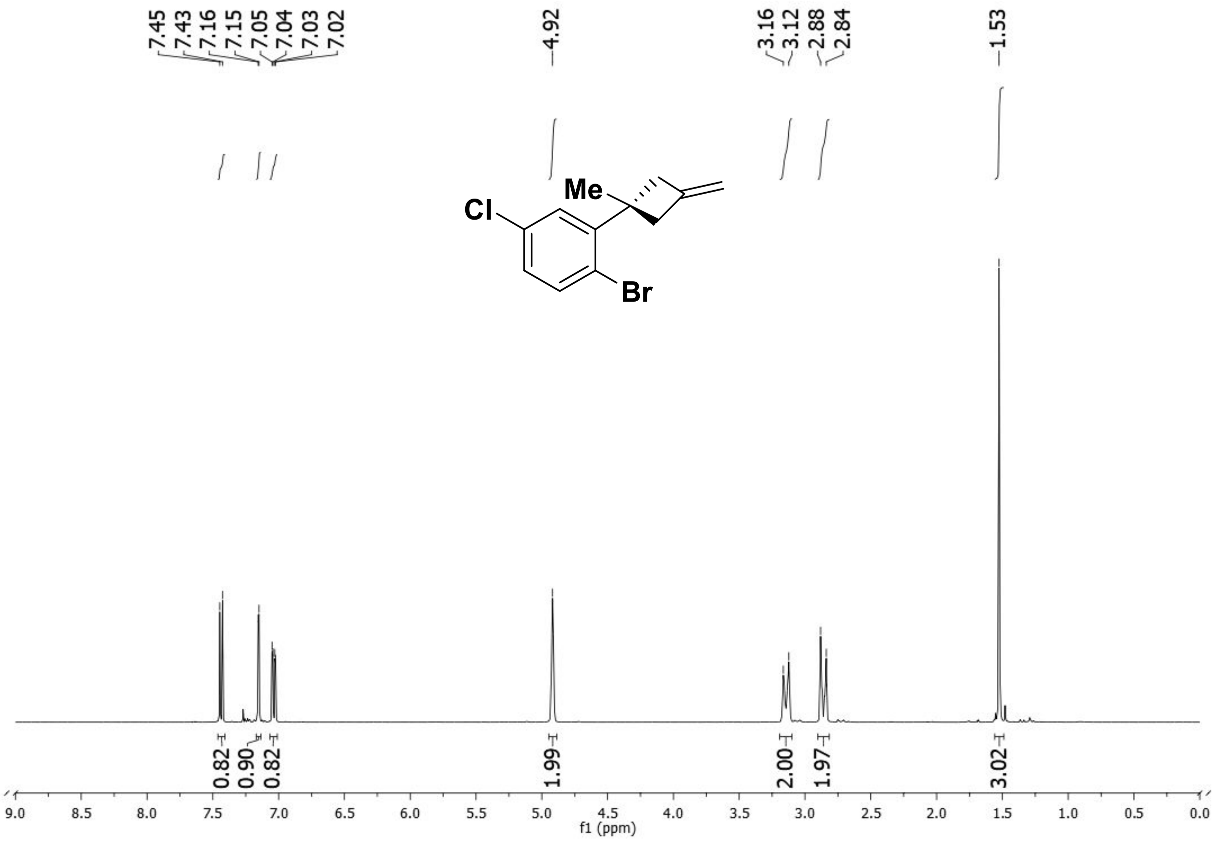

1i ^{13}CNMR (100 MHz, $CDCl_3$)

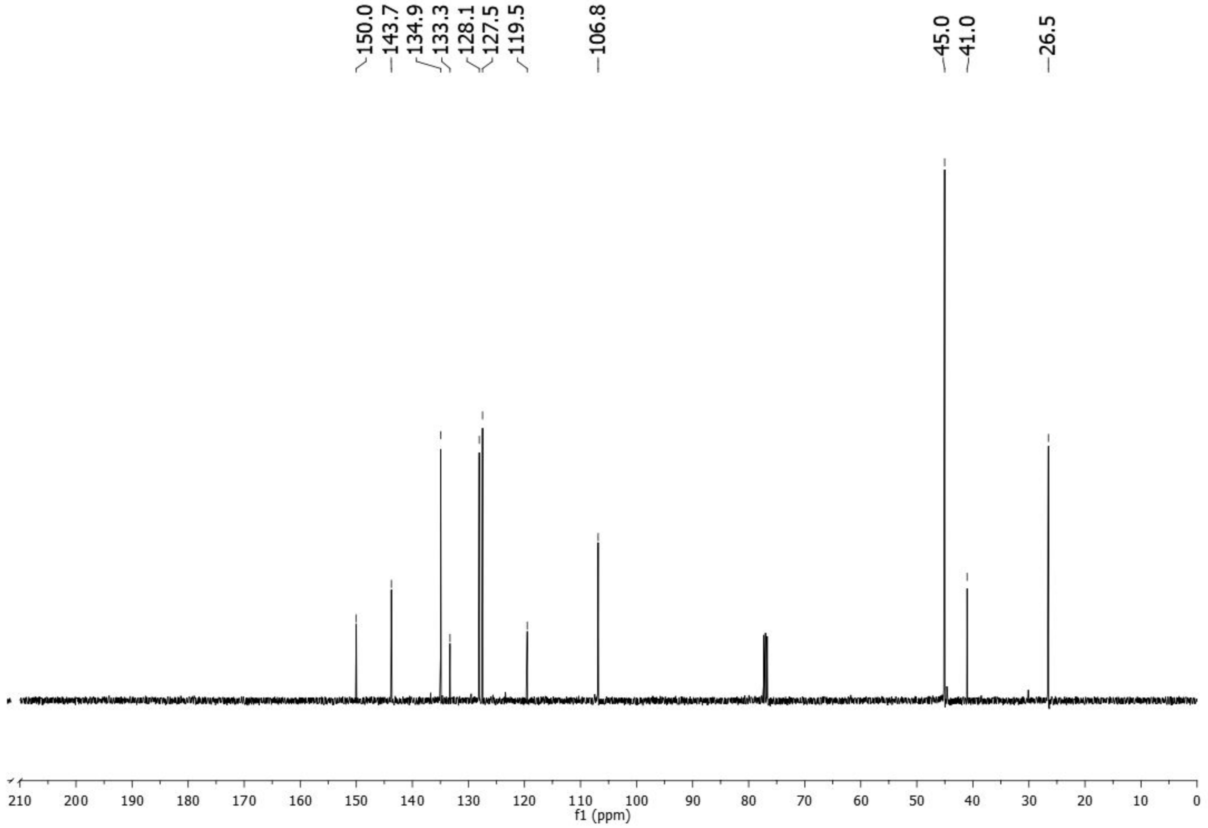

1j ¹HNMR (400 MHz, CDCl$_3$)

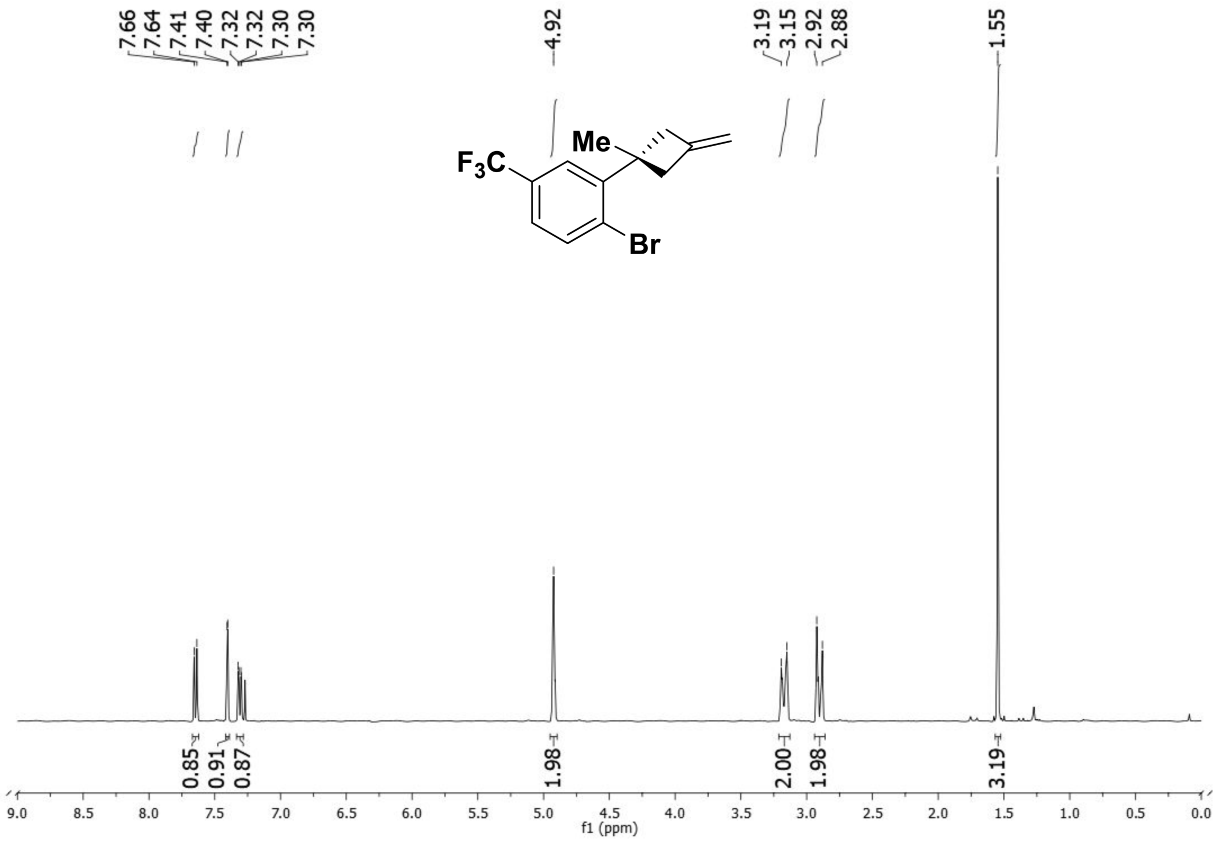

1j ¹³CNMR (100 MHz, CDCl$_3$)

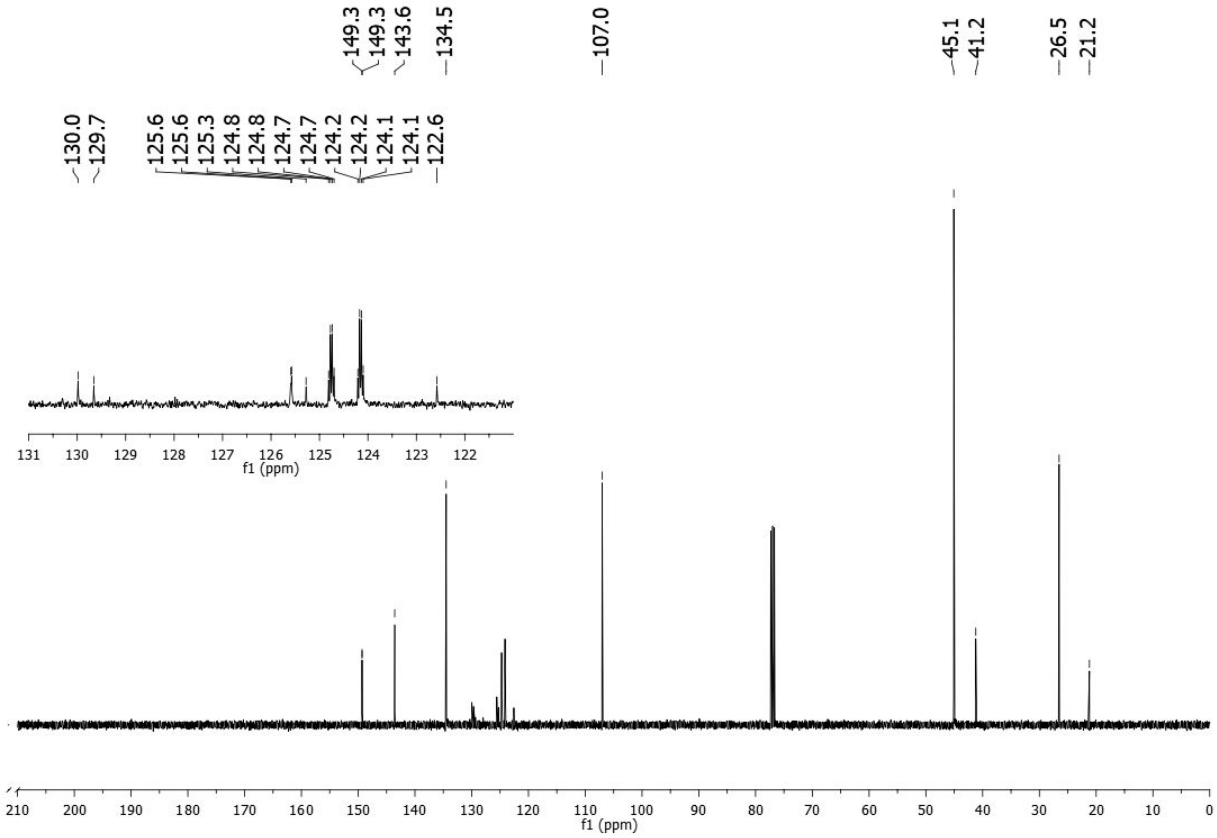

1j ^{19}FNMR (377 MHz, $CDCl_3$)

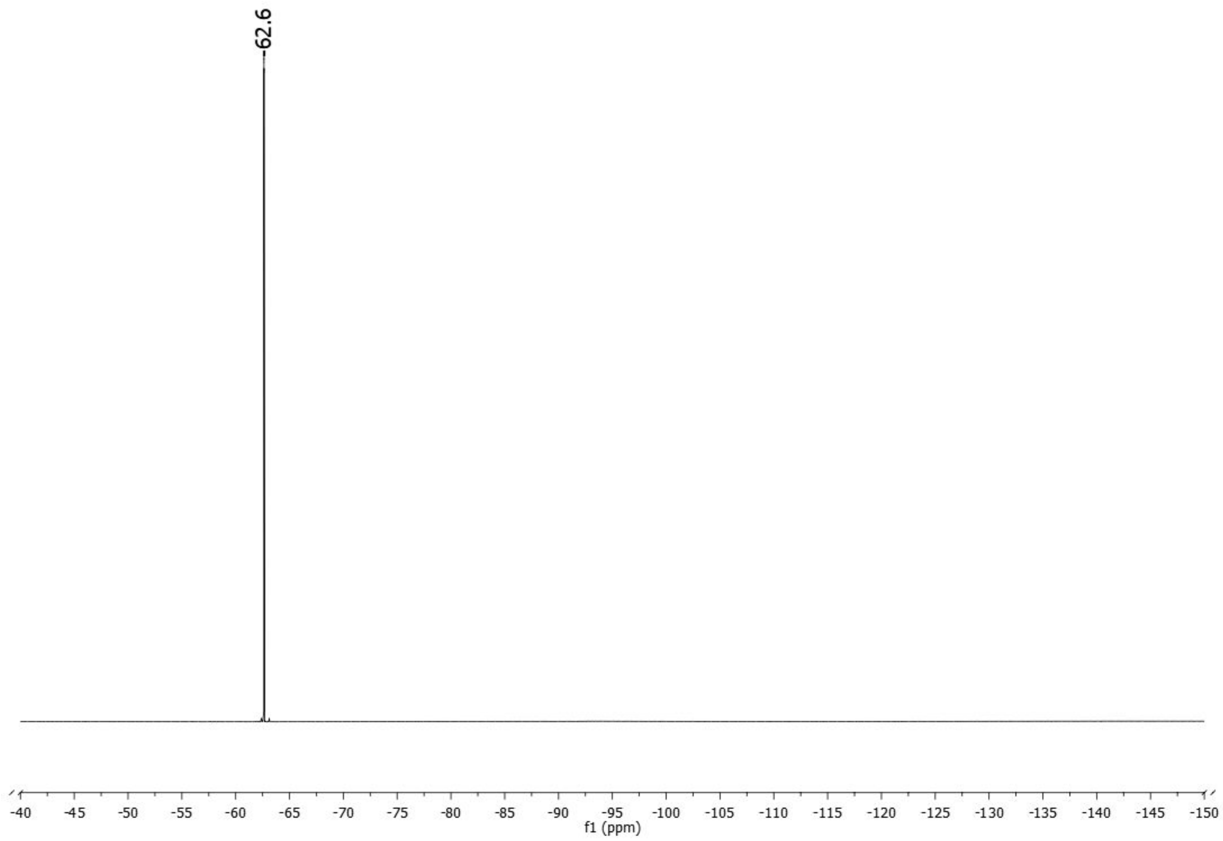

1k ^{1}HNMR (400 MHz, $CDCl_3$)

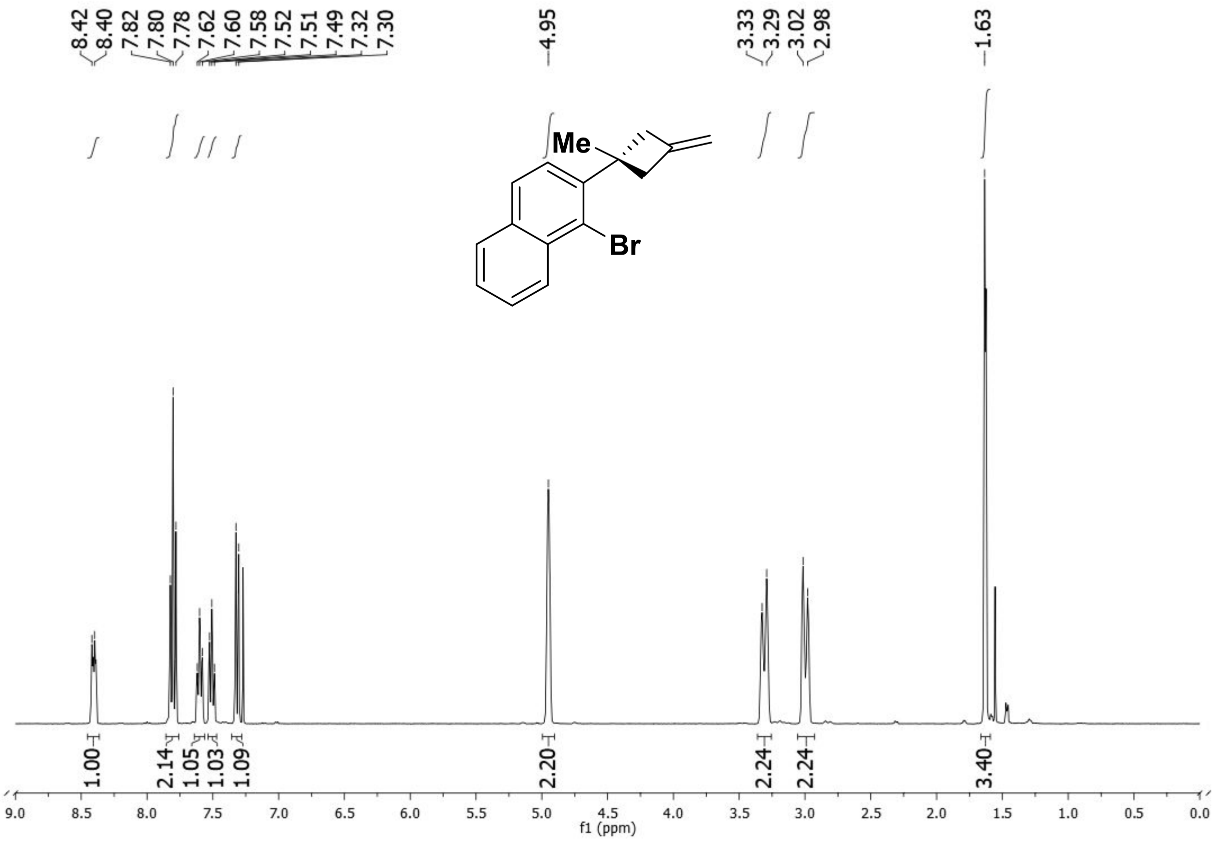

1k ^{13}CNMR (100 MHz, $CDCl_3$)

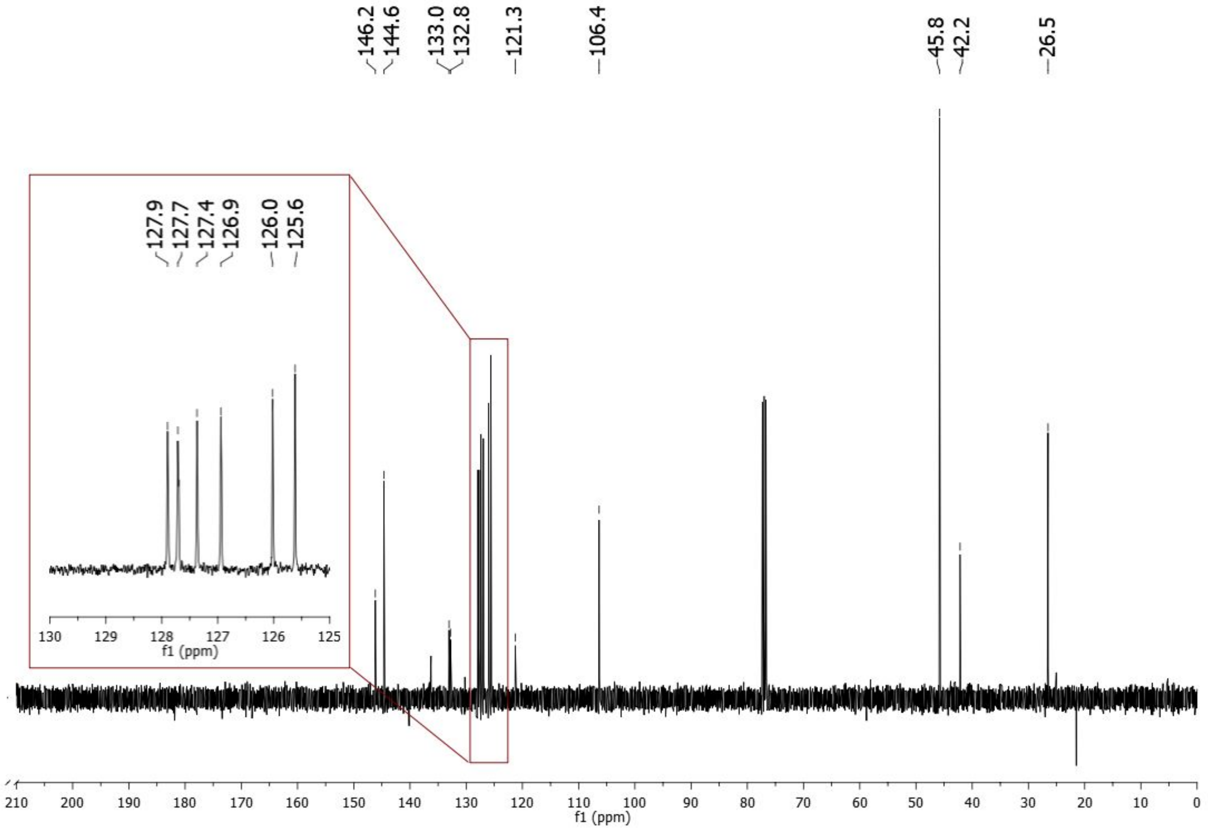

1l ^{1}HNMR (400 MHz, $CDCl_3$)

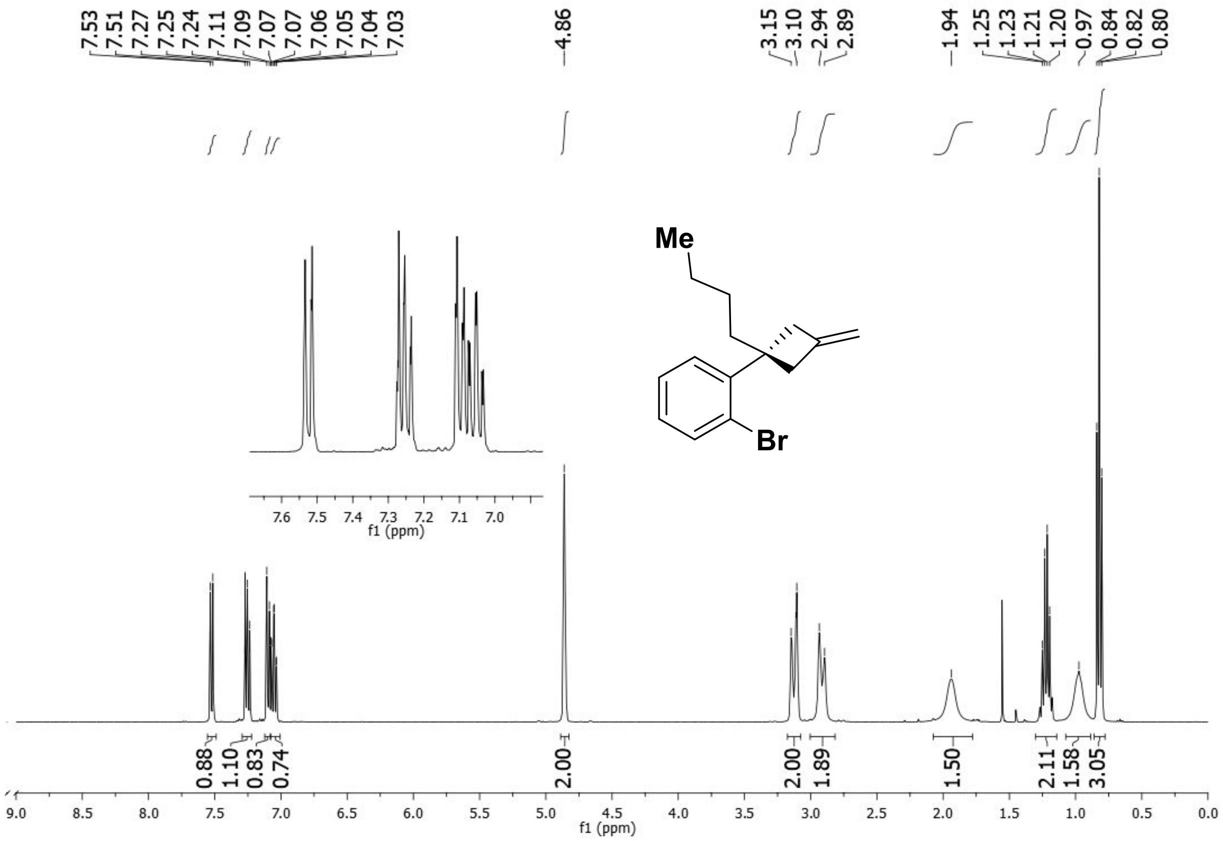

1l ^{13}CNMR (100 MHz, $CDCl_3$)

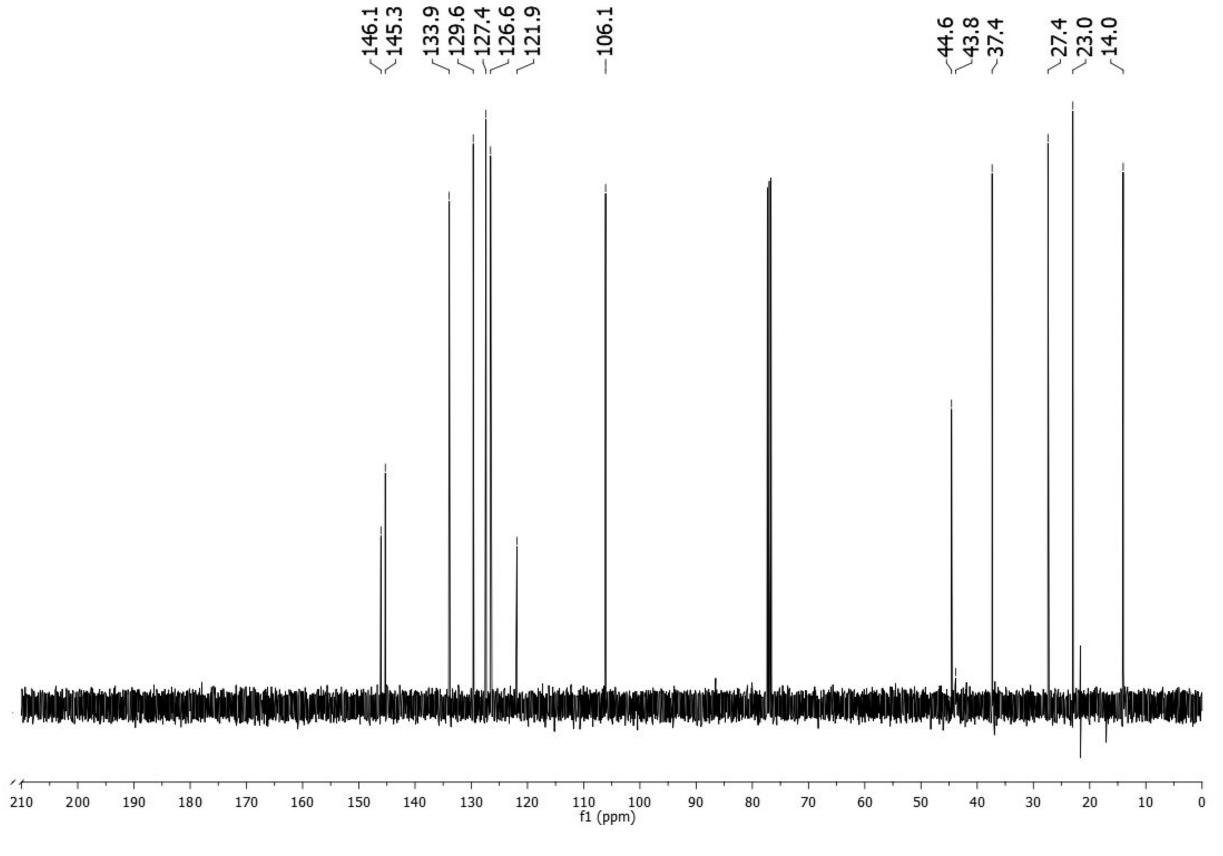

1m ^{1}HNMR (400 MHz, $CDCl_3$)

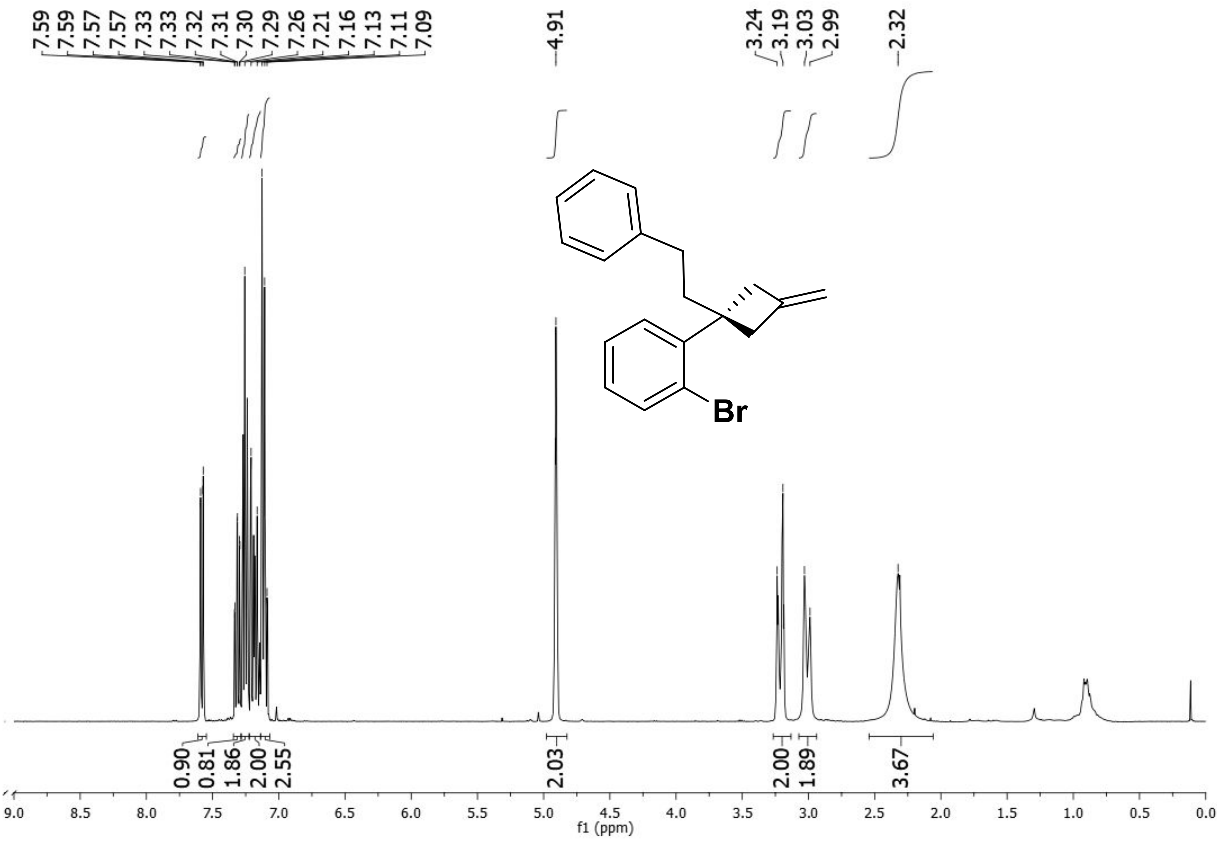

1m ^{13}CNMR (100 MHz, $CDCl_3$)

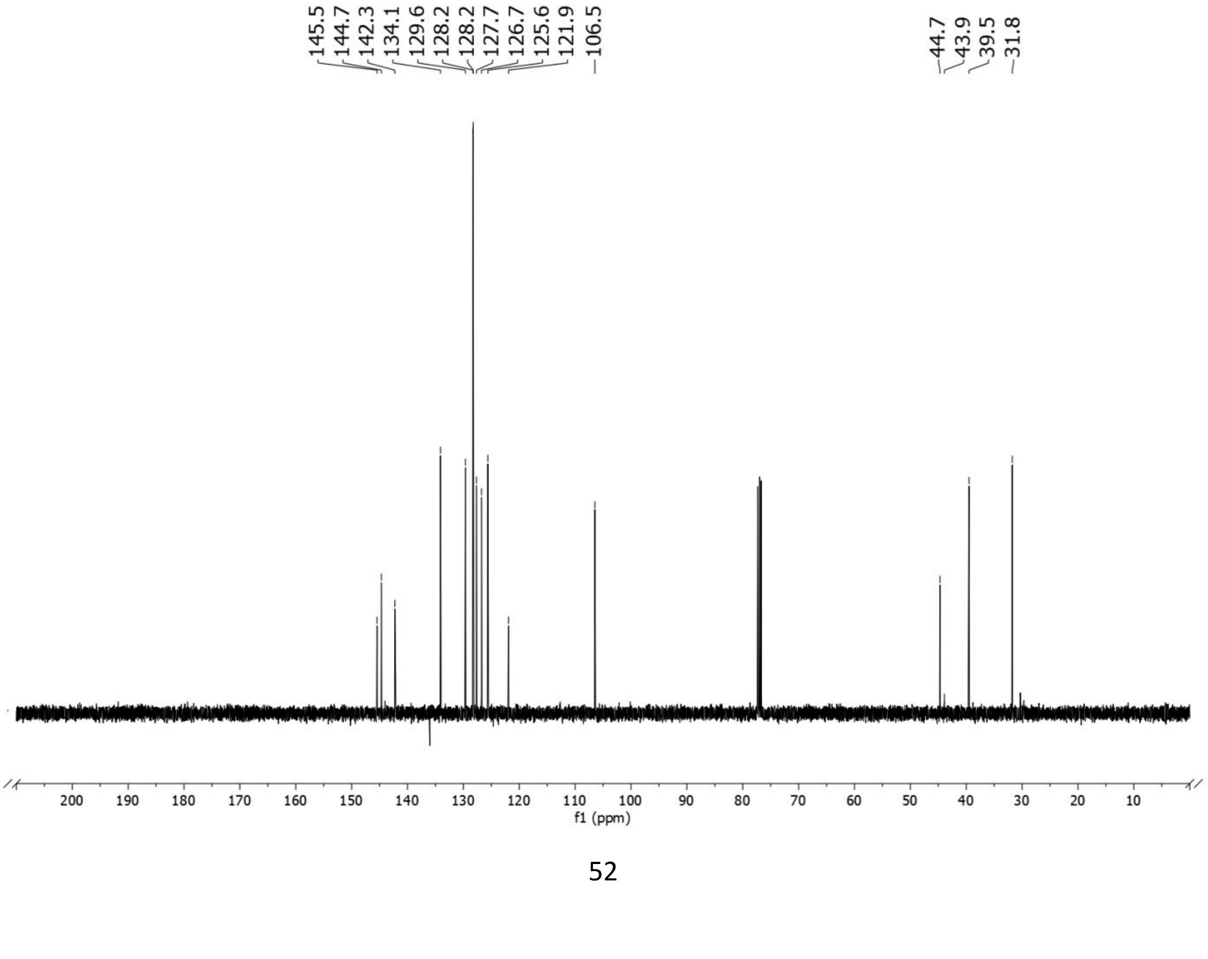

1n ^{1}HNMR (400 MHz, $CDCl_3$)

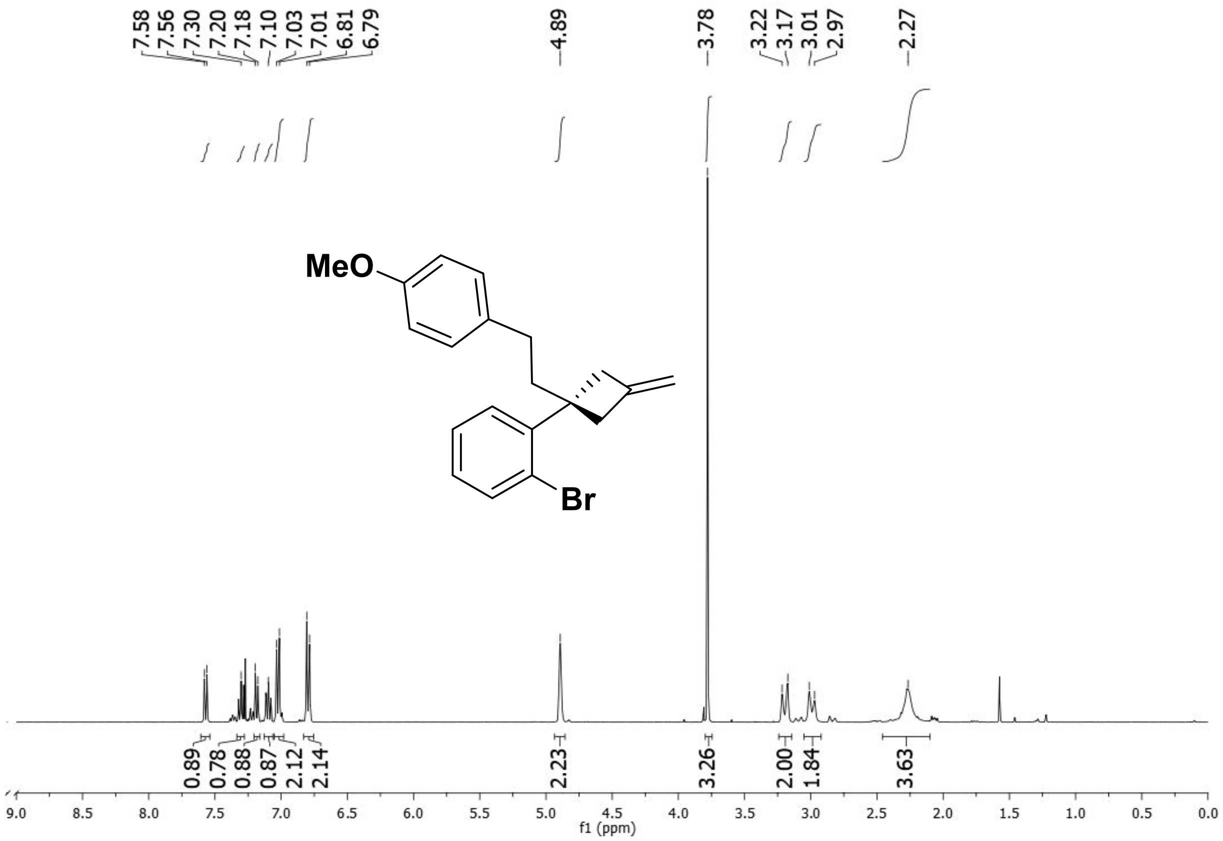

1n ^{13}CNMR (100 MHz, $CDCl_3$)

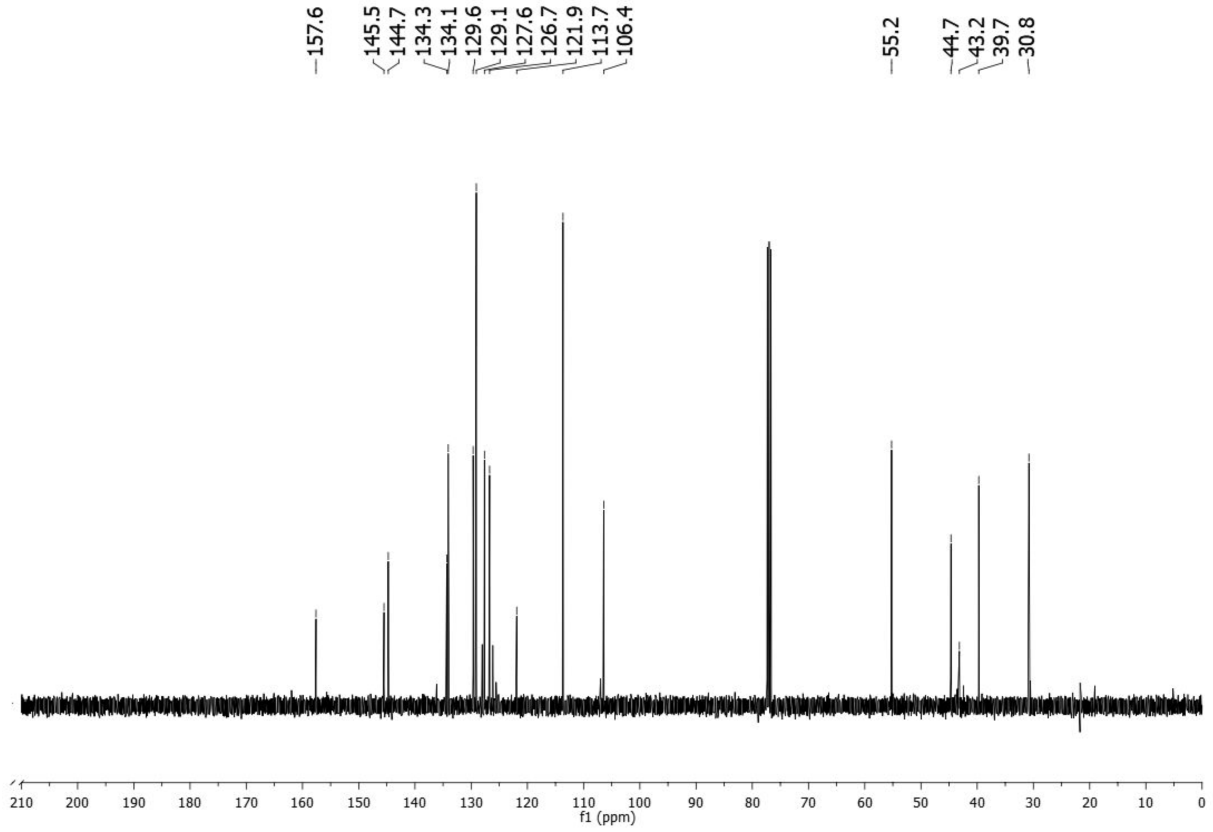

1o ^{1}HNMR (400 MHz, $CDCl_3$)

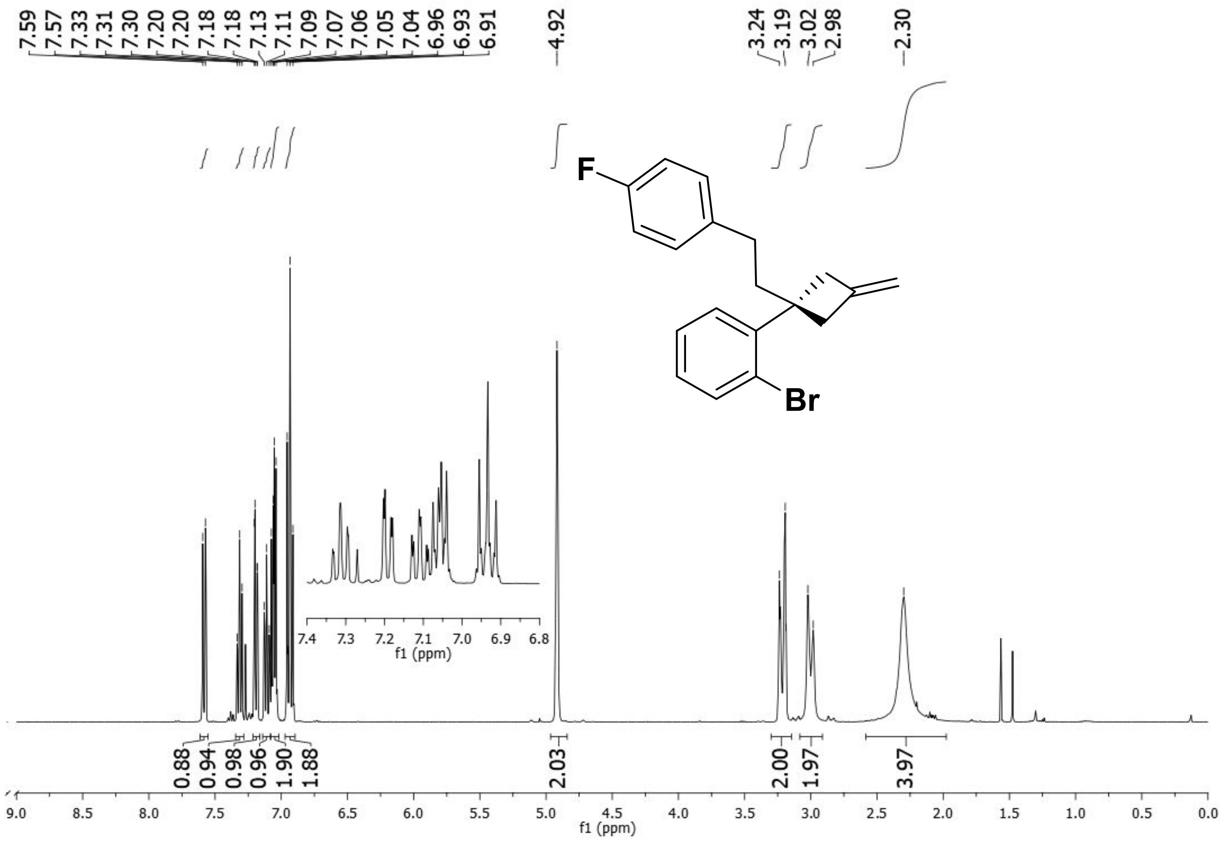

1o ^{13}CNMR (100 MHz, $CDCl_3$)

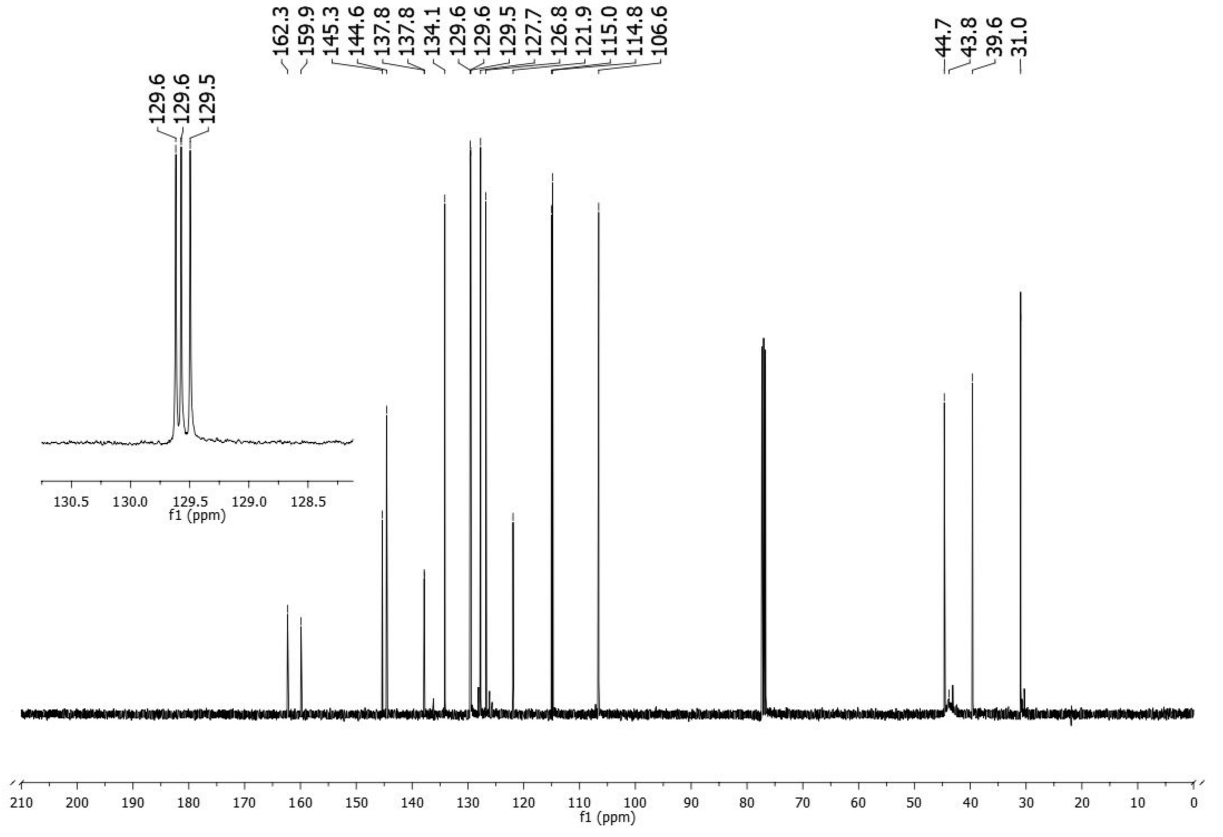

1o ^{19}FNMR (377 MHz, CDCl$_3$)

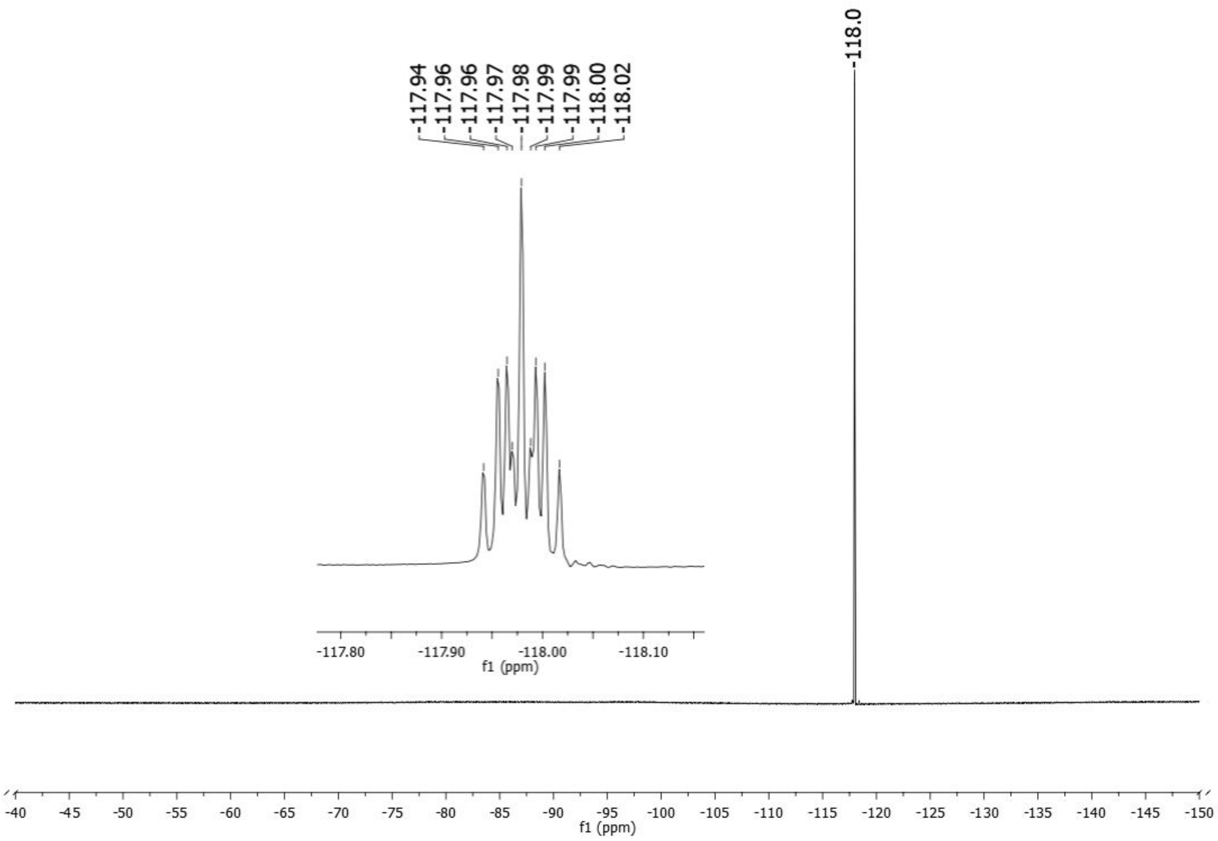

1p ^{1}HNMR (400 MHz, $CDCl_3$)

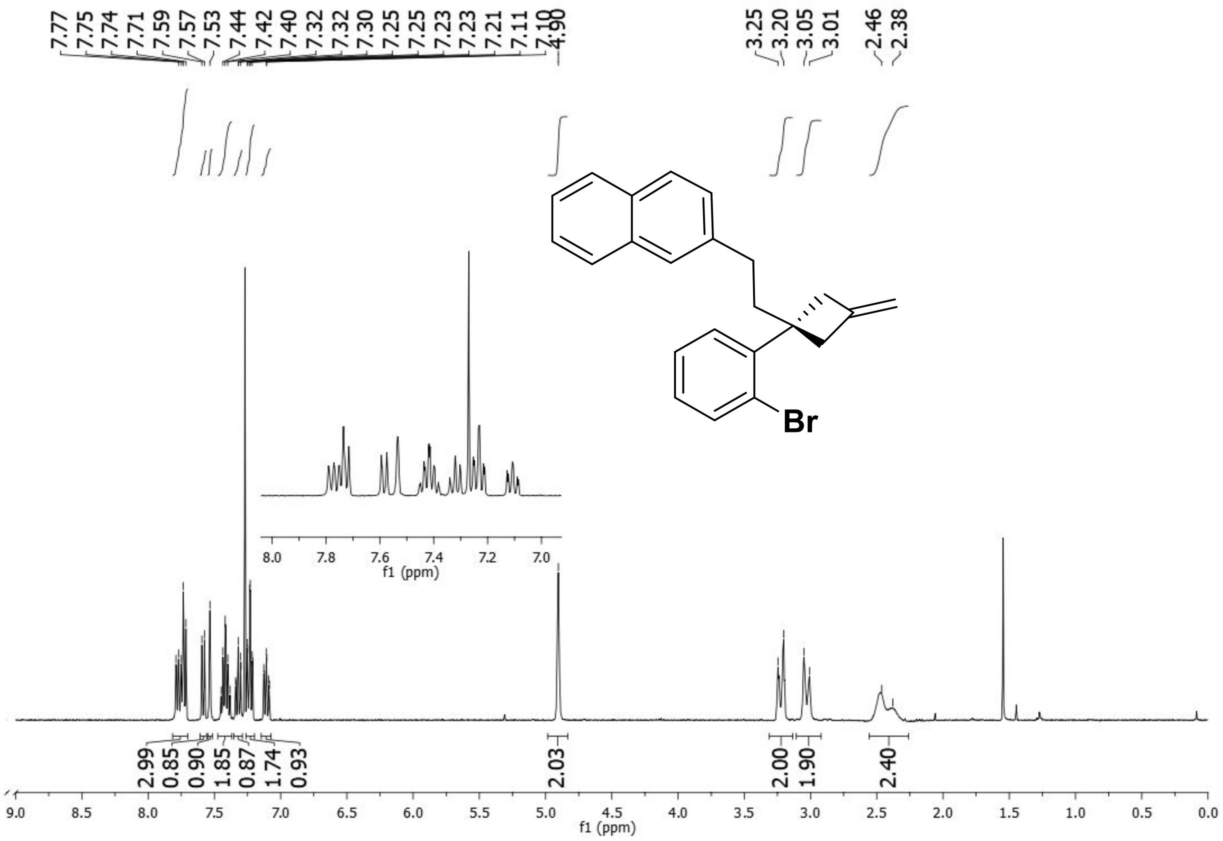

1p ^{13}C NMR (100 MHz, $CDCl_3$)

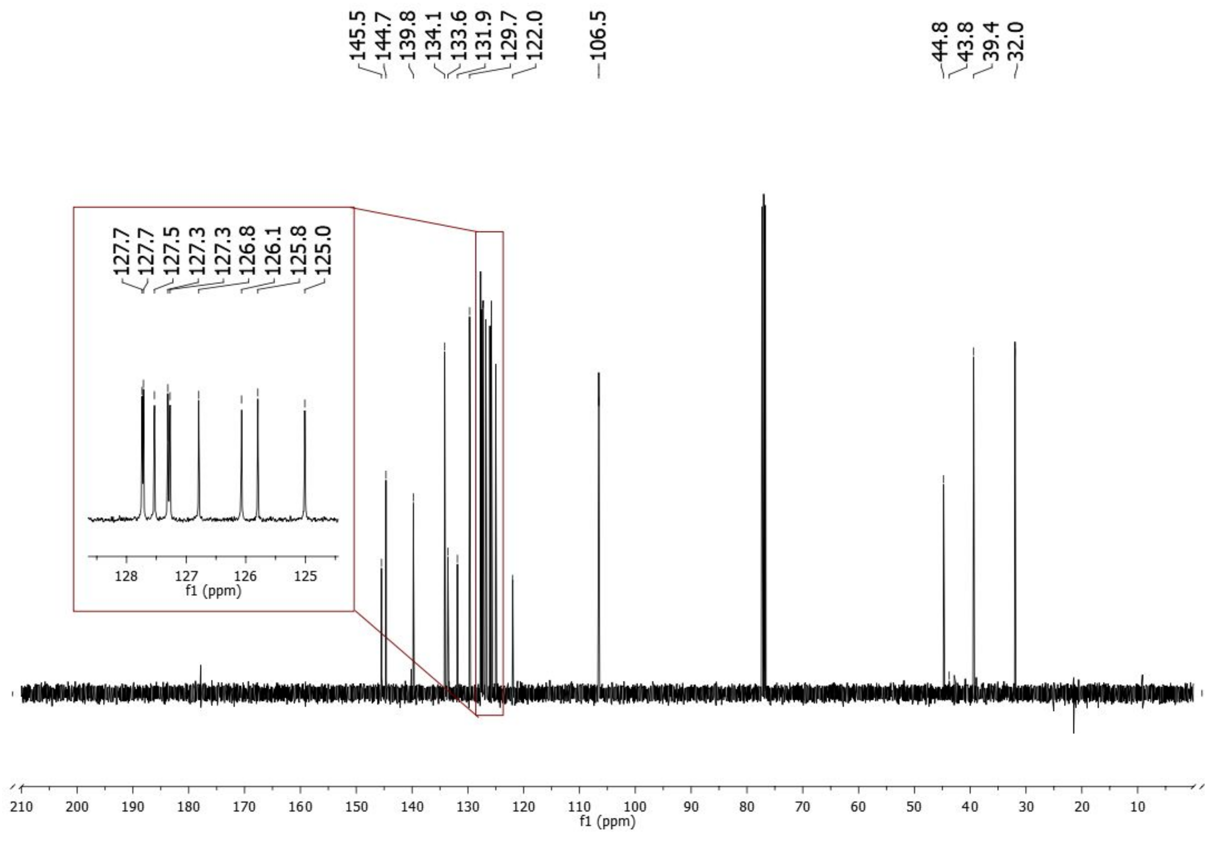

1q ¹HNMR (400 MHz, $CDCl_3$)

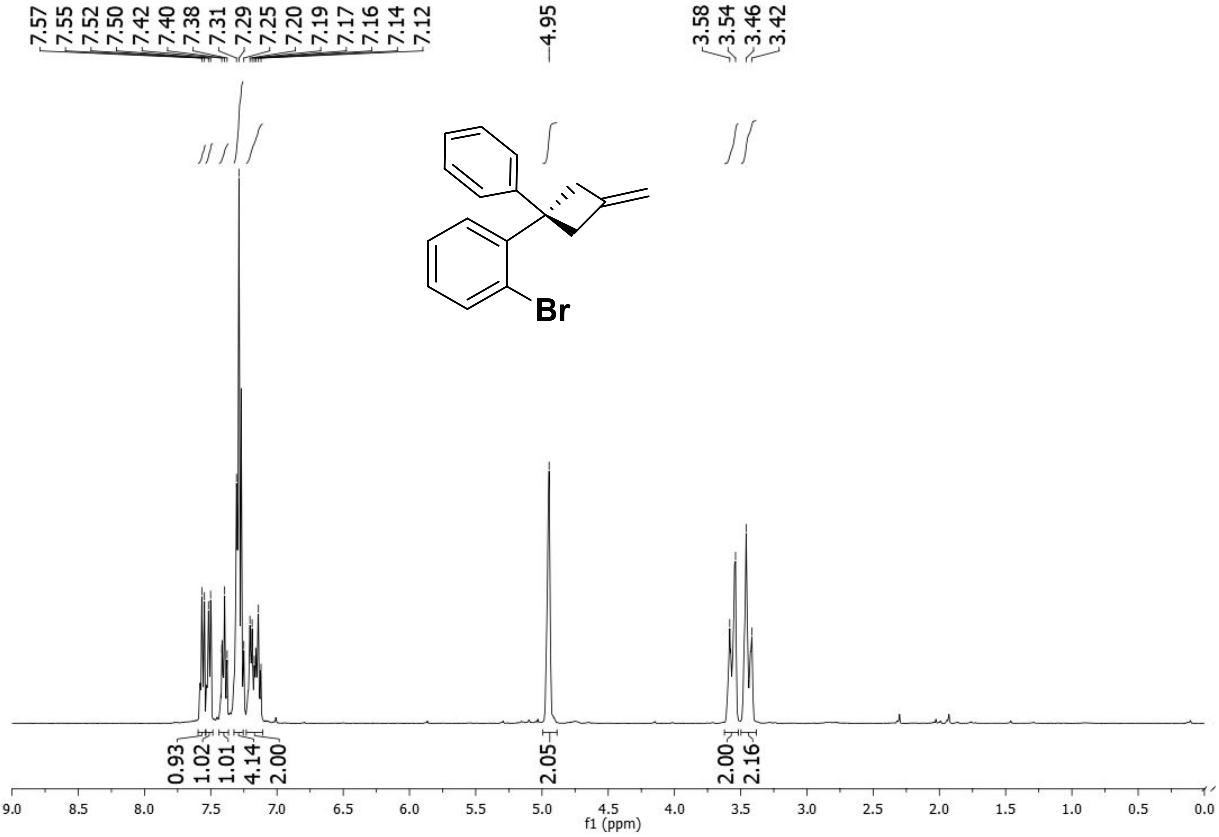

1q ¹³CNMR (100 MHz, $CDCl_3$)

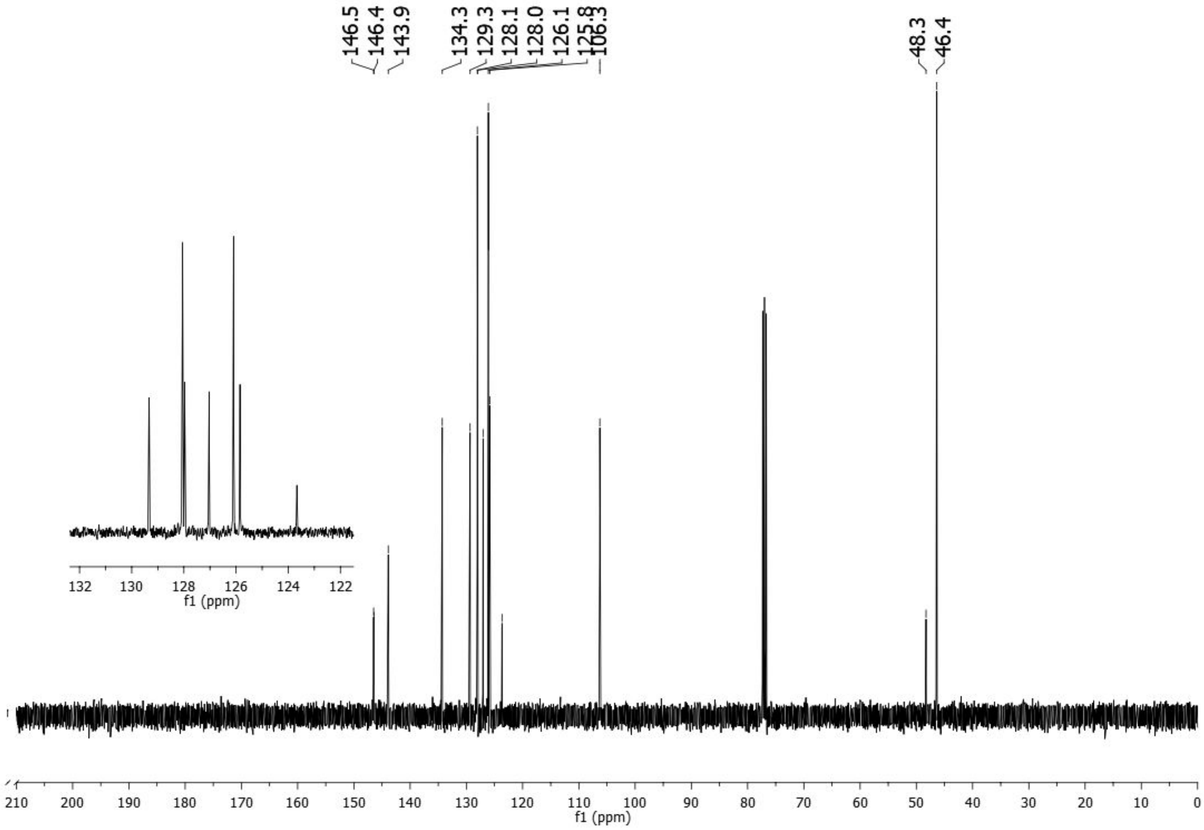

1r ^{1}HNMR (400 MHz, $CDCl_3$)

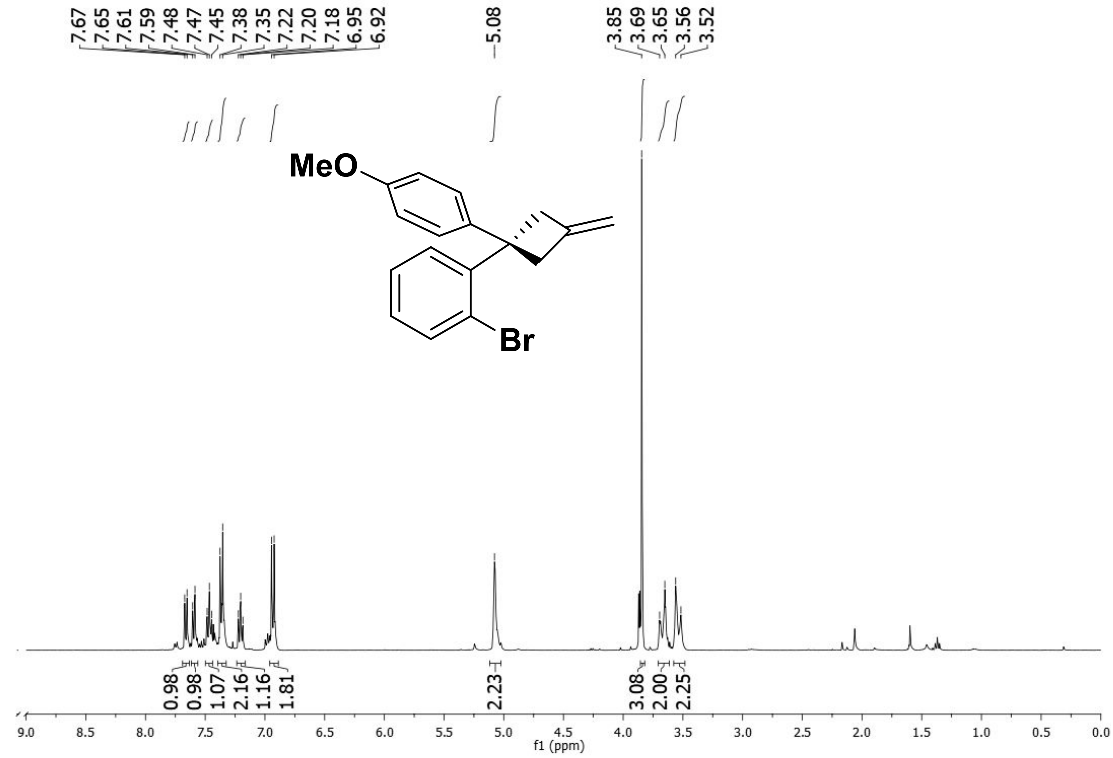

1r ^{13}CNMR (100 MHz, $CDCl_3$)[23]

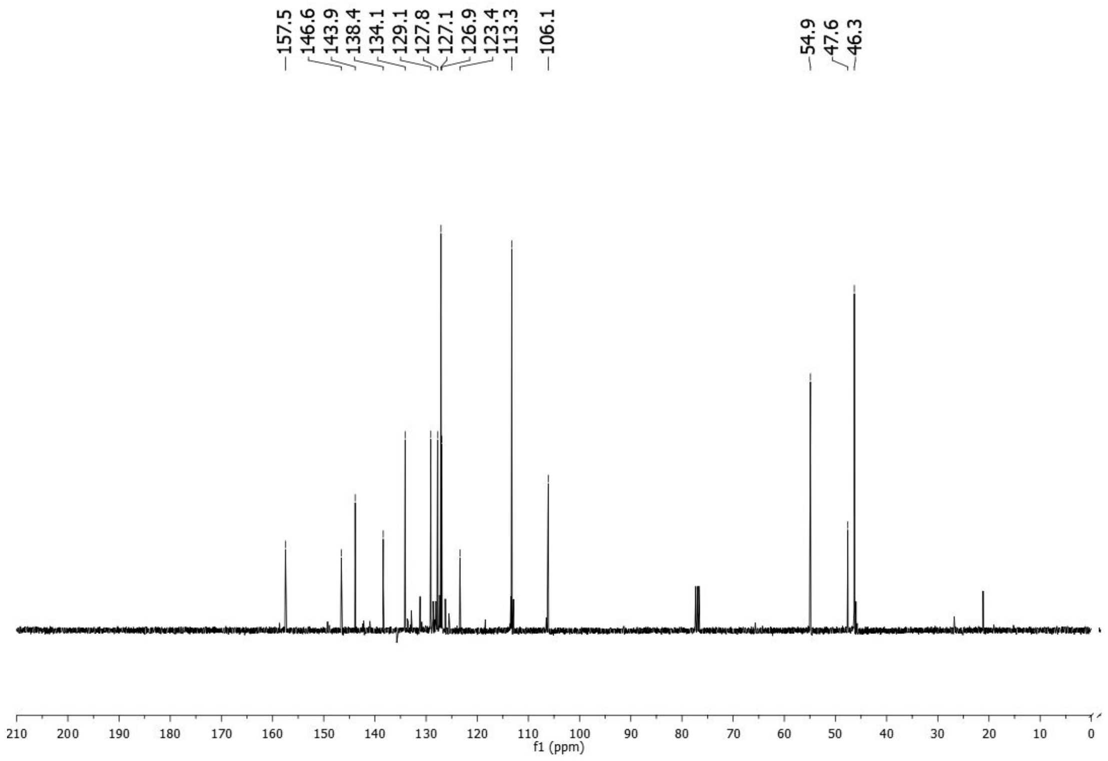

[23] An amount of about 12% of monochlorinated alkylidene-cyclobutane is present (unseparable residue from the preparation).

2a ¹HNMR (400 MHz, $CDCl_3$)[24]

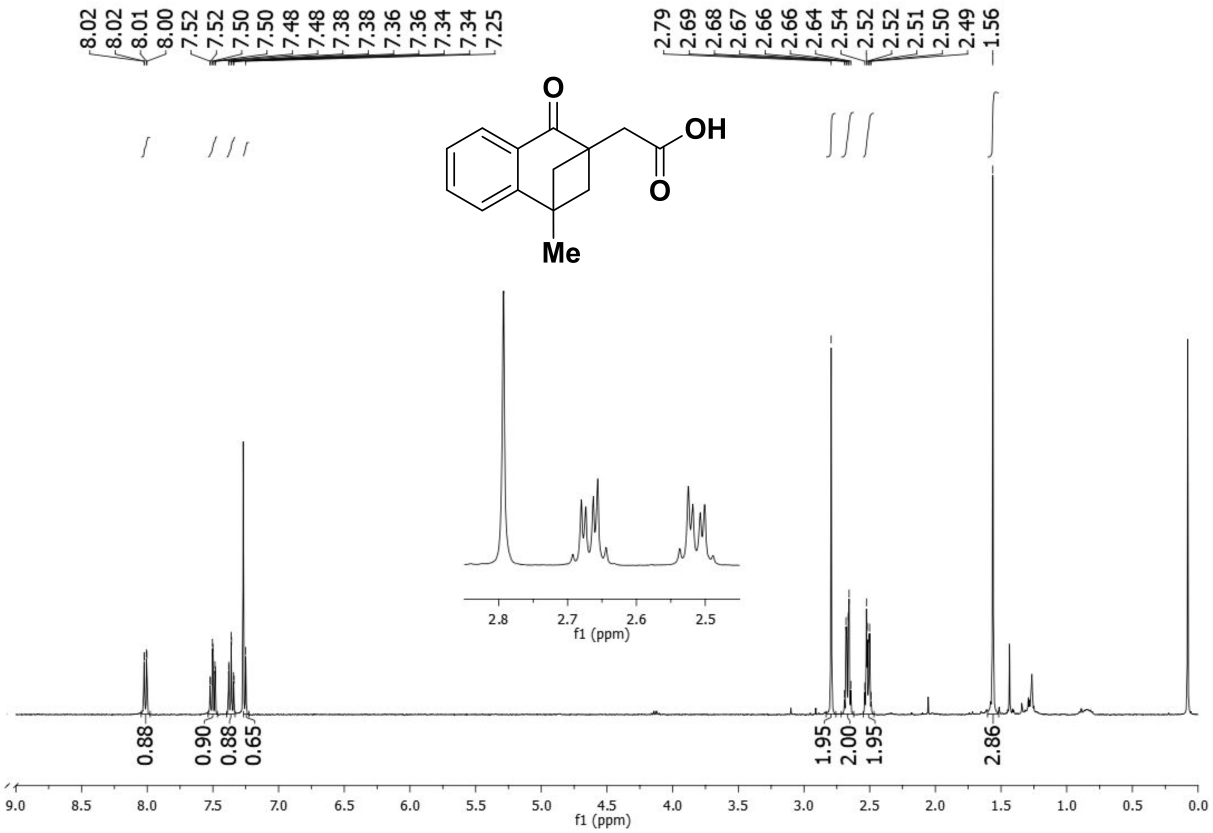

2a ¹³CNMR (100 MHz, $CDCl_3$)

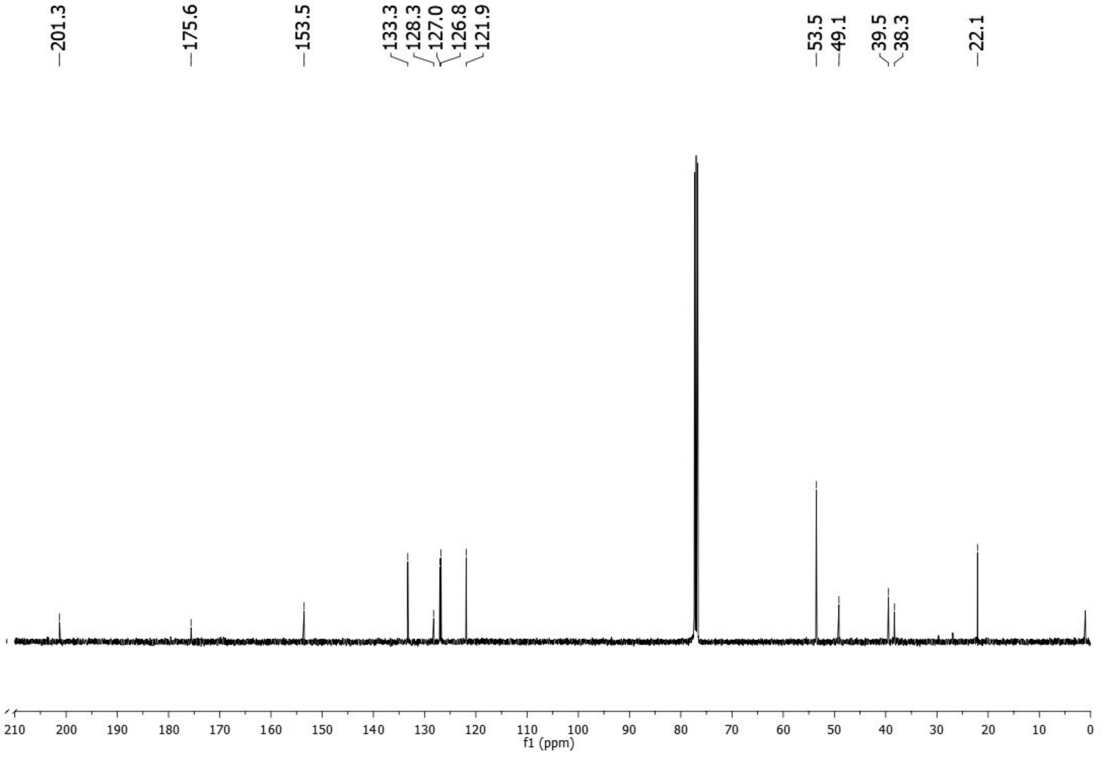

[24] Typical ¹H NMR signals pattern generated by these kind of α-methylene cyclobutanes is highlighted.

(^{13}C)$_2$-2a ^{1}HNMR (600 MHz, CDCl$_3$)[25]

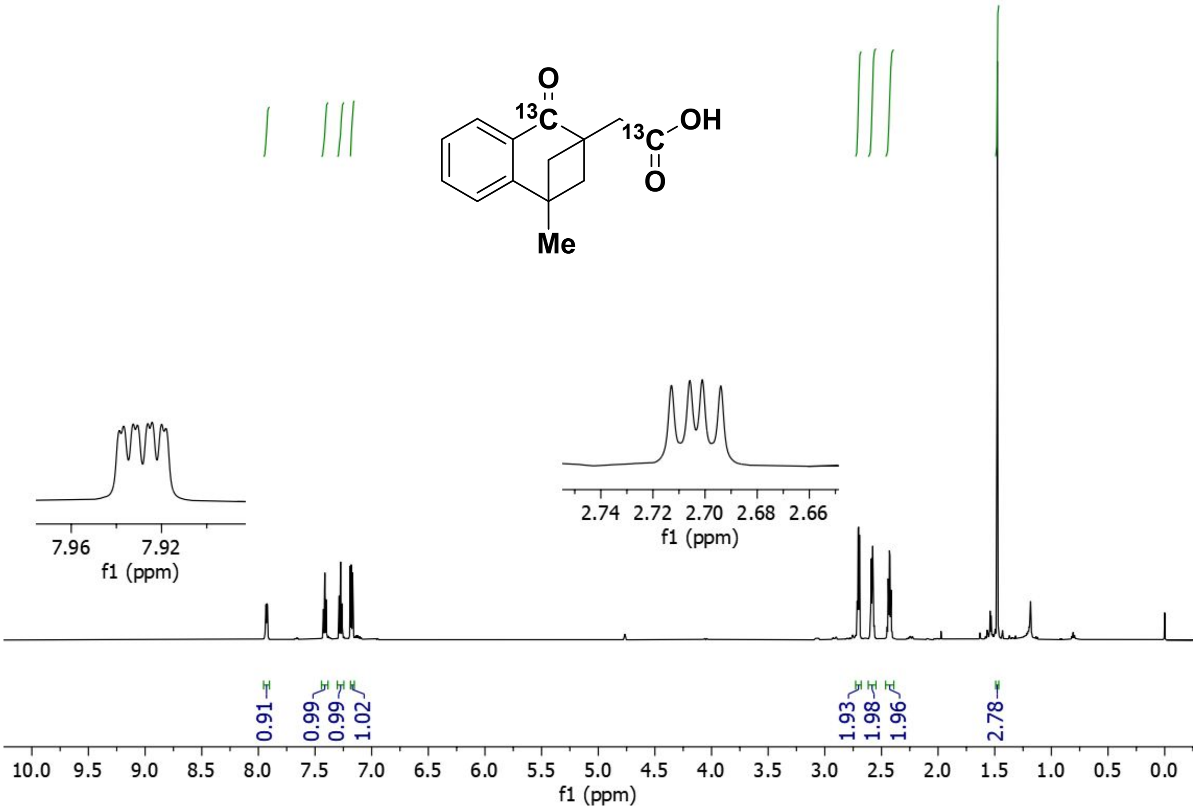

(^{13}C)$_2$-2a ^{13}CNMR (151 MHz, CDCl$_3$)

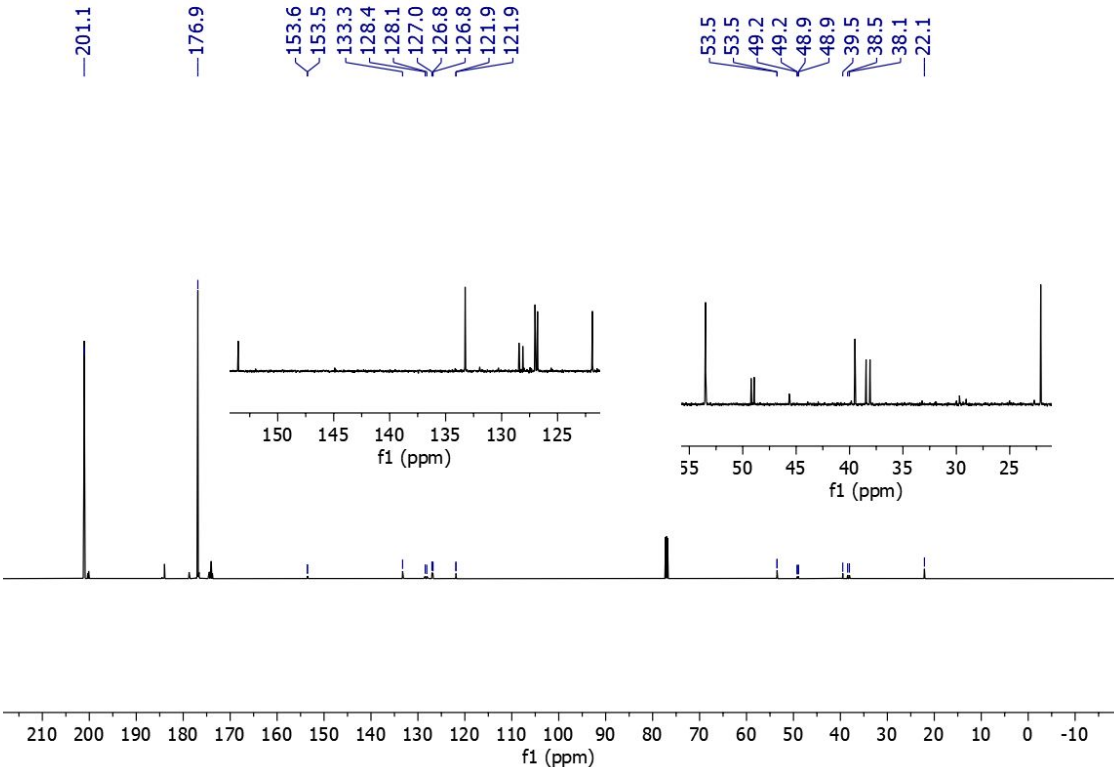

[25] Signals showing ^{1}H-^{13}C couplings are highlighted.

2b ^{1}HNMR (400 MHz, $CDCl_3$)

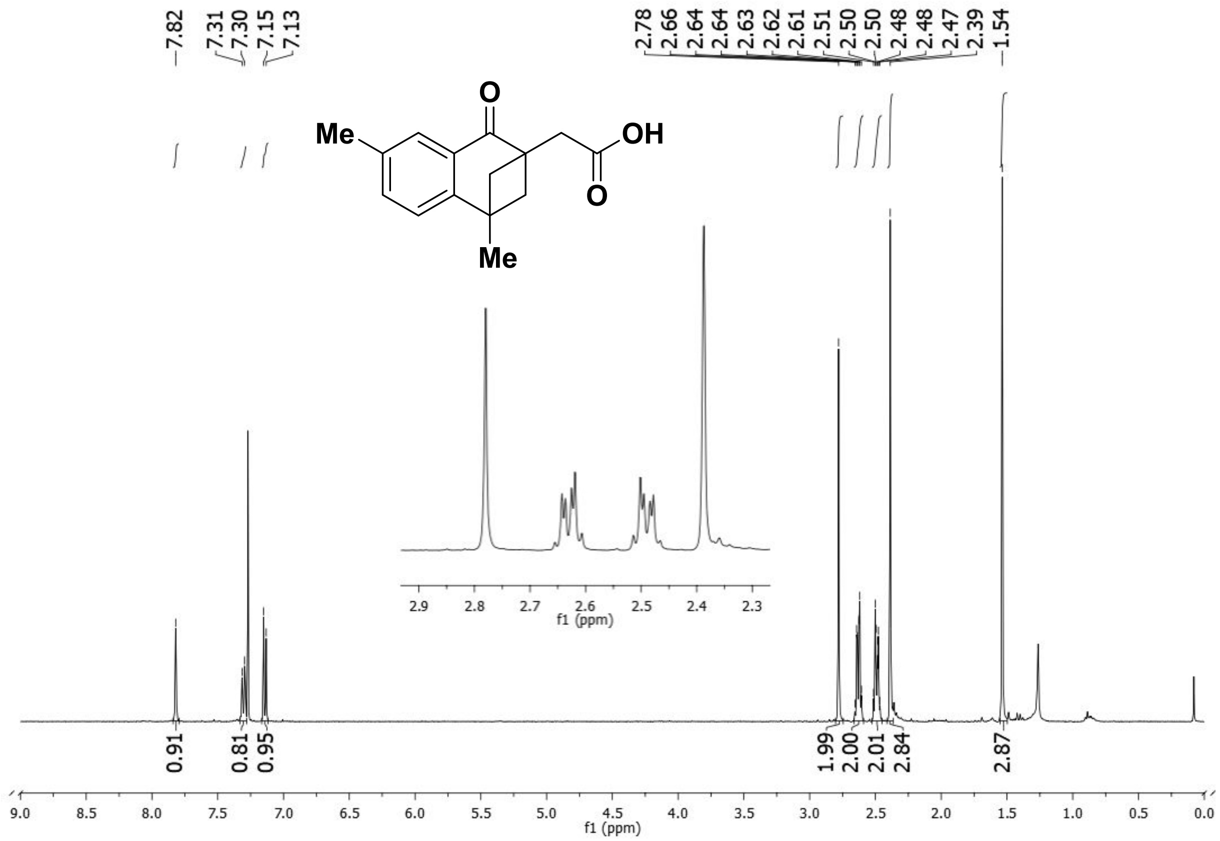

2b ^{13}CNMR (100 MHz, $CDCl_3$)

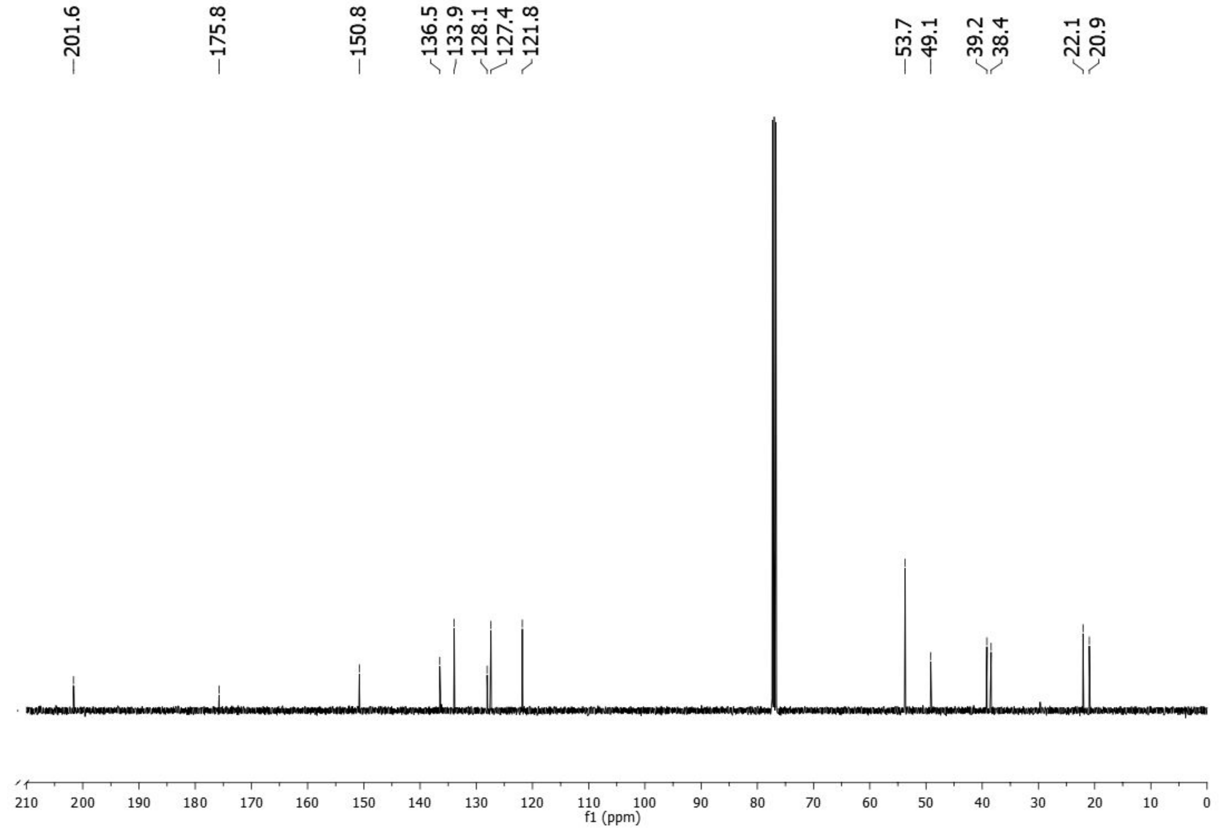

2c ^{1}HNMR (400 MHz, $CDCl_3$)

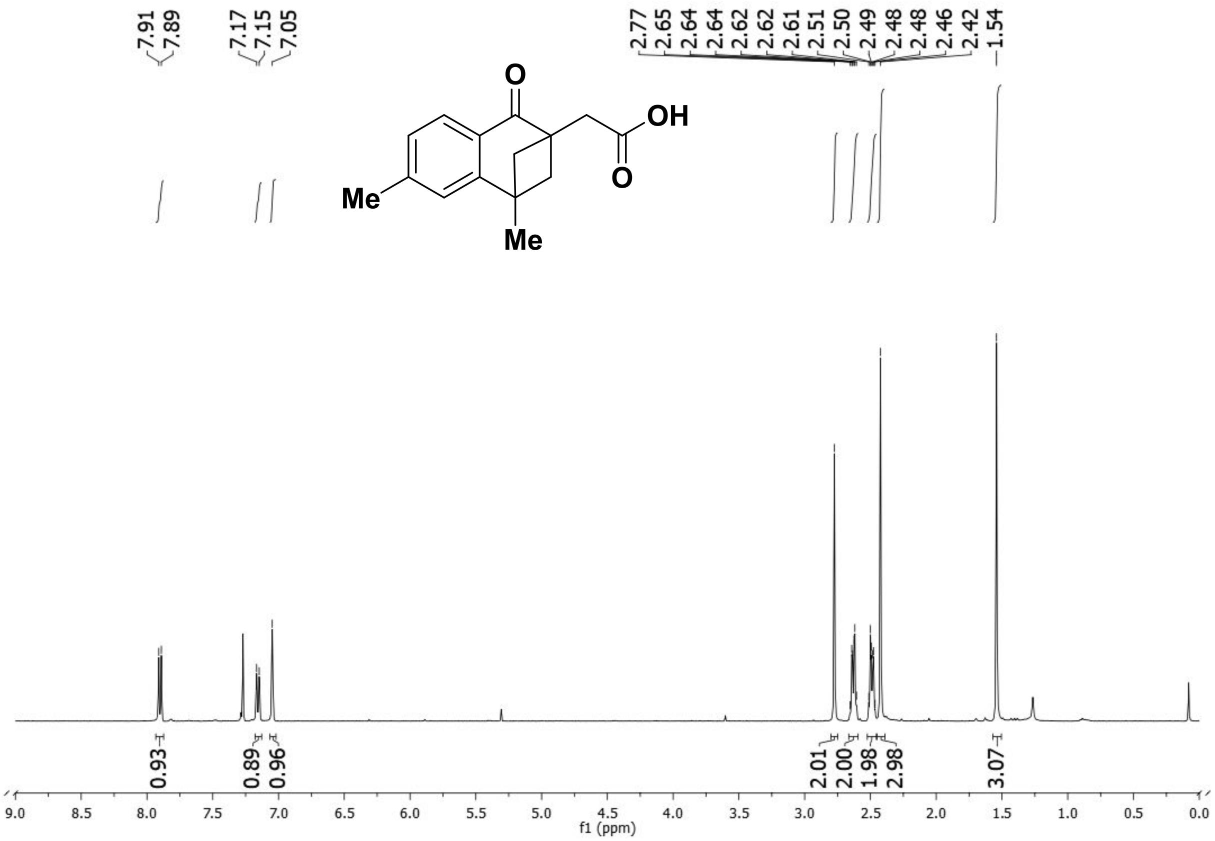

2c ^{13}CNMR (100 MHz, $CDCl_3$)

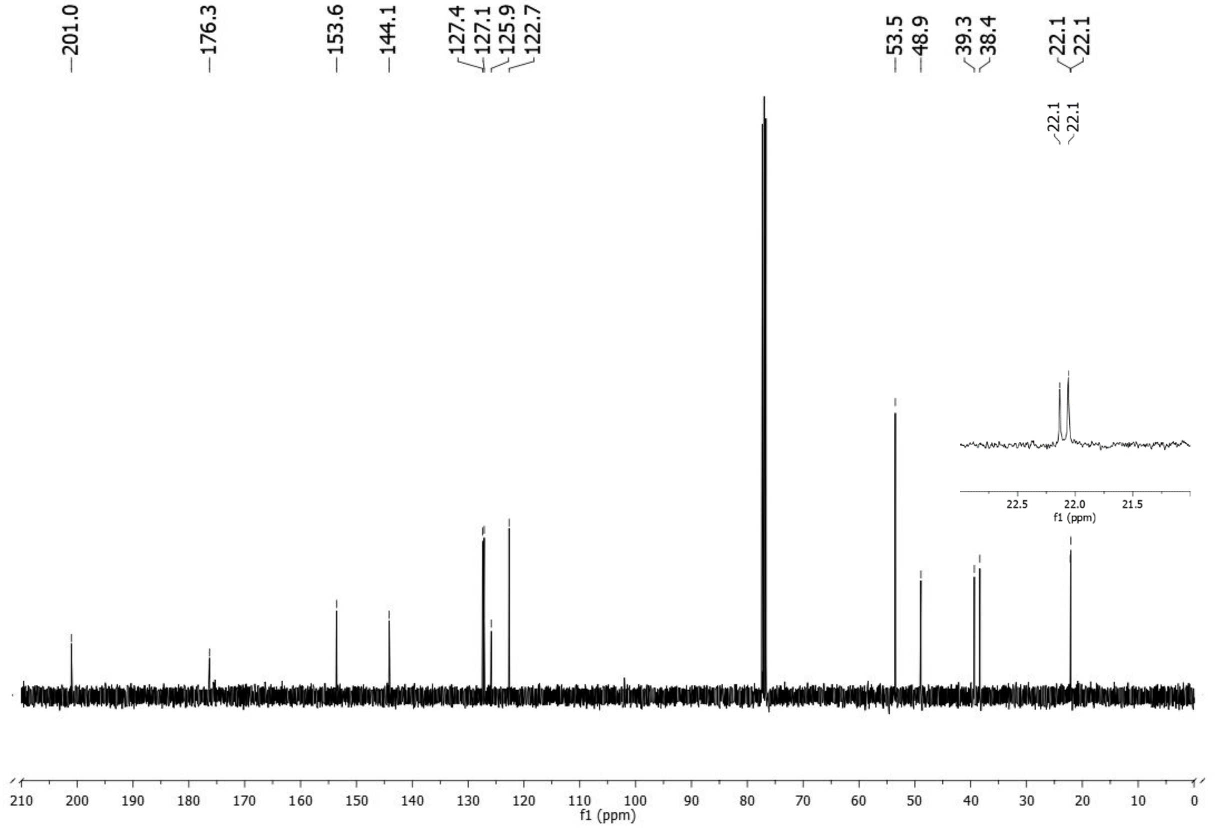

2d ^{1}HNMR (400 MHz, acetone-d_6)

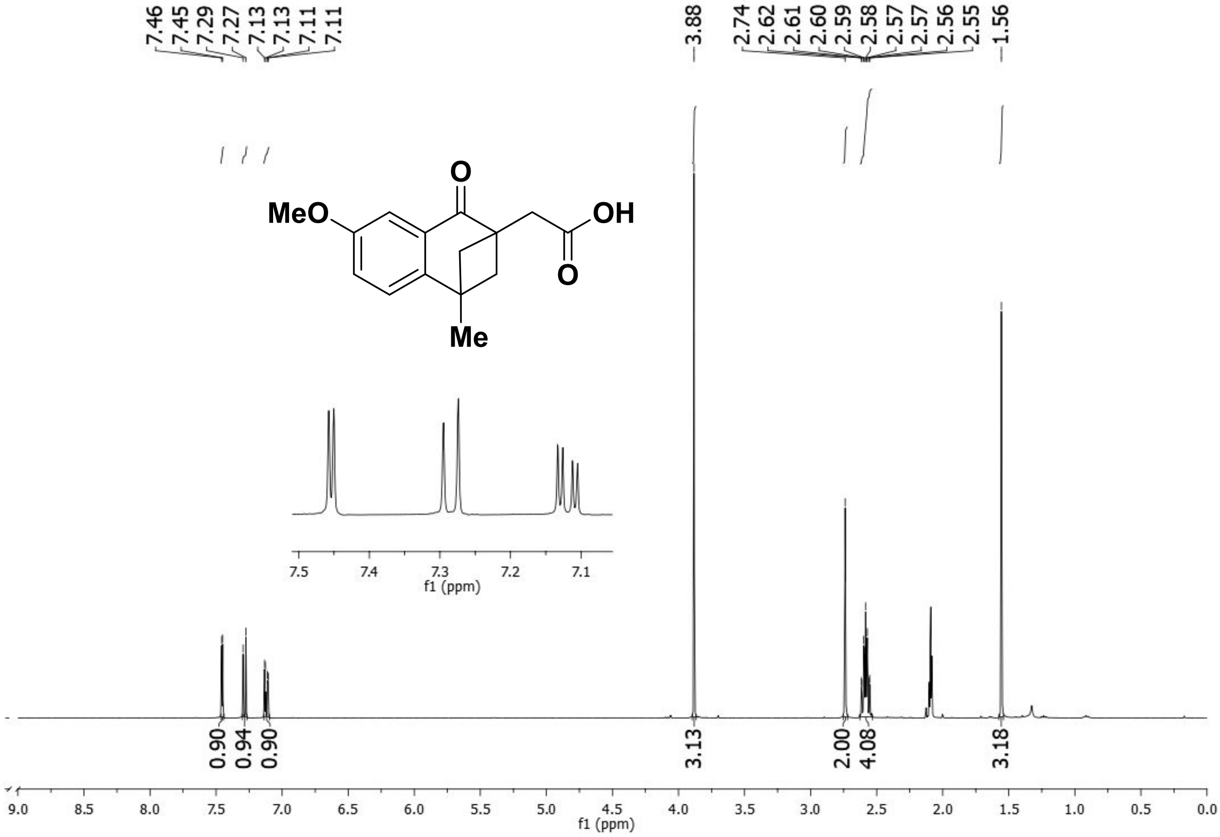

2d ^{13}CNMR (100 MHz, acetone-d_6)

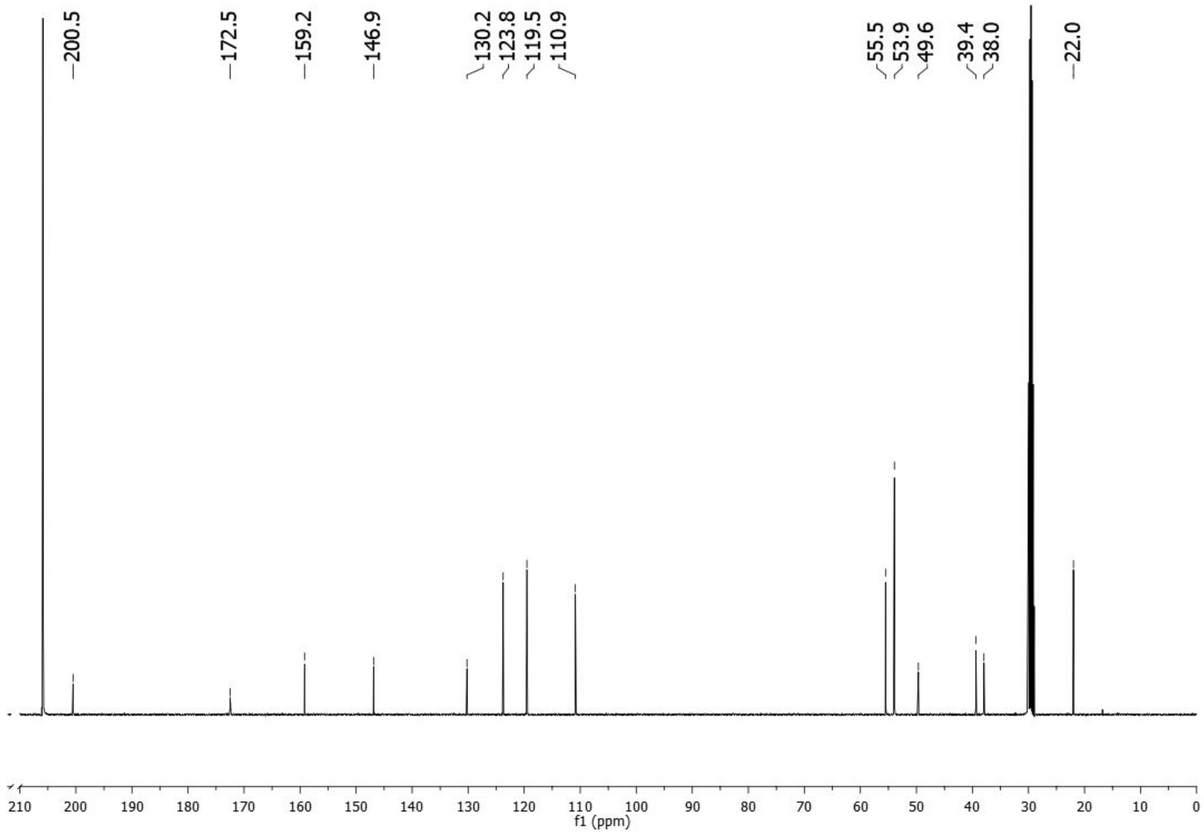

2e ^{1}HNMR (400 MHz, $CDCl_3$)

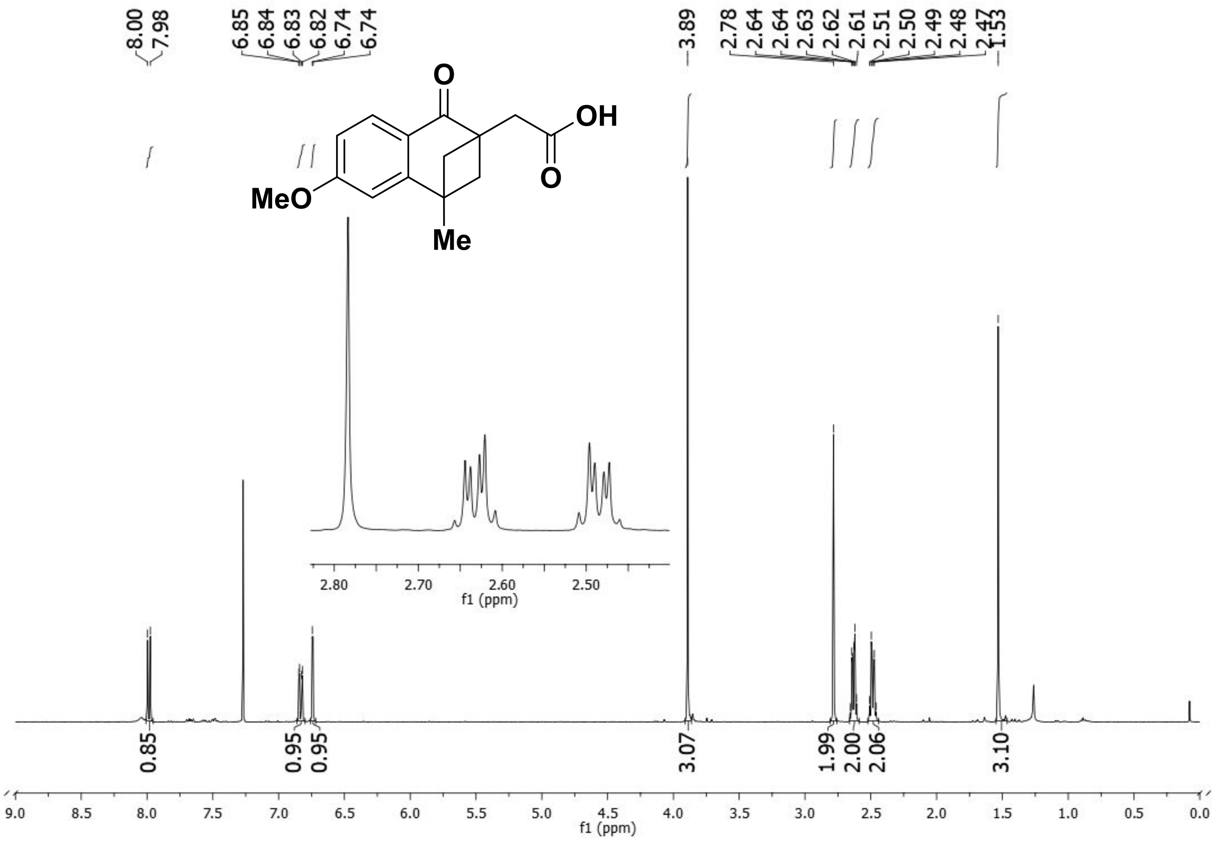

2e ^{13}CNMR (100 MHz, $CDCl_3$)

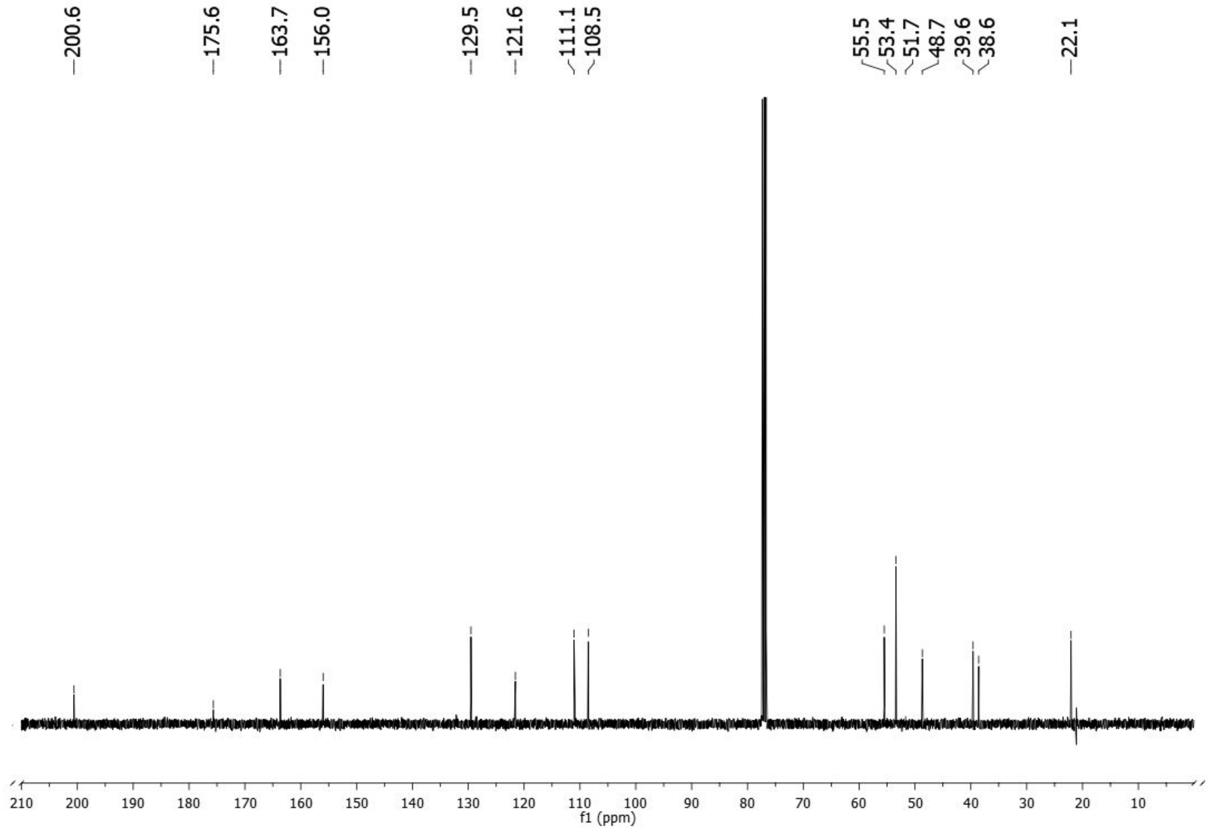

2f ^{1}HNMR (400 MHz, $CDCl_3$)

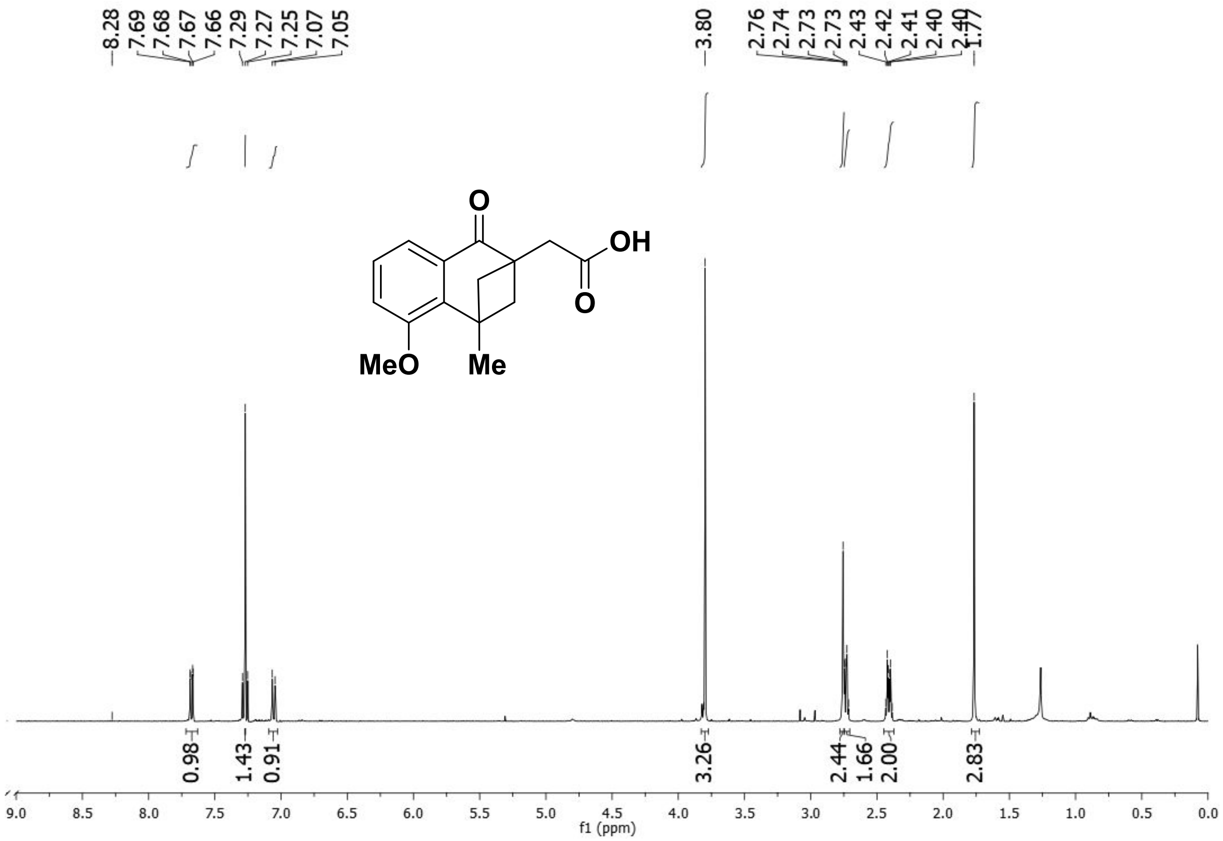

2f ^{13}CNMR (100 MHz, $CDCl_3$)

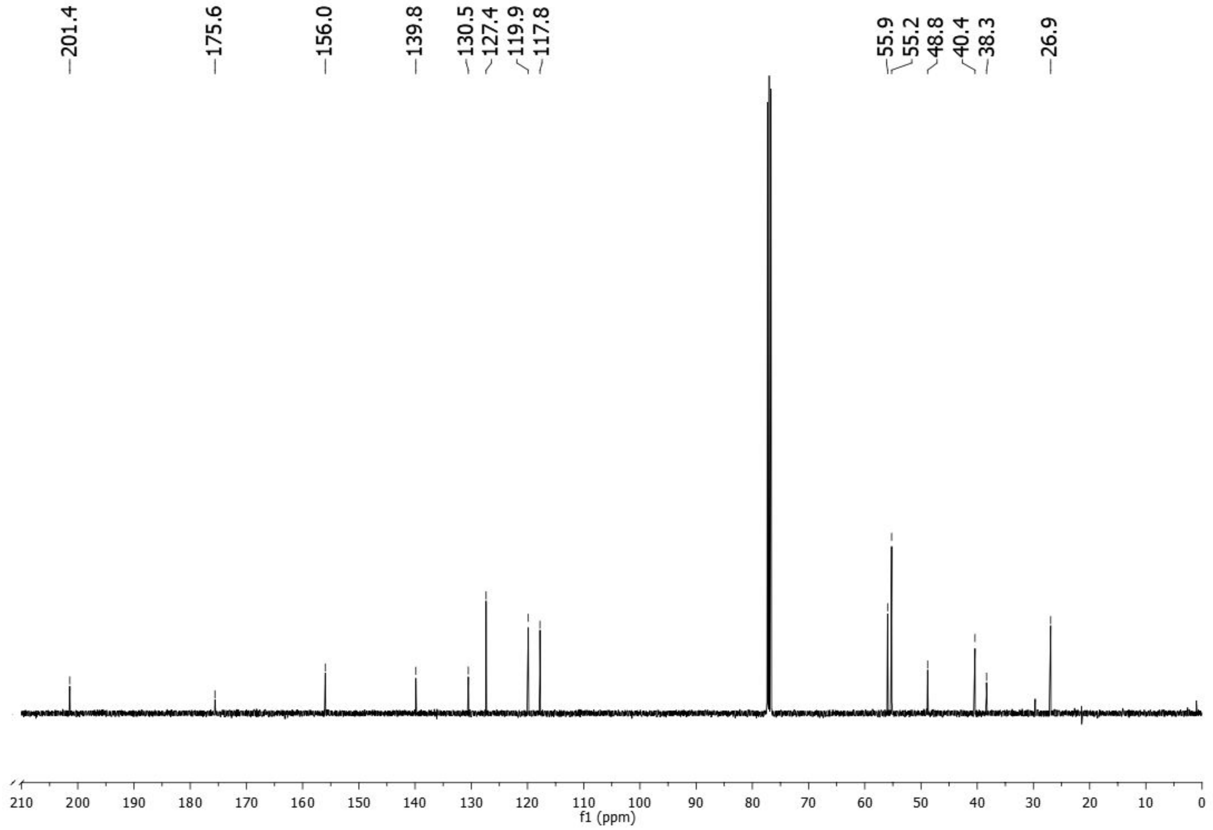

2g ¹HNMR (400 MHz, acetone-d_6)

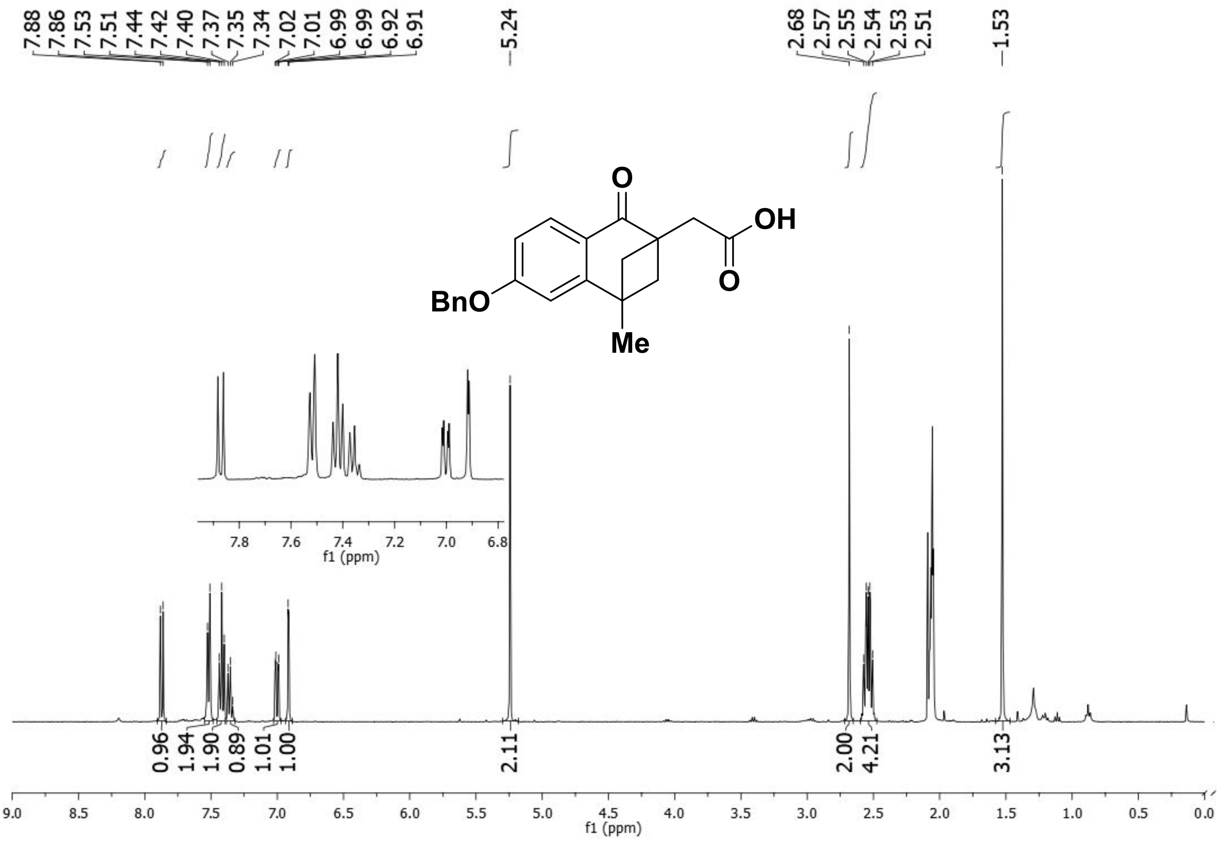

2g ¹³CNMR (100 MHz, acetone-d_6)

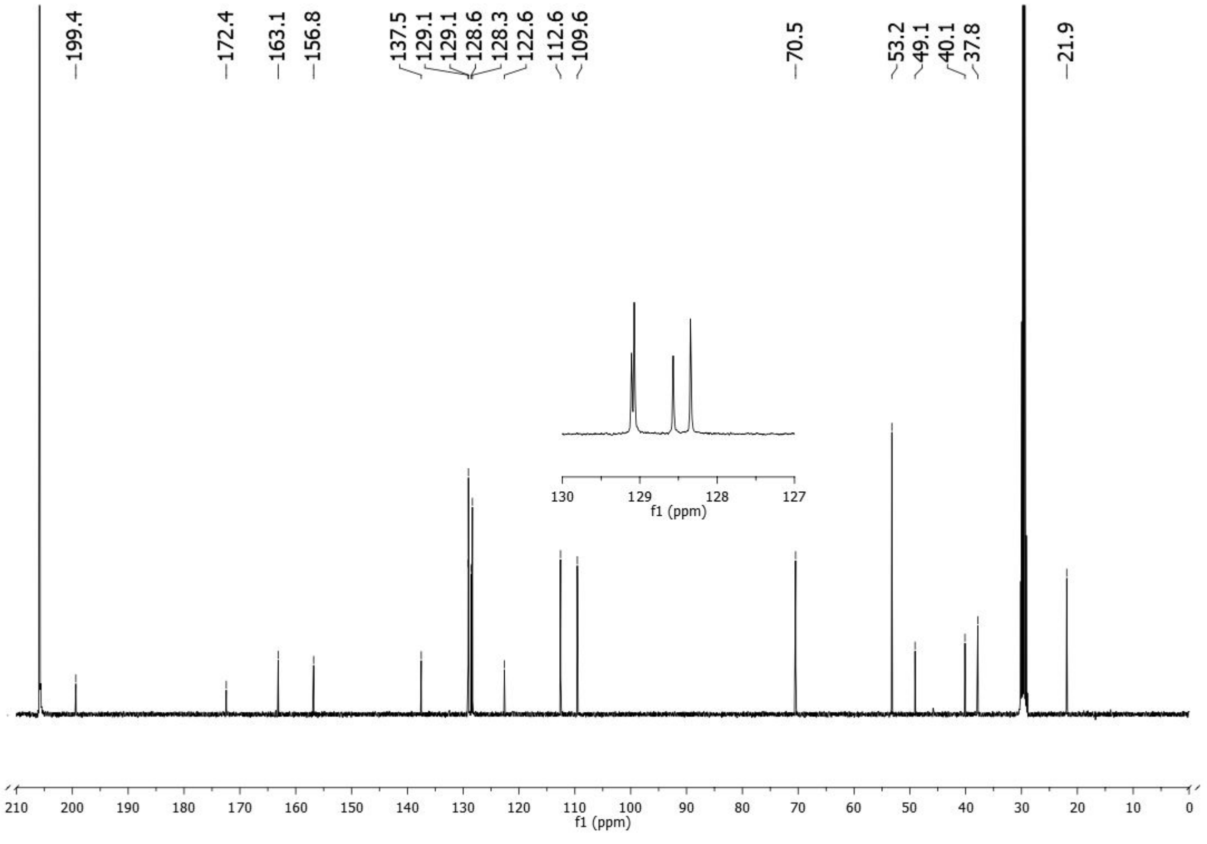

2h ^{1}HNMR (400 MHz, acetone-d$_6$)

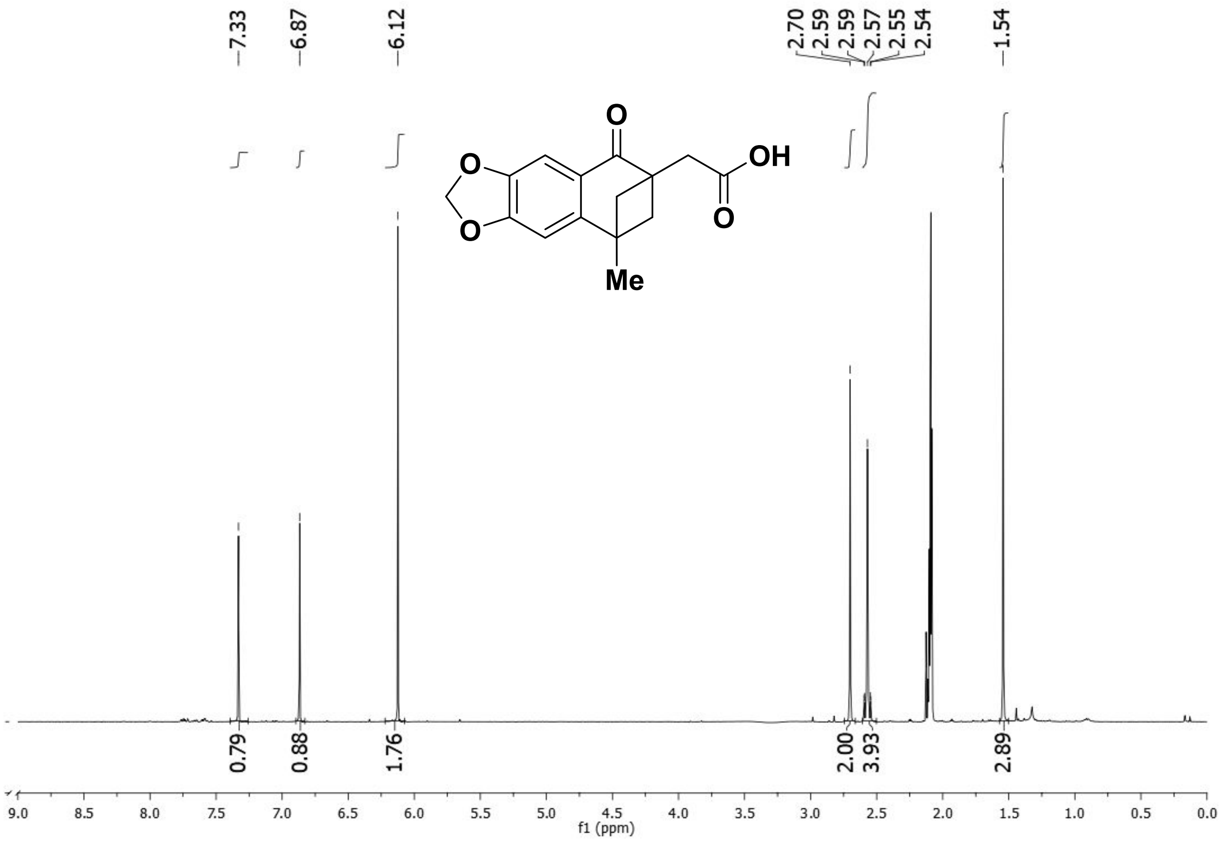

2h ^{13}CNMR (100 MHz, acetone-d$_6$)

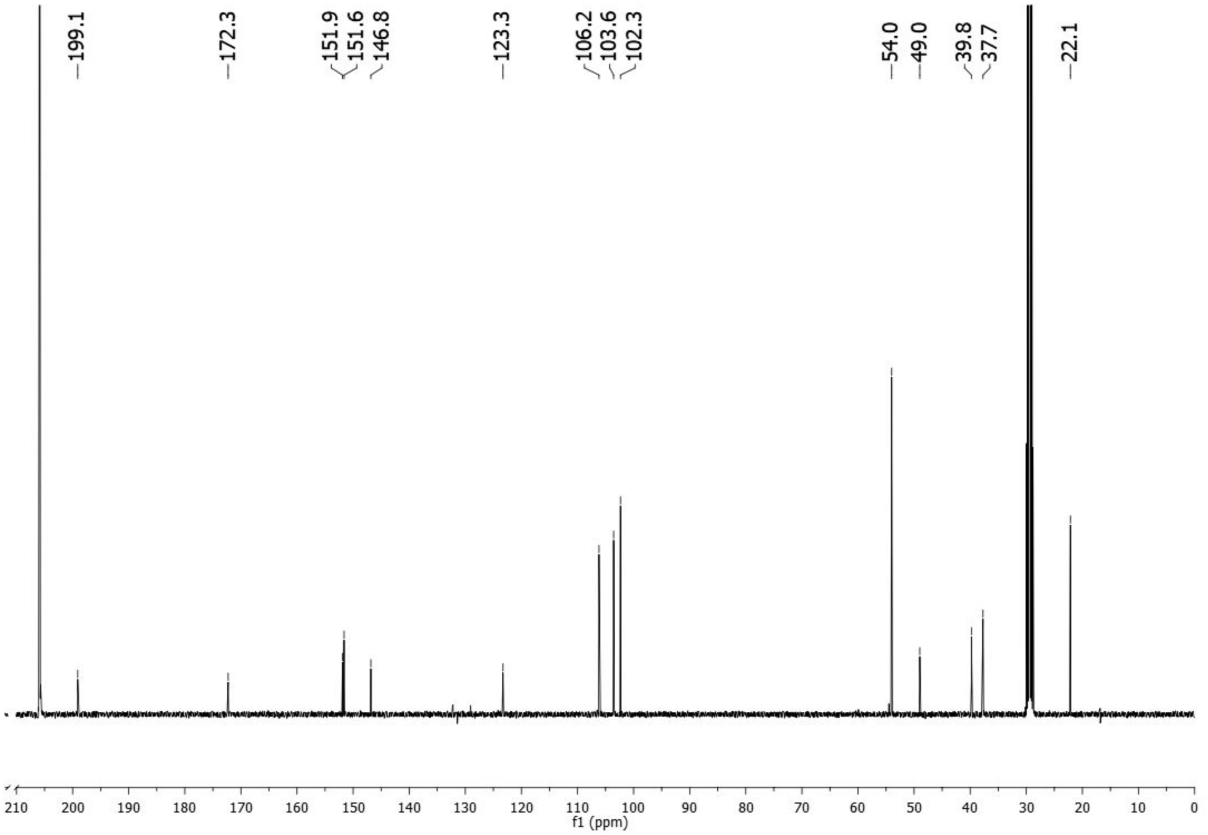

2i ¹HNMR (400 MHz, DMSO)

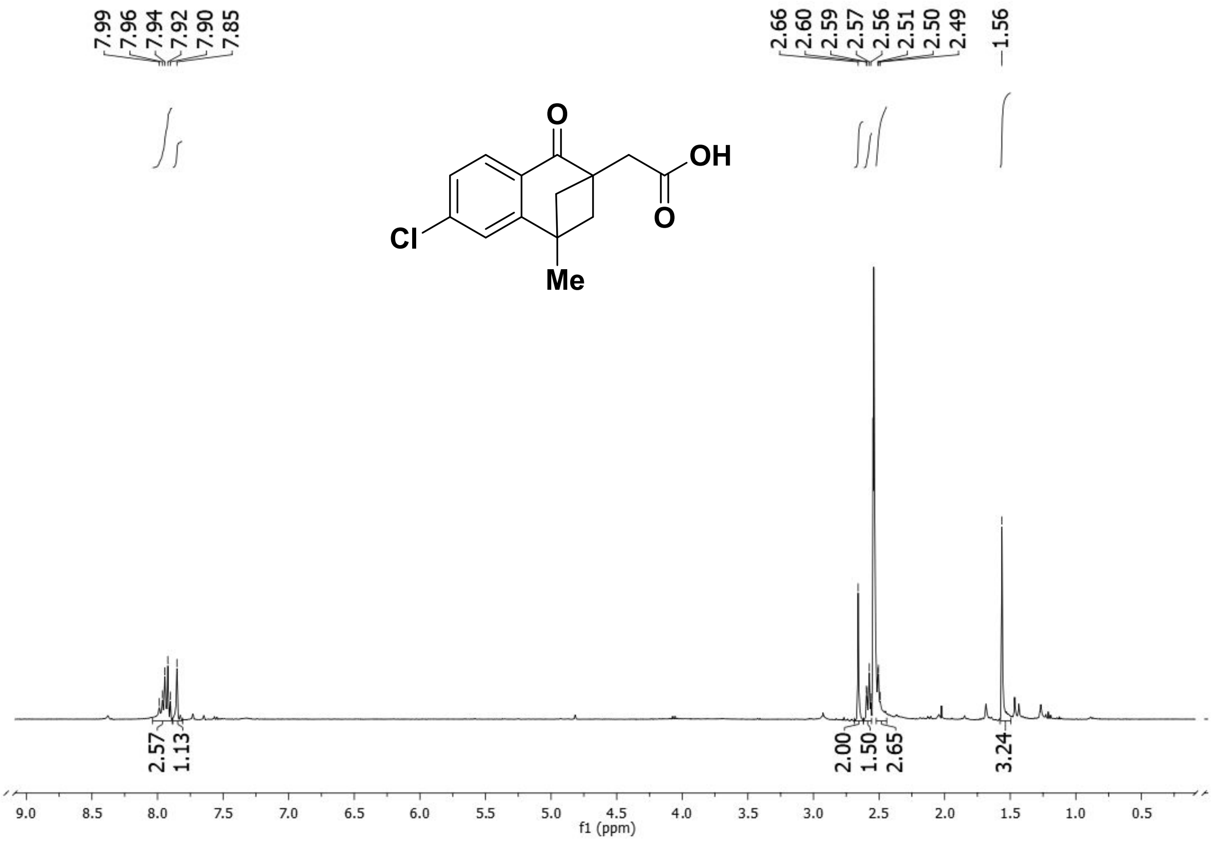

2i ¹³CNMR (100 MHz, DMSO)

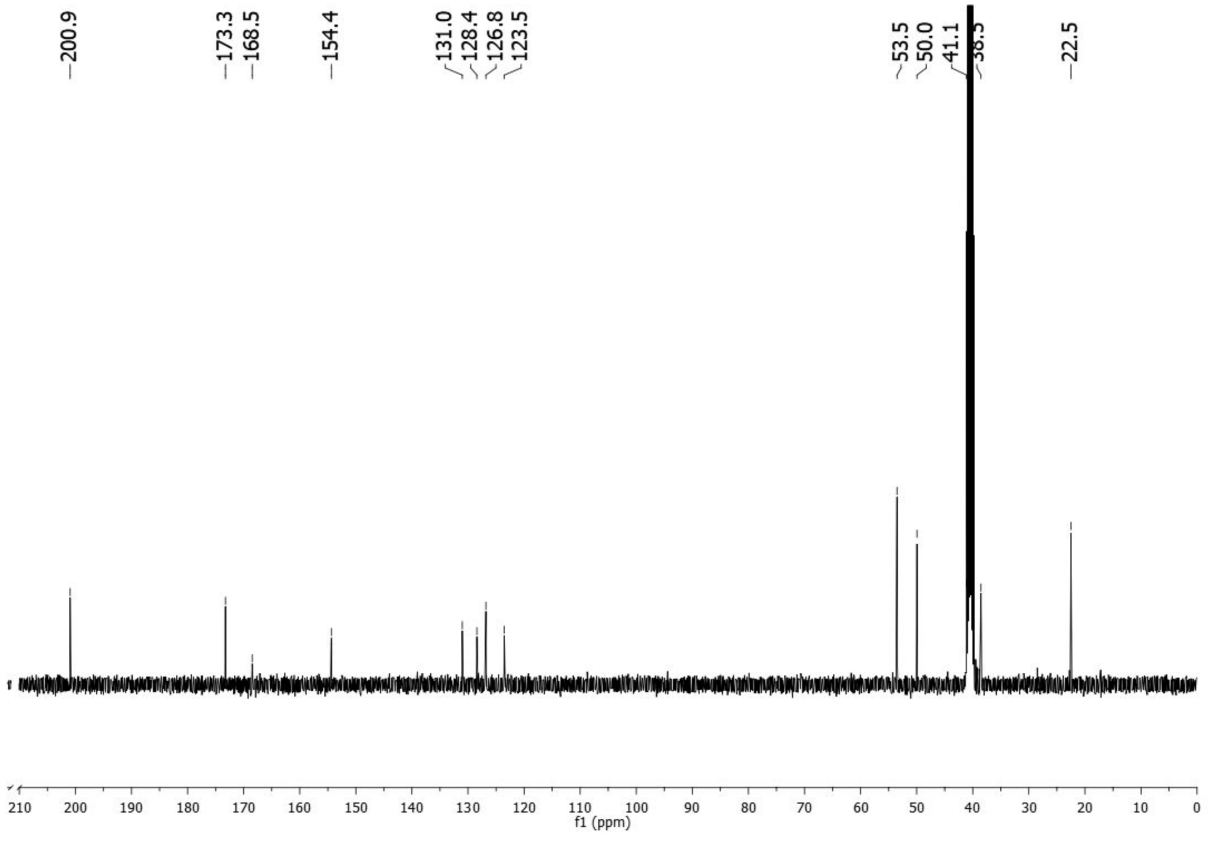

2j ^{1}HNMR (400 MHz, $CDCl_3$)

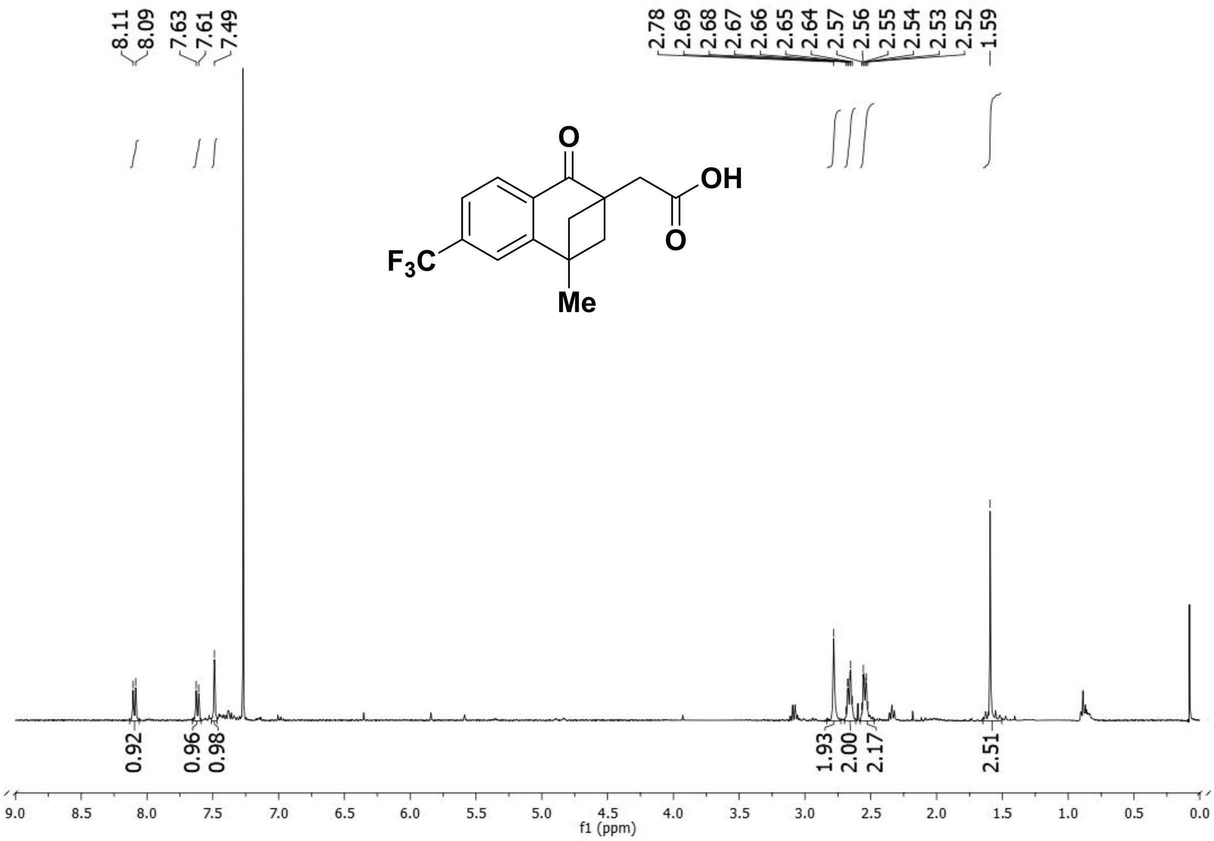

2j ^{13}CNMR (100 MHz, $CDCl_3$)

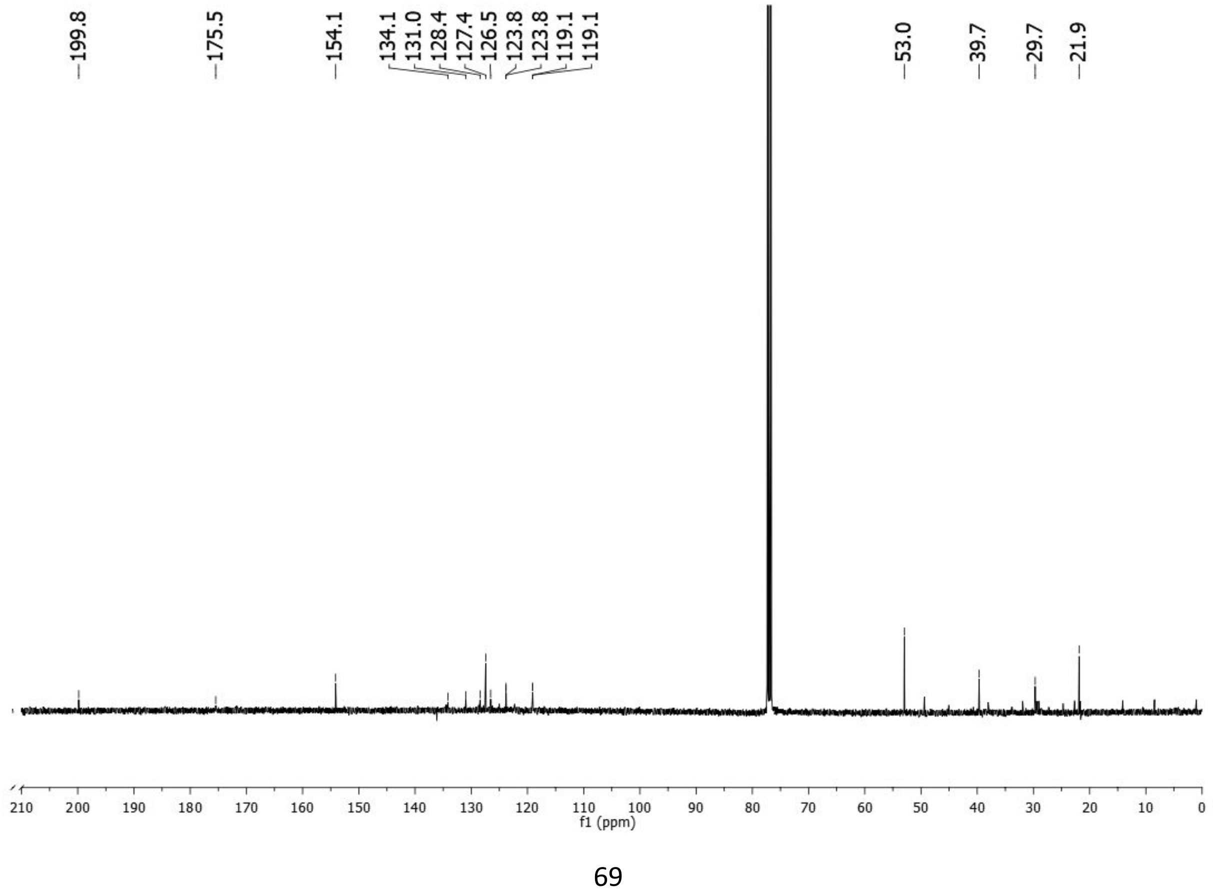

2j ^{19}FNMR (377 MHz, $CDCl_3$)

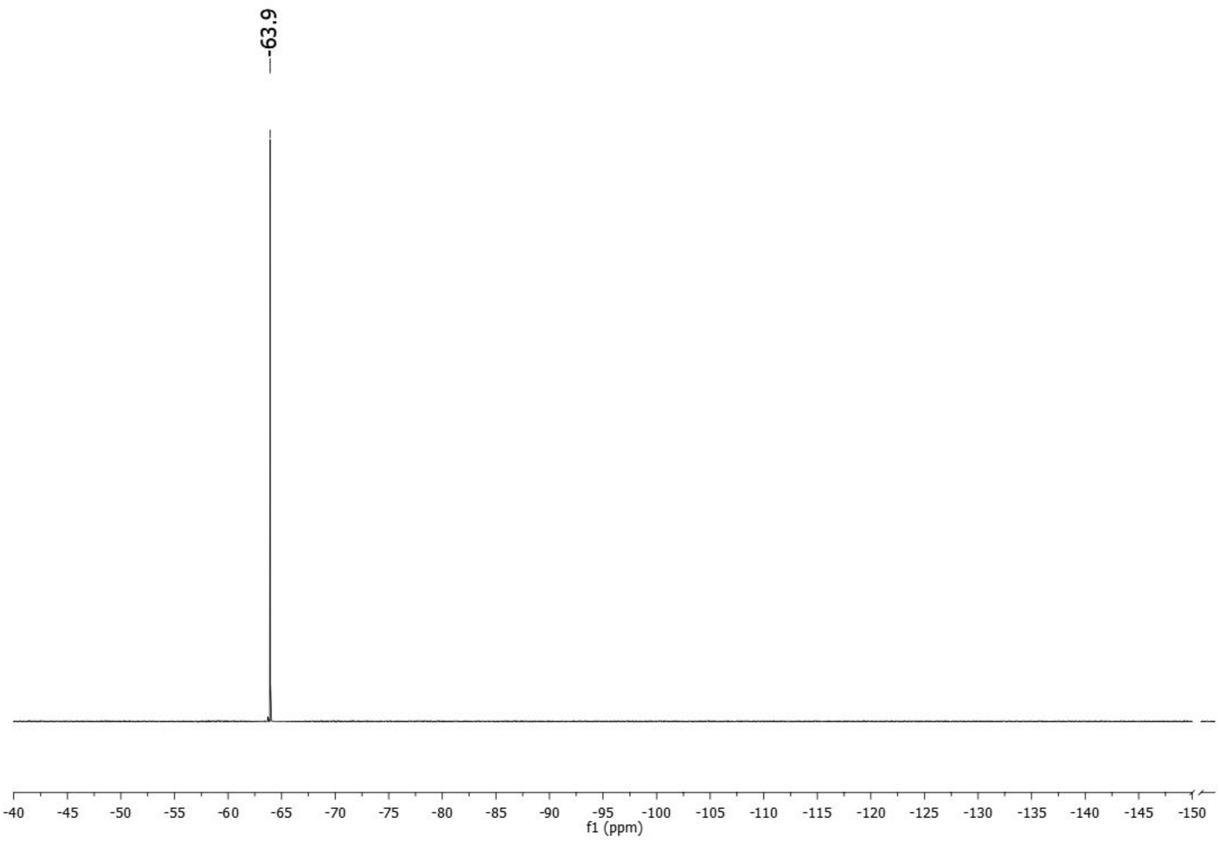

2k ^{1}HNMR (400 MHz, acetone-d$_6$)

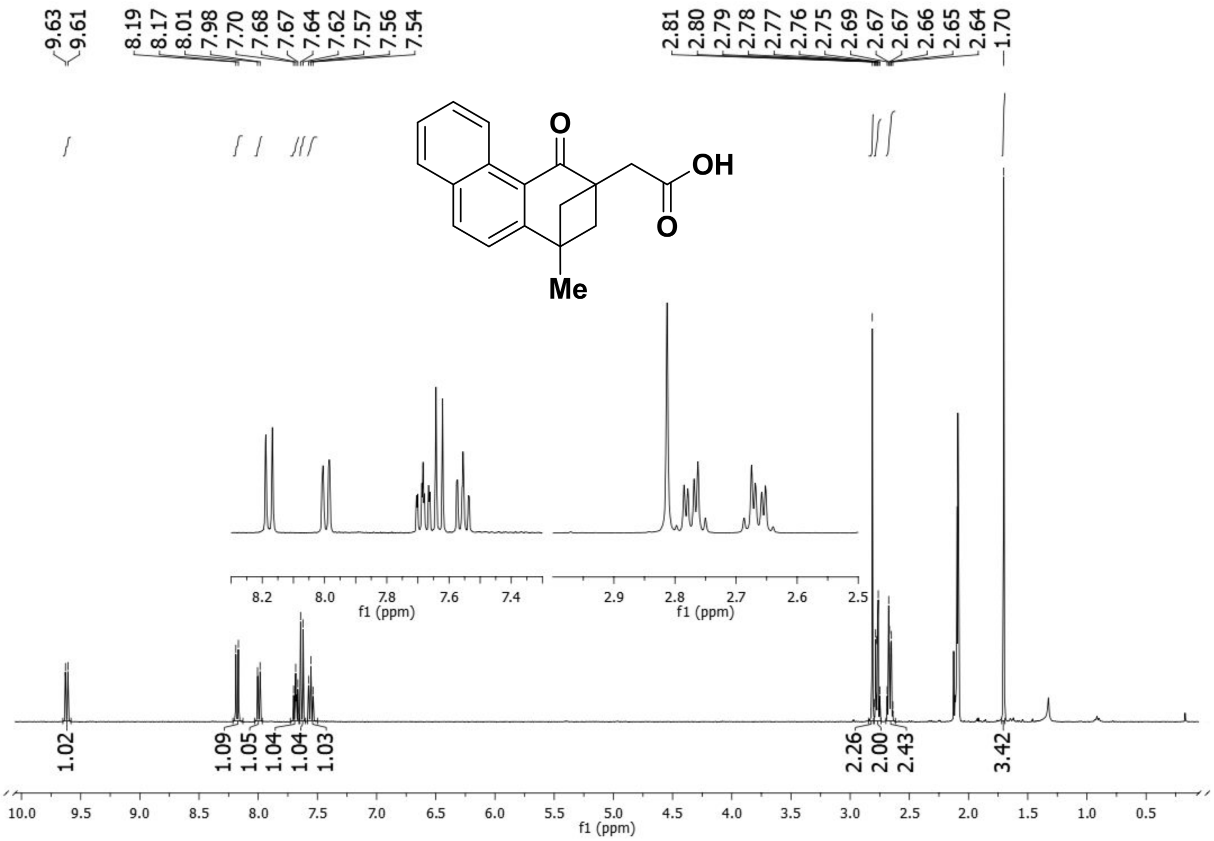

2k ^{13}CNMR (100 MHz, acetone-d$_6$)

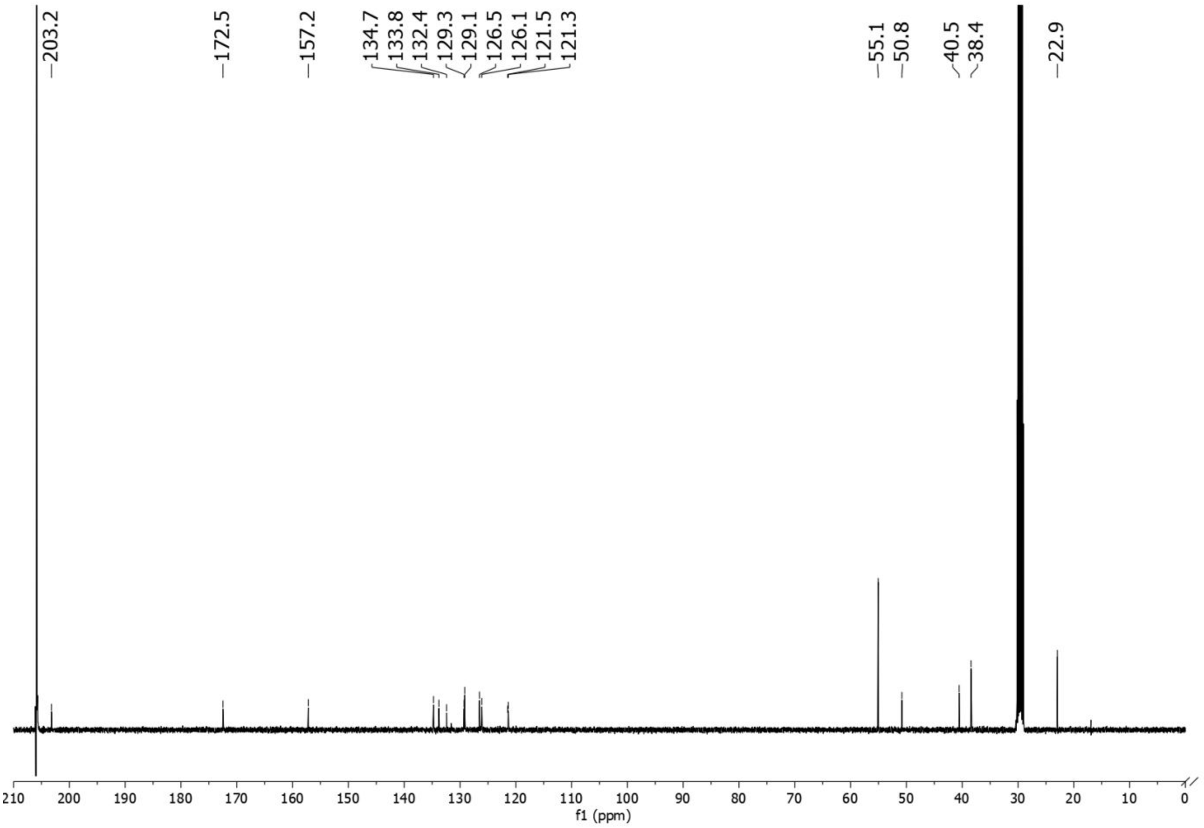

2l ^{1}HNMR (400 MHz, $CDCl_3$)

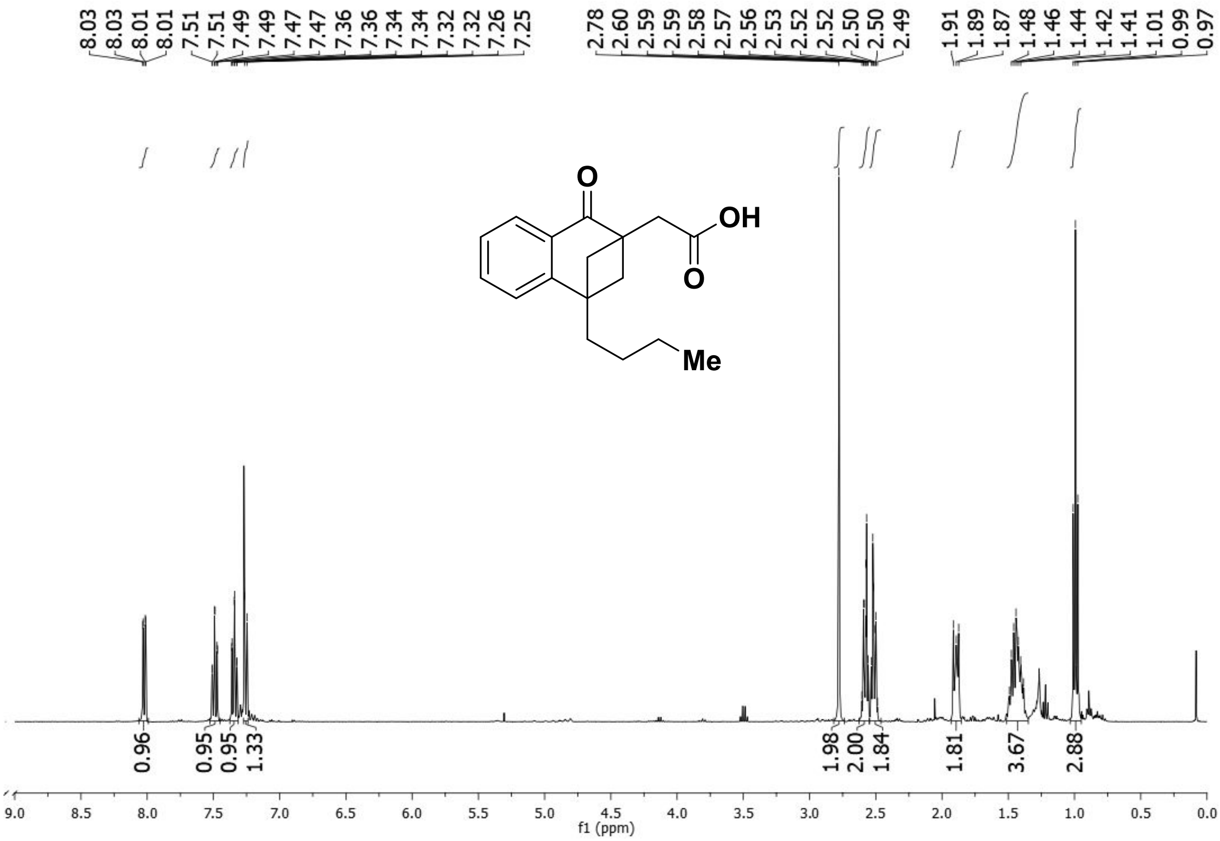

2l ^{13}CNMR (100 MHz, $CDCl_3$)

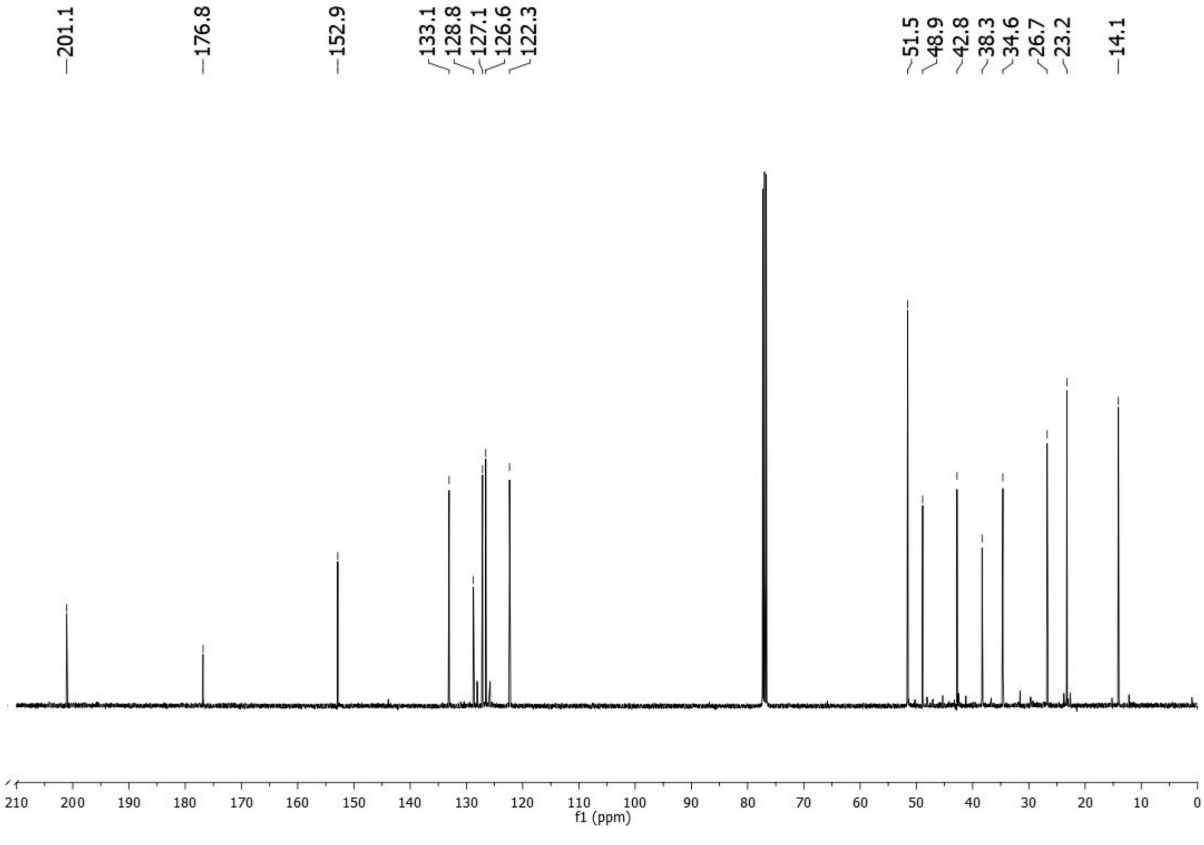

2m ^{1}HNMR (400 MHz, acetone-d$_6$)

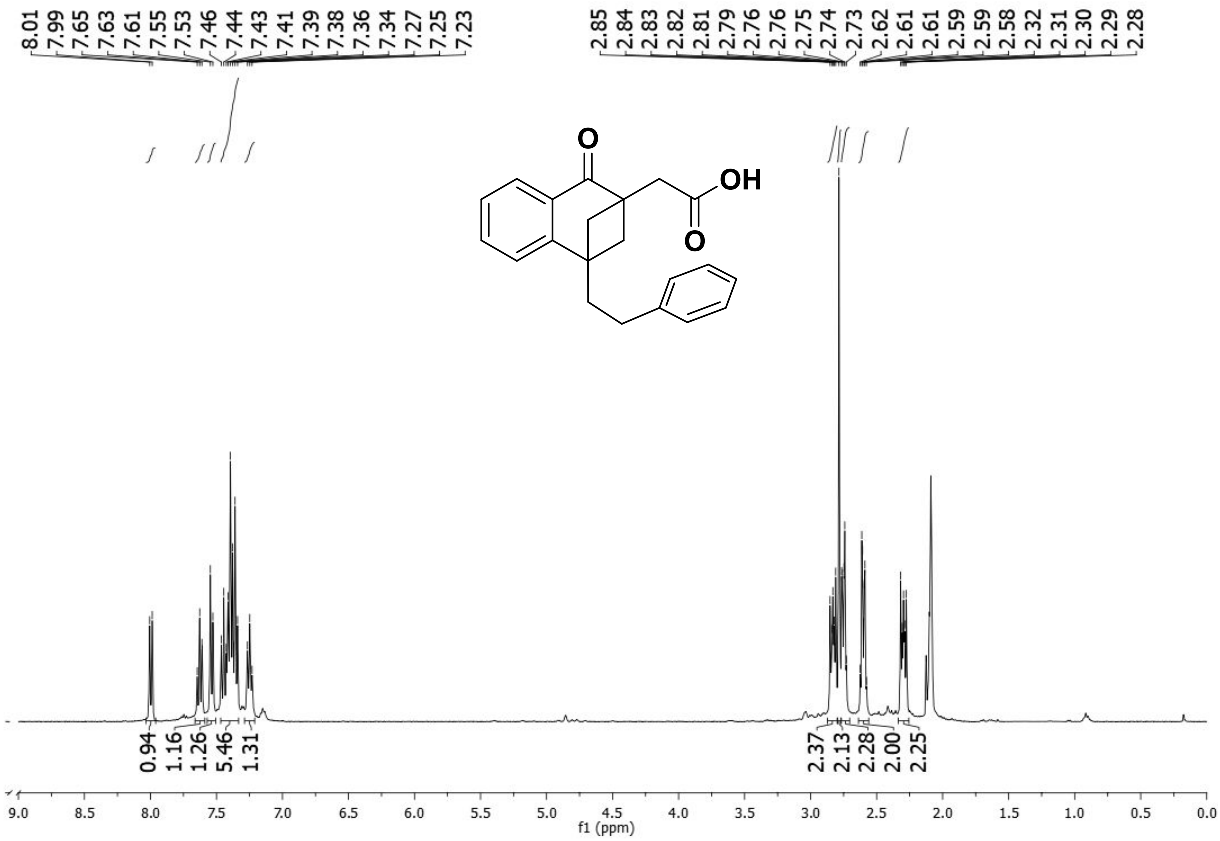

2m ^{13}CNMR (100 MHz, acetone-d$_6$)

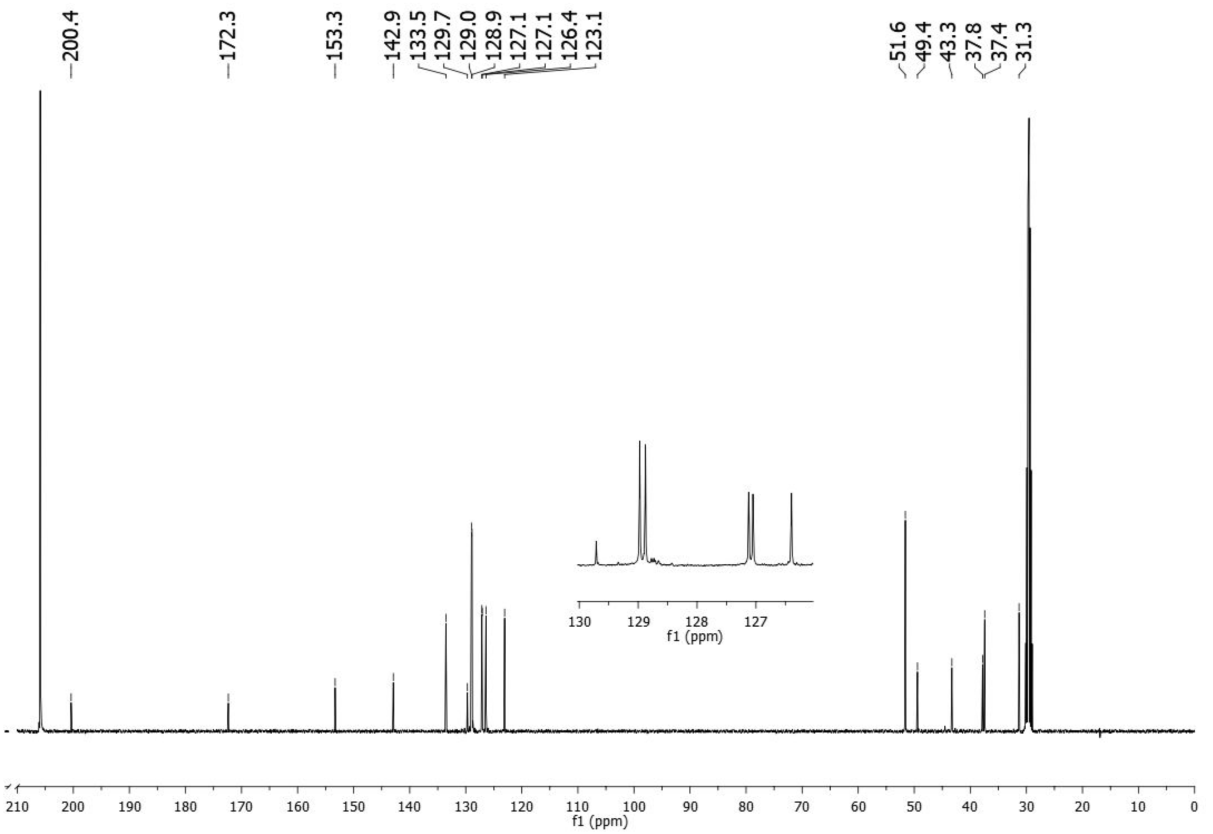

2n ^{1}HNMR (400 MHz, acetone-d_6)

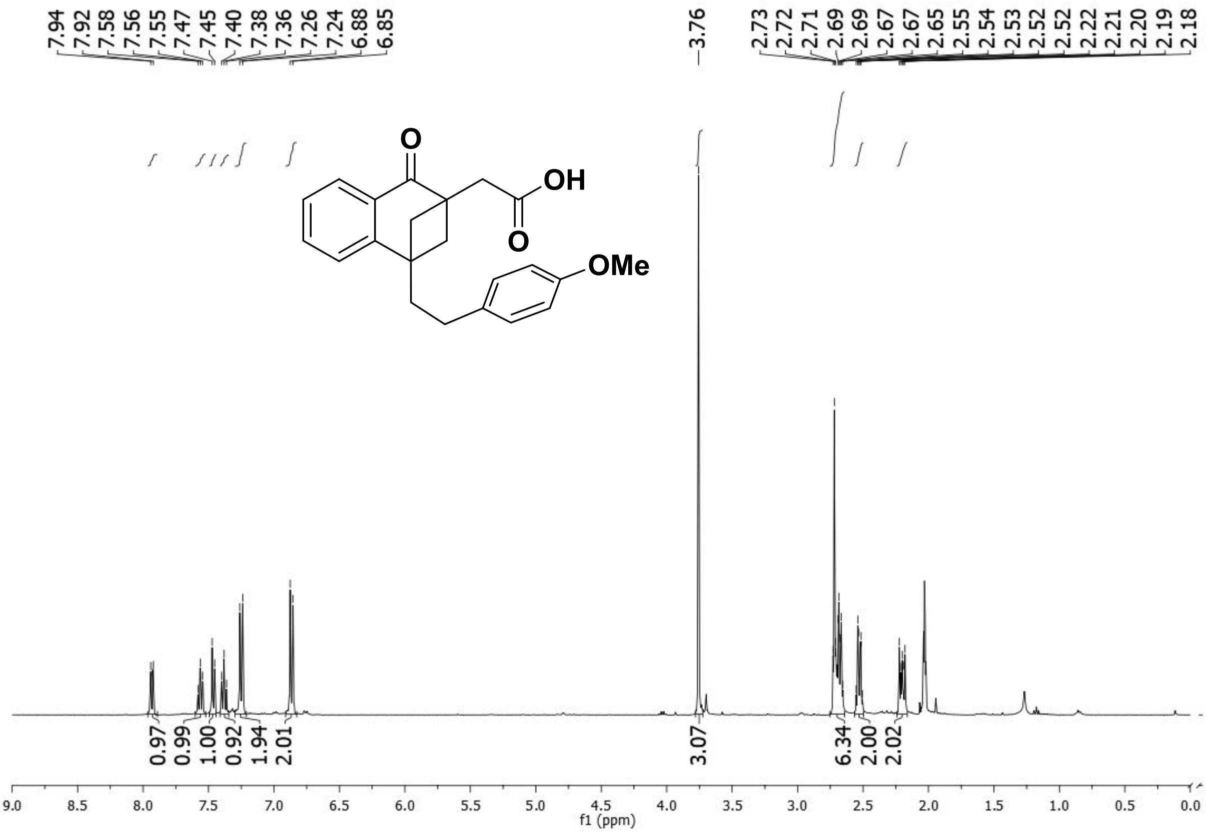

2n ^{13}CNMR (100 MHz, acetone-d_6)

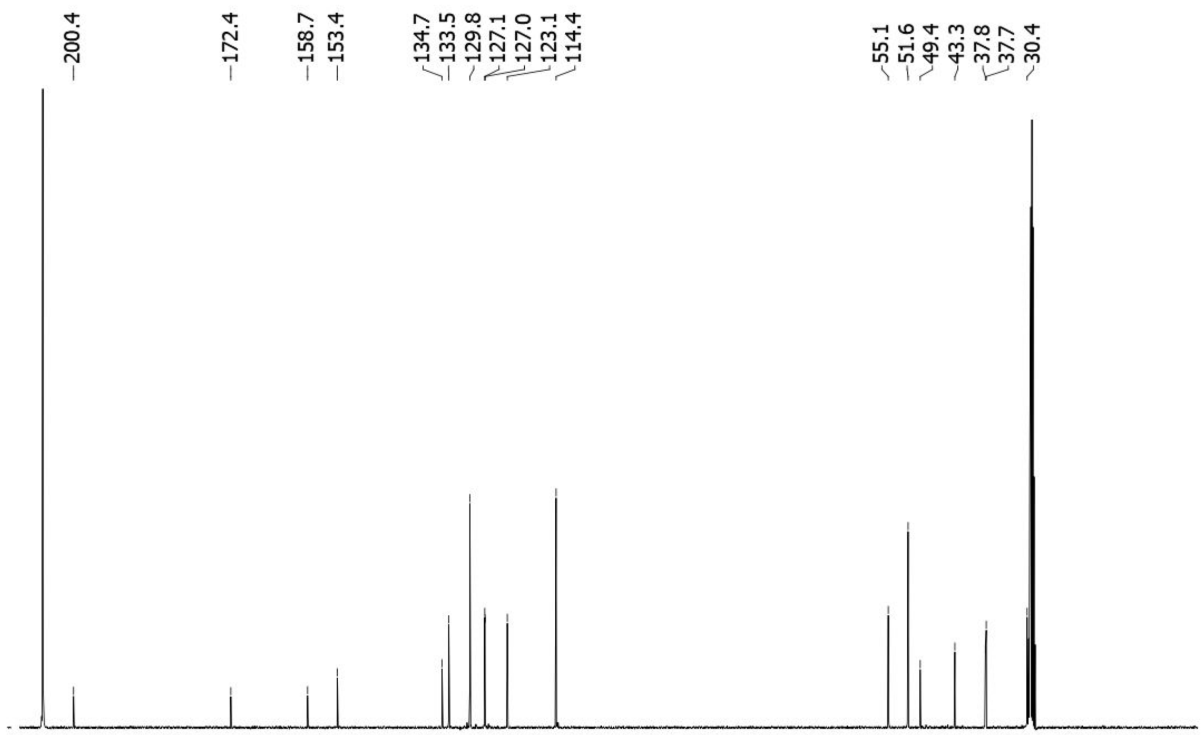

2o ^{1}HNMR (400 MHz, acetone-d_6)

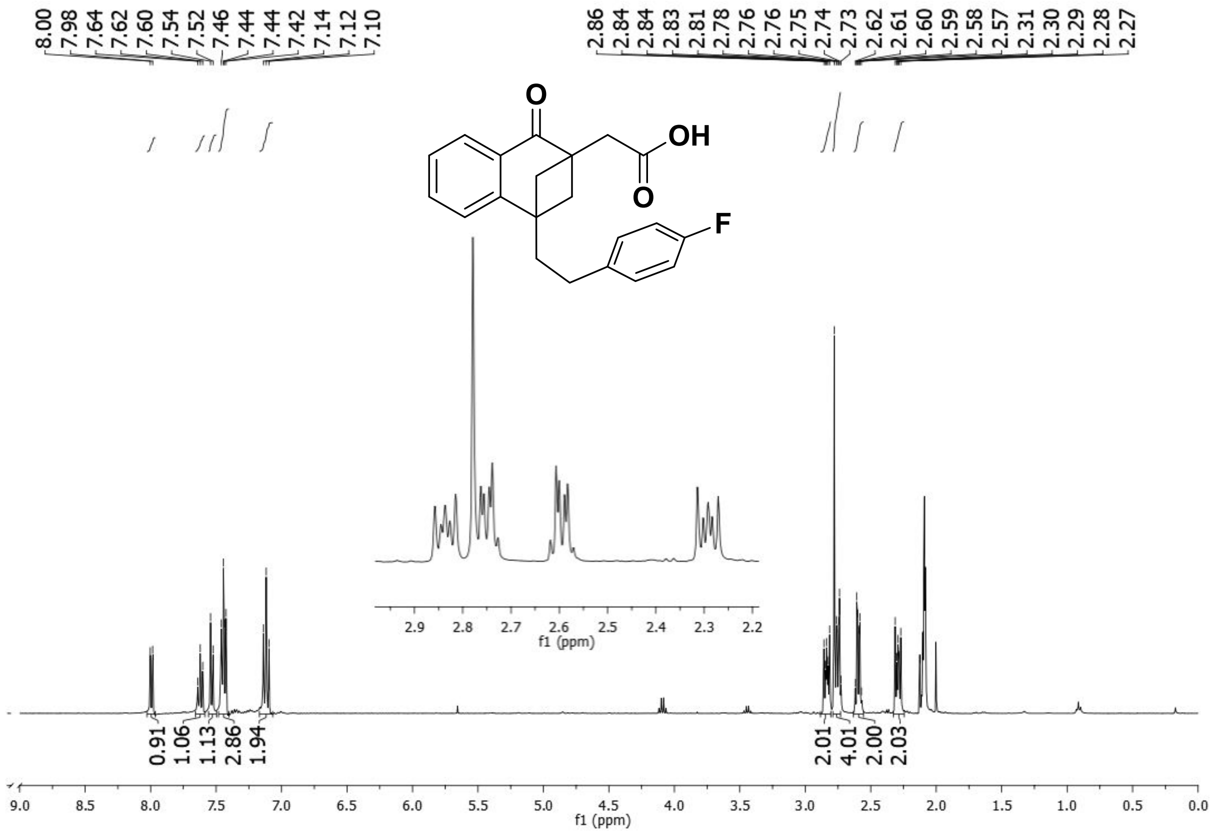

2o ^{13}CNMR (100 MHz, acetone-d_6) [26]

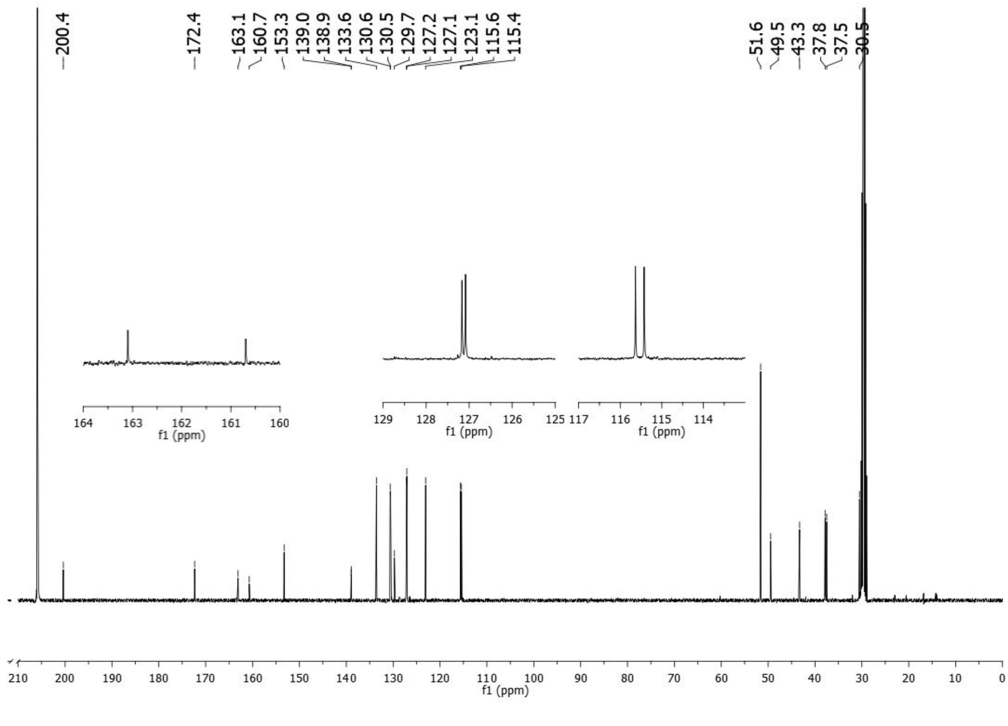

[26] Selected windows are all 4 ppm wide, thus *J* for ipso, orto and para position (from left to right) can be compared.

2o ^{19}FNMR (377 MHz, acetone-d_6)

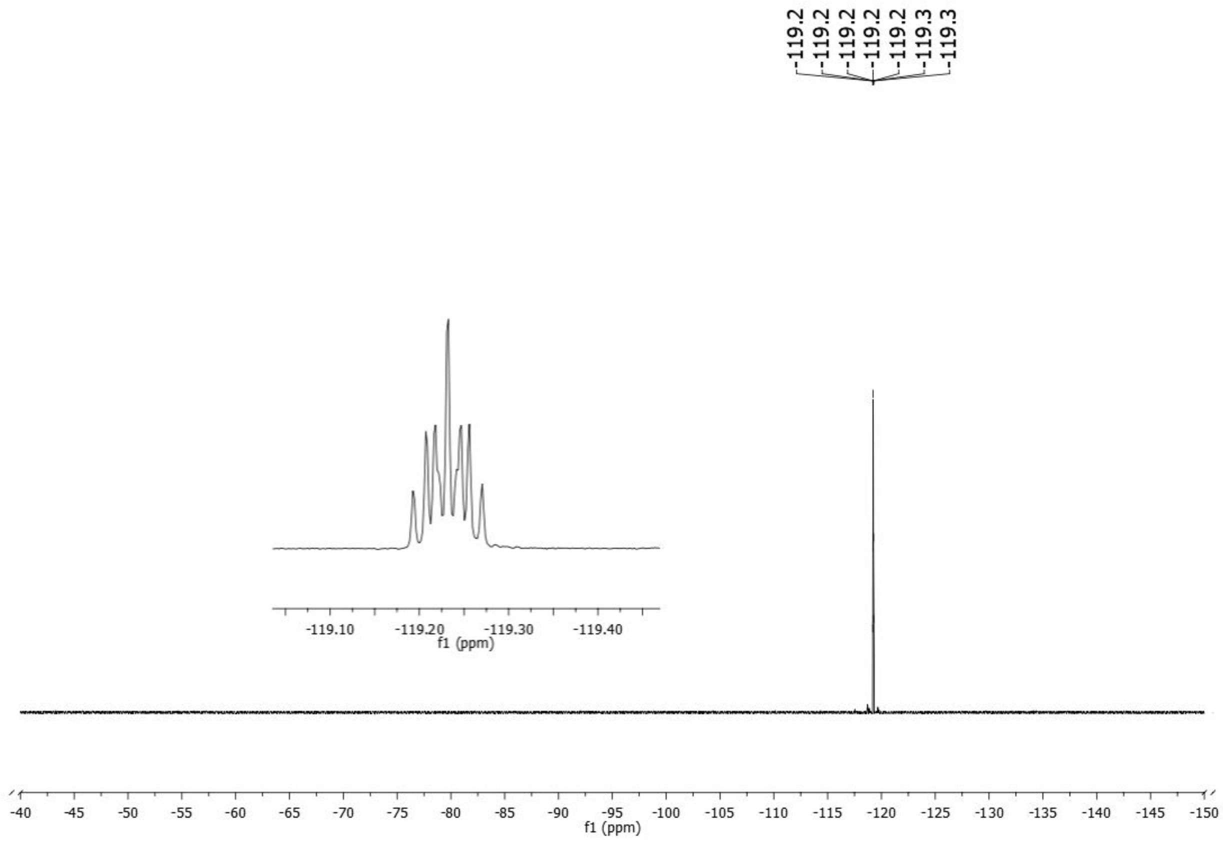

2p ^{1}HNMR (400 MHz, acetone-d$_6$)

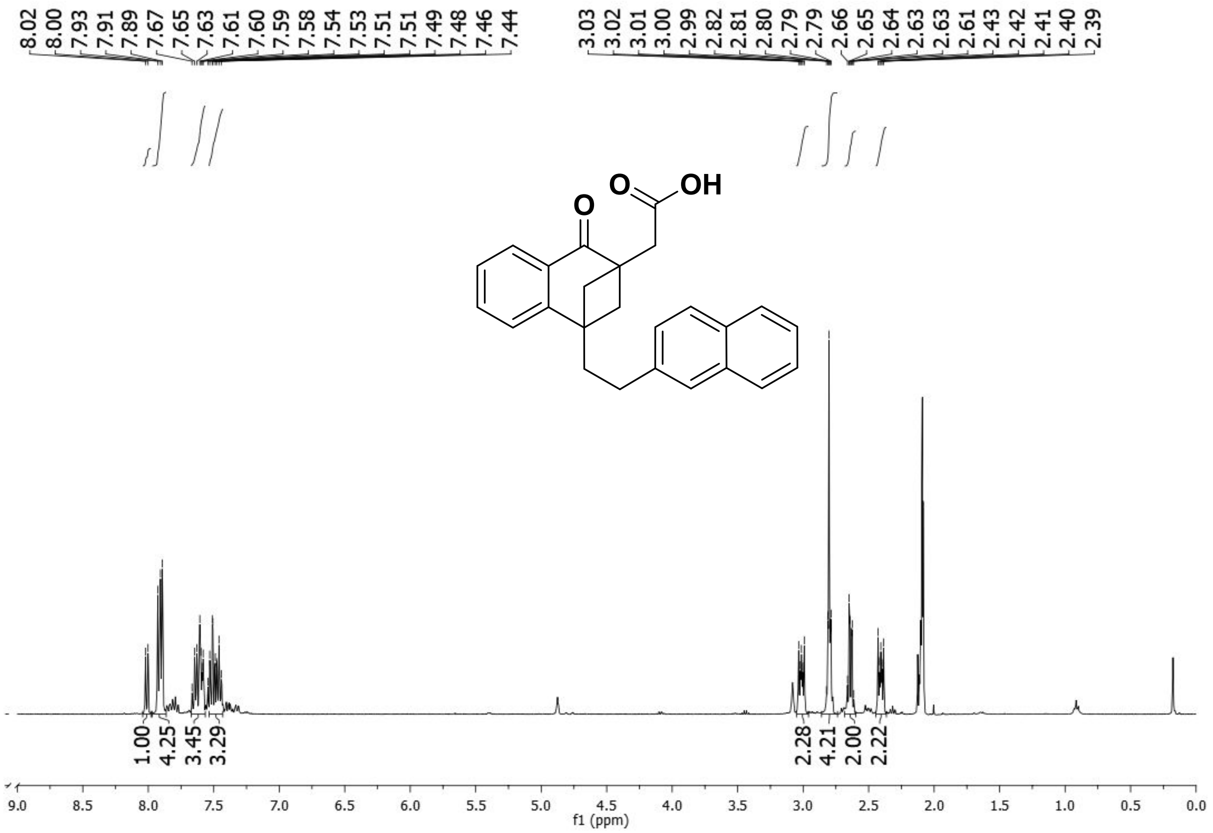

2p ^{13}CNMR (100 MHz, acetone-d$_6$)[27]

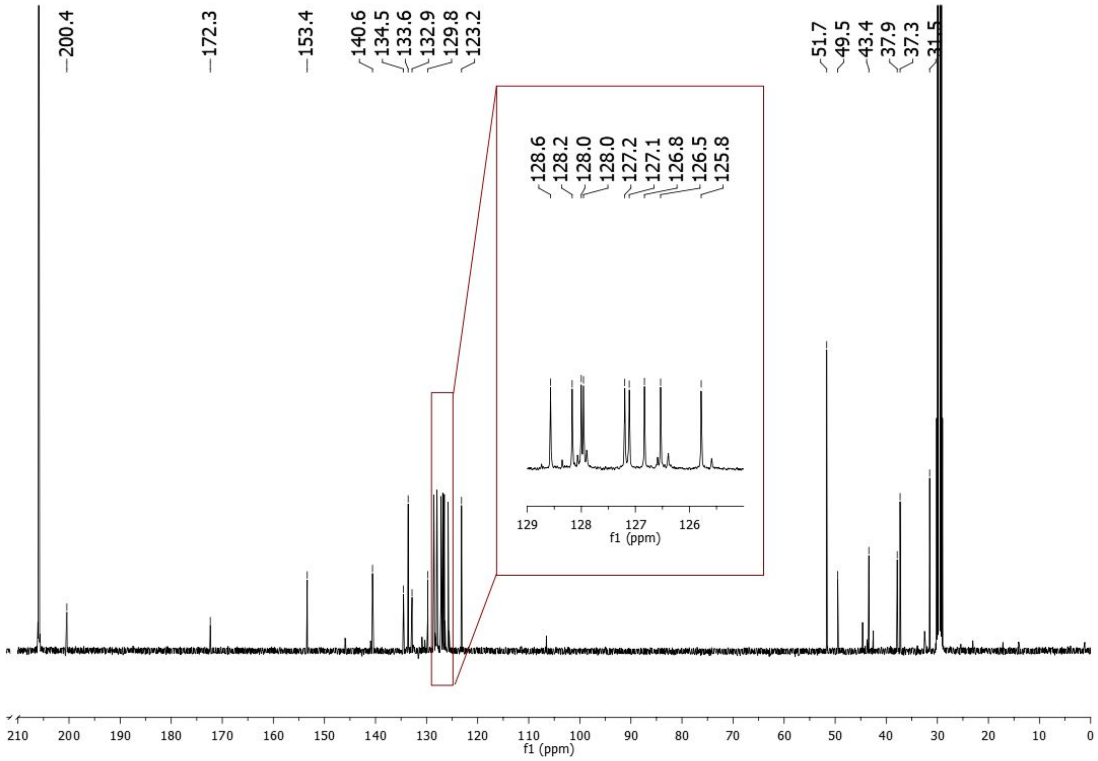

[27] For sake of clarity in ^{13}C NMR spectra signals between 129.0 and 125.0 ppm are reported only in the red window.

2q ^{1}HNMR (400 MHz, acetone-d_6)

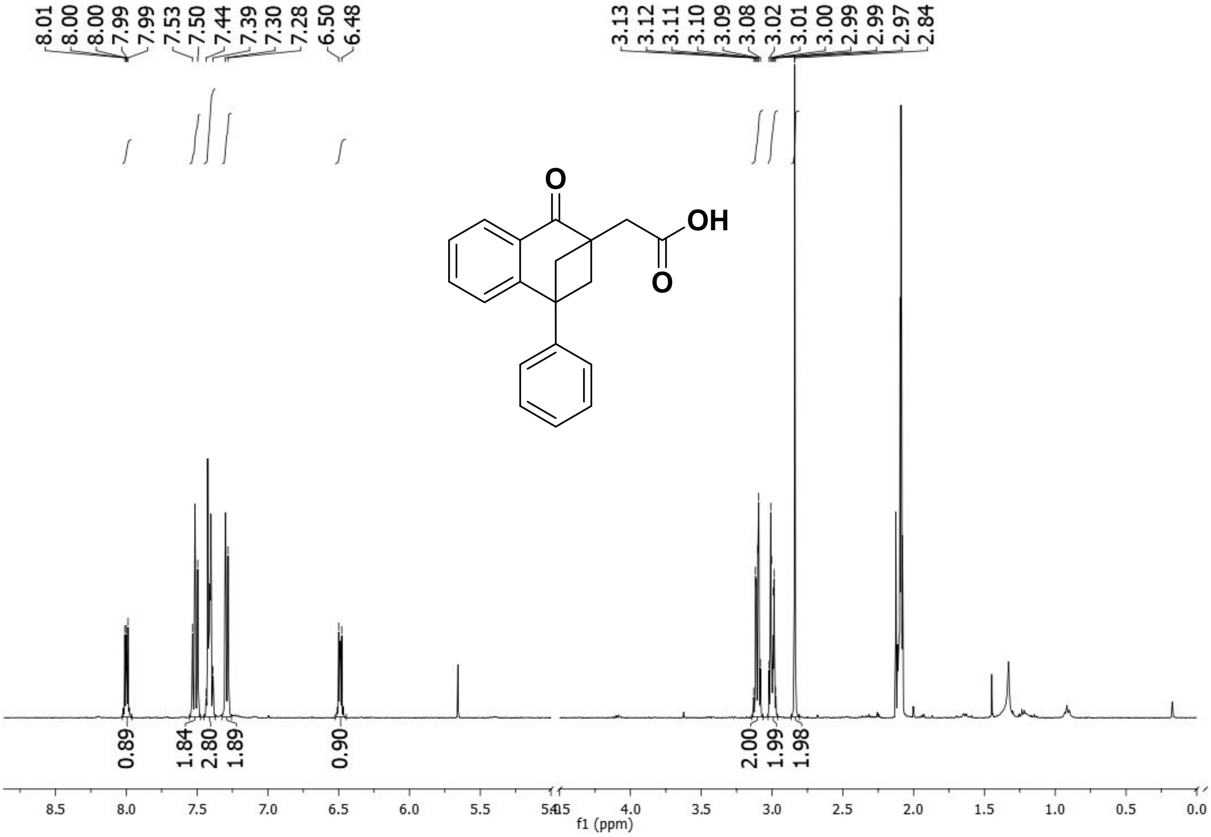

2q ^{13}CNMR (100 MHz, acetone-d_6)

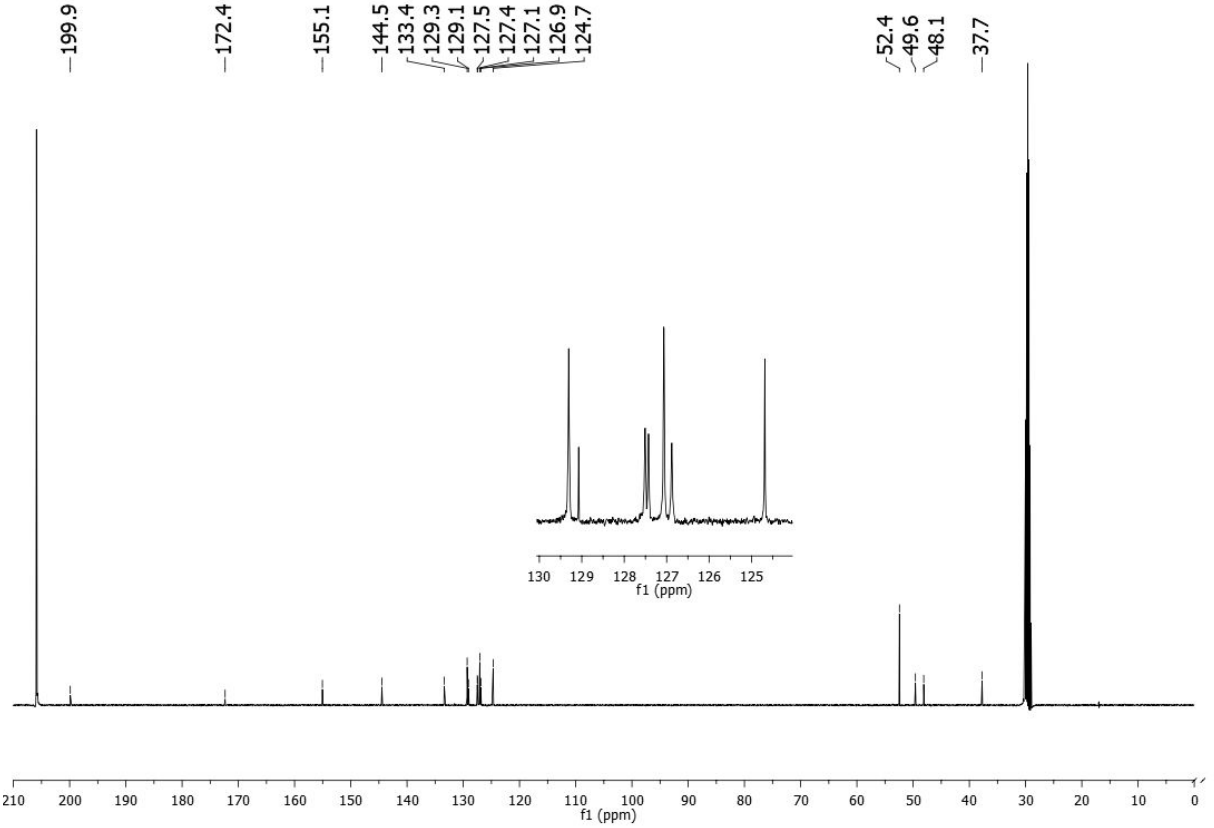

2r ^{1}HNMR (400 MHz, $CDCl_3$)[28]

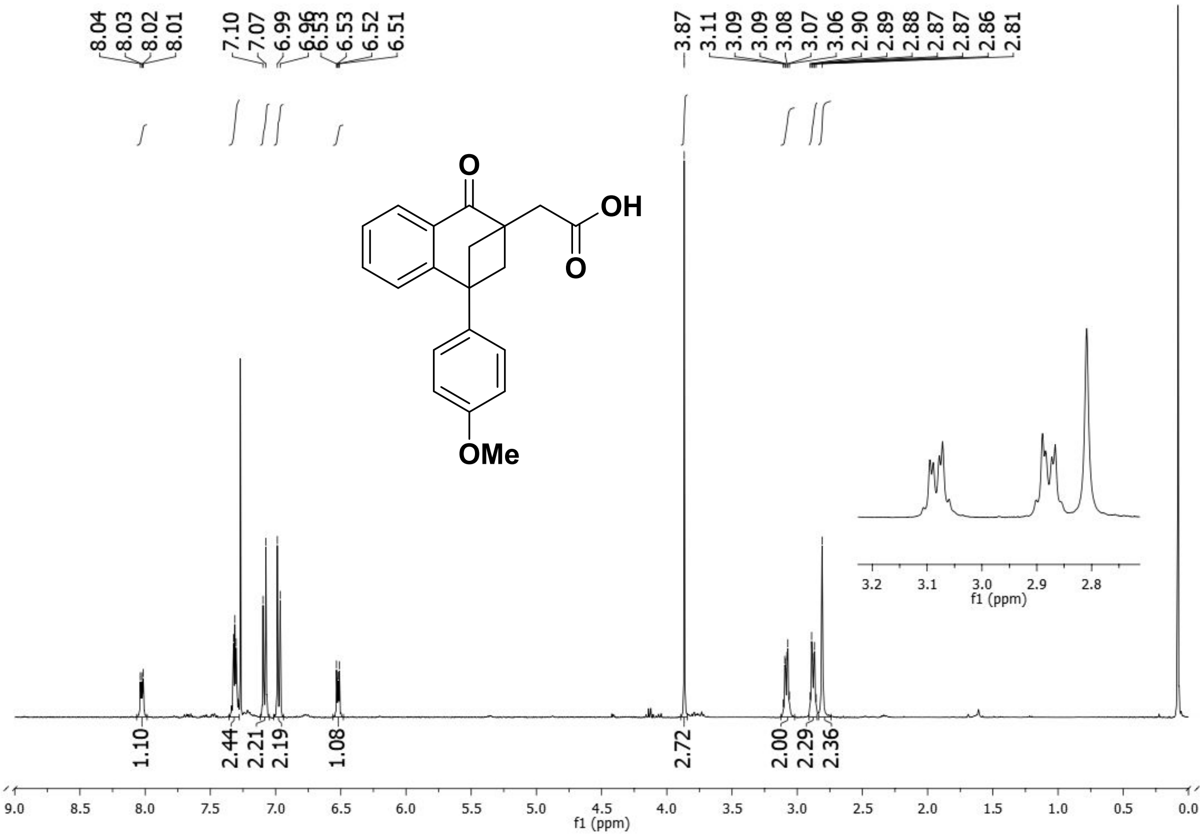

2r ^{13}CNMR (100 MHz, $CDCl_3$)

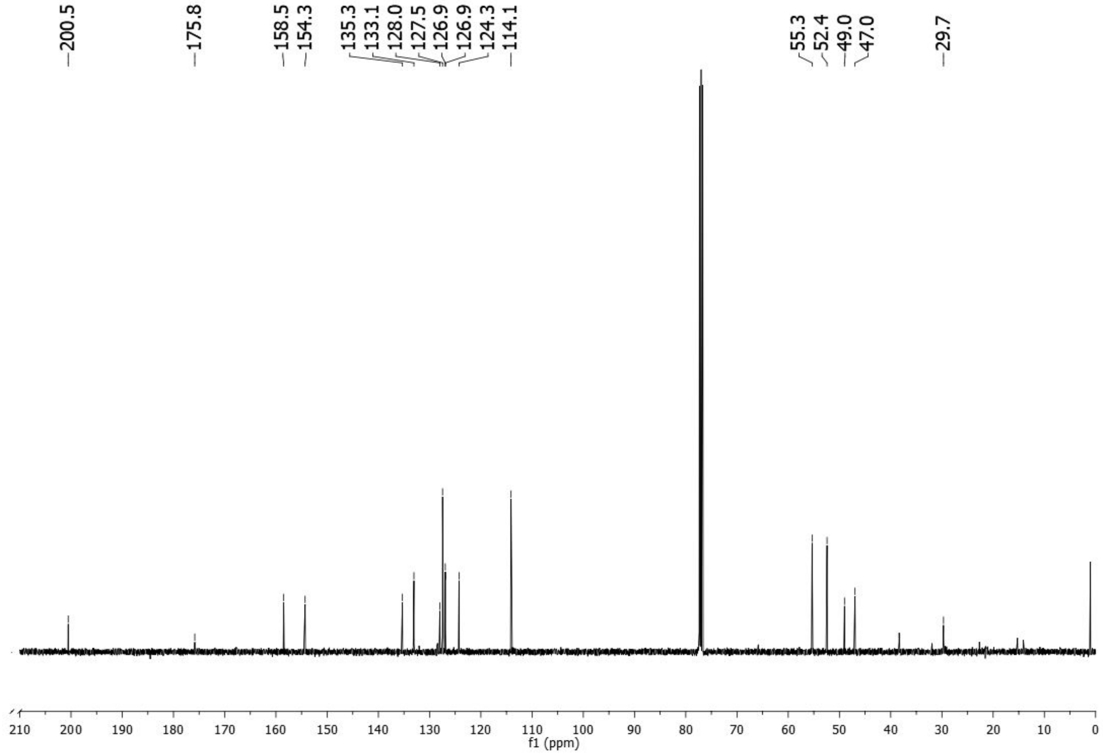

[28] One of few examples where ^{1}H NMR signals pattern of cyclobutane is more deshielded than that of α-CH_2.

3 ^{1}HNMR (400 MHz, CDCl$_3$)

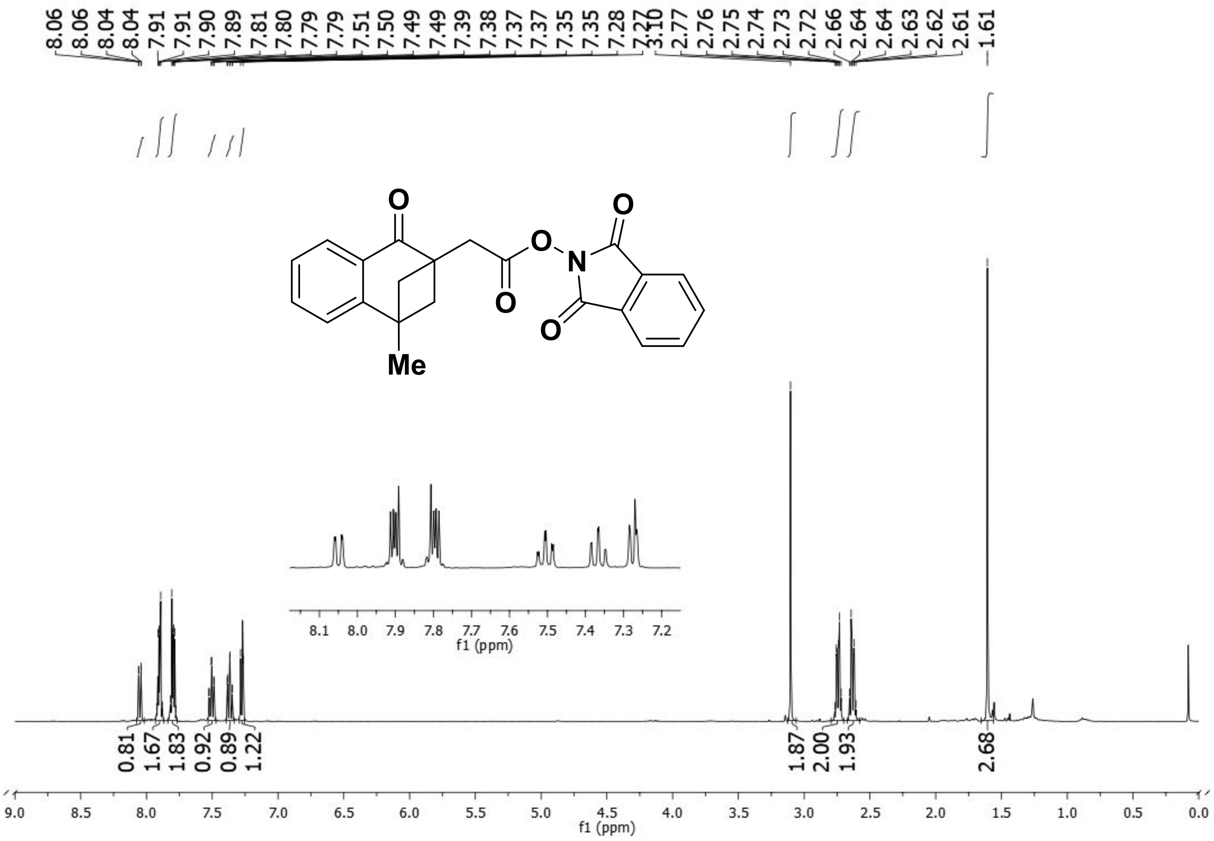

3 ^{13}CNMR (100 MHz, CDCl$_3$)

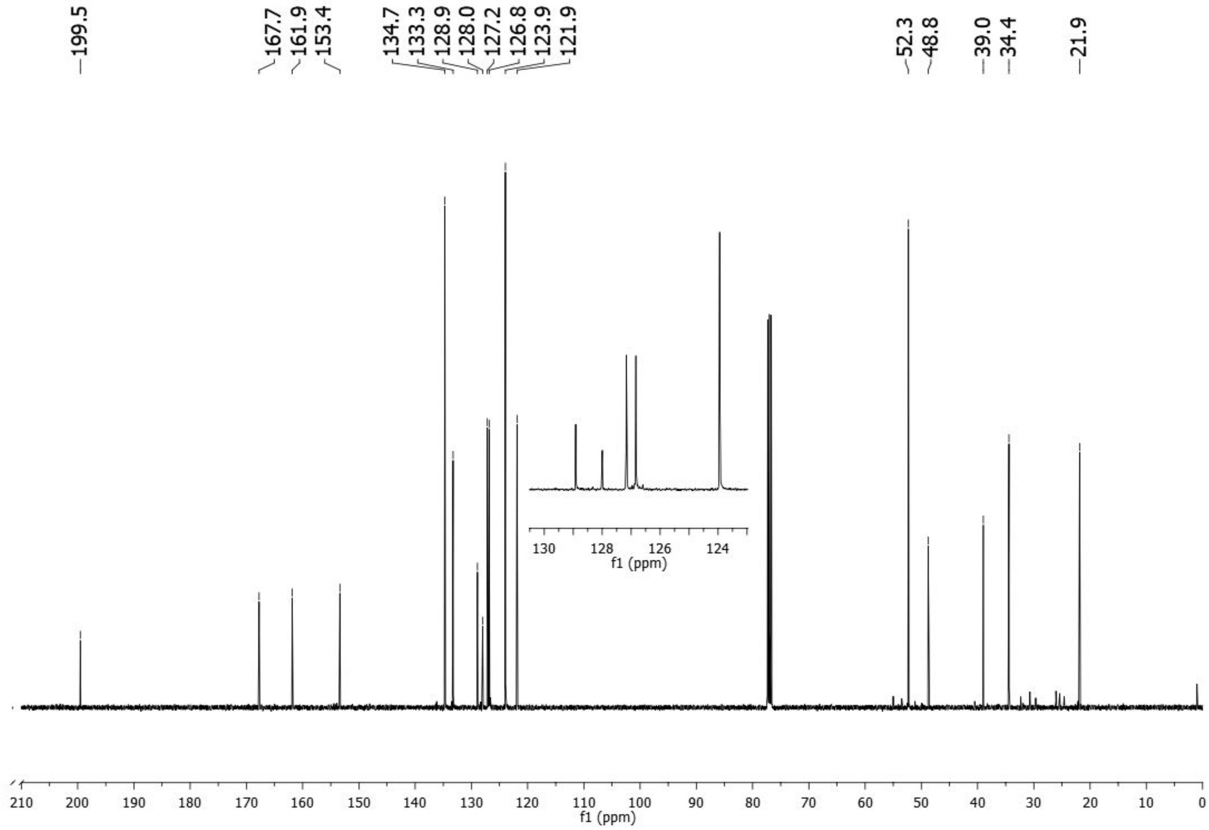

5 ^{1}HNMR (400 MHz, $CDCl_3$)

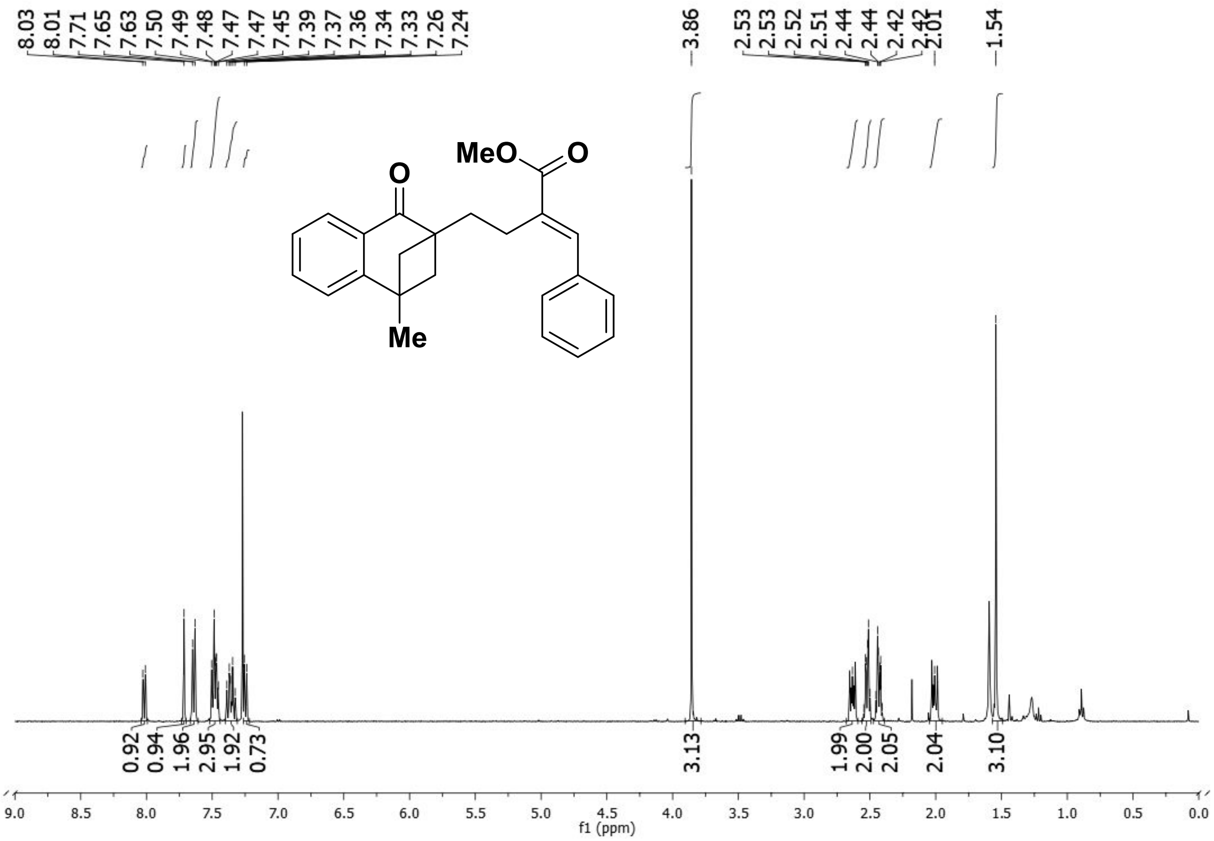

5 ^{13}CNMR (100 MHz, $CDCl_3$)

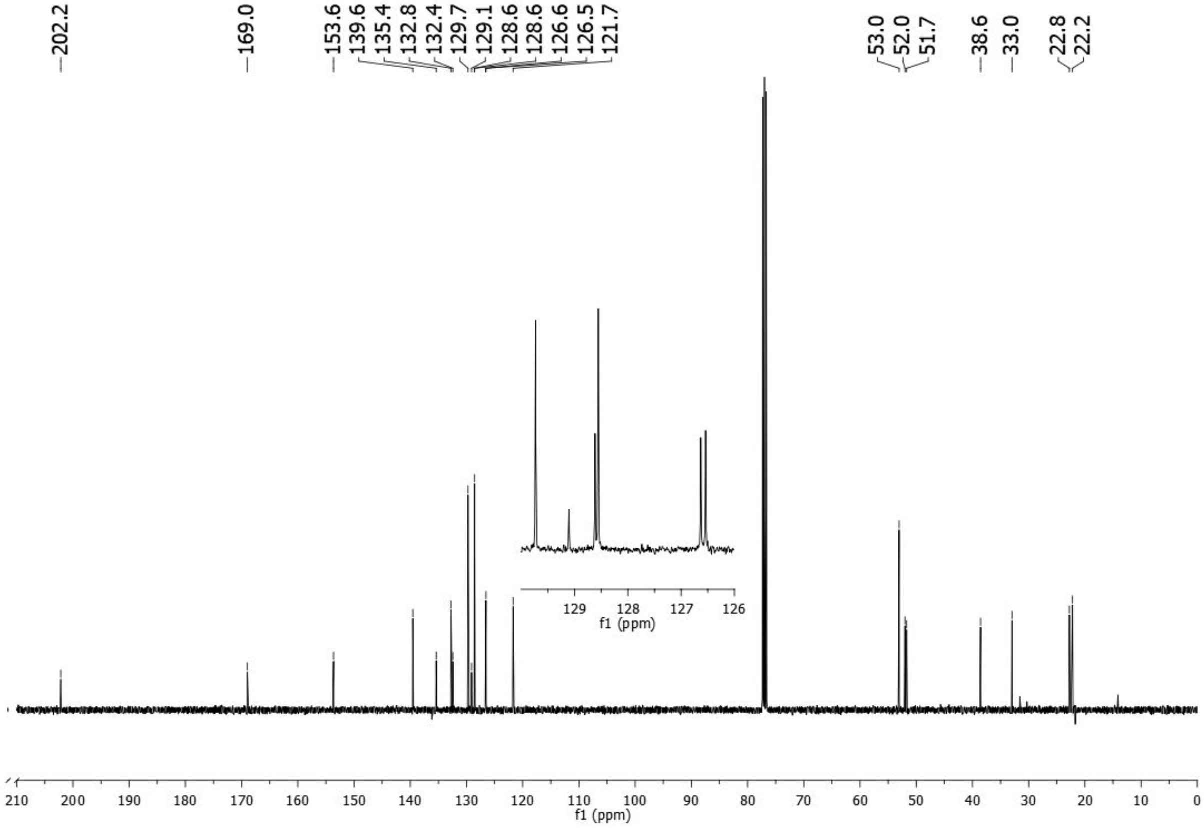

6 ^{1}HNMR (400 MHz, CDCl$_3$)

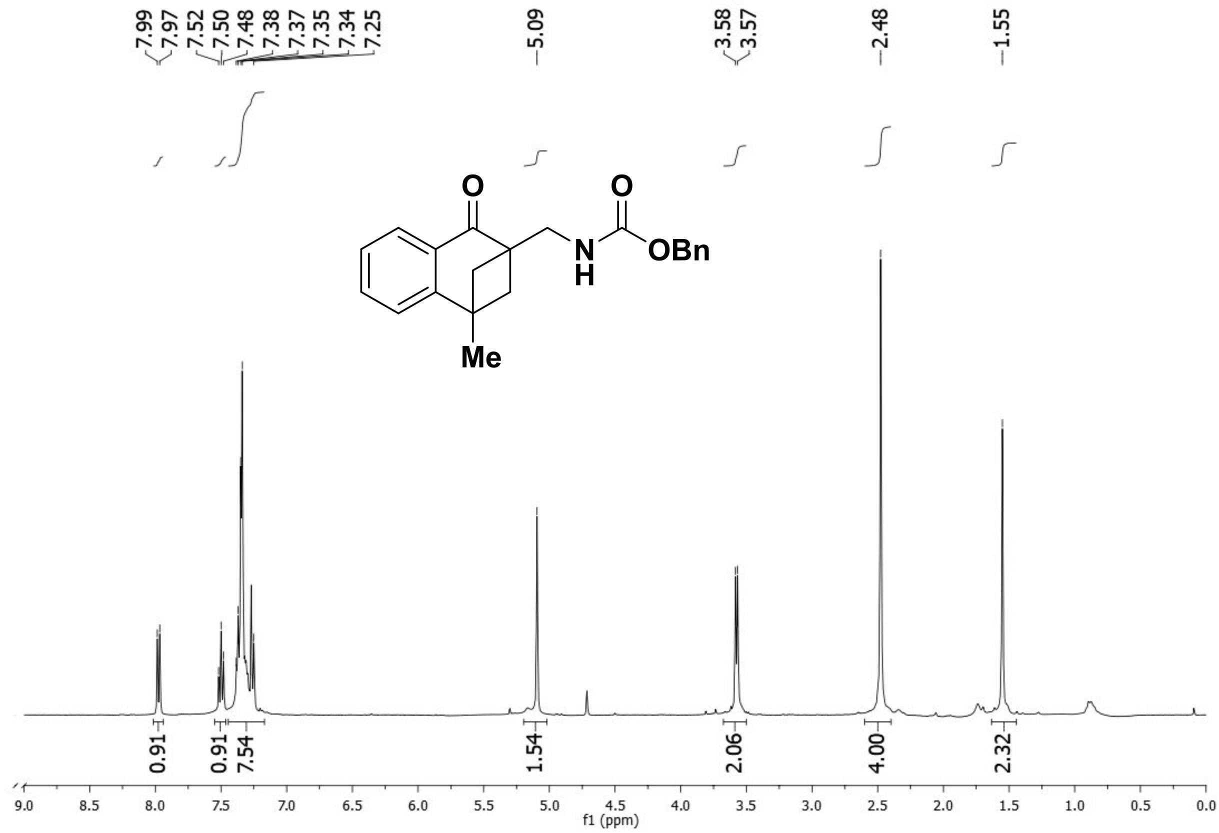

6 ^{13}CNMR (100 MHz, CDCl$_3$)

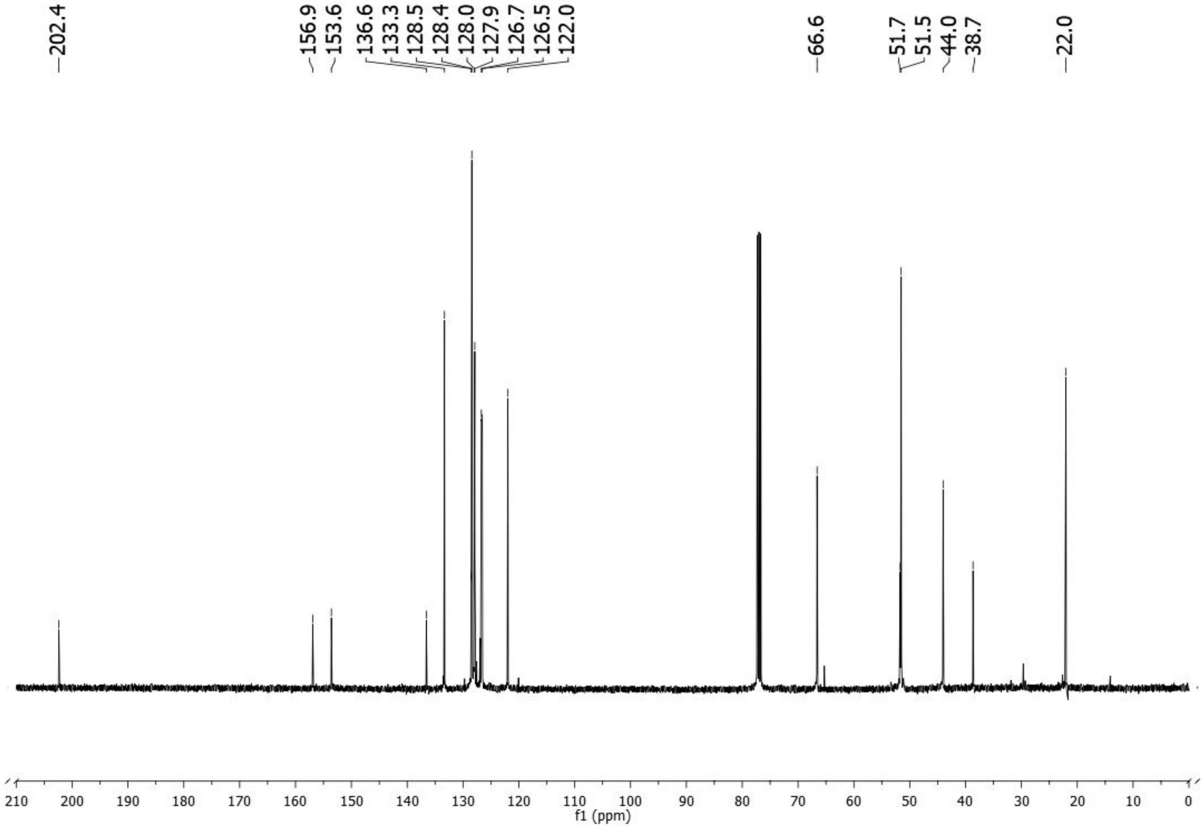

7 ^{1}HNMR (400 MHz, $CDCl_3$)

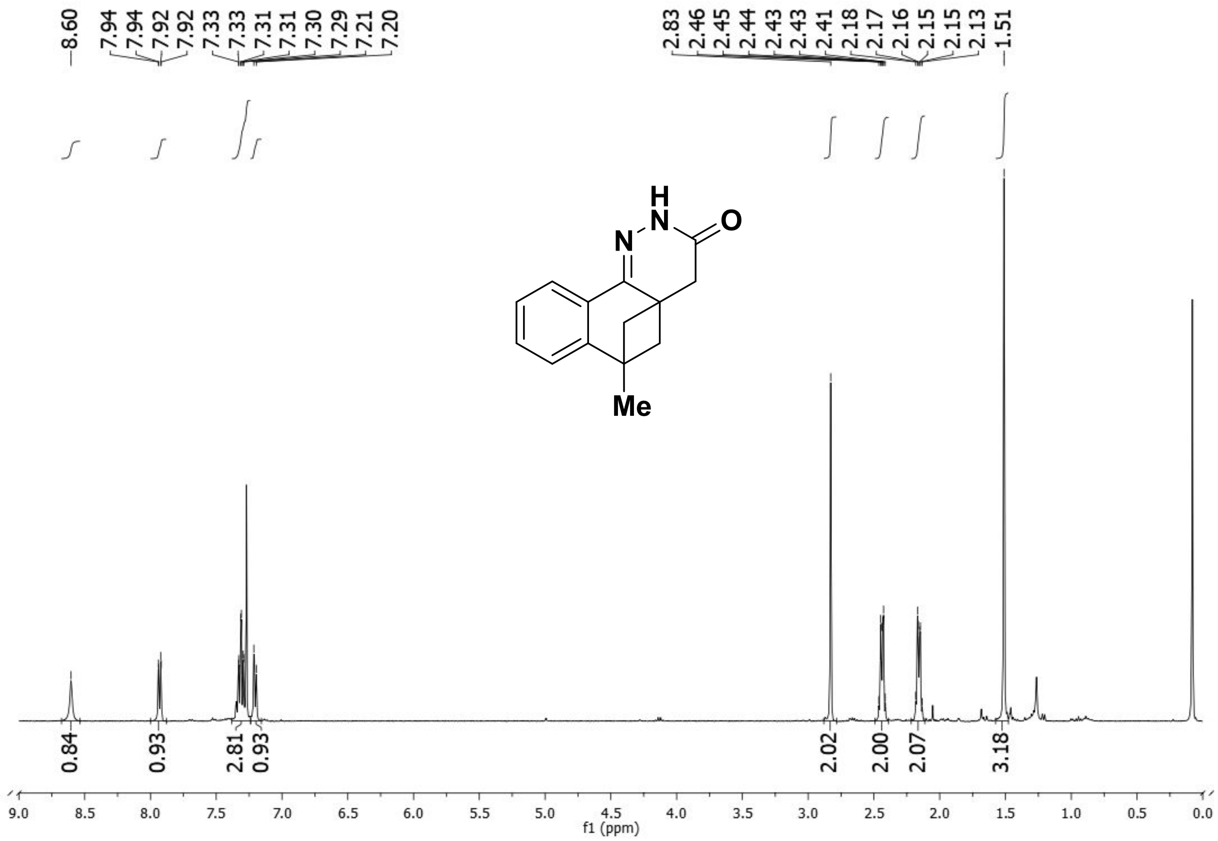

7 ^{13}CNMR (100 MHz, $CDCl_3$)

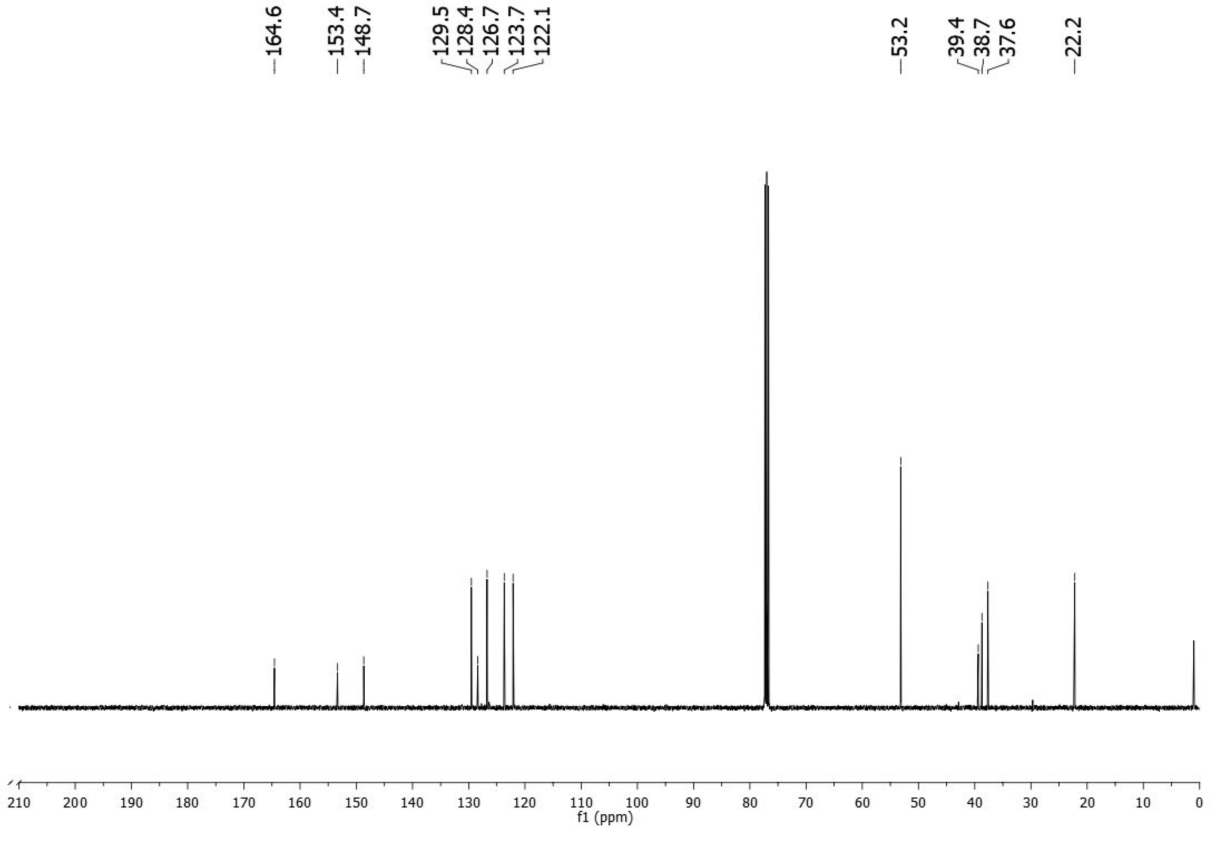

I1 ^{1}HNMR (400 MHz, $CDCl_3$)

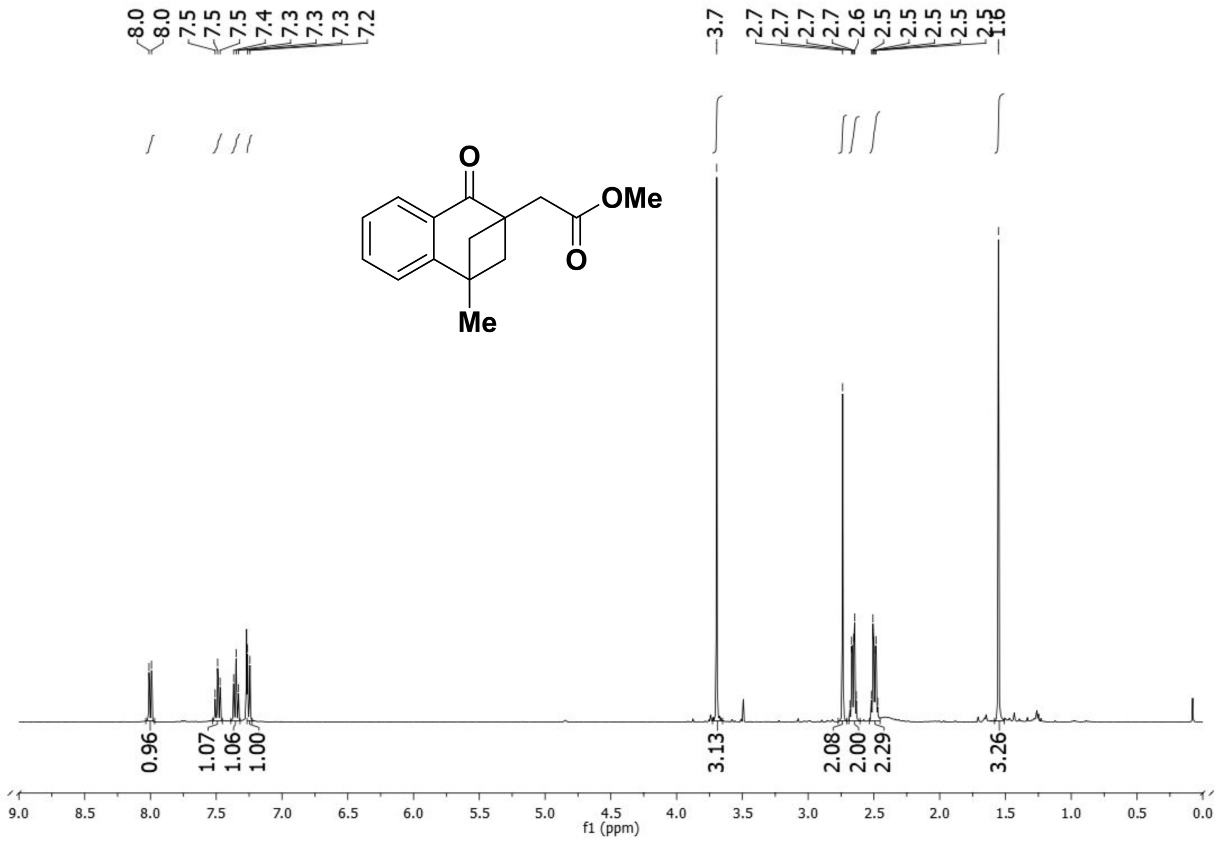

I1 ^{13}CNMR (100 MHz, $CDCl_3$)

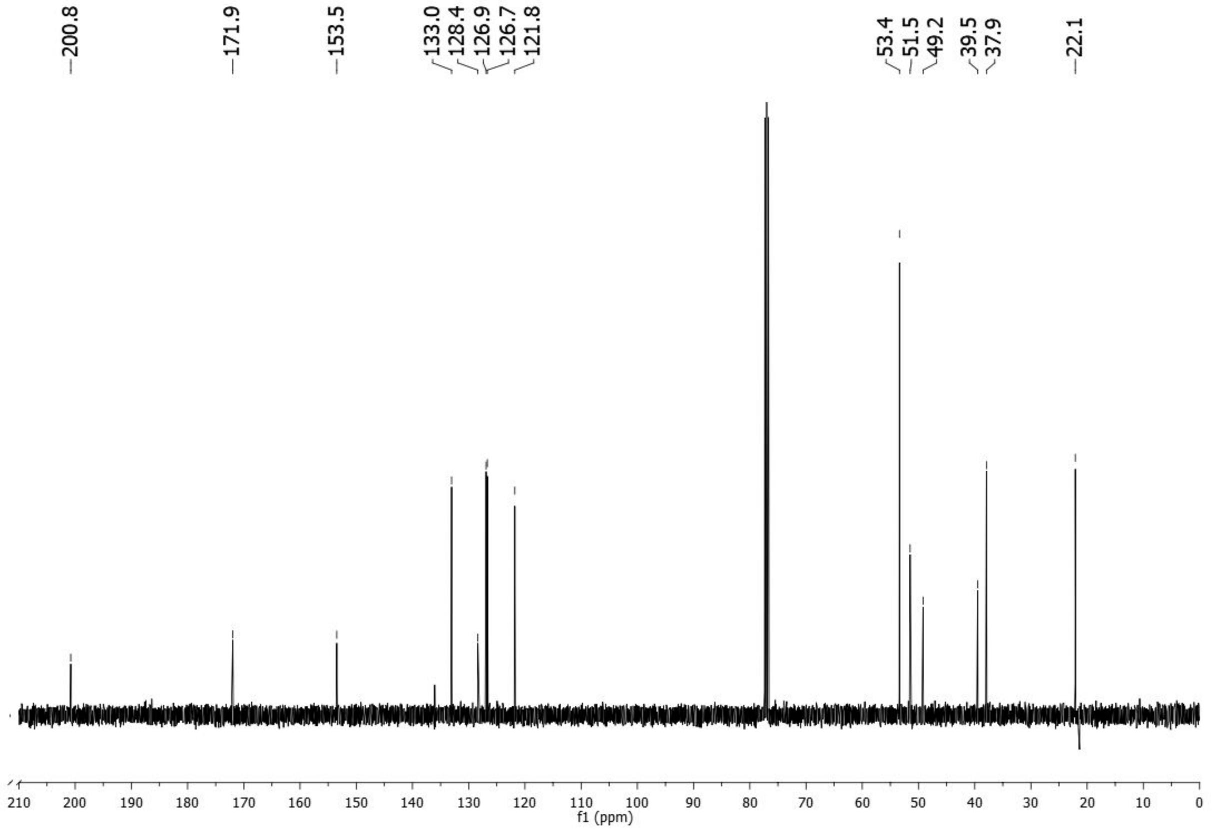

8 ^{1}HNMR (400 MHz, $CDCl_3$)

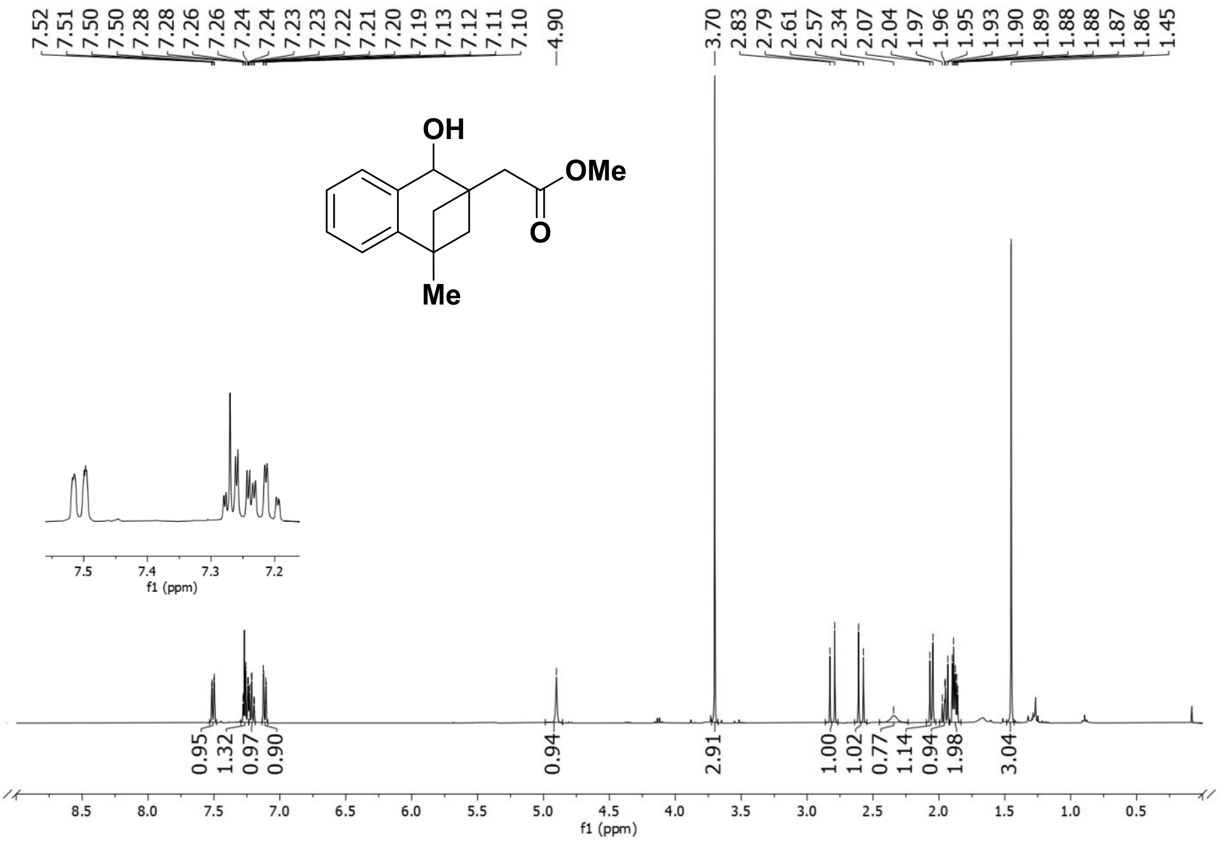

8 ^{13}CNMR (100 MHz, $CDCl_3$)

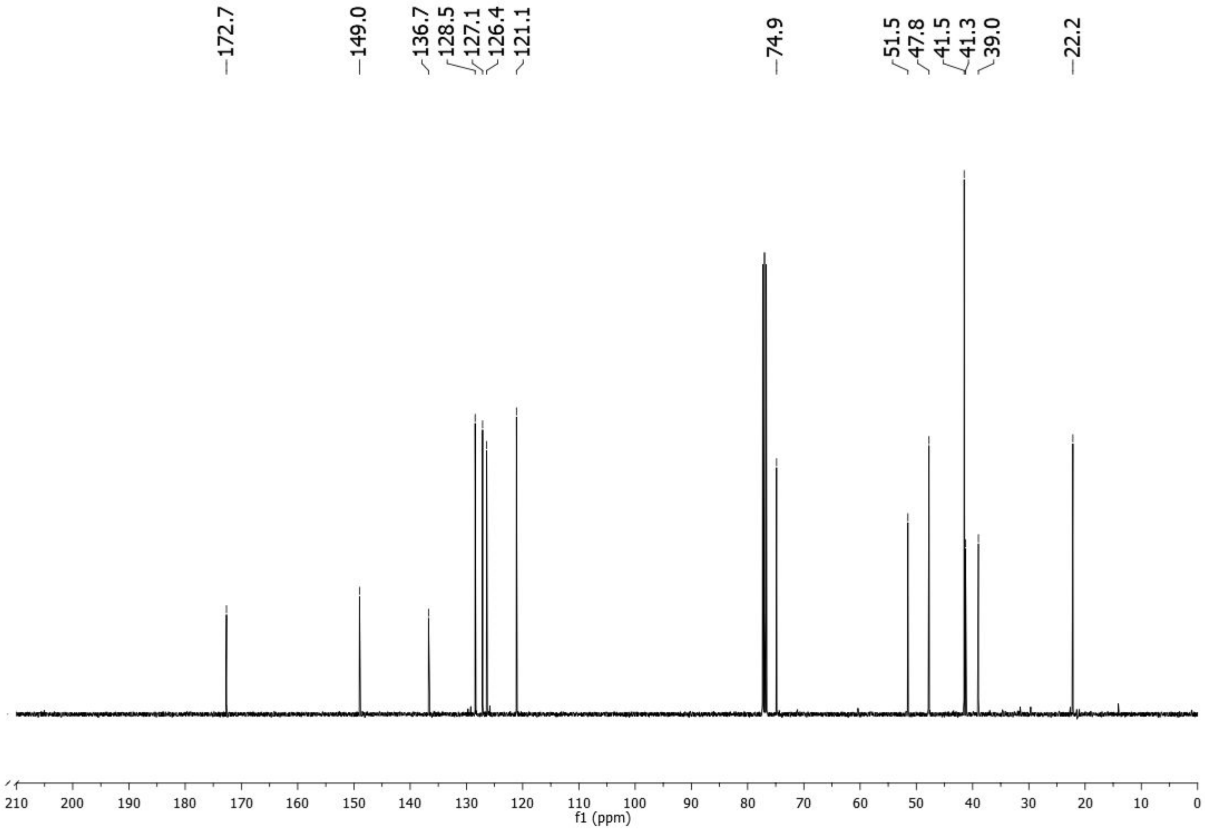

9 ^{1}HNMR (400 MHz, $CDCl_3$)

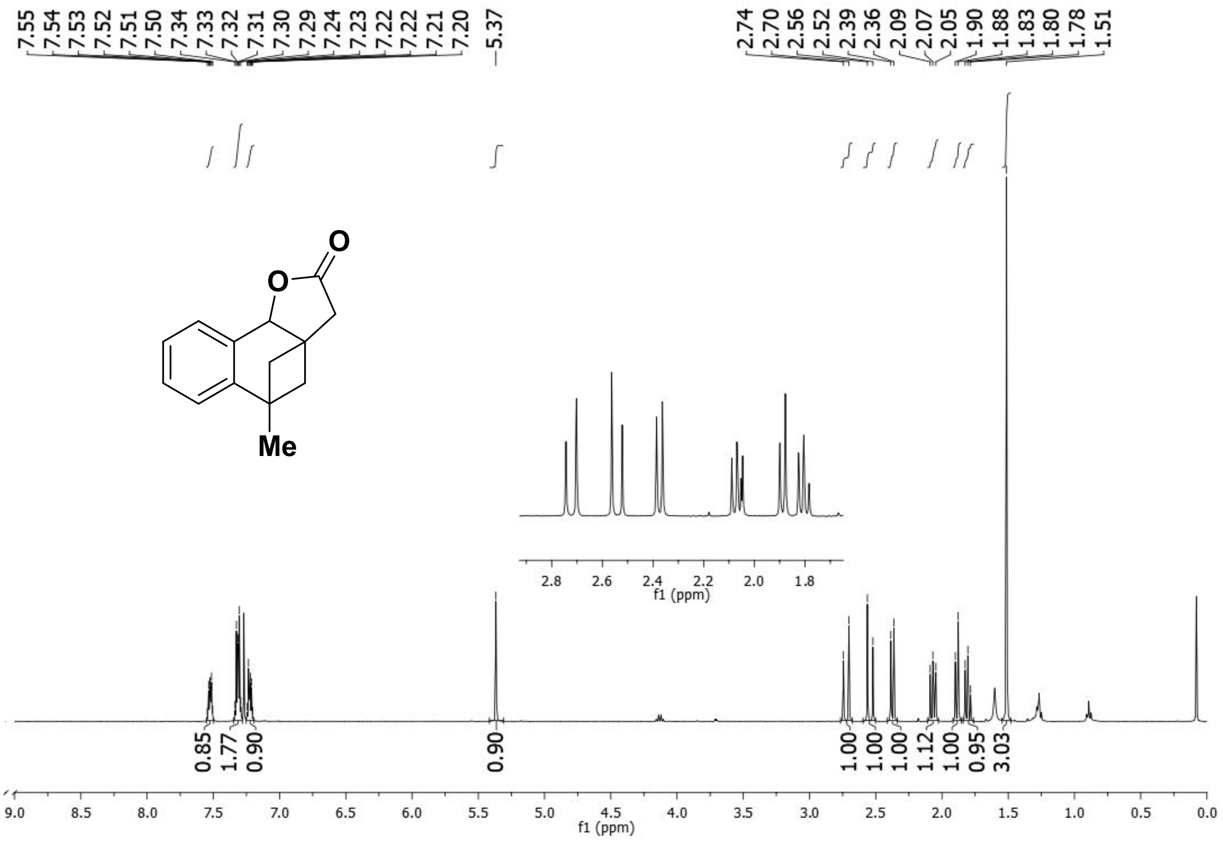

9 ^{13}CNMR (100 MHz, $CDCl_3$)

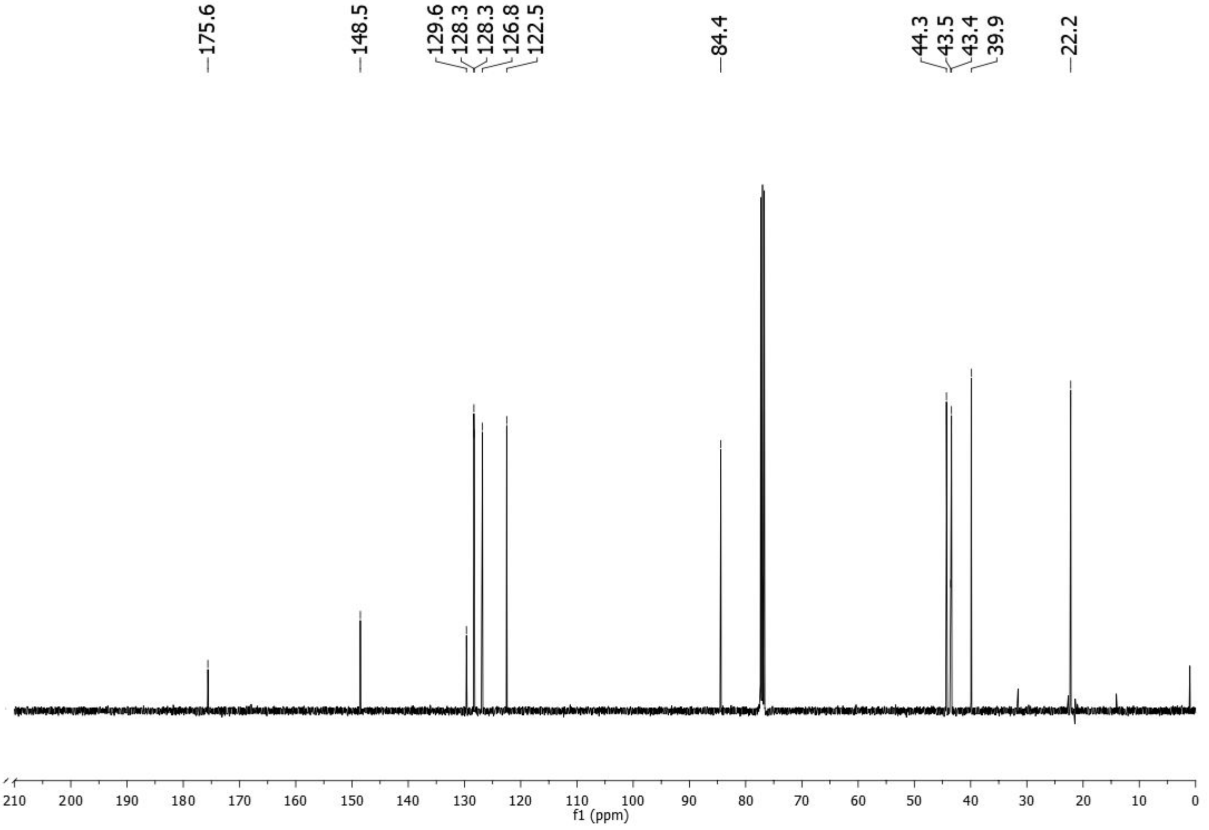

8. Cartesians

I-d

C	0.382422	0.716848	-0.071215
C	-1.240654	2.319409	-0.572531
C	-0.323796	3.353774	-0.436127
C	0.970618	3.054910	-0.021388
C	1.333348	1.722167	0.215616
C	0.578183	-0.738702	0.059805
C	-0.532382	-2.724120	0.584677
C	0.630081	-3.466003	0.426975
C	1.788381	-2.826529	-0.003411
C	1.764131	-1.445565	-0.236654
H	-2.294723	2.492499	-0.823222
H	-0.623918	4.390621	-0.617021
H	1.685212	3.865285	0.150058
H	-1.482753	-3.190510	0.868994
H	0.625199	-4.545229	0.608414
H	2.695609	-3.409352	-0.186810
N	-0.882607	1.048499	-0.391267
N	-0.551491	-1.400071	0.397447
O	2.495183	1.351252	0.756912
O	2.772584	-0.764510	-0.783702
C	3.488252	2.317118	1.002195
H	3.788038	2.832766	0.069679
H	3.150334	3.066757	1.743764
H	4.356574	1.776880	1.408705
C	4.002397	-1.407425	-1.014793
H	4.685783	-0.642219	-1.412974
H	4.425124	-1.816910	-0.077289
H	3.901069	-2.221902	-1.757985
Ni	-2.175697	-0.387004	0.070698
Cl	-4.302433	0.044071	-0.081275

I-q

C	-0.352012	0.693301	0.018794
C	1.370106	2.295416	0.389966
C	0.479049	3.343708	0.376645
C	-0.870104	3.071724	0.041291
C	-1.279722	1.769096	-0.188360
C	-0.599992	-0.715621	-0.056784
C	0.445512	-2.812352	-0.484897
C	-0.754164	-3.481657	-0.462750
C	-1.915335	-2.764881	-0.073868
C	-1.835906	-1.406654	0.179175
H	2.447442	2.440732	0.542232
H	0.818689	4.369193	0.550133
H	-1.565362	3.904683	-0.099472
H	1.392762	-3.332540	-0.677094
H	-0.798953	-4.555553	-0.666875
H	-2.851918	-3.306945	0.087048
N	0.973969	1.026636	0.200205
N	0.532304	-1.487096	-0.263438
O	-2.482886	1.445883	-0.685066
O	-2.833861	-0.692845	0.721633
C	-3.461642	2.439652	-0.819325
H	-3.676884	2.930859	0.150326
H	-3.163651	3.213192	-1.555319
H	-4.374799	1.939088	-1.178125
C	-4.089697	-1.289616	0.895007
H	-4.759296	-0.510488	1.292194
H	-4.498591	-1.661663	-0.065331
H	-4.048571	-2.129924	1.616912
Ni	2.185001	-0.503840	0.031617
Cl	4.298388	0.067831	0.022284

II

C	1.165358	0.318100	-0.096337
C	2.179329	1.207386	-0.452595
C	-0.103305	0.754427	0.332726
C	1.961877	2.580093	-0.379386

H	3.146619	0.816118	-0.786216
C	-0.274626	2.144592	0.407135
C	0.727851	3.047517	0.060304
H	2.760315	3.275320	-0.661216
H	-1.230741	2.544734	0.758466
H	0.536614	4.123650	0.136427
C	-1.209029	-0.201257	0.752328
C	-2.652460	0.390151	0.692554
C	-1.641515	-1.159564	-0.411035
C	-2.830661	-0.267301	-0.647586
H	-2.820282	1.478258	0.767186
H	-3.276674	-0.095082	1.469222
H	-0.947044	-1.304128	-1.257414
H	-1.921551	-2.157468	-0.017505
C	-0.881859	-0.837527	2.097122
H	0.095345	-1.353467	2.098403
H	-0.857992	-0.060418	2.886017
H	-1.657770	-1.577406	2.372670
C	-3.605191	-0.022305	-1.701532
H	-3.499088	-0.595047	-2.633408
H	-4.373808	0.762650	-1.675100
Br	1.629470	-1.530959	-0.210849

III-d

C	-1.825833	-0.914198	-0.529332
C	-1.744994	-2.665777	1.012927
C	-3.095480	-2.527312	1.305471
C	-3.848718	-1.600779	0.588092
C	-3.227088	-0.802555	-0.380556
C	-0.978783	-0.128606	-1.443439
C	0.909462	-0.259049	-2.804499
C	0.701836	1.026364	-3.285805
C	-0.329758	1.791549	-2.751327
C	-1.159120	1.232298	-1.772641
H	-1.106173	-3.418627	1.493045
H	-3.568395	-3.159912	2.063036

H	-4.925544	-1.525234	0.765153
H	1.754564	-0.872550	-3.139041
H	1.369356	1.445550	-4.045449
H	-0.457361	2.831036	-3.067597
N	-1.149117	-1.879757	0.115780
N	0.092264	-0.812725	-1.903527
O	-3.881632	0.010793	-1.211963
O	-2.068885	1.922993	-1.078830
C	-5.268072	0.193379	-1.058068
H	-5.821918	-0.751571	-1.220352
H	-5.512956	0.594298	-0.055683
H	-5.574441	0.924048	-1.821923
C	-2.382001	3.239160	-1.464710
H	-3.197833	3.573922	-0.806112
H	-1.513858	3.914969	-1.337403
H	-2.726267	3.277080	-2.516179
Ni	0.596763	-2.292186	-0.725525
Cl	1.720845	-3.965175	0.128963
Br	3.186449	-0.364070	0.047404
C	2.295158	1.325307	0.103950
C	2.771291	2.219810	-0.865909
C	1.253556	1.668385	0.987018
C	2.224650	3.485946	-1.002615
H	3.586509	1.899751	-1.524283
C	0.714992	2.959997	0.792001
C	1.174479	3.853991	-0.164202
H	2.612732	4.173504	-1.762254
H	-0.104912	3.290504	1.441297
H	0.715156	4.846082	-0.245500
C	0.658012	0.879702	2.150629
C	0.647010	1.682633	3.499513
C	-0.909815	0.878979	2.204317
C	-0.841969	1.456123	3.597146
H	0.930441	2.746837	3.397825
H	1.269741	1.245122	4.303775
H	-1.398013	1.532415	1.456726
H	-1.369211	-0.124753	2.119620

C	1.160127	-0.528521	2.426790
H	0.997921	-1.215766	1.575529
H	2.231519	-0.547134	2.696934
H	0.596049	-0.930772	3.292210
C	-1.737313	1.626685	4.567321
H	-2.792770	1.355415	4.424767
H	-1.451470	2.043449	5.543197

III-q

C	-0.626134	1.847330	0.104595
C	0.745635	2.582400	-1.696568
C	-0.335795	3.055697	-2.404633
C	-1.603110	3.057270	-1.768291
C	-1.747301	2.503825	-0.507727
C	-0.597481	1.103902	1.327919
C	0.817031	0.439742	3.118300
C	-0.236065	-0.069937	3.841849
C	-1.503429	-0.145767	3.212056
C	-1.678141	0.387818	1.947163
H	1.772979	2.638187	-2.081442
H	-0.207286	3.488780	-3.401106
H	-2.448016	3.551702	-2.257339
H	1.845003	0.418185	3.504904
H	-0.079260	-0.482525	4.843135
H	-2.319152	-0.681400	3.707209
N	0.609392	2.032736	-0.480208
N	0.656555	0.975730	1.896296
O	-2.863219	2.611555	0.230526
O	-2.782789	0.210028	1.208134
C	-3.996826	3.221711	-0.321913
H	-3.806841	4.283594	-0.578036
H	-4.344886	2.691692	-1.231111
H	-4.787538	3.173838	0.443246
C	-3.847615	-0.533694	1.729728
H	-4.624430	-0.563210	0.948973
H	-3.539826	-1.571542	1.972172

H	-4.269033	-0.063304	2.640657
Ni	2.099043	1.411731	0.642096
Cl	4.045748	1.908994	-0.298883
Br	2.227103	-1.008502	-0.584921
C	0.892081	-2.156270	0.179297
C	1.080512	-2.447407	1.528168
C	-0.184212	-2.666620	-0.571358
C	0.176190	-3.271577	2.190908
H	1.939073	-2.024849	2.061217
C	-1.062701	-3.502034	0.133463
C	-0.898344	-3.800701	1.485109
H	0.318930	-3.492582	3.254520
H	-1.911547	-3.947254	-0.394087
H	-1.619363	-4.458250	1.983488
C	-0.367428	-2.386291	-2.054377
C	-1.808231	-2.637004	-2.596248
C	-0.623162	-0.862305	-2.322333
C	-2.094723	-1.175736	-2.393887
H	-2.472596	-3.380413	-2.123055
H	-1.743107	-2.888936	-3.673668
H	-0.298720	-0.131540	-1.559639
H	-0.201019	-0.558313	-3.302116
C	0.705454	-3.086309	-2.878110
H	1.733256	-2.830527	-2.563846
H	0.590195	-4.184345	-2.790643
H	0.603444	-2.818303	-3.947049
C	-3.202044	-0.478394	-2.152750
H	-3.157438	0.595977	-1.922119
H	-4.190881	-0.958263	-2.163760

TS-III-IV-d

C	-2.205588	-0.902415	-0.300770
C	-0.564506	-2.548940	-0.268935
C	-1.516797	-3.560276	-0.210367
C	-2.861782	-3.223198	-0.298790
C	-3.227268	-1.875298	-0.398448

C	-2.445807	0.554936	-0.286353
C	-1.641238	2.610729	-1.012585
C	-2.763234	3.273335	-0.527206
C	-3.703907	2.553765	0.199675
C	-3.532772	1.174381	0.371647
H	0.509210	-2.766755	-0.284379
H	-1.208493	-4.607519	-0.136510
H	-3.620404	-4.011207	-0.321558
H	-0.825999	3.149670	-1.509652
H	-2.879858	4.350629	-0.678527
H	-4.555646	3.071445	0.650709
N	-0.915037	-1.267013	-0.316635
N	-1.491720	1.294930	-0.873239
O	-4.467826	-1.451706	-0.642137
O	-4.291220	0.419540	1.166539
C	-5.538078	-2.364869	-0.600859
H	-5.453060	-3.125923	-1.400259
H	-5.596418	-2.869083	0.382898
H	-6.455817	-1.778878	-0.760767
C	-5.468358	0.955103	1.722589
H	-5.958005	0.134610	2.268676
H	-5.245118	1.775937	2.431099
H	-6.151586	1.324353	0.933836
Ni	0.373925	0.256396	-0.897455
Cl	1.371638	-0.832018	-2.651620
Br	1.802183	2.228899	-0.576967
C	1.250140	0.649378	0.870540
C	0.257029	1.145580	1.738504
C	2.200226	-0.293410	1.321271
C	0.057906	0.579418	2.987387
H	-0.389019	1.966888	1.407739
C	1.956890	-0.840336	2.597228
C	0.907232	-0.444383	3.415306
H	-0.751523	0.946659	3.629046
H	2.646182	-1.615400	2.958852
H	0.773834	-0.907344	4.399334
C	3.421999	-0.855991	0.616333

C	4.239587	-0.023110	-0.409974
C	4.717233	-0.907612	1.489297
C	5.490114	-0.546585	0.246548
H	4.139314	1.060947	-0.223200
H	4.049565	-0.217083	-1.482319
H	4.733060	-0.086654	2.234260
H	4.956601	-1.860706	2.000140
C	3.149464	-2.250859	0.057851
H	2.754277	-2.925345	0.842471
H	2.434159	-2.197159	-0.782372
H	4.087110	-2.696790	-0.329028
C	6.739911	-0.759056	-0.158517
H	7.481898	-1.229946	0.501511
H	7.070863	-0.471397	-1.166218

TS-III-IV-q

C	2.689869	-0.793599	0.325260
C	1.093312	-2.445486	0.795157
C	2.066211	-3.438998	0.826538
C	3.396771	-3.075107	0.676963
C	3.739425	-1.727666	0.487765
C	2.844877	0.648249	0.045675
C	1.849749	2.739158	0.413889
C	2.938600	3.400813	-0.144998
C	3.950386	2.649463	-0.724479
C	3.890373	1.247233	-0.694804
H	0.033163	-2.655192	0.980954
H	1.787364	-4.482086	1.001938
H	4.179428	-3.835973	0.753046
H	0.977651	3.275730	0.806356
H	2.971788	4.493588	-0.165601
H	4.778188	3.155165	-1.229827
N	1.418495	-1.182586	0.538875
N	1.823898	1.411487	0.491169
O	4.988275	-1.276164	0.513925
O	4.715151	0.457202	-1.371068

C	6.064961	-2.171523	0.335280
H	6.191558	-2.836792	1.209566
H	5.923815	-2.781582	-0.577030
H	6.968898	-1.554549	0.221664
C	5.837836	1.002750	-2.030235
H	6.389175	0.152050	-2.457752
H	5.532227	1.683945	-2.847317
H	6.494923	1.541839	-1.321469
Ni	0.128143	0.340762	0.730953
Cl	-0.594652	0.157009	2.790373
Br	-3.446289	3.010960	0.275047
C	-2.147854	0.428325	-0.723439
C	-0.977281	1.025836	-1.182008
C	-2.478319	-0.899761	-0.940047
C	-0.066784	0.235055	-1.912181
H	-0.821178	2.107194	-1.052009
C	-1.557489	-1.660020	-1.690505
C	-0.372043	-1.105571	-2.167733
H	0.858625	0.681409	-2.299236
H	-1.780966	-2.717346	-1.892466
H	0.329107	-1.723059	-2.739729
C	-3.716099	-1.540888	-0.374742
C	-4.987412	-0.658945	-0.247362
C	-4.519319	-2.479494	-1.320198
C	-5.775902	-1.819991	-0.804639
H	-4.950809	0.208546	-0.935730
H	-5.246619	-0.284144	0.761282
H	-4.333959	-2.240591	-2.386874
H	-4.404957	-3.571433	-1.175659
C	-3.381744	-2.231469	0.942245
H	-2.672025	-3.067120	0.779232
H	-2.919487	-1.518338	1.653097
H	-4.296699	-2.651228	1.405312
C	-7.046814	-2.207362	-0.724043
H	-7.379503	-3.162326	-1.155122
H	-7.804530	-1.588449	-0.223595

IV-d

C	1.767227	0.978492	-0.431329
C	-0.097256	2.081814	-1.292885
C	0.637438	3.236233	-1.528386
C	2.005184	3.222604	-1.294431
C	2.604445	2.061803	-0.791364
C	2.250174	-0.277548	0.166881
C	1.931660	-2.585527	0.173600
C	3.074077	-2.780481	0.944599
C	3.751721	-1.671861	1.434433
C	3.314792	-0.384974	1.090795
H	-1.158632	2.024171	-1.552755
H	0.146822	4.124430	-1.936569
H	2.608815	4.101305	-1.538856
H	1.294208	-3.415823	-0.152828
H	3.402191	-3.792347	1.199925
H	4.604735	-1.808574	2.105630
N	0.463674	0.999897	-0.758559
N	1.543429	-1.363898	-0.178083
O	3.918638	1.886105	-0.692037
O	3.792681	0.734788	1.625729
C	4.793135	2.957939	-0.961444
H	4.721388	3.280028	-2.017904
H	4.588308	3.818410	-0.296758
H	5.809980	2.585414	-0.767217
C	4.950659	0.689756	2.427504
H	5.196182	1.732206	2.679755
H	4.772717	0.124843	3.362370
H	5.798731	0.238718	1.878061
Ni	-0.366602	-0.833345	-0.805518
Cl	0.156717	-0.920431	-3.041669
Br	-1.420604	-2.947373	-0.665936
C	-1.157616	-0.480561	0.960655
C	-0.442463	-0.912313	2.079758
C	-2.328378	0.285124	1.101975
C	-0.824166	-0.537836	3.365683

H	0.429282	-1.566538	1.969819
C	-2.672505	0.664715	2.417411
C	-1.941512	0.275837	3.531687
H	-0.244874	-0.885202	4.229251
H	-3.554146	1.299585	2.573300
H	-2.256552	0.598221	4.530721
C	-3.238163	0.772612	-0.019896
C	-4.752628	0.792574	0.378177
C	-3.302735	2.341896	-0.088820
C	-4.690275	2.294122	0.502745
H	-5.048266	0.219390	1.276208
H	-5.393975	0.454236	-0.459159
H	-2.536459	2.891805	0.490826
H	-3.287626	2.722196	-1.130431
C	-3.054428	0.095855	-1.363357
H	-2.035357	0.172850	-1.799879
H	-3.310338	-0.977646	-1.311809
H	-3.720412	0.567175	-2.111278
C	-5.502415	3.212699	1.020755
H	-5.223550	4.275329	1.042291
H	-6.477484	2.937877	1.446437

IV-q

C	-1.561083	-0.899754	-0.786298
C	0.552148	-1.465102	-1.593218
C	0.130740	-2.747166	-1.924235
C	-1.209126	-3.075990	-1.759694
C	-2.097926	-2.126801	-1.238249
C	-2.324778	0.172133	-0.120097
C	-2.485052	2.488552	0.132691
C	-3.624160	2.364494	0.919211
C	-4.057388	1.094545	1.278735
C	-3.380781	-0.033163	0.795258
H	1.574471	-1.119307	-1.786608
H	0.836426	-3.470364	-2.344362
H	-1.569070	-4.060177	-2.073580

H	-2.034774	3.461139	-0.094820
H	-4.141117	3.255181	1.287837
H	-4.908012	0.984226	1.957615
N	-0.279565	-0.592268	-1.034390
N	-1.864157	1.415112	-0.354719
O	-3.419264	-2.282850	-1.197779
O	-3.616560	-1.278277	1.203467
C	-3.986571	-3.523610	-1.548106
H	-3.798880	-3.767158	-2.611453
H	-3.595699	-4.337624	-0.908384
H	-5.070684	-3.427439	-1.387203
C	-4.658377	-1.528054	2.118470
H	-4.666799	-2.614528	2.291156
H	-4.480219	-1.009479	3.080675
H	-5.638167	-1.219327	1.707357
Ni	0.135098	1.402361	-0.815662
Cl	0.810121	1.607690	-2.953033
Br	0.911705	3.444182	0.204105
C	1.344534	0.373945	1.558997
C	0.118631	0.466327	2.186906
C	2.061249	-0.785830	1.314197
C	-0.480135	-0.730975	2.594429
H	-0.369412	1.435228	2.360902
C	1.428283	-1.967937	1.739544
C	0.177099	-1.940610	2.357772
H	-1.455566	-0.718516	3.097244
H	1.924427	-2.937054	1.590406
H	-0.287451	-2.882324	2.672473
C	3.436289	-0.781342	0.691229
C	4.583220	-1.310053	1.613274
C	3.798919	-1.926692	-0.304859
C	5.101864	-2.099481	0.437706
H	4.193439	-1.963976	2.418334
H	5.246189	-0.549035	2.067994
H	3.140770	-2.812573	-0.211884
H	3.855328	-1.647438	-1.375645
C	3.804094	0.568487	0.098329

H	3.149012	0.836150	-0.755083
H	3.720475	1.376692	0.850234
H	4.846403	0.550007	-0.274882
C	6.288427	-2.607267	0.115230
H	6.457423	-3.092536	-0.856071
H	7.138927	-2.555795	0.809080

Va-s

C	1.757304	0.776007	0.405724
C	0.118620	1.898352	1.615019
C	0.876685	3.065216	1.626744
C	2.036815	3.124838	0.868513
C	2.457047	1.979211	0.181841
C	2.234024	-0.560267	0.027420
C	1.535132	-2.715746	-0.506062
C	2.842945	-3.125149	-0.742570
C	3.882920	-2.245522	-0.479468
C	3.593173	-0.949058	-0.034164
H	-0.862345	1.851238	2.098999
H	0.521294	3.940452	2.178223
H	2.597669	4.060968	0.786275
H	0.680801	-3.395518	-0.606405
H	3.047322	-4.143404	-1.086709
H	4.919052	-2.577078	-0.592928
N	0.540712	0.789915	1.000643
N	1.253147	-1.472790	-0.120247
O	3.444307	1.962921	-0.712528
O	4.518576	-0.089765	0.394445
C	4.482526	2.910354	-0.609926
H	4.141666	3.924086	-0.894063
H	4.892223	2.929844	0.418863
H	5.270919	2.592552	-1.309792
C	5.882968	-0.432463	0.311577
H	6.443979	0.428574	0.705136
H	6.113157	-1.324316	0.925265
H	6.188276	-0.615603	-0.736213

Ni	-0.519246	-0.781983	0.554198
Br	-1.690715	-2.792550	0.319997
C	-2.125059	0.062236	1.047534
C	-2.618991	-0.109019	2.349795
C	-2.813635	0.930191	0.178368
C	-3.744282	0.574222	2.810179
H	-2.104989	-0.801521	3.032761
C	-3.952224	1.606700	0.649239
C	-4.419049	1.442563	1.950469
H	-4.100360	0.422789	3.837401
H	-4.486715	2.286290	-0.031405
H	-5.309554	1.985517	2.290228
C	-2.373760	1.242418	-1.238057
C	-1.578714	0.185112	-2.054888
C	-3.478504	1.164155	-2.336886
C	-2.456528	0.516142	-3.236472
H	-1.765535	-0.843136	-1.688955
H	-0.483963	0.331539	-2.157865
H	-4.281074	0.456818	-2.046322
H	-3.942842	2.112565	-2.671517
C	-1.672911	2.595552	-1.314758
H	-2.318853	3.401676	-0.915629
H	-0.729885	2.594103	-0.735596
H	-1.421267	2.848317	-2.364242
C	-2.310030	0.427721	-4.556558
H	-3.076561	0.812985	-5.243632
H	-1.417429	-0.032670	-5.003126

Va-t

C	1.806527	0.853034	-0.658885
C	0.084112	1.828044	-1.883509
C	0.891477	2.894119	-2.262330
C	2.227615	2.889539	-1.886332
C	2.721766	1.831821	-1.112607
C	2.177343	-0.296925	0.191000
C	1.776802	-2.567742	0.537688

C	2.814608	-2.652690	1.460746
C	3.474823	-1.489192	1.834575
C	3.126244	-0.269216	1.237032
H	-0.957969	1.760292	-2.216564
H	0.486418	3.701660	-2.879161
H	2.890998	3.690560	-2.225053
H	1.151880	-3.428947	0.268413
H	3.074104	-3.612275	1.917582
H	4.244665	-1.527761	2.611012
N	0.538988	0.845783	-1.108031
N	1.480461	-1.416826	-0.059519
O	4.011100	1.653608	-0.833162
O	3.587212	0.915780	1.630959
C	4.949012	2.643568	-1.186058
H	5.013212	2.765750	-2.284309
H	4.700148	3.615888	-0.720012
H	5.922470	2.299855	-0.805270
C	4.638078	0.981785	2.566457
H	4.891348	2.046814	2.677170
H	4.329164	0.580877	3.550885
H	5.528699	0.432167	2.206401
Ni	-0.421605	-0.973203	-0.729050
Br	-1.019712	-3.097039	-1.716121
C	-1.388005	-0.537722	0.965586
C	-0.816921	-1.012791	2.157859
C	-2.595521	0.196270	1.072194
C	-1.373837	-0.781357	3.416905
H	0.114116	-1.598704	2.125321
C	-3.145429	0.434717	2.344639
C	-2.549047	-0.043751	3.509470
H	-0.887999	-1.176559	4.318498
H	-4.067000	1.018933	2.448305
H	-3.010036	0.160621	4.483774
C	-3.278518	0.801030	-0.152538
C	-4.762993	1.228392	0.075581
C	-2.942623	2.328507	-0.285218
C	-4.288365	2.637707	0.321104

H	-5.354563	0.721980	0.859776
H	-5.331475	1.153787	-0.872757
H	-2.039005	2.697602	0.236989
H	-2.891312	2.643541	-1.348338
C	-3.094738	-0.005012	-1.422398
H	-2.039385	-0.047165	-1.768247
H	-3.442421	-1.048293	-1.304072
H	-3.653301	0.456985	-2.259143
C	-4.797465	3.690774	0.956304
H	-4.222167	4.619488	1.075693
H	-5.808073	3.663695	1.387606

V-d

C	2.299865	0.625545	-0.248326
C	0.995223	2.547388	-0.374063
C	2.019494	3.255411	-0.991660
C	3.201031	2.593998	-1.309594
C	3.342339	1.241187	-0.976749
C	2.284550	-0.784293	0.182617
C	0.908678	-2.646238	0.427901
C	1.980117	-3.440651	0.813871
C	3.233674	-2.857516	0.969891
C	3.393146	-1.494603	0.692599
H	0.021896	3.004336	-0.155302
H	1.885169	4.309837	-1.252627
H	3.994095	3.123186	-1.846046
H	-0.106503	-3.054187	0.347676
H	1.830782	-4.504052	1.024637
H	4.072743	-3.458457	1.332300
N	1.143940	1.271803	-0.015862
N	1.061296	-1.351116	0.133393
O	4.369818	0.481035	-1.362707
O	4.510302	-0.806735	0.936482
C	5.456185	1.070769	-2.034329
H	5.147760	1.496568	-3.008862
H	5.927195	1.862882	-1.421025

H	6.189004	0.268620	-2.209463
C	5.668966	-1.488295	1.351423
H	6.465095	-0.732781	1.431743
H	5.525776	-1.967250	2.339396
H	5.971156	-2.255013	0.612338
Ni	-0.411383	-0.023850	0.271906
C	-2.013812	0.930927	0.801961
C	-1.843055	2.090724	1.593723
C	-3.354180	0.618399	0.455902
C	-2.888854	2.916498	2.004709
H	-0.823844	2.366600	1.911048
C	-4.409032	1.463081	0.847503
C	-4.191311	2.605486	1.613161
H	-2.690621	3.803448	2.621946
H	-5.442036	1.232402	0.548643
H	-5.035149	3.243491	1.905319
C	-3.722374	-0.618068	-0.353822
C	-4.957070	-1.448305	0.132542
C	-4.500762	-0.372916	-1.688747
C	-5.453321	-1.473297	-1.292411
H	-5.624793	-0.877999	0.805248
H	-4.737263	-2.421321	0.614369
H	-4.988494	0.621157	-1.708136
H	-3.929906	-0.498047	-2.630000
C	-2.574811	-1.578939	-0.609029
H	-1.767391	-1.109467	-1.208539
H	-2.137744	-1.930806	0.347719
H	-2.929698	-2.467203	-1.168600
C	-6.288480	-2.260862	-1.966796
H	-6.448823	-2.133287	-3.046789
H	-6.844554	-3.066656	-1.466939

V-q

C	2.050169	0.838855	0.027225
C	0.260273	2.299914	0.575320
C	1.118393	3.298060	0.976165

C	2.505051	3.125234	0.728695
C	2.966746	1.929766	0.206567
C	2.363034	-0.517174	-0.319988
C	1.481163	-2.486662	-1.314886
C	2.683887	-3.149520	-1.222582
C	3.745080	-2.511339	-0.534201
C	3.578733	-1.228448	-0.040688
H	-0.831596	2.418914	0.640124
H	0.734199	4.229918	1.401768
H	3.189313	3.963447	0.892127
H	0.589712	-2.962111	-1.747331
H	2.794791	-4.168033	-1.606418
H	4.667673	-3.066023	-0.338388
N	0.698176	1.131318	0.077886
N	1.318754	-1.231709	-0.868131
O	4.222273	1.742918	-0.231171
O	4.458807	-0.627843	0.775842
C	5.194744	2.713311	0.041977
H	4.975887	3.671346	-0.471334
H	5.283712	2.902523	1.130353
H	6.152507	2.319303	-0.333495
C	5.689909	-1.244966	1.032396
H	6.260531	-0.559201	1.678495
H	5.562666	-2.211994	1.559487
H	6.260328	-1.418466	0.098278
Ni	-0.384635	-0.238836	-0.820275
C	-1.805138	-1.092456	0.207025
C	-1.413624	-2.140370	1.058426
C	-3.156747	-0.688234	0.225146
C	-2.310603	-2.787604	1.906601
H	-0.361394	-2.463861	1.062912
C	-4.053458	-1.335151	1.095265
C	-3.642282	-2.373266	1.926427
H	-1.973137	-3.606244	2.554944
H	-5.104490	-1.017994	1.138673
H	-4.366826	-2.859086	2.591602
C	-3.677365	0.459948	-0.628033

C	-5.135418	0.252874	-1.154805
C	-4.220039	1.646015	0.241709
C	-5.627655	1.252355	-0.136685
H	-5.550614	-0.770487	-1.096557
H	-5.252232	0.603588	-2.199177
H	-3.968674	1.615198	1.319071
H	-3.915997	2.636060	-0.154604
C	-2.727897	0.916628	-1.716608
H	-1.811966	1.393389	-1.301885
H	-2.441141	0.088380	-2.394891
H	-3.198204	1.702565	-2.337574
C	-6.832445	1.584844	0.322098
H	-6.954360	2.327197	1.123264
H	-7.745136	1.125543	-0.082784

VI'-d

C	3.416277	0.419913	-0.413374
C	2.582595	2.587392	-0.562191
C	3.861863	3.131106	-0.523422
C	4.954449	2.274842	-0.526423
C	4.745083	0.889402	-0.521126
C	3.046407	-1.002892	-0.272811
C	1.462605	-2.611580	-0.855111
C	2.203586	-3.621583	-0.251179
C	3.354523	-3.283109	0.447974
C	3.771843	-1.945486	0.490291
H	1.695449	3.228893	-0.627516
H	4.000918	4.216013	-0.529986
H	5.967971	2.684923	-0.560833
H	0.506658	-2.812867	-1.351049
H	1.860482	-4.659323	-0.292447
H	3.911729	-4.057169	0.983761
N	2.382165	1.272035	-0.520890
N	1.882924	-1.348204	-0.850238
O	5.710934	-0.012877	-0.671374
O	4.768086	-1.496535	1.248781

C	7.060440	0.391194	-0.628109
H	7.312828	1.048251	-1.482152
H	7.290133	0.912194	0.320734
H	7.663007	-0.527208	-0.691778
C	5.609904	-2.408977	1.915827
H	6.390256	-1.809639	2.408286
H	5.056523	-2.981914	2.684086
H	6.085129	-3.108261	1.201917
Ni	0.612352	0.261759	-0.980082
C	-0.363137	0.343126	0.738785
C	-0.093584	-0.667947	1.674739
C	-1.255980	1.370672	1.109636
C	-0.676485	-0.690408	2.941833
H	0.597900	-1.486079	1.419917
C	-1.829530	1.345168	2.393398
C	-1.554260	0.329243	3.303805
H	-0.445554	-1.502628	3.642912
H	-2.512178	2.148350	2.703299
H	-2.023700	0.338803	4.295052
C	-1.596789	2.544522	0.206284
C	-3.075161	3.051384	0.187634
C	-1.191585	3.956504	0.747083
C	-2.554188	4.459209	0.339802
H	-3.662167	2.712081	1.060767
H	-3.662302	2.814010	-0.720736
H	-1.054217	3.950537	1.845819
H	-0.304984	4.438661	0.288721
C	-1.135959	2.396375	-1.232007
H	-0.031769	2.285119	-1.311546
H	-1.632520	1.550271	-1.739302
H	-1.371818	3.308160	-1.813526
C	-3.051270	5.664118	0.070618
H	-2.447115	6.573433	0.197231
H	-4.082534	5.789617	-0.288090
O	-0.732505	-0.785410	-2.152959
C	-1.858494	-1.178878	-1.921021
O	-2.592206	-0.990381	-0.920856

Al	-4.186127	-1.598283	-0.291072
Cl	-3.728371	-2.822891	1.396521
Cl	-5.288489	0.149524	0.250349
Cl	-5.096277	-2.694331	-1.882975

VI'-q

C	3.030784	0.343742	-0.591891
C	2.214546	2.438556	-1.211123
C	3.500662	2.949079	-1.349460
C	4.581226	2.091349	-1.197702
C	4.357224	0.748197	-0.866342
C	2.641482	-0.991699	-0.095165
C	0.939941	-2.591862	-0.151927
C	1.688781	-3.480952	0.611718
C	2.913875	-3.062361	1.112256
C	3.393445	-1.779476	0.806490
H	1.330837	3.064651	-1.386820
H	3.652018	4.000023	-1.611952
H	5.597133	2.460299	-1.366380
H	-0.075366	-2.836412	-0.486092
H	1.295090	-4.473491	0.849700
H	3.484768	-3.724569	1.769609
N	2.004571	1.172909	-0.857558
N	1.415159	-1.392835	-0.474315
O	5.305013	-0.183825	-0.853094
O	4.472267	-1.237538	1.364673
C	6.661746	0.193584	-0.911234
H	6.914393	0.644700	-1.889671
H	6.912015	0.903273	-0.099768
H	7.247905	-0.728241	-0.779624
C	5.296462	-2.014573	2.203276
H	6.136984	-1.368520	2.497721
H	4.754730	-2.339486	3.111991
H	5.689376	-2.900558	1.669400
Ni	0.196648	0.198886	-0.876568
C	-0.466015	0.814939	0.875297

C	-0.039448	0.132564	2.026131
C	-1.268947	1.961852	1.042996
C	-0.381459	0.548423	3.312017
H	0.578699	-0.773138	1.929802
C	-1.596965	2.384256	2.344876
C	-1.163576	1.691738	3.470639
H	-0.035700	-0.016326	4.187153
H	-2.207754	3.286822	2.488003
H	-1.439715	2.045299	4.471637
C	-1.783706	2.793569	-0.122003
C	-3.255720	3.292473	0.053890
C	-1.323930	4.290405	-0.050514
C	-2.739265	4.690416	0.292649
H	-3.839403	2.828454	0.870911
H	-3.847848	3.190682	-0.877490
H	-0.551080	4.523846	0.706634
H	-0.974660	4.679836	-1.028351
C	-1.566204	2.165994	-1.481983
H	-0.485739	2.039011	-1.723496
H	-2.090927	1.196502	-1.565179
H	-1.952011	2.823017	-2.284503
C	-3.297938	5.818906	0.725329
H	-2.705061	6.733274	0.867806
H	-4.370884	5.872830	0.957378
O	-0.820852	-0.849890	-2.446216
C	-1.603826	-1.762271	-2.610357
O	-2.371195	-2.383920	-1.835453
Al	-3.393334	-2.218326	-0.332806
Cl	-2.195826	-2.764888	1.350468
Cl	-4.055829	-0.184416	-0.281330
Cl	-5.008912	-3.584965	-0.619218

VI-d

C	2.872169	-0.224490	0.516975
C	2.263190	1.565584	1.886639
C	3.579460	2.009246	1.963263
C	4.548297	1.364827	1.205804

C	4.198444	0.254209	0.423579
C	2.357231	-1.437510	-0.149916
C	0.485052	-2.378601	-1.183292
C	1.188959	-3.535410	-1.497363
C	2.492715	-3.674459	-1.043874
C	3.087067	-2.635552	-0.313625
H	1.440191	2.088222	2.391331
H	3.837385	2.876564	2.577790
H	5.574770	1.743114	1.203199
H	-0.567102	-2.262598	-1.468754
H	0.701020	-4.336236	-2.060602
H	3.037231	-4.604209	-1.232362
N	1.940801	0.489072	1.173957
N	1.063102	-1.374719	-0.523056
O	5.016316	-0.336820	-0.441717
O	4.269010	-2.727267	0.288157
C	6.376934	0.030088	-0.485186
H	6.500615	1.081087	-0.808681
H	6.860915	-0.115075	0.499190
H	6.853864	-0.631598	-1.223409
C	5.097496	-3.841755	0.044954
H	6.036007	-3.655756	0.588078
H	4.639648	-4.774968	0.424388
H	5.316716	-3.947864	-1.034428
Ni	0.063619	0.151598	0.368257
C	-0.981482	1.577504	1.095106
C	-1.561534	1.322812	2.341307
C	-0.965057	2.881472	0.562025
C	-2.119961	2.350575	3.099110
H	-1.586851	0.294792	2.729262
C	-1.555647	3.895510	1.334382
C	-2.119219	3.644482	2.583147
H	-2.562561	2.138386	4.079439
H	-1.587726	4.922995	0.952833
H	-2.565889	4.468698	3.151368
C	-0.414725	3.205987	-0.815580
C	-1.492980	3.447521	-1.922504

C	0.100830	4.653416	-1.096361
C	-0.690239	4.637919	-2.382170
H	-2.462177	3.744504	-1.474374
H	-1.672481	2.618462	-2.631533
H	-0.274443	5.410373	-0.384867
H	1.199029	4.773829	-1.168816
C	0.644592	2.238045	-1.322409
H	1.553323	2.253785	-0.691841
H	0.263476	1.199201	-1.443786
H	0.961418	2.523164	-2.343978
C	-0.601529	5.310113	-3.527075
H	0.129207	6.120358	-3.658773
H	-1.255952	5.077115	-4.378524
O	-2.022183	0.232857	-1.443053
C	-1.648560	-0.156210	-0.366623
O	-2.170797	-1.055870	0.403293
Al	-3.597127	-2.111420	0.032757
Cl	-5.318281	-0.876418	-0.266113
Cl	-3.121279	-3.255641	-1.723791
Cl	-3.795936	-3.370191	1.752208

VI-q

C	3.030784	0.343742	-0.591891
C	2.214546	2.438556	-1.211123
C	3.500662	2.949079	-1.349460
C	4.581226	2.091349	-1.197702
C	4.357224	0.748197	-0.866342
C	2.641482	-0.991699	-0.095165
C	0.939941	-2.591862	-0.151927
C	1.688781	-3.480952	0.611718
C	2.913875	-3.062361	1.112256
C	3.393445	-1.779476	0.806490
H	1.330837	3.064651	-1.386820
H	3.652018	4.000023	-1.611952
H	5.597133	2.460299	-1.366380
H	-0.075366	-2.836412	-0.486092
H	1.295090	-4.473491	0.849700

H	3.484768	-3.724569	1.769609
N	2.004571	1.172909	-0.857558
N	1.415159	-1.392835	-0.474315
O	5.305013	-0.183825	-0.853094
O	4.472267	-1.237538	1.364673
C	6.661746	0.193584	-0.911234
H	6.914393	0.644700	-1.889671
H	6.912015	0.903273	-0.099768
H	7.247905	-0.728241	-0.779624
C	5.296462	-2.014573	2.203276
H	6.136984	-1.368520	2.497721
H	4.754730	-2.339486	3.111991
H	5.689376	-2.900558	1.669400
Ni	0.196648	0.198886	-0.876568
C	-0.466015	0.814939	0.875297
C	-0.039448	0.132564	2.026131
C	-1.268947	1.961852	1.042996
C	-0.381459	0.548423	3.312017
H	0.578699	-0.773138	1.929802
C	-1.596965	2.384256	2.344876
C	-1.163576	1.691738	3.470639
H	-0.035700	-0.016326	4.187153
H	-2.207754	3.286822	2.488003
H	-1.439715	2.045299	4.471637
C	-1.783706	2.793569	-0.122003
C	-3.255720	3.292473	0.053890
C	-1.323930	4.290405	-0.050514
C	-2.739265	4.690416	0.292649
H	-3.839403	2.828454	0.870911
H	-3.847848	3.190682	-0.877490
H	-0.551080	4.523846	0.706634
H	-0.974660	4.679836	-1.028351
C	-1.566204	2.165994	-1.481983
H	-0.485739	2.039011	-1.723496
H	-2.090927	1.196502	-1.565179
H	-1.952011	2.823017	-2.284503
C	-3.297938	5.818906	0.725329

H	-2.705061	6.733274	0.867806
H	-4.370884	5.872830	0.957378
O	-0.820852	-0.849890	-2.446216
C	-1.603826	-1.762271	-2.610357
O	-2.371195	-2.383920	-1.835453
Al	-3.393334	-2.218326	-0.332806
Cl	-2.195826	-2.764888	1.350468
Cl	-4.055829	-0.184416	-0.281330
Cl	-5.008912	-3.584965	-0.619218

TS-VI-VII-d

C	2.865619	0.325351	0.449839
C	1.950091	1.923546	1.885138
C	3.200008	2.485259	2.125976
C	4.298326	1.997221	1.430666
C	4.139692	0.929961	0.534411
C	2.539565	-0.856491	-0.378311
C	0.866826	-1.838689	-1.684196
C	1.692403	-2.906494	-2.016499
C	2.953922	-2.982145	-1.442642
C	3.385835	-1.970810	-0.571553
H	1.034142	2.315535	2.347760
H	3.307937	3.321459	2.822878
H	5.276872	2.467774	1.563998
H	-0.161038	-1.771001	-2.060521
H	1.331441	-3.691212	-2.688006
H	3.593213	-3.845433	-1.649566
N	1.810597	0.880504	1.071356
N	1.290722	-0.862051	-0.883425
O	5.093356	0.493736	-0.283418
O	4.512124	-2.025730	0.133947
C	6.400728	1.009402	-0.175435
H	6.428442	2.090163	-0.412151
H	6.815011	0.840920	0.836553
H	7.011557	0.464645	-0.910534
C	5.419657	-3.083730	-0.075360

H	6.280394	-2.889381	0.581570
H	4.971050	-4.058554	0.195331
H	5.764709	-3.113440	-1.126183
Ni	0.034458	0.350233	0.131722
C	-1.360098	1.340337	1.028553
C	-1.711938	0.931679	2.319379
C	-1.678067	2.639992	0.577381
C	-2.373580	1.791304	3.192718
H	-1.470411	-0.089465	2.641196
C	-2.366913	3.477834	1.466719
C	-2.709888	3.069644	2.754302
H	-2.634180	1.459335	4.204316
H	-2.650436	4.489540	1.154736
H	-3.245841	3.759985	3.415851
C	-1.335950	3.123573	-0.818910
C	-2.512050	3.125294	-1.848347
C	-1.199642	4.658049	-1.073254
C	-2.041941	4.479909	-2.313277
H	-3.490612	3.175664	-1.330181
H	-2.541595	2.291458	-2.573335
H	-1.705513	5.284816	-0.317589
H	-0.169318	5.043287	-1.197686
C	-0.113848	2.443347	-1.418440
H	0.793105	2.610993	-0.805741
H	-0.279866	1.358287	-1.614060
H	0.104709	2.850620	-2.423991
C	-2.182865	5.173974	-3.439596
H	-1.670741	6.135182	-3.586232
H	-2.815774	4.808613	-4.260196
O	-2.364165	-0.024600	-1.235642
C	-1.703339	-0.294278	-0.263036
O	-1.757620	-1.356977	0.482932
Al	-2.777452	-2.823366	0.189292
Cl	-4.841782	-2.258029	0.229217
Cl	-2.219954	-3.666459	-1.707485
Cl	-2.282341	-4.145592	1.799876

TS-VI-VII-q

C	3.115300	0.079516	0.580241
C	1.923647	1.411214	2.086872
C	3.072093	2.104638	2.446787
C	4.260078	1.830639	1.780701
C	4.289969	0.836685	0.793635
C	2.997115	-1.045794	-0.364467
C	1.562107	-2.083682	-1.883337
C	2.560206	-2.965350	-2.282537
C	3.782681	-2.944686	-1.622803
C	4.004999	-2.006332	-0.606739
H	0.946686	1.637777	2.532237
H	3.029289	2.881019	3.216011
H	5.156776	2.413671	2.010078
H	0.551584	-2.106258	-2.308638
H	2.368815	-3.691633	-3.077919
H	4.551729	-3.678525	-1.880902
N	1.962903	0.434260	1.178185
N	1.790803	-1.162374	-0.947866
O	5.331638	0.601311	0.001178
O	5.075342	-1.996632	0.184142
C	6.547916	1.278858	0.221228
H	6.432460	2.370040	0.076681
H	6.939844	1.078707	1.236390
H	7.258997	0.890677	-0.523010
C	6.149066	-2.869653	-0.081962
H	6.926610	-2.643392	0.662733
H	5.843649	-3.928152	0.024525
H	6.558198	-2.702931	-1.096497
Ni	0.285397	-0.171026	0.164910
C	-1.226366	1.144494	0.546796
C	-1.676996	0.874876	1.840361
C	-1.146354	2.451968	0.023522
C	-1.875734	1.937472	2.724638
H	-1.838247	-0.162902	2.167011
C	-1.361651	3.497175	0.925479

C	-1.714146	3.240808	2.256790
H	-2.178503	1.749661	3.761006
H	-1.272821	4.539209	0.598826
H	-1.891375	4.085957	2.931837
C	-0.864865	2.645350	-1.450578
C	-2.195622	2.453423	-2.253153
C	-0.869280	4.121765	-1.933372
C	-2.329958	3.955807	-2.269663
H	-2.994746	1.850425	-1.781957
H	-1.982316	2.038917	-3.259347
H	-0.562572	4.930401	-1.246387
H	-0.254035	4.209361	-2.850558
C	0.298297	1.789319	-1.916752
H	1.219587	1.978332	-1.329646
H	0.030635	0.711037	-1.876057
H	0.533912	1.988264	-2.979128
C	-3.361576	4.792296	-2.346137
H	-3.225155	5.877212	-2.237680
H	-4.382635	4.424830	-2.517612
O	-1.590764	-1.337784	-1.724372
C	-1.281498	-1.329449	-0.545668
O	-1.983404	-1.870678	0.417684
Al	-3.762336	-2.070999	0.450929
Cl	-4.664004	-0.225158	-0.198379
Cl	-4.399531	-3.724589	-0.762032
Cl	-4.215413	-2.421493	2.525582

VII-d

C	-0.902208	1.442017	1.068166
C	0.336838	0.367777	2.738599
C	1.225604	1.434874	2.849770
C	1.084448	2.481505	1.960036
C	0.057967	2.475744	1.003906
C	-2.183078	1.338815	0.328604
C	-3.827127	-0.153223	-0.403662
C	-4.543776	0.861076	-1.029396

C	-4.094531	2.167327	-0.899869
C	-2.929889	2.430604	-0.165311
H	0.448128	-0.526271	3.364793
H	2.039220	1.408802	3.579880
H	1.790205	3.317142	1.934067
H	-4.166188	-1.196304	-0.420190
H	-5.460553	0.635112	-1.581690
H	-4.670716	2.988883	-1.335913
N	-0.685049	0.389021	1.895459
N	-2.691371	0.094313	0.244201
O	0.094149	3.471507	0.103143
O	-2.504051	3.655362	0.135282
C	0.100554	3.140422	-1.270237
H	0.903893	2.407937	-1.477930
H	-0.869002	2.731937	-1.619253
H	0.302154	4.076266	-1.815849
C	-3.023700	4.772020	-0.548184
H	-2.413558	5.635188	-0.241451
H	-2.941053	4.639048	-1.644806
H	-4.080028	4.959873	-0.276770
Ni	-1.864696	-1.215879	1.483730
C	0.085712	-1.265503	-1.617253
C	-0.398431	-0.299084	-2.509427
C	-0.701191	-2.394032	-1.302857
C	-1.616111	-0.458638	-3.160876
H	0.223950	0.578486	-2.714825
C	-1.908785	-2.548399	-1.997777
C	-2.363148	-1.608847	-2.918476
H	-1.970383	0.301966	-3.866315
H	-2.522615	-3.438356	-1.812083
H	-3.314970	-1.772583	-3.437581
C	-0.300018	-3.472410	-0.306867
C	0.371032	-2.949337	1.005081
C	-1.501688	-3.928704	0.584068
C	-0.970569	-3.044699	1.685168
H	0.883321	-1.971122	1.017851
H	1.087155	-3.698064	1.401380

H	-2.538917	-3.762206	0.238182
H	-1.403608	-4.998815	0.858632
C	0.418992	-4.607161	-1.020539
H	-0.234219	-5.054045	-1.795428
H	1.338938	-4.235710	-1.504673
H	0.694099	-5.406522	-0.305245
C	-1.492340	-2.644536	2.892605
H	-2.465700	-3.040474	3.221523
H	-0.855127	-2.211247	3.677564
O	1.836768	0.134324	-0.850161
C	1.490840	-1.096103	-1.134720
O	2.264904	-2.036081	-1.060367
Al	3.525773	0.483690	-0.337850
Cl	3.623026	2.637725	-0.216899
Cl	4.976425	-0.142527	-1.797738
Cl	3.881750	-0.403760	1.592261

VII-q

C	-2.015262	-1.113112	-0.453815
C	0.165239	-1.890576	-0.992736
C	-0.093571	-3.136794	-0.473699
C	-1.305683	-3.338447	0.224484
C	-2.240751	-2.318866	0.287855
C	-2.923192	-0.024470	-0.644484
C	-3.062262	2.302378	-1.095884
C	-4.422177	2.313209	-0.908936
C	-5.091584	1.080294	-0.719124
C	-4.355871	-0.090343	-0.647712
H	1.127115	-1.646855	-1.460528
H	0.666164	-3.922026	-0.534755
H	-1.467891	-4.276434	0.763961
H	-2.525748	3.221948	-1.358573
H	-4.981895	3.249838	-0.986675
H	-6.184973	1.056173	-0.705405
N	-0.749979	-0.902293	-0.959007
N	-2.328497	1.175288	-0.989540

O	-3.319735	-2.346171	1.082286
O	-4.900723	-1.312375	-0.667780
C	-3.662279	-3.539384	1.734941
H	-3.809640	-4.366786	1.013219
H	-2.891717	-3.839247	2.473650
H	-4.608976	-3.350556	2.264826
C	-6.293419	-1.449182	-0.569368
H	-6.504045	-2.529756	-0.544077
H	-6.681075	-0.982977	0.357650
H	-6.810307	-1.002605	-1.441899
Ni	-0.405509	0.969006	-1.340724
C	1.084540	0.404517	1.845827
C	0.496105	-0.765683	2.344744
C	0.303593	1.576322	1.687902
C	-0.834961	-0.800139	2.738338
H	1.115042	-1.664959	2.428151
C	-1.043157	1.507076	2.083007
C	-1.610527	0.348351	2.603480
H	-1.267354	-1.722723	3.141965
H	-1.675696	2.397729	1.995671
H	-2.667969	0.342065	2.892564
C	0.852150	2.920075	1.214715
C	1.742969	2.847158	-0.065461
C	-0.167994	3.711955	0.328909
C	0.525743	3.155885	-0.877727
H	2.321958	1.948451	-0.325072
H	2.438748	3.709982	-0.090781
H	-1.256495	3.581866	0.441257
H	0.050981	4.796544	0.395933
C	1.415147	3.693092	2.396185
H	0.628342	3.866311	3.155557
H	2.240741	3.128349	2.863946
H	1.801803	4.678228	2.070721
C	0.184102	2.893316	-2.168899
H	-0.749340	3.276285	-2.604765
H	0.957471	2.531779	-2.865443
O	2.902212	-0.825925	1.007702

C	2.546412	0.327702	1.520545
O	3.322261	1.242099	1.723021
Al	3.935077	-1.138641	-0.396585
Cl	3.706572	-3.219255	-0.875481
Cl	5.996022	-0.641354	-0.103597
Cl	3.079191	0.068348	-1.993026

VIII-d

C	-3.005252	-0.873054	-0.020329
C	-2.027700	-2.940918	-0.381447
C	-3.246092	-3.594749	-0.254614
C	-4.400123	-2.842101	-0.079189
C	-4.299013	-1.449869	-0.002453
C	-2.775127	0.578495	0.067878
C	-1.620823	2.366614	-0.842426
C	-2.432671	3.281604	-0.177310
C	-3.374414	2.815928	0.728188
C	-3.517062	1.434770	0.903065
H	-1.113379	-3.505539	-0.586279
H	-3.295338	-4.685263	-0.326780
H	-5.373761	-3.338434	-0.034007
H	-0.810009	2.699843	-1.497791
H	-2.286454	4.354733	-0.333607
H	-3.972815	3.518188	1.317064
N	-1.915584	-1.616144	-0.268755
N	-1.783530	1.050775	-0.712510
O	-5.350462	-0.627127	0.022264
O	-4.271742	0.873157	1.851760
C	-6.652250	-1.162148	0.075904
H	-6.876527	-1.770567	-0.821302
H	-6.796612	-1.777674	0.984489
H	-7.340598	-0.304044	0.108741
C	-5.402955	1.566740	2.323516
H	-5.990581	0.848976	2.917154
H	-5.122971	2.419296	2.971490
H	-6.018764	1.930314	1.477458

Ni	-0.330288	-0.406988	-1.016983
C	2.986236	-1.030330	1.436317
C	3.509566	-0.781443	2.711016
C	3.282411	-2.235176	0.756062
C	4.290828	-1.724853	3.368410
H	3.286223	0.177028	3.195253
C	4.092786	-3.155483	1.437553
C	4.581071	-2.920967	2.720828
H	4.677839	-1.519672	4.372536
H	4.363773	-4.097641	0.952562
H	5.204442	-3.679328	3.208152
C	2.766034	-2.573649	-0.634995
C	1.235358	-2.880867	-0.679506
C	2.526322	-1.385918	-1.615007
C	1.051826	-1.753129	-1.684167
H	0.733844	-2.727079	0.293210
H	0.996567	-3.899183	-1.045961
H	2.782939	-0.380961	-1.246881
H	3.057596	-1.503014	-2.578715
C	3.547708	-3.679808	-1.326948
H	4.621787	-3.429012	-1.421977
H	3.458877	-4.650128	-0.804524
H	3.143204	-3.829524	-2.345922
C	0.108721	-1.502386	-2.668783
H	0.382494	-0.858945	-3.519892
H	-0.740880	-2.181858	-2.836886
O	2.788709	1.182149	0.692981
C	2.140590	0.076403	0.882696
O	0.934600	-0.050735	0.674090
Al	2.314771	2.633514	-0.247347
Cl	4.115648	3.728504	-0.611050
Cl	0.906623	3.803721	0.856619
Cl	1.511508	1.908084	-2.129804

VIII-q

C	3.026875	0.609685	-0.390957

C	2.040768	2.404023	-1.495877
C	3.254364	3.076616	-1.575607
C	4.405714	2.447148	-1.118192
C	4.314833	1.169740	-0.552267
C	2.767053	-0.696384	0.240907
C	1.513910	-2.645478	0.119069
C	2.238594	-3.225287	1.156291
C	3.203069	-2.471446	1.810220
C	3.446291	-1.159966	1.384905
H	1.118291	2.831475	-1.904013
H	3.305014	4.072081	-2.026281
H	5.375625	2.940705	-1.229941
H	0.701951	-3.184653	-0.380030
H	2.017064	-4.249024	1.472891
H	3.739937	-2.887288	2.668650
N	1.946957	1.206507	-0.919186
N	1.774564	-1.413770	-0.315478
O	5.365303	0.423545	-0.211212
O	4.231587	-0.295681	2.028638
C	6.661485	0.972564	-0.274490
H	6.937651	1.237307	-1.313177
H	6.748755	1.867180	0.371210
H	7.347475	0.194223	0.092283
C	5.262339	-0.782544	2.856353
H	5.897514	0.080488	3.109401
H	4.864727	-1.219917	3.791940
H	5.870584	-1.538513	2.322447
Ni	0.386899	-0.140701	-1.280300
C	-2.549252	1.118609	1.426009
C	-2.748630	0.570453	2.697856
C	-2.971405	2.433481	1.128898
C	-3.355924	1.304975	3.712044
H	-2.420575	-0.461754	2.883523
C	-3.585802	3.145392	2.168635
C	-3.776250	2.601870	3.438319
H	-3.502189	0.862624	4.703665
H	-3.933612	4.167395	1.990652

H	-4.261263	3.203302	4.215837
C	-2.758040	3.071433	-0.232780
C	-1.263249	2.974159	-0.678951
C	-3.116917	2.117449	-1.418559
C	-1.651883	1.844220	-1.583225
H	-0.516352	2.769531	0.118739
H	-0.934865	3.899781	-1.208343
H	-3.792312	1.263731	-1.202773
H	-3.568301	2.681390	-2.268216
C	-3.346243	4.461824	-0.365988
H	-4.442313	4.469549	-0.209976
H	-2.891148	5.178915	0.343876
H	-3.154259	4.845426	-1.385619
C	-0.868394	0.952374	-2.430946
H	-1.520713	0.275425	-3.016263
H	-0.215124	1.501778	-3.147457
O	-2.693738	-0.658412	-0.116495
C	-1.889789	0.157670	0.482574
O	-0.652098	0.032073	0.470723
Al	-2.491060	-2.375579	-0.568307
Cl	-4.394719	-3.077306	-1.225704
Cl	-1.792808	-3.371937	1.198812
Cl	-1.036070	-2.565806	-2.167971

IX-d

C	-3.436041	-0.131162	-0.858891
C	-3.377105	-2.446965	-1.046908
C	-4.685396	-2.559665	-0.585261
C	-5.416326	-1.400458	-0.356994
C	-4.808970	-0.155078	-0.554472
C	-2.591486	1.079282	-0.875741
C	-0.680103	2.014158	-1.835139
C	-0.771495	3.142124	-1.027253
C	-1.775391	3.199885	-0.071882
C	-2.704639	2.153883	0.032469
H	-2.784589	-3.332507	-1.309166

H	-5.139181	-3.545174	-0.444790
H	-6.468036	-1.461725	-0.059324
H	0.137038	1.893201	-2.556866
H	-0.032271	3.943781	-1.122250
H	-1.832882	4.057267	0.605403
N	-2.784770	-1.263336	-1.178560
N	-1.561897	1.021960	-1.741004
O	-5.461935	1.008359	-0.524092
O	-3.644691	2.096283	0.976167
C	-6.562834	1.147864	0.344690
H	-7.460443	0.627814	-0.041039
H	-6.312780	0.762151	1.352503
H	-6.778677	2.225545	0.413533
C	-3.543907	2.931338	2.105963
H	-4.326358	2.600774	2.806573
H	-2.553121	2.823709	2.590135
H	-3.719134	3.993382	1.849067
Ni	-1.111187	-0.860228	-2.252781
C	2.560968	-0.739274	0.886554
C	3.322727	-0.223812	1.941482
C	2.811015	-2.018848	0.343314
C	4.341858	-0.976557	2.513974
H	3.101065	0.780726	2.325215
C	3.853968	-2.739965	0.938163
C	4.600936	-2.242987	2.005308
H	4.927382	-0.570435	3.345310
H	4.108474	-3.733499	0.560476
H	5.401390	-2.857306	2.432308
C	2.052227	-2.599507	-0.839177
C	0.511008	-2.841106	-0.688701
C	1.742567	-1.657903	-2.046601
C	0.345076	-2.245939	-2.070622
H	0.046102	-2.231121	0.104485
H	0.196647	-3.893184	-0.550045
H	1.762324	-0.580905	-1.805459
H	2.369045	-1.803050	-2.946632
C	2.681026	-3.873560	-1.388403

H	3.726725	-3.706238	-1.708331
H	2.661211	-4.697890	-0.652111
H	2.112698	-4.216953	-2.273033
C	-0.466990	-2.579580	-3.132831
H	-0.161639	-2.318615	-4.158583
H	-1.240187	-3.357034	-3.037829
O	1.790567	1.224614	-0.216299
C	1.488518	0.198906	0.450097
O	0.293467	0.014935	0.819991
Al	3.308347	2.001016	-0.903995
Cl	4.662104	0.478203	-1.542549
Cl	4.076481	3.204764	0.680903
Cl	2.596569	3.155978	-2.557146
Al	-0.398005	-0.507265	2.446321
Cl	-2.503553	-0.681939	2.210152
Cl	0.190148	1.126207	3.699469
Cl	0.460492	-2.366121	3.050352

IX-q

C	-3.368300	0.112356	-0.983239
C	-2.636931	-0.993046	-2.891955
C	-3.943648	-1.224747	-3.309405
C	-4.988778	-0.665922	-2.587161
C	-4.703956	0.073982	-1.433202
C	-2.949713	0.671984	0.311497
C	-1.229788	1.770480	1.434609
C	-1.984739	1.848082	2.598479
C	-3.220222	1.219863	2.642775
C	-3.702042	0.563148	1.502648
H	-1.785946	-1.353867	-3.478598
H	-4.135579	-1.802589	-4.217972
H	-6.019780	-0.774790	-2.937716
H	-0.205161	2.154494	1.400860
H	-1.578016	2.351260	3.480488
H	-3.792314	1.211345	3.575029
N	-2.370063	-0.337486	-1.765077

N	-1.713387	1.203050	0.331373
O	-5.601354	0.793509	-0.763769
O	-4.790633	-0.203540	1.485943
C	-6.966683	0.452534	-0.848675
H	-7.391962	0.713194	-1.836349
H	-7.113074	-0.627400	-0.652867
H	-7.485374	1.035959	-0.072526
C	-5.584509	-0.317311	2.645363
H	-6.419761	-0.984927	2.385936
H	-5.012785	-0.762769	3.481741
H	-5.987824	0.666030	2.953275
Ni	-0.548274	0.506724	-1.219119
C	2.928569	-0.664967	0.464388
C	3.170663	-0.374437	1.811165
C	3.839071	-1.429309	-0.293793
C	4.296348	-0.888000	2.450154
H	2.451967	0.246521	2.361825
C	4.958874	-1.934464	0.371535
C	5.180842	-1.680793	1.726082
H	4.477442	-0.669950	3.508230
H	5.690834	-2.539197	-0.172646
H	6.068635	-2.099911	2.213215
C	3.629896	-1.618884	-1.784067
C	2.172921	-2.052884	-2.141272
C	3.294643	-0.247097	-2.449147
C	1.841715	-0.609502	-2.392449
H	1.585700	-2.617883	-1.393873
H	2.149972	-2.628213	-3.091212
H	3.651089	0.683476	-1.969570
H	3.627503	-0.227020	-3.509482
C	4.717929	-2.416500	-2.468610
H	5.709947	-1.938623	-2.362185
H	4.787370	-3.446294	-2.069939
H	4.501193	-2.492825	-3.550437
C	0.672837	0.030868	-2.853271
H	0.839387	1.002909	-3.352358
H	-0.001826	-0.639237	-3.411242

O	1.436571	1.138357	-0.219757
C	1.661046	-0.150265	-0.104696
O	0.523497	-0.830970	0.044424
Al	2.215156	2.752901	-0.019959
Cl	4.271494	2.769999	-0.603647
Cl	2.064401	3.260201	2.062885
Cl	0.955202	3.967326	-1.255375
Al	-0.023212	-2.082708	1.246991
Cl	-1.961917	-2.693243	0.577178
Cl	-0.115011	-1.087491	3.142650
Cl	1.360420	-3.713809	1.256129

TS-IX-X-d

C	-3.013856	-0.397176	-1.052011
C	-1.804480	-1.915881	-2.319397
C	-2.924928	-2.712388	-2.520592
C	-4.155450	-2.268871	-2.054469
C	-4.221226	-1.057794	-1.359940
C	-2.941915	0.770296	-0.158548
C	-1.877845	2.797689	0.235795
C	-2.771154	3.115608	1.253182
C	-3.697456	2.164981	1.653331
C	-3.752665	0.934319	0.986708
H	-0.832603	-2.209238	-2.730700
H	-2.837869	-3.652375	-3.072932
H	-5.064046	-2.843573	-2.259338
H	-1.068023	3.478423	-0.047236
H	-2.704697	4.081574	1.762584
H	-4.353870	2.367970	2.504666
N	-1.855380	-0.790514	-1.608308
N	-1.972271	1.656127	-0.443562
O	-5.358678	-0.455341	-1.012134
O	-4.472828	-0.107997	1.400401
C	-6.507759	-1.237565	-0.779680
H	-6.940075	-1.622227	-1.723016
H	-6.273183	-2.083782	-0.104830

H	-7.244687	-0.579641	-0.293388
C	-5.154544	-0.030472	2.631242
H	-5.604359	-1.020502	2.800383
H	-4.451631	0.197625	3.456294
H	-5.957716	0.730513	2.604926
Ni	-0.392092	0.670313	-1.483330
C	3.103063	-0.941198	0.605628
C	3.662247	-0.393148	1.764462
C	3.536518	-2.185524	0.113583
C	4.603646	-1.103999	2.500620
H	3.327225	0.597383	2.103015
C	4.472288	-2.889161	0.877445
C	4.991493	-2.366012	2.061787
H	5.025150	-0.674654	3.416108
H	4.831147	-3.864188	0.532876
H	5.725547	-2.946835	2.631969
C	3.117896	-2.635618	-1.267902
C	1.581996	-2.513931	-1.506455
C	3.297486	-1.421044	-2.232321
C	1.823966	-1.137721	-2.074450
H	0.901736	-2.616491	-0.636658
H	1.240082	-3.235883	-2.279562
H	4.041085	-0.645857	-1.965390
H	3.506954	-1.757251	-3.269994
C	3.742529	-3.933827	-1.726162
H	4.847225	-3.876644	-1.742929
H	3.452160	-4.783362	-1.079150
H	3.406966	-4.168108	-2.753732
C	0.992056	-0.228253	-2.747534
H	1.534007	0.569622	-3.283362
H	0.157892	-0.639801	-3.344945
O	2.526555	1.061764	-0.457270
C	2.078175	-0.069185	-0.048788
O	0.807901	-0.155002	0.329137
Al	2.207137	2.801378	-0.516430
Cl	3.930467	3.823096	-1.210880
Cl	1.525105	3.404000	1.407442

Cl	0.602080	2.960170	-2.001277
Al	-0.015693	-0.952895	1.783808
Cl	-1.543024	-2.292725	1.096826
Cl	-0.848977	0.674213	2.898484
Cl	1.246988	-2.125998	3.050610

X-d

C	-3.198965	-0.550522	-0.872661
C	-1.972756	-2.256408	-1.878481
C	-3.133219	-2.942577	-2.218651
C	-4.362028	-2.337523	-1.997994
C	-4.403322	-1.085923	-1.372030
C	-3.125601	0.575060	0.070536
C	-1.733727	2.268416	0.874976
C	-2.666223	2.629484	1.841712
C	-3.828582	1.883567	1.967405
C	-4.058100	0.800335	1.108008
H	-0.988794	-2.673821	-2.110935
H	-3.066075	-3.922211	-2.699530
H	-5.283804	-2.822922	-2.333189
H	-0.768691	2.782310	0.784669
H	-2.460055	3.462579	2.519767
H	-4.540809	2.121222	2.762776
N	-2.010799	-1.084916	-1.242240
N	-1.979228	1.272113	0.028120
O	-5.496548	-0.339572	-1.255549
O	-5.047408	-0.075446	1.267783
C	-6.762164	-0.963929	-1.227201
H	-7.052933	-1.340080	-2.225998
H	-6.768227	-1.795043	-0.495748
H	-7.484382	-0.195711	-0.911316
C	-5.959769	0.082051	2.332122
H	-6.660215	-0.763411	2.265379
H	-5.442169	0.048779	3.309768
H	-6.524459	1.029244	2.241640
Ni	-0.579941	0.337703	-1.134230

C	3.240864	-0.774349	0.367448
C	3.819477	0.003848	1.366659
C	3.747856	-2.046213	0.058288
C	4.878690	-0.503650	2.117081
H	3.430200	1.013366	1.556868
C	4.812709	-2.541360	0.810029
C	5.367968	-1.778732	1.840009
H	5.328748	0.100454	2.913143
H	5.227899	-3.532093	0.593616
H	6.203297	-2.184089	2.423074
C	3.123048	-2.768419	-1.117277
C	1.582158	-2.631119	-1.016317
C	3.043741	-1.743617	-2.284751
C	1.747564	-1.218609	-1.601228
H	1.107246	-2.753768	-0.024637
H	1.095754	-3.305979	-1.747257
H	3.866143	-1.017864	-2.418687
H	2.813939	-2.247555	-3.242160
C	3.691554	-4.132033	-1.419796
H	4.770672	-4.081590	-1.658779
H	3.564861	-4.822206	-0.563918
H	3.174219	-4.576066	-2.290593
C	0.730607	-0.610010	-2.486191
H	1.057139	0.281004	-3.042804
H	0.106360	-1.283861	-3.092459
O	2.521419	0.961182	-1.028239
C	2.121327	-0.220645	-0.489517
O	0.880912	-0.047144	0.251213
Al	2.082741	2.623186	-1.092587
Cl	3.325192	3.686462	-2.459629
Cl	1.907079	3.623750	0.785662
Cl	0.032534	2.532657	-1.997580
Al	0.498381	-0.552611	1.962428
Cl	-1.539339	-1.268339	2.037568
Cl	0.719365	1.174039	3.220959
Cl	1.603549	-2.220282	2.761369

X-q

C	-3.315515	-0.425785	-0.904592
C	-2.292407	-1.943207	-2.327562
C	-3.535069	-2.504790	-2.685464
C	-4.697066	-1.932212	-2.217394
C	-4.609816	-0.826169	-1.347748
C	-3.062530	0.543360	0.136819
C	-1.580053	2.138873	0.928187
C	-2.399802	2.424546	2.051905
C	-3.538520	1.692091	2.258138
C	-3.873407	0.682556	1.326555
H	-1.355602	-2.328562	-2.745446
H	-3.568593	-3.363822	-3.361504
H	-5.669601	-2.317216	-2.535977
H	-0.643662	2.691850	0.772927
H	-2.093043	3.205790	2.752768
H	-4.160028	1.860729	3.141527
N	-2.201908	-0.939251	-1.480160
N	-1.908332	1.235699	0.025911
O	-5.633257	-0.098840	-0.966488
O	-4.804163	-0.202912	1.518548
C	-6.960275	-0.471705	-1.307204
H	-7.102721	-0.441908	-2.402178
H	-7.192638	-1.478634	-0.917437
H	-7.616076	0.269995	-0.830992
C	-5.524132	-0.273347	2.746643
H	-6.192767	-1.139194	2.652112
H	-4.819351	-0.427905	3.582768
H	-6.117996	0.643935	2.899980
Ni	-0.516985	0.312367	-1.303049
C	3.218907	-0.842395	0.341092
C	3.742141	-0.144427	1.426553
C	3.741225	-2.092526	-0.027916
C	4.763528	-0.705362	2.192847
H	3.340099	0.849294	1.668938
C	4.767607	-2.642462	0.740752

C	5.270399	-1.957207	1.849644
H	5.170198	-0.161146	3.053322
H	5.193611	-3.617088	0.475189
H	6.076581	-2.404875	2.443308
C	3.177454	-2.721476	-1.283139
C	1.633516	-2.573989	-1.256159
C	3.159227	-1.606568	-2.362614
C	1.826717	-1.122004	-1.743463
H	1.105632	-2.765344	-0.300574
H	1.175640	-3.190667	-2.055393
H	4.010816	-0.899844	-2.400783
H	2.979623	-2.034336	-3.368695
C	3.757304	-4.065150	-1.650034
H	4.848911	-4.008474	-1.826355
H	3.582185	-4.819130	-0.857950
H	3.288071	-4.439646	-2.579524
C	0.769470	-0.471541	-2.600795
H	1.200274	0.363449	-3.189425
H	0.345534	-1.201442	-3.320355
O	2.517105	1.010516	-0.916680
C	2.124606	-0.233246	-0.518361
O	0.875042	-0.145072	0.217595
Al	2.109441	2.678650	-0.900290
Cl	3.569036	3.777922	-2.030958
Cl	1.928697	3.518193	1.079788
Cl	0.153704	2.946818	-1.870991
Al	0.434574	-0.751192	1.838768
Cl	-1.619026	-1.499975	1.785244
Cl	0.443075	0.929328	3.198728
Cl	1.487520	-2.424569	2.678820

XI-d

C	-3.005252	-0.873054	-0.020329
C	-2.027700	-2.940918	-0.381447
C	-3.246092	-3.594749	-0.254614
C	-4.400123	-2.842101	-0.079189

C	-4.299013	-1.449869	-0.002453
C	-2.775127	0.578495	0.067878
C	-1.620823	2.366614	-0.842426
C	-2.432671	3.281604	-0.177310
C	-3.374414	2.815928	0.728188
C	-3.517062	1.434770	0.903065
H	-1.113379	-3.505539	-0.586279
H	-3.295338	-4.685263	-0.326780
H	-5.373761	-3.338434	-0.034007
H	-0.810009	2.699843	-1.497791
H	-2.286454	4.354733	-0.333607
H	-3.972815	3.518188	1.317064
N	-1.915584	-1.616144	-0.268755
N	-1.783530	1.050775	-0.712510
O	-5.350462	-0.627127	0.022264
O	-4.271742	0.873157	1.851760
C	-6.652250	-1.162148	0.075904
H	-6.876527	-1.770567	-0.821302
H	-6.796612	-1.777674	0.984489
H	-7.340598	-0.304044	0.108741
C	-5.402955	1.566740	2.323516
H	-5.990581	0.848976	2.917154
H	-5.122971	2.419296	2.971490
H	-6.018764	1.930314	1.477458
Ni	-0.330288	-0.406988	-1.016983
C	2.986236	-1.030330	1.436317
C	3.509566	-0.781443	2.711016
C	3.282411	-2.235176	0.756062
C	4.290828	-1.724853	3.368410
H	3.286223	0.177028	3.195253
C	4.092786	-3.155483	1.437553
C	4.581071	-2.920967	2.720828
H	4.677839	-1.519672	4.372536
H	4.363773	-4.097641	0.952562
H	5.204442	-3.679328	3.208152
C	2.766034	-2.573649	-0.634995
C	1.235358	-2.880867	-0.679506

C	2.526322	-1.385918	-1.615007
C	1.051826	-1.753129	-1.684167
H	0.733844	-2.727079	0.293210
H	0.996567	-3.899183	-1.045961
H	2.782939	-0.380961	-1.246881
H	3.057596	-1.503014	-2.578715
C	3.547708	-3.679808	-1.326948
H	4.621787	-3.429012	-1.421977
H	3.458877	-4.650128	-0.804524
H	3.143204	-3.829524	-2.345922
C	0.108721	-1.502386	-2.668783
H	0.382494	-0.858945	-3.519892
H	-0.740880	-2.181858	-2.836886
O	2.788709	1.182149	0.692981
C	2.140590	0.076403	0.882696
O	0.934600	-0.050735	0.674090
Al	2.314771	2.633514	-0.247347
Cl	4.115648	3.728504	-0.611050
Cl	0.906623	3.803721	0.856619
Cl	1.511508	1.908084	-2.129804

XII-d

C	-2.835125	-0.647969	0.085488
C	-1.808513	-2.742138	0.025342
C	-3.032099	-3.388552	0.135685
C	-4.184723	-2.637077	0.322126
C	-4.090028	-1.241111	0.351056
C	-2.582867	0.796280	-0.007677
C	-0.939796	2.405538	0.400533
C	-1.811601	3.435506	0.065408
C	-3.074664	3.118529	-0.420855
C	-3.464502	1.776127	-0.518674
H	-0.875669	-3.307164	-0.048462
H	-3.074559	-4.481535	0.118810
H	-5.144774	-3.136957	0.478490
H	0.102525	2.573385	0.694438

H	-1.490223	4.478839	0.139002
H	-3.742890	3.916893	-0.756769
N	-1.713311	-1.408108	0.012305
N	-1.330827	1.133696	0.356350
O	-5.097581	-0.433868	0.677627
O	-4.579455	1.366369	-1.122332
C	-6.392879	-0.962066	0.837717
H	-6.732978	-1.476479	-0.081295
H	-6.440635	-1.664049	1.692363
H	-7.055785	-0.107029	1.038510
C	-5.520934	2.313398	-1.567879
H	-6.365992	1.742749	-1.981582
H	-5.883525	2.943201	-0.733158
H	-5.099823	2.960470	-2.361367
Ni	-0.098097	-0.410906	0.256851
C	3.758209	0.819840	0.378861
C	3.937150	2.158728	0.823088
C	4.896091	0.097794	-0.132144
C	5.191535	2.749984	0.782211
H	3.069301	2.713341	1.202343
C	6.133108	0.718398	-0.161970
C	6.300248	2.041898	0.295382
H	5.316973	3.782892	1.133167
H	7.005884	0.180510	-0.553741
H	7.290331	2.512214	0.263304
C	4.621786	-1.310301	-0.619163
C	3.763786	-2.031190	0.454962
C	3.320380	-1.299906	-1.473870
C	2.537737	-1.291556	-0.134241
H	4.009638	-1.866163	1.522826
H	3.705473	-3.117998	0.243995
H	3.160063	-0.461400	-2.180329
H	3.197592	-2.267661	-2.000757
C	5.818249	-2.044104	-1.173614
H	6.252351	-1.520795	-2.047900
H	6.619189	-2.152244	-0.416388
H	5.525878	-3.060235	-1.500548

C	1.133047	-1.854334	-0.005591
H	0.876772	-2.491154	-0.878837
H	1.058805	-2.492349	0.903720
C	2.531979	0.123247	0.361222
O	1.369328	0.641899	0.659372

XII-q

C	2.923121	-0.681119	-0.156921
C	1.928684	-2.831654	-0.142585
C	3.130425	-3.459980	-0.388206
C	4.253621	-2.648010	-0.694851
C	4.143650	-1.270096	-0.632138
C	2.673863	0.706727	0.111424
C	0.971241	2.353277	0.212851
C	1.871634	3.364155	0.471782
C	3.227608	3.009273	0.681599
C	3.624430	1.689451	0.549475
H	0.998069	-3.401790	-0.007314
H	3.198827	-4.551706	-0.422352
H	5.178470	-3.118753	-1.042483
H	-0.107157	2.547669	0.127401
H	1.538493	4.400926	0.580122
H	3.937943	3.773333	1.012013
N	1.813627	-1.499209	-0.059105
N	1.347886	1.078016	0.055424
O	5.090148	-0.425595	-1.079857
O	4.841734	1.240535	0.902427
C	6.328335	-0.938459	-1.483270
H	6.823008	-1.500265	-0.665677
H	6.231135	-1.603952	-2.365078
H	6.957556	-0.076357	-1.756386
C	5.824970	2.159403	1.287653
H	6.741376	1.581153	1.486202
H	6.031539	2.896180	0.485704
H	5.539715	2.705758	2.209530
Ni	0.106564	-0.471994	-0.099728

C	-3.949528	0.558440	-0.783607
C	-4.158670	1.330626	-1.928469
C	-5.031199	0.006585	-0.063655
C	-5.456077	1.574216	-2.366957
H	-3.291249	1.734410	-2.462674
C	-6.324397	0.252471	-0.518599
C	-6.531139	1.032910	-1.659618
H	-5.634484	2.182622	-3.260156
H	-7.187973	-0.162771	0.012083
H	-7.555371	1.219400	-2.002952
C	-4.678568	-0.813219	1.155607
C	-3.499872	-1.750937	0.780282
C	-3.638031	0.003876	1.960366
C	-2.538647	-0.540960	1.001018
H	-3.517290	-2.246793	-0.209621
H	-3.325226	-2.505020	1.571240
H	-3.779347	1.098919	2.041464
H	-3.484461	-0.429158	2.967209
C	-5.853126	-1.406989	1.893338
H	-6.540424	-0.623729	2.266205
H	-6.436886	-2.093605	1.250693
H	-5.497826	-1.984531	2.766913
C	-1.117133	-0.742079	1.467992
H	-0.840346	0.073574	2.170172
H	-1.017289	-1.697667	2.020704
C	-2.604208	0.238721	-0.287778
O	-1.582021	0.530879	-0.916970

VI-d-a

C	1.761292	0.870812	0.350649
C	-0.197169	1.538554	1.423868
C	0.340649	2.787183	1.711657
C	1.592346	3.118862	1.213355
C	2.305953	2.169060	0.471814
C	2.425480	-0.261894	-0.310891
C	2.015770	-2.152056	-1.609392

C	3.377203	-2.370849	-1.791103
C	4.284982	-1.566291	-1.116203
C	3.814434	-0.519084	-0.312197
H	-1.211735	1.266002	1.734189
H	-0.240168	3.511494	2.290243
H	1.998123	4.121648	1.375027
H	1.256400	-2.801544	-2.056754
H	3.723542	-3.194009	-2.422916
H	5.356815	-1.771536	-1.192620
N	0.494596	0.614889	0.750246
N	1.568492	-1.132869	-0.877964
O	3.436027	2.430249	-0.181182
O	4.584823	0.205756	0.495670
C	4.091862	3.660920	0.017173
H	3.481311	4.508243	-0.349503
H	4.336594	3.815935	1.085181
H	5.025158	3.613836	-0.563906
C	5.986630	0.097091	0.411484
H	6.399560	0.843307	1.106881
H	6.333773	-0.909147	0.715056
H	6.343648	0.318367	-0.612381
Ni	-0.253198	-0.983841	-0.079523
C	-1.886905	-0.894117	0.863613
C	-1.878285	-1.542218	2.111185
C	-3.048961	-0.194655	0.474167
C	-2.966965	-1.499435	2.980406
H	-0.983608	-2.101408	2.420293
C	-4.134694	-0.150048	1.370028
C	-4.106726	-0.788390	2.605934
H	-2.924343	-2.014391	3.948809
H	-5.039510	0.412659	1.101448
H	-4.974648	-0.728125	3.273956
C	-3.184888	0.582447	-0.826914
C	-4.550998	0.466508	-1.580557
C	-3.476093	2.113088	-0.670569
C	-4.573023	1.971369	-1.696814
H	-5.370520	0.073632	-0.950409

H	-4.538099	-0.116850	-2.521886
H	-3.864413	2.366260	0.335464
H	-2.640342	2.802641	-0.903450
C	-2.065058	0.383763	-1.835009
H	-1.088127	0.732164	-1.446214
H	-1.968769	-0.676518	-2.131749
H	-2.275833	0.974652	-2.748536
C	-5.206546	2.827299	-2.495552
H	-5.012210	3.907954	-2.444905
H	-5.944094	2.482090	-3.233794
O	-0.879742	-2.579778	-0.978034
C	-0.661326	-3.680751	-0.430416
O	-0.126654	-4.064598	0.580401

IX-d-a

C	3.453424	0.497575	0.049896
C	2.997074	2.770508	0.259680
C	4.309127	3.046569	0.626012
C	5.238947	2.013740	0.622728
C	4.828665	0.717224	0.288335
C	2.844342	-0.818232	-0.235673
C	1.160349	-1.877792	-1.448184
C	1.512614	-3.121333	-0.935402
C	2.524395	-3.194577	0.013723
C	3.184921	-2.024448	0.413819
H	2.243523	3.565052	0.190320
H	4.610032	4.067324	0.880082
H	6.287303	2.225635	0.852471
H	0.315539	-1.764039	-2.138837
H	0.969691	-4.018832	-1.248044
H	2.775378	-4.158198	0.466791
N	2.598174	1.531619	-0.021671
N	1.816157	-0.772480	-1.100469
O	5.658601	-0.311849	0.121432
O	4.064054	-1.967437	1.415179
C	7.021081	-0.167365	0.445934

H	7.515712	0.577332	-0.206946
H	7.152063	0.126648	1.504905
H	7.486730	-1.151186	0.285049
C	4.462847	-3.152764	2.061464
H	5.207186	-2.860875	2.817517
H	3.609356	-3.644920	2.566363
H	4.928501	-3.861792	1.350551
Ni	0.864337	1.027523	-1.001291
C	-2.533166	0.770364	1.300584
C	-3.199378	0.100285	2.332633
C	-2.957864	2.051626	0.870663
C	-4.278964	0.680073	2.990303
H	-2.860738	-0.902917	2.621040
C	-4.058955	2.598794	1.544052
C	-4.706439	1.939851	2.588292
H	-4.783593	0.146293	3.802975
H	-4.444366	3.577692	1.245677
H	-5.561088	2.418950	3.079477
C	-2.324146	2.807959	-0.290503
C	-0.838197	3.273502	-0.093680
C	-1.869102	1.953074	-1.515523
C	-0.461209	2.504048	-1.348333
H	-0.372800	2.905560	0.836881
H	-0.701876	4.371069	-0.150010
H	-1.995087	0.858645	-1.422255
H	-2.365202	2.238689	-2.462924
C	-3.160271	3.981242	-0.779220
H	-4.171493	3.663013	-1.097738
H	-3.262926	4.771983	-0.012859
H	-2.663001	4.444229	-1.652606
C	0.565898	2.586997	-2.273147
H	0.405371	2.216597	-3.297801
H	1.386914	3.310032	-2.150300
O	-1.605170	-1.125685	0.190869
C	-1.369954	0.032207	0.715775
O	-0.234378	0.500873	0.769152
Al	-2.969146	-2.103225	-0.427553

Cl	-4.540065	-0.800805	-1.092383
Cl	-3.615305	-3.413189	1.143449
Cl	-2.159634	-3.224044	-2.075119

IX-d-b

C	2.470033	-0.691618	-0.249339
C	1.077589	-2.406062	-0.978926
C	2.159411	-3.163538	-1.412117
C	3.446035	-2.698043	-1.166183
C	3.621243	-1.462373	-0.531523
C	2.483365	0.673691	0.314693
C	1.320917	2.142636	1.699870
C	2.261015	3.145822	1.497899
C	3.307261	2.920501	0.610226
C	3.406709	1.684084	-0.039205
H	0.045803	-2.765546	-1.085300
H	1.999579	-4.127274	-1.904945
H	4.308361	-3.311662	-1.443110
H	0.427351	2.299792	2.315436
H	2.158364	4.111804	2.001766
H	4.021496	3.721665	0.398660
N	1.242642	-1.212810	-0.411068
N	1.447800	0.947731	1.124112
O	4.799080	-0.992485	-0.119776
O	4.275269	1.420571	-1.017052
C	5.981200	-1.668180	-0.475981
H	6.020507	-2.679843	-0.027959
H	6.081732	-1.748548	-1.575364
H	6.815460	-1.069049	-0.081061
C	5.247397	2.377655	-1.362338
H	5.863012	1.926092	-2.154874
H	4.783104	3.304778	-1.750544
H	5.894267	2.624307	-0.498738
Ni	-0.150594	-0.291242	0.835503
C	-3.527166	1.137335	-0.626948
C	-4.101709	2.146616	-1.406569

C	-4.222965	-0.079559	-0.416102
C	-5.331954	1.977168	-2.035962
H	-3.553491	3.091008	-1.521691
C	-5.467574	-0.217067	-1.050089
C	-6.015619	0.779586	-1.856862
H	-5.754447	2.778457	-2.653566
H	-6.048386	-1.134269	-0.911958
H	-6.989740	0.616696	-2.333043
C	-3.700830	-1.200454	0.477502
C	-2.399586	-1.928246	-0.012052
C	-2.933896	-0.746491	1.759561
C	-1.699030	-1.482363	1.262684
H	-1.979149	-1.511056	-0.944404
H	-2.523264	-3.023380	-0.131760
H	-2.843047	0.342794	1.913853
H	-3.359495	-1.172333	2.689294
C	-4.758107	-2.232009	0.840544
H	-5.631332	-1.772870	1.343404
H	-5.117909	-2.794054	-0.041860
H	-4.321364	-2.974039	1.535904
C	-0.589495	-1.912109	1.983650
H	-0.544595	-1.707399	3.065270
H	0.025650	-2.757272	1.637131
O	-2.076183	2.340756	0.811151
C	-2.162925	1.458938	-0.049777
O	-1.201023	0.781909	-0.535057
